# Unveiling health inequalities and frontier gaps in elderly-onset rheumatoid arthritis: evolving impact of smoking and future challenges

**DOI:** 10.3389/fmed.2025.1664232

**Published:** 2025-10-09

**Authors:** Yan Gao, Chen Jia, Hailong Yu, Wenfeng Han, Ning Wang, Bin Zheng, Aoxiang Yang, Yu Wang

**Affiliations:** ^1^Department of Disease Prevention and Control, General Hospital of Northern Theater Command, Shenyang, China; ^2^Department of Orthopedics, General Hospital of Northern Theater Command, Shenyang, China

**Keywords:** rheumatoid arthritis, global burden of disease study, incidence, prevalence, mortality, disability-adjusted life years

## Abstract

**Background:**

Elderly-onset rheumatoid arthritis (EORA) poses a growing public health burden worldwide. Significant health inequalities and frontier gaps persist across countries, while the impact of smoking on EORA has evolved over time.

**Methods:**

Using data from the 2021 Global Burden of Disease data, we assessed the incidence, prevalence, mortality, and disability-adjusted life years (DALYs) of EORA and analyzed trends by calculating the estimated annual percentage changes. We explored associations with the sociodemographic index (SDI), evaluated frontier gaps, quantified health inequalities, examined the impact of smoking, and predicted trends to 2050 using Bayesian age-period-cohort (BAPC) models.

**Results:**

In 2021, global EORA case numbers were as follows: incidence: 0.33 million (95% uncertainty interval [UI]: 0.22, 0.47), prevalence: 7.92 million (95% UI: 6.90, 9.10), mortality: 33.20 thousand (95% UI: 26.86, 38.57), and DALYs: 1.55 million (95% UI: 1.23, 1.93). The disease burden was higher in females than in males, with high SDI-regions such as Australia experiencing the highest burden. Trends varied across different regions and countries; India and China had the highest case numbers, whereas Guam and Singapore showed significant improvements in mortality rates. Cross-national inequality analysis revealed significant disparities in disease burden. Frontier analysis identified considerable potential for improvement in disease burden in several countries and regions. The impact of smoking on EORA has declined, but BAPC model projections indicate that the burden will continue to rise until 2050.

**Conclusion:**

Elderly-onset rheumatoid arthritis has become a significant public health concern. Addressing socio-economic inequalities, enhancing monitoring systems, and implementing targeted prevention and treatment strategies are crucial for alleviating the global EORA burden.

## 1 Introduction

Rheumatoid arthritis (RA) is a chronic autoimmune disease characterized by symmetric polyarthritis (1). It often causes chronic pain and joint deformities, imposing a lifelong burden on patients, with approximately 18 million people affected globally (2, 3). Additionally, RA negatively impacts mental health, potentially leading to social isolation and a decline in quality of life (4), while also imposing a substantial economic burden by increasing healthcare demand (5). Over the past three decades, the global incidence of RA has steadily increased at an average annual rate of 0.21%, with a higher burden in regions with a higher sociodemographic index (SDI) and a significantly higher prevalence in females compared to males (6, 7). As age increases, the prevalence of RA continues to rise, and with the accelerating global population aging, the burden of RA in the older adult population is increasingly becoming pronounced (8).

Elderly-onset RA (EORA) is an age-related category of RA, defined as the onset of RA symptoms in individuals aged 60 years or older (9). Patients with EORA typically present with more severe disease activity at the time of diagnosis compared to that observed with patients with younger-onset RA (YORA) and are at a higher risk of disability (10, 11). Furthermore, patients with EORA often exhibit a higher inflammatory burden, making them more susceptible to chronic joint damage and functional loss (9). Due to the frequent occurrence of complex comorbidities (such as pulmonary diseases and osteoporosis) in patients with EORA (12), treatment in such patients is more challenging and requires a comprehensive approach to address multiple health issues to develop individualized treatment plans (13, 14). Given the increasing global disease burden of EORA and its significant public health implications, a thorough analysis based on existing epidemiological data is critically important.

Previous studies on the global, regional, and national disease burden of RA, particularly regarding systematic research on EORA, are limited. This study, based on the 2021 Global Burden of Disease (GBD) data, analyzed the disease burden of EORA and its trends from 1990 to 2021. We systematically assessed the incidence, prevalence, mortality, and disability-adjusted life years (DALYs) associated with EORA and employed cross-national health inequality analysis to evaluate health disparities across different regions and countries. Through advanced analysis, we estimated the improvement potential in reducing the EORA burden in various countries based on the SDI, providing corresponding benchmark standards. Additionally, we quantified the impact of smoking on EORA and projected its development trends until 2050, aiming to reveal potential future public health challenges. By performing in-depth analysis of global epidemiological differences, this study provides scientific evidence to support public health decisions, policy formulation, and the optimal allocation of health resources.

## 2 Methods

### 2.1 Data source

The 2021 GBD study provides comprehensive epidemiological data from 1990 to 2021, aiming to quantify the mortality and morbidity caused by 369 diseases and injuries (15). The 2021 GBD study utilized the Bayesian meta-regression tool DisMod-MR 2.1 to estimate multiple dimensions of RA disease burden, including age, sex, year, and region. The study design and methods have been extensively detailed in the existing GBD literature (16). This study strictly adhered to the “Guidelines for Accurate and Transparent Health Estimates Reporting” (17).

We extracted data on RA incidence, prevalence, mortality, DALYs, and age-standardized rates (ASRs) from the GBD study results tool (https://vizhub.healthdata.org/gbd-results/) for the years 1990–2021. This data covers 204 countries and territories, 21 GBD regions, and 5 SDI regions (low, lower-middle, middle, upper-middle, and high SDI) at both global and regional levels. The 95% uncertainty intervals (UI) for each metric were determined by calculating the 2.5th and 97.5th percentiles of 1,000 sampling values. The SDI is a composite index based on female per capita income, average years of schooling, and fertility rates for females aged under 25 years, with a score ranging from 0 (lowest development) to 1 (highest development) (18).

### 2.2 Definition of elderly-onset rheumatoid arthritis and the study population

Elderly-onset rheumatoid arthritis is defined as RA onset at 60 years of age or older (9). However, the GBD study estimates incidence and prevalence based on current age groups rather than exact age at onset, which may include cases where RA onset occurred before age 60 but persisted into older age. In the 2021 GBD study, RA cases were defined based on the 1987 American College of Rheumatology classification criteria (19), with diagnoses aligning with the International Classification of Diseases, 10th Edition codes, including M05-M05.9 and M08-M09.8. In this study, the target population was individuals aged 60 years and older, categorized into eight age subgroups (60–64, 65–69, …, 90–94, and ≥95 years), serving as a proxy for EORA burden.

### 2.3 Statistical analysis

#### 2.3.1 Descriptive analysis

This study evaluated the disease burden of EORA across dimensions such as age, sex, year, and region, including age-standardized incidence (ASIR), prevalence (ASPR), mortality (ASMR), and DALYs (ASDR) rates, along with crude case numbers, to provide a more comprehensive epidemiological profile. ASRs (per 100,000 population) for specific populations were calculated using direct standardization based on the world standard population defined by the 2021 GBD (20). We computed the estimated annual percentage change (EAPCs) and 95% confidence intervals (CI) for ASIR, ASPR, ASMR, and ASDR at the global, regional, and national levels to assess trends from 1990 to 2021 (21). Additionally, we used local regression smoothing (loess) to model the correlation between EORA burden and SDI across 21 regions and 204 countries and regions. We further conducted Spearman’s correlation analysis to calculate the *r*-value and *p*-value for the relationship between EORA burden and SDI, with *p* < 0.05 considered statistically significant.

#### 2.3.2 Cross-country inequality analysis

We used the slope inequality index (SII) and concentration index defined by the World Health Organization to quantify the distributional inequalities of EORA burden across 204 countries and territories. A robust regression model was employed to effectively control for bias and heterogeneity, ensuring a more accurate assessment of health inequalities. The SII measures the relationship between the EORA burden and SDI through regression analysis, where the relative position scale associated with the SDI is determined by the midpoint of the cumulative population distribution ranked by the SDI. The concentration index is calculated based on the Lorenz concentration curve by matching the cumulative proportion of EORA burden with the cumulative population distribution ranked by the SDI (22). The final index is derived by numerically integrating the area under the curve (23).

#### 2.3.3 Frontier analysis

To comprehensively assess the relationship between the EORA burden and sociodemographic development levels, we employed frontier analysis using the SDI to construct frontier models based on ASRs. This method identifies high-performing countries and regions that can serve as benchmarks for others to improve their EORA disease burden (24). By measuring the absolute distance (i.e., effective difference) between each country’s 2021 EORA burden and the frontier boundary, we evaluated their potential for reducing the disease burden.

#### 2.3.4 Risk factor analysis

In the 2021 GBD study, smoking was the only identified risk factor for RA. This study assessed the impact of smoking on ASMR and ASDR related to EORA across 204 countries and regions, 21 GBD regions, 5 SDI subgroups, and globally from 1990 to 2021.

#### 2.3.5 Predictive analysis

We used the Bayesian age-period-cohort (BAPC) model to project EORA case numbers and ASRs by sex until 2050. This model accounts for the complex interactions between age, period, and cohort effects (25). All statistical analyzes and visualizations were conducted using R (version 4.4.0; R Foundation for Statistical Computing, Vienna, Austria).

## 3 Results

### 3.1 Descriptive analysis

Between 1990 and 2021, the global burden of EORA increased significantly. In 2021, 0.33 million (95% UI: 0.22, 0.47) new EORA cases and 7.92 million (95% UI: 6.90, 9.10) prevalent cases were estimated. In the same year, EORA-related deaths totaled 33.20 thousand (95% UI: 26.86, 38.57), resulting in 1.55 million (95% UI: 1.23, 1.93) lost DALYs (Supplementary Table 1). Regarding ASRs per 100,000 population, the global ASIR was 30.66 (95% UI: 19.93, 43.01), ASPR was 726.35 (95% UI: 632.64, 835.35), ASMR was 3.05 (95% UI: 2.43, 3.57), and ASDR was 142.16 (95% UI: 112.24, 177.73). Between 1990 and 2021, ASIR and ASPR showed upward trends, with EAPCs of 0.69 (95% CI: 0.65, 0.73) and 0.55 (95% CI: 0.50, 0.60), respectively (Supplementary Figure 2 and Supplementary Tables 2–5). Conversely, ASMR and ASDR exhibited downward trends, with EAPCs of −0.51 (95% CI: −0.61, −0.40) and −0.02 (95% CI: −0.08, 0.03), respectively (Table 1). By sex, females compared to males experienced a significantly higher global EORA burden, particularly in the prevalence rates (Table 1).

**TABLE 1:** Global and regional EAPCs of age-standardiszed rate (ASR) for elderly-onset rheumatoid arthritis (EORA) (1990–2021).

Category	Incidence			Prevalence			Mortality			DALYs		
	ASR in 1990 (95% UI)	ASR in 2021 (95% UI)	EAPCs (95% CI)	ASR in 1990 (95% UI)	ASR in 2021 (95% UI)	EAPCs (95% CI)	ASR in 1990 (95% UI)	ASR in 2021 (95% UI)	EAPCs (95% CI)	ASR in 1990 (95% UI)	ASR in 2021 (95% UI)	EAPCs (95% CI)
Global	25.49 (16.12, 36.43)	30.66 (19.93, 43.01)	0.69 (0.65, 0.73)	632.28 (547.04, 734.20)	726.35 (632.64, 835.35)	0.55 (0.50, 0.60)	3.68 (3.15, 4.23)	3.05 (2.43, 3.57)	−0.51 (−0.61, −0.40)	147.21 (119.91, 178.76)	142.16 (112.24, 177.73)	−0.02 (−0.08, 0.03)
Men	18.83 (11.84, 26.96)	22.74 (14.82, 31.72)	0.70 (0.66, 0.75)	369.44 (312.25, 438.86)	442.08 (379.4, 517.25)	0.69 (0.64, 0.73)	2.30 (1.63, 2.78)	2.15 (1.34, 2.66)	−0.07 (−0.18, 0.04)	90.27 (71.11, 112.57)	92.45 (70.52, 116.41)	0.19 (0.13, 0.25)
Females	30.93 (19.58, 44.22)	37.49 (24.29, 52.78)	0.71 (0.68, 0.75)	846.96 (737.31, 974.98)	971.46 (850.45, 1111.02)	0.54 (0.50, 0.59)	4.81 (4.11, 5.61)	3.82 (3.05, 4.64)	−0.65 (−0.76, −0.55)	193.72 (158.16, 235.47)	185.02 (143.93, 232.28)	−0.07 (−0.12, −0.02)
**SDI regions**												
Low SDI	15.75 (9.57, 23.27)	22.13 (13.90, 31.58)	1.23 (1.15, 1.32)	288.89 (242.96, 343.37)	406.23 (346.36, 478.70)	1.23 (1.10, 1.35)	2.09 (1.17, 3.86)	2.20 (1.35, 3.82)	0.49 (0.32, 0.66)	76.48 (55.82, 107.45)	91.15 (68.43, 121.97)	0.73 (0.65, 0.82)
Low-middle SDI	23.87 (14.83, 34.51)	33.55 (21.47, 47.42)	1.17 (1.14, 1.21)	405.67 (339.88, 485.95)	608.19 (517.91, 717.97)	1.45 (1.39, 1.50)	4.04 (2.60, 5.91)	4.17 (2.90, 5.99)	0.25 (0.11, 0.38)	127.02 (95.11, 161.67)	149.38 (115.53, 189.94)	0.62 (0.55, 0.69)
Middle SDI	20.10 (11.76, 30.24)	27.55 (17.12, 39.95)	1.07 (1.06, 1.09)	514.32 (436.36, 605.68)	651.56 (560.12, 760.45)	0.85 (0.82, 0.89)	3.24 (2.52, 3.94)	3.03 (2.25, 3.60)	−0.01 (−0.24, 0.23)	125.29 (100.89, 155.86)	133.72 (104.91, 167.27)	0.34 (0.24, 0.44)
High-middle SDI	16.26 (9.79, 24.38)	22.43 (14.18, 32.21)	1.11 (1.08, 1.15)	517.32 (447.61, 597.81)	644.94 (563.32, 737.48)	0.77 (0.74, 0.80)	2.39 (2.03, 2.80)	2.41 (1.89, 2.89)	0.10 (−0.07, 0.28)	112.10 (90.26, 139.33)	122.08 (94.73, 155.12)	0.32 (0.26, 0.38)
High SDI	40.66 (26.80, 56.68)	42.23 (28.53, 58.09)	0.22 (0.17, 0.27)	1003.19 (876.99, 1154.46)	1036.19 (916.72, 1176.48)	0.21 (0.13, 0.29)	5.31 (4.73, 5.71)	3.14 (2.58, 3.50)	−1.79 (−1.94, −1.64)	218.81 (177.77, 267.24)	177.61 (135.78, 226.44)	−0.64 (−0.68, −0.60)
**GBD regions**												
East Asia	23.34 (13.24, 36.12)	29.93 (18.44, 43.87)	0.82 (0.81, 0.83)	640.86 (541.20, 760.85)	745.40 (638.19, 872.07)	0.54 (0.51, 0.58)	3.39 (2.61, 4.41)	3.31 (2.24, 4.18)	0.26 (−0.14, 0.66)	147.83 (117.08, 188.18)	149.64 (114.58, 190.71)	0.21 (0.05, 0.38)
South Asia	35.04 (21.95, 50.63)	50.74 (32.37, 71.81)	1.25 (1.23, 1.28)	526.77 (434.98, 640.71)	811.35 (680.68, 970.40)	1.53 (1.45, 1.62)	5.34 (3.44, 7.89)	5.06 (3.54, 7.55)	−0.07 (−0.18, 0.05)	166.60 (123.71, 213.67)	190.05 (147.07, 244.79)	0.49 (0.43, 0.54)
Southeast Asia	6.04 (3.34, 9.34)	8.86 (5.18, 13.18)	1.12 (1.05, 1.18)	159.93 (133.57, 191.06)	230.53 (196.29, 270.10)	1.18 (1.15, 1.20)	1.18 (0.61, 1.71)	1.13 (0.64, 1.51)	−0.12 (−0.32, 0.09)	41.63 (30.07, 54.34)	48.62 (35.82, 62.88)	0.46 (0.40, 0.53)
Central Asia	6.29 (3.29, 10.30)	8.43 (4.49, 13.41)	1.02 (0.92, 1.12)	295.48 (257.78, 338.46)	415.52 (368.96, 467.33)	1.22 (1.00, 1.44)	0.17 (0.06, 0.43)	0.93 (0.62, 1.31)	5.14 (3.78, 6.52)	40.76 (26.74, 58.43)	69.43 (50.64, 93.43)	2.05 (1.79, 2.30)
High-income Asia Pacific	45.71 (27.38, 68.76)	34.11 (21.17, 49.46)	−0.82 (−0.89, −0.75)	1206.64 (1014.01, 1439.27)	1001.75 (864.53, 1163.50)	−0.41 (−0.53, −0.29)	5.97 (5.06, 6.76)	4.05 (3.06, 4.77)	−1.75 (−2.31, −1.20)	261.80 (208.55, 326.42)	184.59 (141.12, 235.64)	−1.16 (−1.25, −1.06)
Oceania	2.68 (0.45, 7.09)	3.05 (0.93, 6.42)	0.40 (0.29, 0.50)	97.81 (72.23, 129.47)	115.48 (91.69, 144.33)	0.46 (0.39, 0.54)	0.01 (0.00, 1.18)	0.00 (0.00, 0.48)	−2.70 (−3.06, −2.33)	13.35 (5.62, 25.04)	15.28 (7.42, 26.47)	0.36 (0.28, 0.43)
Australasia	66.83 (42.71, 94.70)	72.43 (47.73, 101.99)	0.38 (0.29, 0.46)	1402.43 (1217.45, 1605.86)	1499.59 (1312.25, 1707.94)	0.24 (0.10, 0.38)	6.76 (4.97, 8.82)	3.97 (2.77, 5.30)	−1.83 (−1.99, −1.68)	294.73 (229.04, 373.45)	248.53 (183.06, 325.62)	−0.55 (−0.61, −0.50)
Eastern Europe	3.20 (1.46, 5.57)	3.78 (1.80, 6.42)	0.52 (0.26, 0.79)	335.85 (295.18, 381.84)	395.89 (352.04, 444.59)	0.50 (0.43, 0.57)	1.72 (1.47, 1.95)	2.14 (1.81, 2.50)	0.09 (−0.59, 0.78)	81.24 (66.54, 98.99)	93.54 (77.24, 113.82)	0.14 (−0.17, 0.46)
Western Europe	41.18 (27.71, 56.83)	45.17 (30.98, 61.43)	0.36 (0.31, 0.41)	962.00 (839.67, 1107.05)	1039.52 (913.75, 1179.98)	0.31 (0.25, 0.37)	5.48 (4.82, 6.02)	3.13 (2.53, 3.56)	−1.61 (−1.92, −1.31)	212.09 (172.03, 258.98)	176.32 (134.18, 226.28)	−0.48 (−0.56, −0.39)
Central Europe	7.86 (4.01, 12.72)	9.83 (5.27, 15.58)	0.87 (0.78, 0.96)	488.88 (429.10, 556.74)	578.68 (511.26, 652.90)	0.62 (0.58, 0.65)	3.06 (2.64, 3.49)	1.52 (1.22, 1.82)	−2.59 (−2.98, −2.20)	123.29 (102.68, 148.27)	100.61 (76.81, 129.57)	−0.77 (−0.97, −0.57)
High-income North America	39.72 (25.86, 55.45)	48.15 (32.24, 65.98)	0.75 (0.71, 0.79)	946.07 (833.71, 1076.65)	1100.61 (977.37, 1240.71)	0.60 (0.55, 0.64)	3.75 (3.22, 4.17)	2.67 (2.18, 3.08)	−1.41 (−2.06, −0.75)	185.57 (146.81, 230.70)	179.26 (136.02, 228.61)	−0.17 (−0.36, 0.03)
Andean Latin America	21.67 (13.73, 31.42)	32.32 (20.50, 46.55)	1.28 (1.22, 1.35)	601.18 (526.94, 682.37)	1057.76 (940.74, 1184.77)	1.92 (1.87, 1.96)	5.66 (3.45, 8.29)	3.98 (2.64, 5.71)	−1.31 (−1.55, −1.06)	157.25 (117.90, 202.41)	193.18 (143.04, 250.93)	0.60 (0.49, 0.71)
Central Latin America	34.01 (20.42, 50.61)	33.70 (20.97, 49.10)	0.08 (0.02, 0.13)	884.64 (760.50, 1028.87)	1046.32 (922.11, 1190.17)	0.54 (0.49, 0.59)	8.59 (7.45, 9.71)	5.88 (4.82, 6.97)	−1.11 (−1.29, −0.93)	251.18 (211.28, 297.76)	233.23 (188.50, 286.63)	−0.21 (−0.29, −0.12)
Caribbean	15.27 (9.98, 21.79)	18.84 (12.80, 26.23)	0.64 (0.57, 0.72)	330.24 (284.41, 382.57)	465.64 (408.14, 529.99)	1.11 (1.03, 1.18)	3.08 (2.05, 4.36)	3.16 (2.18, 4.35)	−0.06 (−0.18, 0.07)	87.84 (67.76, 110.70)	103.53 (80.29, 131.00)	0.46 (0.40, 0.52)
Tropical Latin America	5.49 (2.57, 9.48)	5.67 (2.89, 9.36)	0.04 (−0.02, 0.10)	318.87 (270.93, 373.16)	319.03 (276.22, 368.88)	0.15 (0.09, 0.22)	1.46 (1.11, 1.85)	1.50 (1.18, 1.81)	0.38 (0.16, 0.60)	68.46 (53.37, 86.79)	68.44 (53.87, 86.20)	0.19 (0.10, 0.28)
Southern Latin America1	9.22 (11.91, 28.06)	26.31 (16.20, 38.83)	1.03 (0.98, 1.08)	478.51 (415.26, 552.04)	802.24 (713.21, 901.93)	1.61 (1.53, 1.68)	2.97 (2.19, 3.87)	2.93 (2.18, 3.76)	0.58 (0.21, 0.95)	115.72 (91.81, 144.34)	149.99 (114.76, 191.30)	1.04 (0.89, 1.19)
Eastern Sub-Saharan Africa1	0.94 (6.22, 16.78)	12.56 (7.44, 18.65)	0.45 (0.40, 0.50)	252.48 (213.26, 299.77)	289.25 (248.11, 336.47)	0.48 (0.41, 0.54)	0.23 (0.03, 2.05)	0.14 (0.02, 1.52)	−1.90 (−2.01, −1.78)	36.42 (23.56, 67.00)	39.25 (26.07, 63.40)	0.25 (0.20, 0.31)
Southern Sub-Saharan Africa	16.53 (8.69, 26.90)	17.57 (9.67, 27.74)	0.29 (0.16, 0.42)	629.06 (542.58, 729.53)	615.49 (534.47, 712.17)	0.07 (−0.07, 0.21)	4.42 (2.45, 6.58)	3.63 (2.50, 5.16)	−1.19 (−1.73, −0.64)	158.90 (119.92, 203.51)	144.34 (112.37, 182.92)	−0.58 (−0.83, −0.33)
Western Sub-Saharan Africa	5.45 (2.91, 8.65)	6.55 (3.73, 9.94)	0.59 (0.51, 0.67)	138.70 (113.15, 168.24)	171.39 (143.17, 204.12)	0.74 (0.59, 0.89)	0.06 (0.00, 0.18)	0.05 (0.01, 0.16)	0.62 (0.06, 1.18)	18.77 (11.91, 27.79)	22.80 (14.95, 33.08)	0.71 (0.58, 0.84)
North Africa and Middle East	3.38 (1.67, 5.59)	5.21 (2.75, 8.30)	1.46 (1.36, 1.55)	168.57 (145.89, 195.46)	279.77 (246.85, 317.07)	1.73 (1.68, 1.79)	0.87 (0.48, 1.42)	0.71 (0.45, 1.01)	−0.28 (−0.44, −0.13)	39.20 (28.82, 52.46)	49.08 (36.31, 64.85)	0.85 (0.77, 0.94)
Central Sub-Saharan Africa	10.16 (5.30, 16.32)	11.96 (6.58, 18.47)	0.54 (0.42, 0.65)	274.57 (234.14, 320.49)	342.16 (295.67, 396.03)	0.73 (0.60, 0.86)	0.34 (0.02, 3.75)	0.23 (0.01, 2.90)	−1.38 (−1.52, −1.23)	41.81 (24.72, 102.68)	47.91 (29.50, 95.09)	0.43 (0.32, 0.54)

EORA, elderly-onset rheumatoid arthritis; ASR, age-standardized rate; CI, confidence interval; EAPC, estimated annual percentage change; GBD, global burden of disease; SDI, sociodemographic index; UI, uncertainty interval.

**FIGURE 1:**
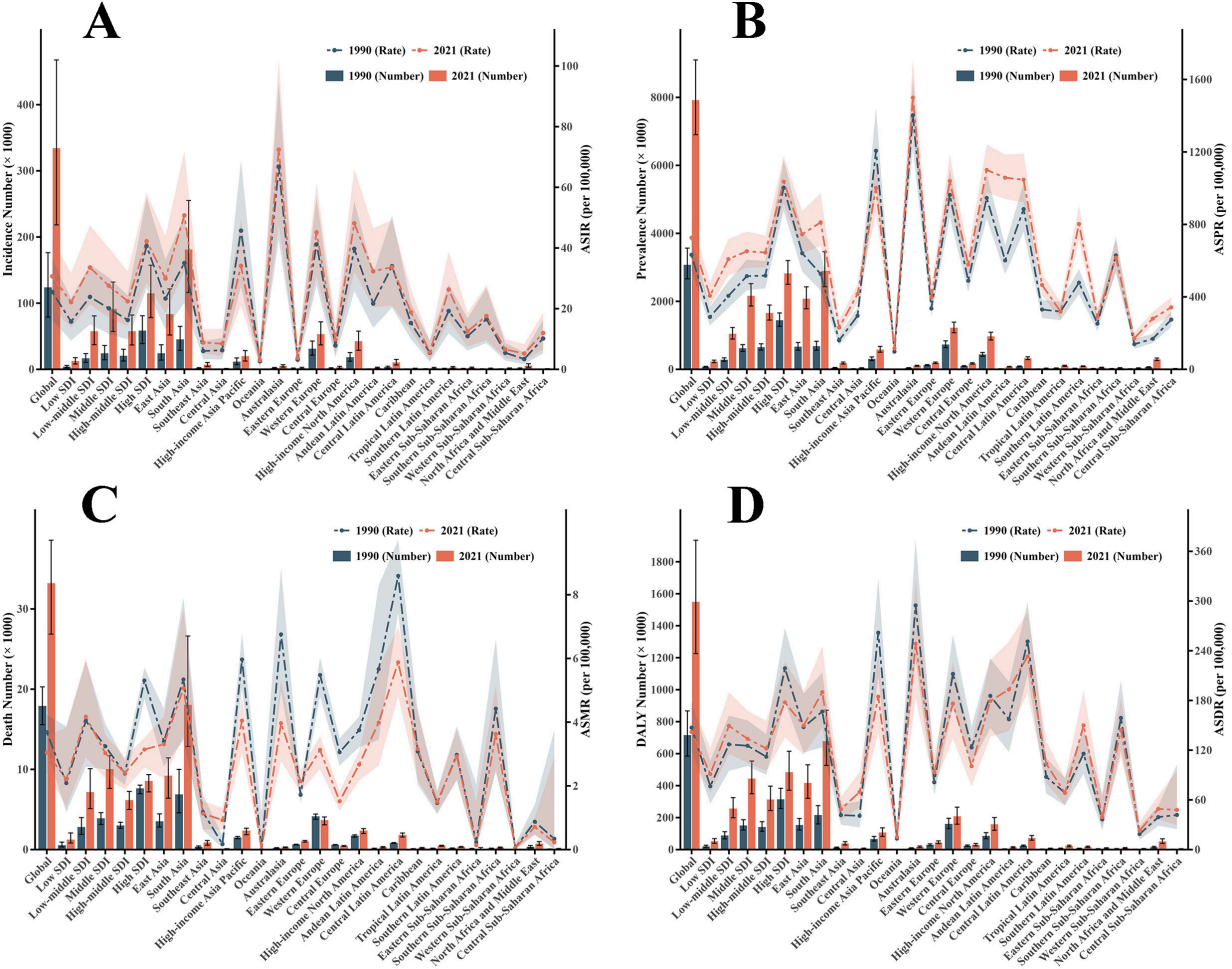
The number of cases and their ASRs for Incidence, Prevalence, Death, and DALYs. **(A)** Incidence; **(B)** Prevalence; **(C)** Mortality; **(D)** DALYs. ASR, age-standardized rate; DALYs, disability-adjusted life years; EORA, elderly-onset rheumatoid arthritis; SDI, sociodemographic index; GBD, global burden of disease.

In 2021, marked disparities in the burden of EORA existed across different SDI regions, with the highest burden observed in high SDI areas and the lowest in low SDI areas (Supplementary Table 1). Among the 21 GBD regions, Australasia reported the highest ASIR (30.66 per 100,000; 95% UI: 19.93, 43.01), ASPR (726.35 per 100,000; 95% UI: 632.64, 835.35), and ASDR (248.62 per 100,000; 95% UI: 183.75, 319.95). Central Latin America had the highest ASMR (5.88 per 100,000; 95% UI: 4.82, 6.97) (Figure 1 and Table 1). Between 1990 and 2021, ASIR and ASPR increased in most regions, with North Africa showing the largest increase in ASIR (EAPC: 1.09; 95% CI: 0.87, 1.31). Trends in ASMR and ASDR varied substantially across regions, with Central Asia experiencing the greatest increases in ASMR (EAPC: 5.14; 95% CI: 3.78, 6.52) and ASDR (EAPC: 2.05; 95% CI: 1.79, 2.05) (Supplementary Figure 1).

**FIGURE 2:**
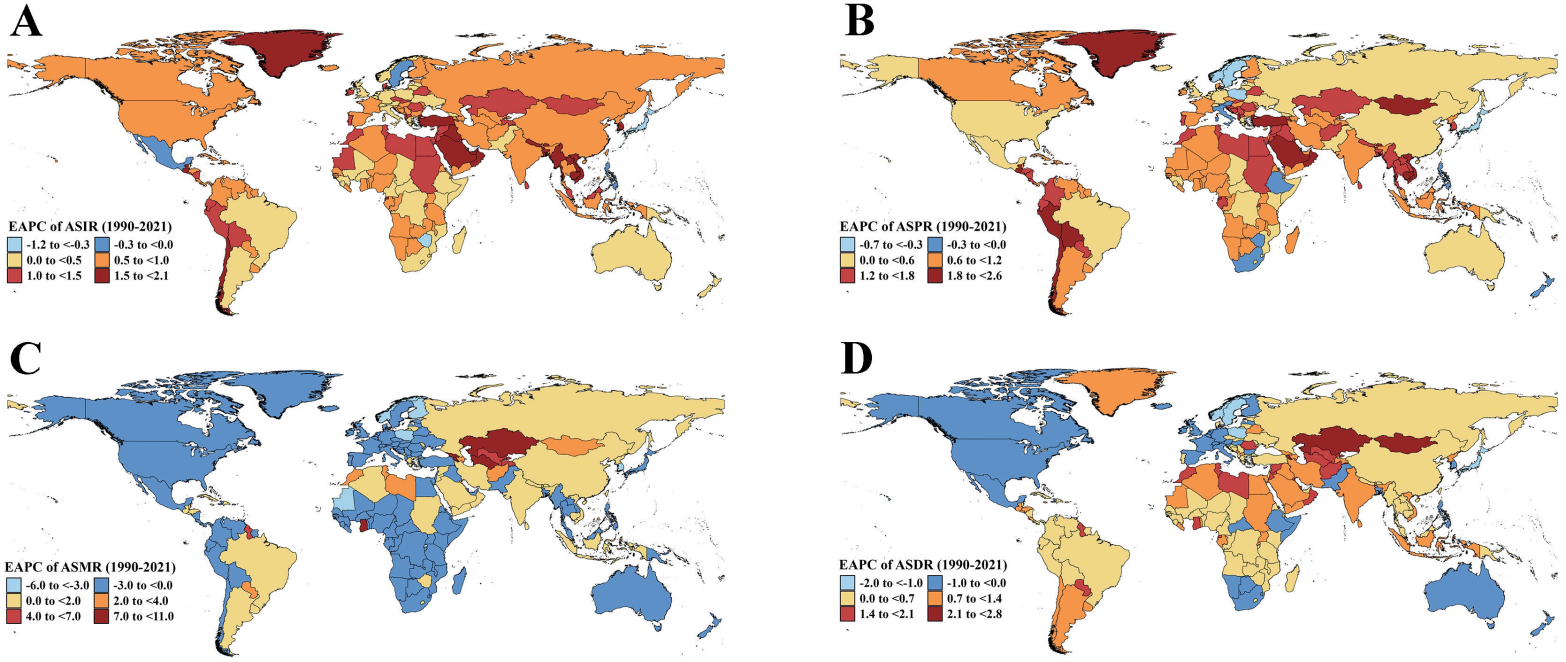
Estimated annual percentage change of ASR for EORA in 204 Countries and Territories (1990–2021). **(A)** EAPC of ASIR; **(B)** EAPC of ASPR; **(C)** EAPC of ASMR; **(D)** EAPC of ASDR. ASR, age-standardized rate; EORA, elderly-onset rheumatoid arthritis; EAPC, estimated annual percentage change; ASIR, age-standardized incidence rate; ASPR, age-standardized prevalence rate; ASMR, age-standardized mortality rate; ASDR, age-standardized disability-adjusted life years rate.

At the national level, India (84,459; 95% UI: 54,029, 119,228) and China (80,905; 95% UI: 49,797, 118,513) had the highest number of incident EORA cases in 2021. Regarding EORA-related deaths, China (8,900; 95% UI: 6,128, 11,094) and India (7,253; 95% UI: 5,145, 9,439) ranked first and second, respectively (Supplementary Tables 2–5). Between 1990 and 2021, among 204 countries and territories, ASIR increased in 197 countries, with Vietnam experiencing the largest increase (EAPC: 2.07; 95% CI: 1.94, 2.20), whereas Japan showed the greatest decline (EAPC: −1.03; 95% CI: −1.09, −0.97) (Figure 2A). Equatorial Guinea had the most significant increase in ASPR (EAPC: 2.52; 95% CI: 2.34, 2.71), whereas Norway experienced the largest reduction (EAPC: −0.62; 95% CI: −0.69, −0.54) (Figure 2B). During the observation period, EORA-related mortality improved in most countries, with Guam (EAPC: −5.86; 95% CI: −8.59, −3.06) and Singapore (EAPC: −4.80; 95% CI: −5.34, −4.26) showing the largest decreases (Figure 2C). Meanwhile, the ASDR increased in 157 countries, with Bahrain (EAPC: 2.80; 95% CI: 2.52, 3.08) and Armenia (EAPC: 2.62; 95% CI: 2.17, 3.07) exhibiting the highest increases (Figure 2D).

### 3.2 Age and sex patterns

**FIGURE 3:**
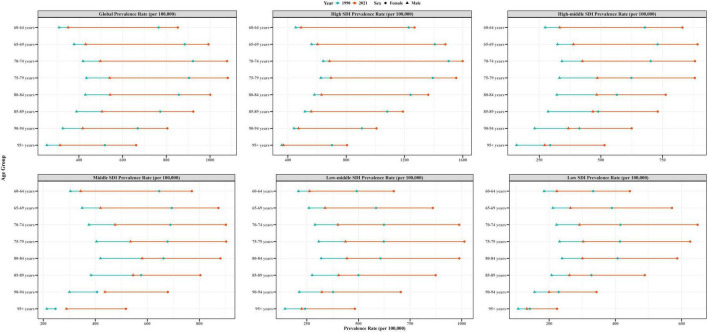
Age-standardized prevalence rates of EORA by sex, age group, and socio-demographic index; 1990 and 2021. EORA, elderly-onset rheumatoid arthritis.

In 2021, the ASPR of global EORA increased with age, peaking between 60 and 79 years (Figure 3). Across all age groups, females generally had higher ASIR, ASPR, ASMR, and ASDR than those had by males (Supplementary Figures 3–5). Between 1990 and 2021, the most significant increases in ASIR and ASPR were observed in the Andean Latin America region among individuals aged 60–69 years, with a faster increase in females than in males. In Central Asia, the largest increases in ASMR and ASDR occurred among females aged 60–89 years. Notably, in the high-income Asia Pacific region, females aged 60–79 years showed the most substantial decline in ASMR and ASDR (Supplementary Figure 6).

### 3.3 Association between elderly-onset rheumatoid arthritis burden and sociodemographic index

From 1990 to 2021, the relationship between EORA burden and SDI across global and regional levels (21 regions) was complex and non-linear, though generally positive, with the strongest correlation observed in ASPR (*r* = 0.65, *p* < 0.001). Overall, the global EORA burden was higher than expected. Among the 21 regions, Australasia and several others exceeded expectations, whereas Central Sub-Saharan Africa and Central Europe had a lower-than-expected burden (Supplementary Figure 7). At the national level, when SDI ranged between 0.4 and 0.7, the EORA burden remained relatively stable in most countries. Notably, the United Kingdom exhibited a higher-than-expected burden, whereas Niger and Chad had a lower-than-expected burden (Supplementary Figure 8).

### 3.4 Cross-country inequality analysis

**FIGURE 4:**
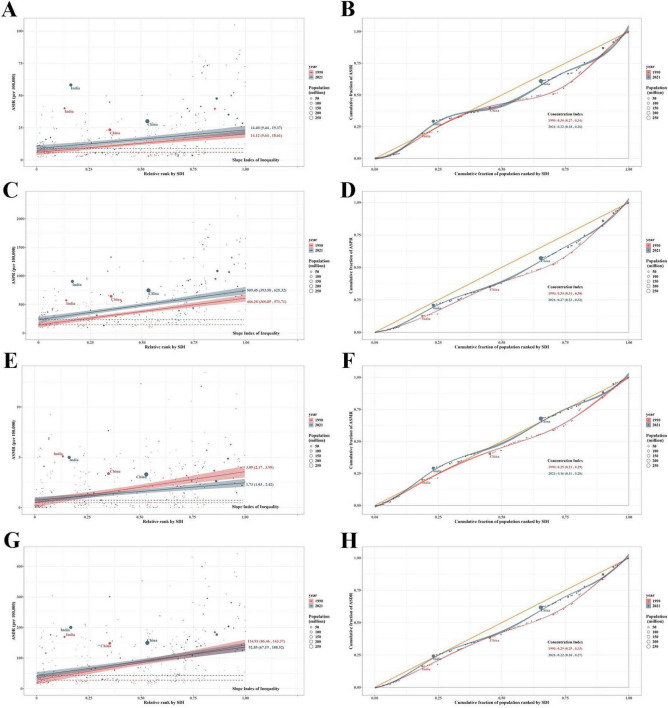
Health inequality regression curves and concentration curves for global EORA in 1990 and 2021. (**A**,**B**) Incidence; (**C**,**D**) Prevalence; (**E**,**F**) Mortality; (**G**,**H**) DALYs. DALYs, disability-adjusted life years; EORA, elderly-onset rheumatoid arthritis.

The burden of EORA exhibits significant inequalities in both absolute and relative terms across different SDI levels, with countries of higher SDI bearing a greater disease burden. In 2021, the SII revealed that the differences in incidence and prevalence between countries with the highest and lowest SDI were 14.40 (95% CI: 9.44, 19.37) and 509.45 (95% CI: 393.58, 625.32) per 100,000, respectively, marking a notable increase compared to the 1990 values (Figures 4A, C). In 2021, the concentration index values for incidence and prevalence were 0.22 (95% CI: 0.18, 0.26) and 0.27 (95% CI: 0.23, 0.32), respectively, reflecting a slight decrease from 1990 (Figures 4B, D). Regarding mortality and DALYs rates, both the SII and concentration index showed downward trends (Figures 4E–H). These results suggest a continued uneven distribution of disease burden across countries with varying SDI levels.

### 3.5 Frontier analysis

**FIGURE 5:**
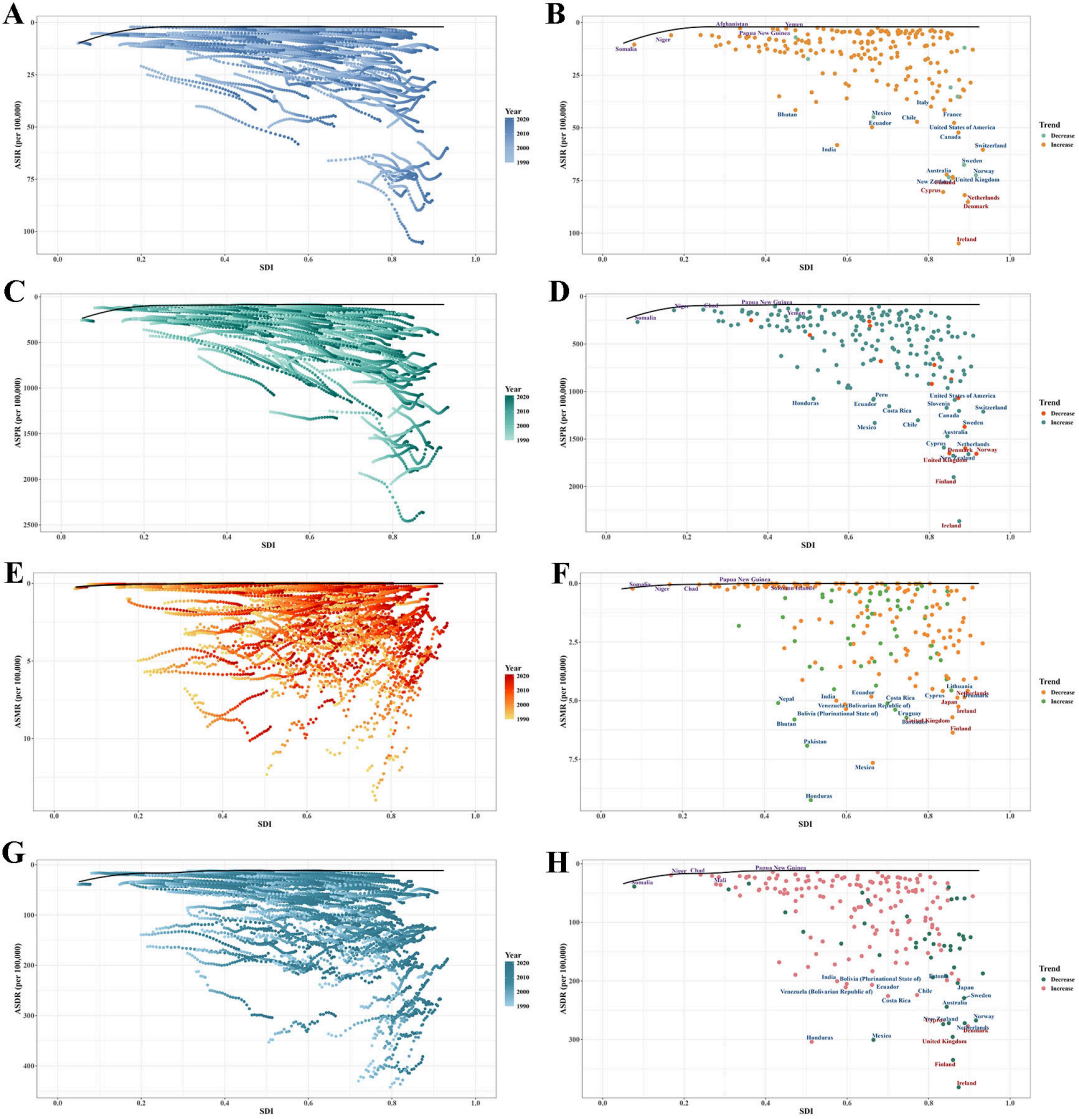
Frontier analysis explored the relationship between the sociodemographic index and ASRs of EORA. (**A**,**B**) Incidence; (**C**,**D**) Prevalence; (**E**,**F**) Mortality; (**G**,**H**) DALYs. ASR, age-standardised rate; DALYs, disability-adjusted life years; EORA, elderly-onset rheumatoid arthritis.

Between 1990 and 2021, a frontier analysis for EORA was conducted based on ASRs and SDI in 204 countries and territories. The results showed an upward trend for ASIR and ASPR but a downward trend for ASMR and ASDR (Figures 5A,C,E, G). Regarding the ASIR and ASPR, countries such as Australia, Switzerland, Canada, and the United States demonstrated substantial gaps relative to the frontier, indicating significant room for improvement (Supplementary Tables 6, 7). Conversely, lower SDI countries such as Somalia, Papua New Guinea, Niger, and Yemen exhibited relatively smaller gaps (Figures 5B, D), which may not reflect superior disease control but rather under-ascertainment due to limited healthcare access, diagnostic capabilities, and reporting systems. Regarding ASMR and ASDR, Honduras, India, and Costa Rica showed larger disparities from the frontier (Supplementary Tables 8, 9). Notably, high SDI countries including Ireland, Finland, and the United Kingdom, despite exhibiting declining trends in 1990–2021, still maintained considerable gaps relative to the frontier (Figures 5F, H).

### 3.6 Risk factors analysis

In 2021, the global number of deaths and DALYs attributable to smoking were 1,889 (95% UI: 1,223, 2,498) and 100,844 (95% UI: 70,615, 137,341), respectively. The corresponding ASMR and ASDR were 0.17 (95% UI: 0.10, 0.24) and 9.25 (95% UI: 6.43, 12.67) per 100,000, respectively (Supplementary Table 10). At the regional level, East Asia had the highest proportions of ASMR (8.42%) and ASDR (8.87%) due to smoking. At the national level, Indonesia had the highest ASMR (13.29%), whereas Greenland had the highest ASDR (14.82%). From 1990 to 2021, the global proportions of ASMR and ASDR attributable to smoking had decreased, with a similar trend observed across 21 regions and 204 countries (Supplementary Figures 9, 10 and Supplementary Table 11).

### 3.7 Predictive analysis

**FIGURE 6:**
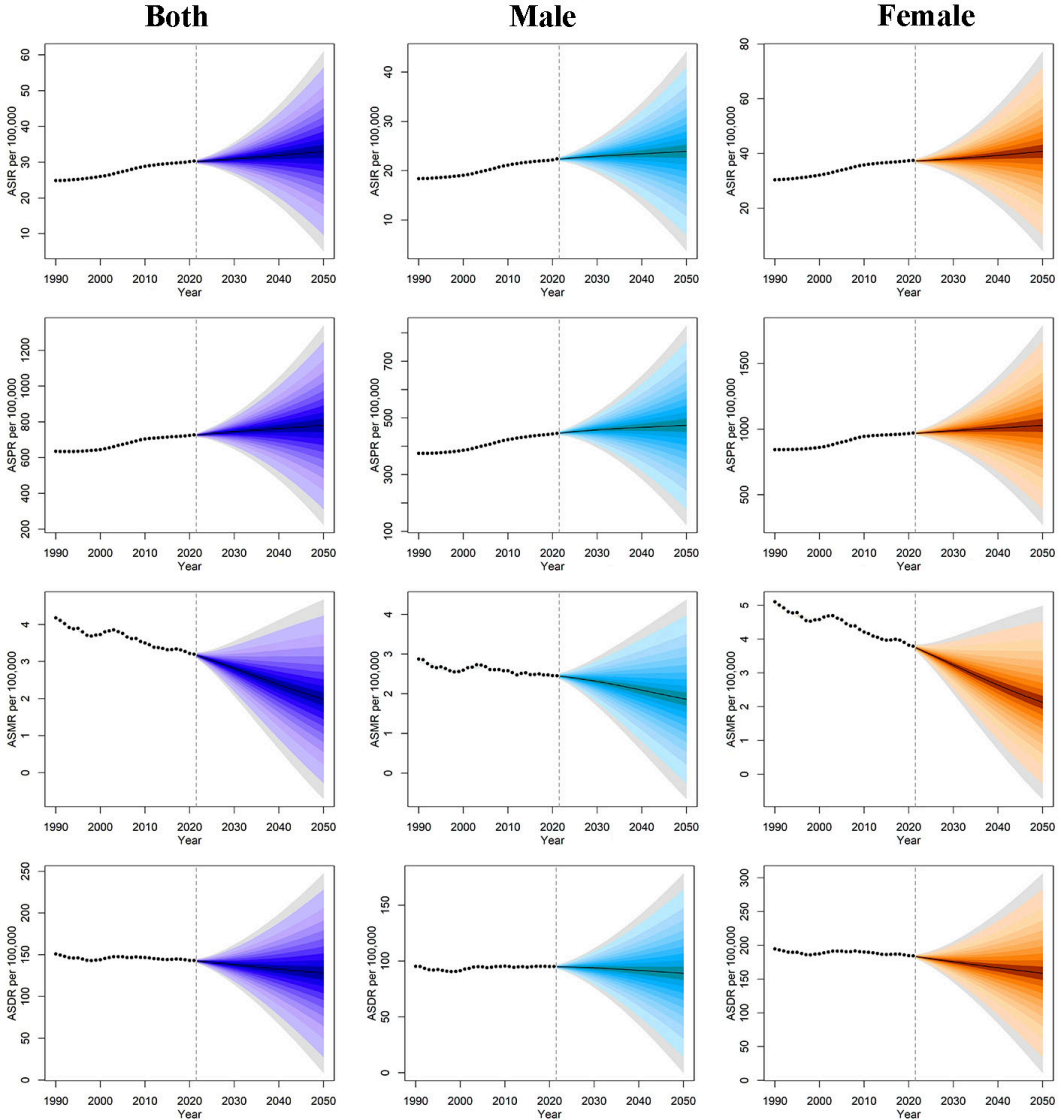
Age-standardiszed rates of EORA from 1990 to 2050 based on the BAPC model, stratified by sex. ASR, age-standardized rate; DALYs, disability-adjusted life years; EORA, elderly-onset rheumatoid arthritis; BAPC, Bayesian age-period cohort.

Between 2021 and 2050, the global burden of EORA is projected to increase. By 2050, the numbers of incident cases, prevalent cases, deaths, and DALYs associated with EORA are expected to reach 710,903 (95% UI: 406,110, 1,015,696), 16,795,385 (95% UI: 10,693,191, 22,897,580), 42,581 (95% UI: 13,118, 72,044), and 2,748,628 (95% UI: 1,442,692, 4,054,564), respectively (Supplementary Tables 12–15). Over the next three decades, ASIR and ASPR are anticipated to increase, whereas ASMR and ASDR are projected to decline. From 2021 to 2050, the burden among females consistently exceeded that among males (Figure 6).

## 4 Discussion

Based on the 2021 GBD database, this study provides the first comprehensive assessment of the incidence, prevalence, mortality, and DALYs of EORA at the global, regional, and national levels from 1990 to 2021. Over the past three decades, the global disease burden of EORA has steadily increased, with the burden for females being generally higher than that for males, and the impact of smoking on EORA has been declining. Additionally, we assessed the influence of SDI on cross-national disease burden disparities and found that the disease burden remains unevenly distributed across regions and countries with different SDI levels.

From 1990 to 2021, both the ASIR and ASPR of global EORA have increased, likely due to factors such as population aging, improvements in diagnostic technologies, better healthcare, and increased patient survival rates (26), which is consistent with previous research findings (27, 28). During the same period, both the ASMR and ASDR for EORA showed a downward trend, suggesting that clinical treatments and early interventions have gradually improved and indicating that preventive and therapeutic strategies were somewhat successful. The projections in this study indicated that the EORA burden will continue to increase in 2025, underscoring the urgent need for effective measures to reduce the EORA disease burden.

The EORA burden shows significant differences across regions, with a lower burden in low-income areas, which may be attributed to poorer accessibility to healthcare services, insufficient diagnostic capabilities, and the lack of data from low- and lower-middle-income regions in the GBD study. Furthermore, the underreporting and limited resources in low-income areas may lead to a degree of underestimation of the actual burden, as the diagnosis of RA is often not timely (29). In regions such as Central Asia, the Andean Latin America, and North Africa and the Middle East, the prevalence of EORA has significantly increased. These regional differences may be associated with lifestyle factors (such as smoking and obesity), environmental exposures (such as pollutants), and genetic factors (30). The disease burden has decreased the most in high-income regions, such as the Asia-Pacific, likely due to well-established healthcare systems that provide broader access to rheumatology services and subsidized treatments (31). Additionally, biologic or targeted synthetic disease-modifying antirheumatic drugs have been widely used in high-income regions (16). In 2021, China and India had the highest number of EORA cases globally, which is closely related to the large populations and increasing age-related risk of RA in these countries (32).

This study quantified and compared the relationship between the EORA disease burden and SDI from multiple perspectives across global and 204 countries and across territories from 1990 to 2021. Correlation analysis results indicated a positive association between the global and regional EORA disease burden (especially prevalence) and SDI levels. This suggests that higher SDI levels are generally associated with a higher EORA disease burden, primarily due to the more pronounced aging trends in populations of higher socio-economic development countries and regions, where the risk of chronic degenerative diseases increases with age. Additionally, age-related physiological changes further exacerbate the decline in physical function and quality of life in patients with RA due to disease activity (33). The cross-national inequality analysis showed that although the EORA disease burden is higher in high-SDI countries, both mortality and DALYs have decreased in terms of the SII and concentration index, suggesting that disparities in mortality and functional loss among countries may gradually diminish with advancements in diagnostic and treatment technologies. The frontier analysis showed that the high-SDI countries (such as Australia, Canada, and the United States) still have a significant gap in ASIR and ASPR compared to the frontier, indicating as considerable potential for reducing new cases and controlling the overall disease course. In contrast, low-SDI countries, such as Somalia, Niger, and Yemen, show smaller gaps, likely due to under-ascertainment from limited healthcare infrastructure and diagnostic capabilities, reflecting global health data inequalities. Low-SDI countries should prioritize strengthening healthcare infrastructure, professional training, and disease surveillance systems to improve EORA burden estimation. Consistent with previous research (34, 35), the prevalence of RA increases with age, with EORA prevalence peaking at 60–69 years. Compared to YORA, EORA is a distinct entity, differing from classic RA, and is characterized by more severe systemic inflammation, more frequent joint function limitations, and cardiovascular complications (36). Additionally, we found that the incidence and prevalence of EORA are higher in females than in males, which may be related to sex hormones and genetic factors. Evidence suggests that genes on the sex chromosomes may increase RA risk in females, whereas estrogen can increase the incidence of autoimmune diseases in females by modulating immune responses (37). The significant decline in estrogen levels during the perimenopausal and postmenopausal periods weakens the protective effects on joint cartilage and decreases bone density, thus increasing the risk of RA in females (38). Notably, this study found a higher prevalence of EORA in females than in males, which contrasts the sex distribution findings of previous studies (39, 40) that reported a relatively balanced sex distribution. This discrepancy may be due to the broader data sources of the 2021 GBD study, where regional, racial, and socio-economic differences, compared to studies based on single countries or regions, are more likely to cause sex distribution variations.

We assessed the impact of smoking on EORA and quantified the proportion of EORA-related ASMR and ASDR attributed to smoking. Although the smoking-related EORA burden remained high in 2021, the proportion of ASMR and ASDR attributable to smoking decreased in most regions globally compared to that observed in 1990. This decline may be attributed to the increasing awareness of the harms of tobacco over the past decades, along with a series of comprehensive interventions, such as increasing tobacco taxes, enacting smoke-free legislation, and banning tobacco advertising, promotion, and sponsorship (41). In addition to smoking, other modifiable environmental and lifestyle factors also play an important role in EORA onset and progression. For example, the acceleration of industrialization and urbanization has led to increased exposure to occupational dust and air pollutants, which in turn has elevated the risk of RA in populations (42). Additionally, unhealthy diets, obesity, and lack of physical activity are closely associated with the rising burden of EORA (43). Therefore, to further reduce the mortality and disease burden caused by EORA, in addition to continuing to strengthen tobacco control measures, multifaceted comprehensive interventions, such as reducing occupational exposure to silica and dust, maintaining healthy body weight, emphasizing oral hygiene, and increasing omega-3 fatty acid and fish intake, are needed (44). However, these additional risk factors were not included in our quantitative analysis due to the absence of attributable risk data in the 2021 GBD study. Given their emerging roles in RA etiology, future research should prioritize incorporating these factors to better understand the evolving epidemiology of EORA.

Similar to other GBD-related studies, our research also has some limitations. First, due to limited overall input data for GBD and missing data in some regions, the reflection of the true disease burden may not be sufficiently precise (45, 46). Second, this study defines patients aged 60 years and older as patients with EORA. However, the age criterion for EORA is not yet unified across different studies or clinical practices, and there is some uncertainty about whether some older patients truly belong to EORA. Additionally, the GBD data does not distinguish between age at onset and current age, potentially including younger-onset RA cases that persist into elderly age groups in our EORA estimates, which may lead to an overestimation of the true EORA burden. Third, although this study uses multiple indicators such as SDI, SII, and concentration index to measure the unequal distribution of EORA burden, potential factors such as access to medical services was not included in the cross-country comparisons, limiting a more comprehensive understanding of EORA burden inequalities. Fourth, the 2021 GBD study identifies smoking as the only attributable risk factor for RA, limiting our analysis to this single factor and potentially underestimating the evolving contributions of other modifiable risk factors, such as obesity, diet, and environmental pollution. We recommend that future GBD iterations incorporate these factors to enable more comprehensive risk modeling of EORA burden.

## 5 Conclusion

From 1990 to 2021, the global EORA burden significantly increased and is projected to continue rising through 2050, with females generally bearing a higher burden than males. The distribution of EORA burden remains uneven across SDI regions, with considerable variation in incidence and prevalence. Although the burden of EORA attributable to smoking has declined, smoking remains a significant modifiable risk factor. Beyond reinforcing tobacco control efforts, there is a need to strengthen research and interventions targeting environmental and lifestyle factors, reduce socio-economic inequalities, and improve monitoring and reporting systems. Future strategies should prioritize targeted prevention strategies and treatment for older adults and high-risk populations to effectively reduce the global EORA burden.

## Data Availability

The original contributions presented in the study are included in the article/Supplementary material, further inquiries can be directed to the corresponding author/s.
