# Supplementary material for: Unveiling health inequalities and frontier gaps in elderly-onset rheumatoid arthritis: evolving impact of smoking and future challenges
Source: Front Med (Lausanne). 2025 Oct 9;12:1664232. doi: 10.3389/fmed.2025.1664232 (PMC12546154; doi:10.3389/fmed.2025.1664232)
Supplement: Supplementary file 1 [file Data_Sheet_1.docx]

**Additional file 1:**

**Unveiling health inequalities and frontier gaps in elderly-onset rheumatoid arthritis: evolving impact of smoking and future challenges**

Contents

[Table S1. Numbers of cases for incidence, prevalence, mortality, and DALYs of elderly-onset rheumatoid arthritis at the global and regional levels in 1990 and 2021. 1](#_Toc194361942)

[Table S2. Incidence cases and age-standardised rates of elderly-onset rheumatoid arthritis across 204 countries and territories in 1990 and 2021, and their estimated annual percentage changes from 1990 to 2021. 4](#_Toc194361943)

[Table S3. Prevalence cases and age-standardised rates of elderly-onset rheumatoid arthritis across 204 countries and territories in 1990 and 2021, and their estimated annual percentage changes from 1990 to 2021. 13](#_Toc194361944)

[Table S4. Mortality cases and age-standardised rates of elderly-onset rheumatoid arthritis across 204 countries and territories in 1990 and 2021, and their estimated annual percentage changes from 1990 to 2021. 22](#_Toc194361945)

[Table S5. DALYs and age-standardised rates of elderly-onset rheumatoid arthritis across 204 countries and territories in 1990 and 2021, and their estimated annual percentage changes from 1990 to 2021. 31](#_Toc194361946)

[Table S6. Age-standardised incidence rates of elderly-onset rheumatoid arthritis with frontier analysis across 204 countries and territories in 1990 and 2021. 40](#_Toc194361947)

[Table S7. Age-standardised prevalence rates of elderly-onset rheumatoid arthritis with frontier analysis across 204 countries and territories in 1990 and 2021. 50](#_Toc194361949)

[Table S8. Age-standardised mortality rates of elderly-onset rheumatoid arthritis with frontier analysis across 204 countries and territories in 1990 and 2021. 61](#_Toc194361950)

[Table S9. Age-standardised DALYs rates of elderly-onset rheumatoid arthritis with frontier analysis across 204 countries and territories in 1990 and 2021. 71](#_Toc194361951)

[Table S10. Age-standardised mortality and DALYs rates attributable to smoking for elderly-onset rheumatoid arthritis globally and regionally in 1990 and 2021, along with their proportions. 80](#_Toc194361952)

[Table S11. Age-standardised mortality rates and DALYs rates attributable to smoking for elderly-onset rheumatoid arthritis across 204 countries and territories in 1990 and 2021, along with their proportions. 82](#_Toc194361953)

[Table S12. Projected number of incident cases and age-standardised incidence rates of elderly-onset rheumatoid arthritis worldwide from 2022 to 2050 based on the BAPC model, by sex. 92](#_Toc194361954)

[Table S13. Projected number of prevalent cases and age-standardised prevalence rates of elderly-onset rheumatoid arthritis worldwide from 2022 to 2050 based on the BAPC model, by sex. 94](#_Toc194361955)

[Table S14. Projected number of deaths and age-standardised mortality rates of elderly-onset rheumatoid arthritis worldwide from 2022 to 2050 based on the BAPC model, by sex. 95](#_Toc194361956)

[Table S15. Projected number of DALYs and age-standardised DALY rates of elderly-onset rheumatoid arthritis worldwide from 2022 to 2050 based on the BAPC model, by sex. 97](#_Toc194361957)

[Figure. S1. Estimated annual percentage change in age-standardised (A), prevalence (B), mortality (C), and DALYs (D) rates of elderly-onset rheumatoid arthritis globally and regionally, 1990-2021. 99](#_Toc194361958)

[Figure. S2. Age-standardised incidence (A), prevalence (B), mortality (C), and DALYs (D) rates of elderly-onset rheumatoid arthritis in 204 countries and territories in 2021. 100](#_Toc194361959)

[Figure. S3. Age-standardised incidence rates of elderly-onset rheumatoid arthritis by sex, age group, and socio-demographic index, 1990 and 2021. 101](#_Toc194361960)

[Figure. S4. Age-standardised mortality rates of elderly-onset rheumatoid arthritis by sex, age group, and socio-demographic index, 1990 and 2021. 102](#_Toc194361961)

[Figure. S5. Age-standardised DALYs rates of elderly-onset rheumatoid arthritis by sex, age group, and socio-demographic index, 1990 and 2021. 103](#_Toc194361962)

[Figure. S6. Estimated annual percentage change in the age-standardized incidence (A), prevalence (B), mortality (C), and DALYs (D) rates of elderly-onset rheumatoid arthritis by sex and age group, globally and regionally. 104](#_Toc194361963)

[Figure. S7. Age-standardised incidence (A), prevalence (B), mortality (C), and DALYs (D) rates of elderly-onset rheumatoid arthritis, globally and for 21 GBD regions, by socio-demographic index, from 1990 to 2021. 105](#_Toc194361964)

[Figure. S8. Age-standardised incidence (A), prevalence (B), mortality (C), and DALYs (D) rates of elderly-onset rheumatoid arthritis in 204 countries and territories in 2021, by socio-demographic index in 2021. 106](#_Toc194361965)

[Figure. S9. Proportions and trends of age-standardised mortality and DALYs rates for elderly-onset rheumatoid arthritis attributable to smoking in 1990 and 2021, globally and regionally. 107](#_Toc194361966)

[Figure. S10. Proportions and trends of age-standardised mortality (A, B) and DALYs (C, D) rates for elderly-onset rheumatoid arthritis attributable to smoking in 1990 and 2021, at the national level. 108](#_Toc194361967)

**Table** **S1. Numbers of cases for incidence, prevalence, mortality, and DALYs of elderly-onset rheumatoid arthritis at the global and regional levels in 1990 and 2021.**

| **Location** | **Incidences (95 % UI)** | | **Prevalence (95 % UI)** | | **Mortality (95 % UI)** | | **DALYs (95 % UI)** | |
| --- | --- | --- | --- | --- | --- | --- | --- | --- |
|  | **Number, 1990** | **Number, 2021** | **Number, 1990** | **Number, 2021** | **Number, 1990** | **Number, 2021** | **Number, 1990** | **Number, 2021** |
| Global | 123945.06(78947.83,176323.84) | 334290.74(218244.71,467590.29) | 3074297.77(2663033.90,3566184.75) | 7919135.55(6902538.91,9101562.52) | 17914.34(15573.41,20290.04) | 33200.28(26862.02,38572.55) | 715789.03(584552.61,867373.57) | 1549877.30(1225839.76,1934956.79) |
| Male | 41166.49(26190.90,58460.69) | 114773.71(75357.70,159320.61) | 807573.69(684190.79,957418.18) | 2231565.88(1918139.28,2607880.25) | 5031.66(3671.24,5920.42) | 10859.62(6951.17,13213.83) | 197325.99(156209.16,245098.47) | 466659.32(357132.46,586104.38) |
| Female | 82778.56(52842.54,117674.08) | 219517.03(142939.80,307929.26) | 2266724.09(1976006.90,2606175.64) | 5687569.67(4983488.81,6499628.06) | 12882.68(11194.94,14764.61) | 22340.67(18108.67,26865.87) | 518463.04(424547.72,628625.03) | 1083217.98(844462.46,1357610.23) |
| **SDI** **regions** | | | | | | | | |
| Low SDI | 4034.68(2548.88,5809.76) | 12552.94(8057.84,17650.15) | 74010.38(62732.20,87385.54) | 230439.23(197348.86,270529.64) | 534.41(335.62,928.13) | 1250.74(819.02,2077.55) | 19594.31(14534.56,27201.44) | 51705.91(39204.67,68675.44) |
| Low-middle SDI | 16638.91(10536.39,23753.38) | 57585.59(37216.47,80821.32) | 282767.67(237866.03,337586.73) | 1043788.42(890691.88,1230019.92) | 2813.82(1899.86,3992.55) | 7161.16(5112.73,10089.21) | 88538.91(66799.48,112034.45) | 256367.63(199154.99,324850.03) |
| Middle SDI | 24197.59(14391.97,36037.31) | 91430.51(57280.85,131870.61) | 619211.67(526768.20,727526.61) | 2162625.21(1861774.44,2520907.30) | 3901.48(3137.07,4606.77) | 10041.52(7626.84,11719.97) | 150840.85(122149.76,186802.65) | 443837.65(349379.12,553722.92) |
| High-middle SDI | 20494.66(12553.00,30386.75) | 57578.71(36776.02,82142.13) | 652000.42(565610.95,751743.38) | 1655819.33(1448631.19,1890716.43) | 3009.28(2661.10,3407.85) | 6175.88(4993.39,7251.06) | 141283.72(114422.67,174788.98) | 313437.89(244170.67,397013.35) |
| High SDI | 58507.76(38954.57,81004.51) | 114994.17(78215.01,157378.39) | 1443686.22(1264277.59,1658853.17) | 2821305.14(2499105.31,3199757.44) | 7638.36(6965.82,8037.57) | 8552.28(7193.69,9349.33) | 314888.64(256815.39,383366.67) | 483578.60(370878.69,615017.05) |
| **GBD regions** | | | | | | | | |
| East Asia | 24268.58(13996.59,37179.16) | 83426.76(51851.49,121574.68) | 666303.27(564153.62,789309.44) | 2077439.86(1781244.38,2427412.74) | 3529.58(2817.90,4451.28) | 9219.82(6404.73,11433.17) | 153700.45(122411.82,194783.06) | 417049.67(320437.39,530081.30) |
| South Asia | 45266.68(28695.35,64904.69) | 181019.54(116163.82,255204.54) | 680550.23(563440.20,825970.22) | 2894699.89(2431560.03,3458498.73) | 6898.77(4579.79,9997.42) | 18059.14(12857.41,26628.04) | 215238.70(160608.60,275020.64) | 678046.73(526135.49,871511.82) |
| Southeast Asia | 1758.20(1033.36,2615.31) | 7058.91(4258.14,10307.37) | 46551.74(39265.92,55150.73) | 183758.51(157246.39,214391.09) | 342.80(205.92,454.97) | 901.79(554.60,1139.17) | 12117.27(8936.52,15570.28) | 38755.11(28883.31,49683.06) |
| Central Asia | 357.34(214.70,537.93) | 834.59(486.93,1255.62) | 16793.10(14888.34,18964.04) | 41124.99(36892.05,45830.35) | 9.73(7.51,14.66) | 91.71(77.86,106.99) | 2316.71(1597.27,3207.86) | 6871.74(5151.37,9057.98) |
| High-income Asia Pacific | 11621.22(7126.26,17222.08) | 19794.82(12503.98,28367.68) | 306750.58(258776.81,364702.86) | 581259.47(503030.94,673504.13) | 1516.88(1358.15,1638.02) | 2348.46(1858.38,2664.60) | 66553.42(53469.69,82416.06) | 107105.89(82447.81,136003.93) |
| Oceania | 8.70(4.68,13.63) | 24.44(13.71,37.42) | 317.59(265.51,380.21) | 925.91(789.27,1090.56) | 0.02(0.01,0.07) | 0.02(0.01,0.07) | 43.36(27.54,63.63) | 122.50(75.56,183.69) |
| Australasia | 2065.19(1392.08,2820.49) | 5044.05(3437.44,6936.90) | 43339.24(38003.88,49189.06) | 104425.21(91973.62,118258.16) | 208.76(178.92,240.06) | 276.24(221.39,331.66) | 9108.08(7244.00,11330.07) | 17306.41(12969.79,22379.55) |
| Eastern Europe | 1175.86(583.34,1960.40) | 1827.92(928.80,2995.15) | 123479.24(109173.64,139652.17) | 191303.07(170923.30,213927.85) | 632.19(588.05,664.23) | 1036.40(931.35,1140.13) | 29867.51(24771.05,36021.75) | 45201.32(37704.43,54538.66) |
| Western Europe | 31259.26(21323.51,42735.79) | 53188.41(36854.93,71806.54) | 730253.45(638961.78,838567.34) | 1224006.96(1077948.01,1387092.32) | 4159.07(3777.78,4434.41) | 3684.78(3090.91,4067.76) | 160998.68(131295.88,195725.38) | 207618.18(158773.60,265432.70) |
| Central Europe | 1538.02(841.70,2392.82) | 2942.73(1656.18,4527.87) | 95700.28(84567.27,108337.46) | 173188.82(153779.26,194534.37) | 598.32(562.67,631.78) | 453.81(403.91,498.99) | 24135.24(20378.71,28690.08) | 30111.32(23285.73,38390.65) |
| High-income North America | 18345.99(12159.00,25297.91) | 42454.74(28757.19,57699.21) | 436947.09(386272.83,495876.53) | 970351.64(863514.15,1091818.79) | 1730.37(1562.35,1838.57) | 2354.14(2005.93,2610.73) | 85705.39(68318.50,105910.33) | 158042.33(120603.70,200669.34) |
| Andean Latin America | 514.77(362.54,692.71) | 2329.41(1553.75,3241.21) | 14278.49(12735.40,15957.28) | 76234.93(68312.64,84815.86) | 134.51(100.60,169.28) | 287.19(217.91,372.01) | 3734.71(2904.77,4671.41) | 13923.18(10509.42,17821.21) |
| Central Latin America | 3272.01(2051.87,4731.85) | 10434.99(6653.09,14961.91) | 85105.11(73693.16,98363.82) | 324017.86(286604.17,367374.86) | 826.58(770.20,874.36) | 1819.89(1569.29,2066.04) | 24164.34(20606.24,28313.79) | 72224.39(58847.14,88177.00) |
| Caribbean | 489.07(355.63,646.25) | 1264.54(917.77,1678.84) | 10580.11(9299.91,12039.64) | 31258.47(27723.92,35208.60) | 98.67(82.66,116.64) | 211.90(171.00,258.77) | 2814.24(2263.25,3429.87) | 6950.08(5534.77,8610.25) |
| Tropical Latin America | 591.14(309.90,958.87) | 1832.33(994.34,2916.73) | 34353.09(29523.89,39808.71) | 103052.44(89810.26,118478.93) | 157.66(142.42,171.64) | 484.15(420.26,537.99) | 7375.57(5899.58,9160.32) | 22106.23(17660.78,27516.00) |
| Southern Latin America | 1135.76(756.46,1577.85) | 2951.85(1902.60,4227.33) | 28269.19(24840.67,32259.68) | 90017.35(80582.62,100579.91) | 175.70(152.77,198.75) | 328.33(275.87,382.02) | 6836.36(5569.16,8346.05) | 16830.04(13100.49,21178.27) |
| Eastern Sub-Saharan Africa | 919.44(568.58,1336.92) | 2317.87(1446.42,3327.71) | 21223.69(18190.09,24887.83) | 53393.88(46220.32,61622.55) | 19.11(6.43,146.56) | 25.33(8.21,247.37) | 3061.72(2068.52,5485.22) | 7245.43(4949.84,11492.00) |
| Southern Sub-Saharan Africa | 525.34(309.86,797.84) | 1208.65(716.55,1822.75) | 19997.79(17506.78,22893.25) | 42343.68(37146.40,48561.10) | 140.65(96.10,180.88) | 249.60(198.96,318.16) | 5051.49(3934.25,6311.82) | 9929.97(7903.70,12364.60) |
| Western Sub-Saharan Africa | 553.22(330.07,820.38) | 1412.18(861.60,2052.43) | 14084.42(11700.98,16827.17) | 36960.46(31220.53,43605.91) | 5.61(2.52,9.63) | 11.43(5.20,22.46) | 1905.93(1278.56,2717.41) | 4917.47(3336.28,6968.68) |
| North Africa and Middle East | 1317.20(702.44,2083.90) | 5469.63(2993.71,8522.40) | 65656.63(57292.14,75586.38) | 293549.43(260006.31,331557.16) | 340.57(214.44,505.52) | 744.62(519.77,1000.91) | 15267.04(11434.17,20151.62) | 51499.47(38476.75,67527.77) |
| Central Sub-Saharan Africa | 254.00(155.97,368.62) | 696.96(422.74,1012.62) | 6866.89(6006.74,7840.04) | 19947.39(17495.45,22789.68) | 8.45(2.45,74.80) | 13.42(3.39,143.70) | 1045.70(667.98,2468.67) | 2792.93(1802.06,5397.60) |

**Abbreviations: UI: uncertainly interval; DALY=disability-adjusted life-years; GBD, global burden of disease;** **SDI: sociodemographic index.**

**Table S2. Incidence cases and age-standardised rates of elderly-onset rheumatoid arthritis across 204 countries and territories in 1990 and 2021, and their estimated annual percentage changes from 1990 to 2021.**

| **Location** | **Number (95 % UI), 1990** | **ASRs (95 % UI),1990** | **Number (95 % UI), 2021** | **ASRs (95 % UI),2021** | **EAPCs (95% CI)** |
| --- | --- | --- | --- | --- | --- |
| Afghanistan | 18.62(9.83,29.34) | 2.21(0.55,4.98) | 21.37(11.75,33.11) | 2.66(0.75,5.79) | 0.69(0.54,0.85) |
| Albania | 16.14(8.89,24.99) | 6.66(1.67,15.22) | 71.12(39.31,108.66) | 11.87(4.67,21.88) | 2.06(1.98,2.14) |
| Algeria | 41.64(22.21,65.67) | 2.81(0.94,5.64) | 178.66(98.44,281.46) | 4.20(1.88,7.44) | 1.25(1.18,1.32) |
| American Samoa | 0.09(0.05,0.14) | 3.50(0.00,160.18) | 0.24(0.13,0.38) | 4.30(0.00,78.48) | 0.54(0.47,0.61) |
| Andorra | 0.97(0.61,1.39) | 13.04(0.03,84.25) | 2.61(1.68,3.73) | 13.49(0.78,50.97) | 0.21(0.14,0.28) |
| Angola | 40.35(24.43,59.43) | 9.91(3.86,18.81) | 155.21(93.46,231.20) | 12.53(6.09,21.23) | 0.80(0.69,0.91) |
| Antigua and Barbuda | 1.58(1.26,1.97) | 22.93(0.89,104.13) | 3.41(2.60,4.37) | 25.47(3.39,80.46) | 0.18(0.09,0.28) |
| Argentina | 643.03(424.13,895.84) | 15.44(9.24,22.97) | 1160.09(741.70,1653.25) | 16.14(9.59,24.14) | 0.19(0.08,0.30) |
| Armenia | 14.30(8.03,22.36) | 4.26(1.04,10.05) | 34.34(19.14,53.19) | 5.69(1.92,11.53) | 0.90(0.79,1.00) |
| Australia | 1669.03(1119.28,2293.24) | 64.84(40.97,92.81) | 4232.17(2845.49,5852.33) | 72.24(46.80,102.49) | 0.45(0.35,0.56) |
| Austria | 500.57(327.19,704.97) | 32.14(18.79,48.74) | 702.92(454.73,969.69) | 30.83(18.15,45.29) | 0.02(-0.11,0.14) |
| Azerbaijan | 20.08(11.53,30.95) | 3.49(1.02,7.64) | 53.86(31.08,82.26) | 4.29(1.68,8.14) | 0.92(0.60,1.24) |
| Bahamas | 3.11(2.33,4.02) | 17.73(2.00,58.53) | 9.49(6.78,12.80) | 19.25(5.43,44.58) | 0.31(0.28,0.34) |
| Bahrain | 0.99(0.52,1.60) | 5.56(0.00,37.03) | 9.93(5.24,16.44) | 10.02(1.78,26.76) | 1.87(1.80,1.93) |
| Bangladesh | 550.40(363.79,758.32) | 10.32(6.14,15.27) | 2696.44(1797.33,3761.62) | 15.95(10.14,22.97) | 1.61(1.41,1.81) |
| Barbados | 13.10(10.31,16.55) | 34.26(13.10,69.72) | 25.30(18.31,33.47) | 36.35(15.67,67.37) | 0.06(-0.03,0.16) |
| Belarus | 62.43(31.91,100.13) | 3.67(1.28,7.15) | 121.23(62.73,197.88) | 5.51(2.19,10.34) | 1.19(0.98,1.40) |
| Belgium | 728.30(488.91,1008.04) | 35.76(21.92,52.64) | 1098.37(724.33,1513.35) | 36.88(22.58,53.44) | 0.14(0.08,0.21) |
| Belize | 1.78(1.34,2.32) | 15.60(0.65,67.76) | 5.69(4.13,7.54) | 16.50(3.36,43.94) | 0.30(0.21,0.38) |
| Benin | 14.12(8.60,20.55) | 6.02(1.64,13.46) | 40.60(24.53,58.82) | 7.16(2.79,13.39) | 0.52(0.47,0.57) |
| Bermuda | 1.77(1.31,2.32) | 22.66(0.90,99.01) | 5.92(4.53,7.67) | 32.70(7.55,84.63) | 1.21(1.08,1.34) |
| Bhutan | 6.58(4.26,9.38) | 24.95(4.65,66.70) | 30.12(19.30,43.00) | 41.57(16.11,79.96) | 1.67(1.65,1.70) |
| Bolivia (Plurinational State of) | 91.33(62.14,127.86) | 24.55(12.81,40.87) | 401.25(255.42,569.32) | 36.05(20.22,55.54) | 1.21(1.13,1.28) |
| Bosnia and Herzegovina | 53.81(29.61,86.49) | 11.07(4.10,21.96) | 121.95(68.32,191.70) | 14.40(6.27,26.07) | 1.00(0.91,1.09) |
| Botswana | 7.94(4.88,11.65) | 12.15(2.38,31.38) | 24.96(15.06,36.66) | 15.06(5.09,30.53) | 0.79(0.71,0.87) |
| Brazil | 573.85(300.38,931.09) | 5.46(2.54,9.45) | 1766.52(957.83,2816.58) | 5.59(2.84,9.25) | 0.01(-0.05,0.08) |
| Brunei Darussalam | 2.84(1.86,3.94) | 26.49(1.86,94.77) | 14.36(9.60,19.93) | 35.84(11.28,76.90) | 0.98(0.95,1.01) |
| Bulgaria | 79.11(43.73,123.04) | 4.70(1.88,8.72) | 113.24(64.53,176.87) | 5.87(2.58,10.63) | 0.82(0.69,0.95) |
| Burkina Faso | 32.05(19.26,46.77) | 6.28(2.28,12.20) | 72.18(44.26,104.19) | 6.92(3.09,12.10) | 0.36(0.31,0.40) |
| Burundi | 26.76(16.81,37.93) | 10.03(3.66,19.52) | 56.94(35.81,81.87) | 10.58(4.66,18.88) | 0.21(0.14,0.28) |
| Cabo Verde | 1.45(0.87,2.17) | 4.94(0.05,25.48) | 4.18(2.52,6.13) | 7.80(0.79,24.71) | 1.21(1.09,1.33) |
| Cambodia | 26.36(16.33,38.49) | 5.20(1.85,10.41) | 143.11(88.26,206.01) | 9.76(4.83,16.10) | 1.95(1.88,2.02) |
| Cameroon | 35.04(20.89,51.96) | 6.95(2.56,13.51) | 112.00(69.05,161.67) | 8.16(3.91,13.74) | 0.45(0.35,0.55) |
| Canada | 1660.11(1267.35,2069.01) | 39.21(28.31,51.03) | 5022.79(3760.37,6242.68) | 52.25(37.88,66.58) | 0.95(0.88,1.03) |
| Central African Republic | 11.50(6.85,17.13) | 9.36(2.22,22.29) | 21.13(12.61,31.06) | 9.49(2.98,19.80) | 0.09(-0.01,0.19) |
| Chad | 18.20(11.00,26.65) | 5.45(1.65,11.65) | 37.57(23.20,54.67) | 5.96(2.34,11.29) | 0.29(0.20,0.38) |
| Chile | 353.60(221.51,521.88) | 28.86(15.78,46.41) | 1564.85(958.04,2326.59) | 47.12(27.05,72.97) | 1.58(1.53,1.63) |
| China | 23456.43(13447.85,36009.54) | 23.42(13.20,36.33) | 80905.29(49797.41,118512.81) | 30.07(18.35,44.31) | 0.82(0.81,0.84) |
| Colombia | 270.42(171.03,393.39) | 13.33(7.22,21.41) | 1172.77(735.49,1704.12) | 16.81(9.79,25.61) | 0.83(0.76,0.90) |
| Comoros | 2.64(1.67,3.79) | 11.71(0.66,44.12) | 8.02(4.93,11.53) | 14.37(2.84,36.49) | 0.76(0.67,0.86) |
| Congo | 13.95(8.20,20.70) | 11.37(2.92,25.88) | 38.95(23.33,58.11) | 13.87(5.28,26.73) | 0.74(0.63,0.86) |
| Cook Islands | 0.04(0.02,0.06) | 2.77(0.00,260.52) | 0.12(0.06,0.19) | 3.49(0.00,119.89) | 0.78(0.68,0.89) |
| Costa Rica | 45.72(28.83,67.00) | 21.90(9.24,40.76) | 207.34(126.21,306.78) | 29.60(15.01,48.98) | 0.92(0.87,0.98) |
| Côte d'Ivoire | 28.47(17.04,42.02) | 6.84(2.39,13.65) | 97.43(59.25,141.20) | 8.23(3.81,14.06) | 0.56(0.47,0.64) |
| Croatia | 96.64(51.61,155.84) | 12.23(4.87,23.08) | 204.46(107.80,322.05) | 17.14(7.41,30.11) | 0.95(0.84,1.05) |
| Cuba | 178.61(116.04,254.94) | 13.93(7.48,22.48) | 417.65(265.48,601.53) | 17.18(9.65,26.81) | 0.61(0.57,0.65) |
| Cyprus | 70.91(45.32,100.57) | 66.14(30.87,114.02) | 219.41(137.48,310.35) | 80.46(42.34,127.21) | 1.03(0.85,1.21) |
| Czechia | 99.36(53.83,156.31) | 5.41(2.20,9.95) | 205.10(112.29,322.45) | 7.29(3.29,12.79) | 1.18(1.04,1.31) |
| Democratic People's Republic of Korea | 326.03(198.19,479.16) | 17.97(9.46,28.89) | 966.93(616.61,1402.11) | 24.29(14.29,37.11) | 0.97(0.92,1.02) |
| Democratic Republic of the Congo | 176.84(108.39,259.32) | 10.06(5.06,16.67) | 452.20(276.36,667.23) | 11.55(6.25,18.39) | 0.43(0.32,0.55) |
| Denmark | 657.93(463.92,871.05) | 62.61(40.22,88.59) | 1300.33(900.54,1743.22) | 85.19(55.20,119.69) | 1.22(1.14,1.29) |
| Djibouti | 1.52(0.93,2.22) | 11.19(0.13,55.73) | 8.94(5.41,13.06) | 13.38(2.78,33.39) | 0.62(0.49,0.74) |
| Dominica | 1.67(1.33,2.06) | 21.59(0.95,94.91) | 2.36(1.87,2.98) | 21.86(1.87,80.96) | -0.12(-0.27,0.03) |
| Dominican Republic | 80.93(66.22,97.49) | 18.67(11.82,27.42) | 276.06(225.12,336.56) | 22.78(16.23,30.91) | 0.52(0.29,0.76) |
| Ecuador | 216.46(161.18,279.11) | 35.14(22.28,50.96) | 1005.35(701.20,1354.45) | 49.69(32.14,70.61) | 1.03(0.94,1.13) |
| Egypt | 84.52(44.52,134.72) | 2.81(1.08,5.30) | 321.74(173.65,504.93) | 4.38(2.02,7.49) | 1.35(1.24,1.45) |
| El Salvador | 33.75(23.91,45.35) | 9.52(4.32,17.10) | 106.42(74.01,144.53) | 13.87(7.57,22.16) | 1.29(1.18,1.40) |
| Equatorial Guinea | 2.18(1.33,3.17) | 10.20(0.34,42.13) | 9.10(5.33,13.89) | 17.33(3.45,44.44) | 1.88(1.83,1.94) |
| Eritrea | 10.52(6.61,15.18) | 9.95(2.43,23.61) | 35.56(21.87,51.57) | 12.57(4.84,23.93) | 0.73(0.66,0.80) |
| Estonia | 15.19(7.40,25.58) | 5.68(1.15,14.06) | 23.03(11.24,39.82) | 6.51(1.60,15.33) | 0.57(0.39,0.75) |
| Eswatini | 4.16(2.57,6.17) | 13.42(1.42,42.84) | 9.40(5.68,14.00) | 14.99(3.21,37.46) | 0.41(0.23,0.60) |
| Ethiopia | 237.81(136.82,361.11) | 10.81(5.22,18.20) | 540.15(327.70,787.93) | 11.26(6.11,17.62) | 0.24(0.14,0.35) |
| Fiji | 0.86(0.45,1.39) | 2.33(0.00,16.87) | 2.38(1.27,3.76) | 2.59(0.07,10.75) | 0.42(0.21,0.64) |
| Finland | 591.87(427.34,779.40) | 63.31(41.48,89.44) | 1214.92(849.40,1614.83) | 73.81(48.19,103.01) | 0.70(0.63,0.77) |
| France | 3780.53(2532.47,5167.08) | 34.86(22.45,48.97) | 7386.92(4862.35,10079.18) | 41.49(26.55,57.73) | 0.64(0.52,0.76) |
| Gabon | 9.18(5.51,13.67) | 13.18(2.75,33.11) | 20.37(12.03,30.53) | 16.99(5.19,36.26) | 0.78(0.69,0.88) |
| Gambia | 2.45(1.48,3.61) | 6.34(0.27,25.01) | 8.26(5.01,12.04) | 7.62(1.50,19.37) | 0.50(0.46,0.54) |
| Georgia | 36.20(20.61,56.29) | 4.45(1.56,8.99) | 40.28(22.97,61.77) | 4.95(1.79,9.74) | 0.28(0.16,0.41) |
| Germany | 4204.37(2789.37,5902.71) | 25.81(16.50,37.17) | 7020.17(4795.35,9804.60) | 28.55(18.95,40.67) | 0.24(0.06,0.42) |
| Ghana | 39.70(22.67,60.58) | 5.73(2.07,11.25) | 143.84(84.48,214.24) | 7.66(3.59,13.05) | 0.79(0.68,0.89) |
| Greece | 324.89(209.60,465.25) | 16.23(9.10,25.46) | 527.84(350.86,735.50) | 17.72(10.58,26.54) | 0.21(0.17,0.26) |
| Greenland | 0.62(0.40,0.88) | 17.27(0.00,149.89) | 2.66(1.71,3.76) | 29.99(1.78,111.39) | 1.97(1.91,2.02) |
| Grenada | 0.90(0.64,1.21) | 9.50(0.03,62.76) | 1.73(1.20,2.38) | 12.49(0.37,56.30) | 0.40(0.21,0.59) |
| Guam | 0.30(0.16,0.48) | 3.40(0.00,52.76) | 1.11(0.61,1.76) | 4.01(0.01,24.70) | 0.46(0.34,0.59) |
| Guatemala | 55.03(41.04,71.91) | 13.87(7.42,22.83) | 322.14(238.42,425.12) | 24.13(15.67,35.03) | 1.74(1.58,1.91) |
| Guinea | 24.14(14.64,35.01) | 5.93(2.00,11.96) | 42.24(25.79,61.03) | 6.56(2.61,12.17) | 0.19(0.09,0.29) |
| Guinea-Bissau | 2.74(1.68,3.93) | 6.15(0.34,22.73) | 5.57(3.48,7.96) | 7.34(1.10,20.70) | 0.53(0.48,0.58) |
| Guyana | 3.02(2.10,4.10) | 6.86(0.62,23.64) | 7.02(5.01,9.48) | 8.98(2.08,22.66) | 0.73(0.63,0.83) |
| Haiti | 35.96(26.38,47.60) | 9.63(4.63,16.91) | 88.82(64.38,120.32) | 11.06(6.18,17.90) | 0.53(0.47,0.60) |
| Honduras | 59.45(40.73,81.82) | 25.27(12.41,43.19) | 249.46(164.62,357.21) | 33.17(18.67,52.68) | 0.82(0.77,0.86) |
| Hungary | 189.13(98.26,302.73) | 9.62(4.06,17.23) | 293.92(155.49,452.53) | 11.24(5.05,18.98) | 0.75(0.62,0.88) |
| Iceland | 11.06(7.15,15.59) | 29.94(7.87,69.01) | 26.82(17.26,37.53) | 35.36(13.31,68.04) | 0.53(0.44,0.62) |
| India | 20693.09(13098.46,29724.84) | 40.09(24.94,58.25) | 84458.60(54028.93,119227.88) | 58.20(36.92,82.63) | 1.27(1.23,1.31) |
| Indonesia | 424.90(235.49,650.12) | 3.90(1.89,6.44) | 1587.89(938.76,2362.49) | 5.63(3.12,8.72) | 0.96(0.84,1.07) |
| Iran (Islamic Republic of) | 95.66(48.44,156.07) | 3.09(1.15,5.89) | 370.52(197.01,592.30) | 4.02(1.85,6.97) | 0.81(0.66,0.95) |
| Iraq | 26.84(14.16,42.91) | 2.97(0.86,6.40) | 126.44(69.56,199.46) | 4.80(2.06,8.70) | 1.65(1.54,1.76) |
| Ireland | 409.06(262.41,560.94) | 76.09(43.08,113.34) | 1065.70(690.88,1477.68) | 104.89(63.02,153.05) | 1.30(1.21,1.38) |
| Israel | 134.62(92.22,185.15) | 21.15(11.68,33.60) | 457.94(312.37,621.40) | 28.87(17.57,42.37) | 1.05(1.00,1.10) |
| Italy | 4473.72(2663.31,6653.41) | 37.86(21.69,57.68) | 7197.97(4473.06,10412.59) | 39.93(24.09,58.88) | 0.18(0.15,0.21) |
| Jamaica | 32.27(24.03,42.13) | 14.04(6.70,24.78) | 59.70(44.03,79.16) | 15.26(8.18,25.21) | 0.24(0.13,0.36) |
| Japan | 10913.96(6612.78,16283.37) | 50.03(29.59,75.80) | 15576.70(9461.80,22929.37) | 35.16(20.93,52.43) | -1.03(-1.09,-0.97) |
| Jordan | 4.37(2.33,6.98) | 3.12(0.25,10.27) | 37.61(20.47,59.01) | 4.63(1.55,9.37) | 1.41(1.30,1.52) |
| Kazakhstan | 62.99(36.16,97.86) | 4.09(1.64,7.74) | 122.00(69.87,189.26) | 5.50(2.45,9.84) | 1.22(0.97,1.48) |
| Kenya | 123.20(73.79,183.09) | 13.17(6.19,22.62) | 376.35(227.81,554.53) | 14.53(7.69,23.27) | 0.20(0.16,0.25) |
| Kiribati | 0.09(0.05,0.14) | 2.16(0.00,96.56) | 0.20(0.11,0.32) | 2.50(0.00,52.88) | 0.50(0.36,0.64) |
| Kuwait | 2.32(1.21,3.74) | 3.97(0.09,16.90) | 15.91(8.50,25.47) | 5.58(1.33,13.14) | 1.13(0.93,1.33) |
| Kyrgyzstan | 71.00(43.46,104.26) | 19.18(8.51,34.12) | 170.01(100.68,259.66) | 30.00(14.46,51.74) | 1.50(1.28,1.72) |
| Lao People's Democratic Republic | 12.12(7.48,17.33) | 5.07(1.30,11.56) | 45.26(28.12,65.67) | 8.66(3.58,16.00) | 1.67(1.58,1.76) |
| Latvia | 16.95(8.39,28.42) | 3.62(0.79,8.76) | 22.33(11.28,37.07) | 4.25(1.08,9.72) | 0.39(0.19,0.59) |
| Lebanon | 8.43(4.44,13.44) | 3.19(0.50,8.62) | 29.51(15.81,46.94) | 4.02(1.23,8.49) | 0.80(0.66,0.95) |
| Lesotho | 11.92(7.51,17.15) | 11.87(3.13,27.31) | 16.91(10.59,24.68) | 13.20(4.06,28.51) | 0.18(0.07,0.29) |
| Liberia | 7.89(4.64,11.66) | 5.64(1.02,14.69) | 15.28(9.24,22.48) | 7.08(1.98,15.70) | 0.78(0.71,0.84) |
| Libya | 5.90(3.10,9.36) | 2.73(0.31,8.11) | 20.03(10.84,32.04) | 3.64(0.98,8.20) | 1.05(0.91,1.19) |
| Lithuania | 27.28(13.22,45.79) | 4.67(1.21,10.47) | 42.80(21.38,71.58) | 5.64(1.75,11.88) | 0.32(0.15,0.49) |
| Luxembourg | 19.42(13.01,27.30) | 27.23(9.71,55.56) | 42.31(28.02,59.06) | 31.81(14.00,57.27) | 0.55(0.42,0.67) |
| Madagascar | 53.34(33.98,76.09) | 9.13(4.03,16.30) | 125.92(79.92,179.08) | 10.65(5.36,17.53) | 0.45(0.42,0.48) |
| Malawi | 50.07(31.67,72.33) | 11.30(4.88,20.55) | 112.79(70.71,165.17) | 13.71(6.71,23.38) | 0.63(0.56,0.70) |
| Malaysia | 39.98(23.25,61.01) | 3.80(1.41,7.46) | 178.97(105.62,273.26) | 5.08(2.46,8.74) | 1.07(1.00,1.15) |
| Maldives | 0.54(0.31,0.81) | 5.47(0.00,53.58) | 3.55(2.11,5.34) | 9.94(0.78,34.00) | 1.99(1.92,2.06) |
| Mali | 25.10(15.03,37.19) | 5.49(1.84,11.21) | 60.92(37.63,88.28) | 6.15(2.68,10.98) | 0.31(0.28,0.35) |
| Malta | 12.97(8.50,18.17) | 23.68(6.90,52.31) | 34.98(23.28,48.53) | 26.79(11.33,49.19) | 0.49(0.40,0.58) |
| Marshall Islands | 0.06(0.03,0.09) | 3.24(0.00,210.87) | 0.16(0.09,0.25) | 4.19(0.00,108.62) | 0.90(0.79,1.01) |
| Mauritania | 7.60(4.56,10.97) | 6.57(1.20,16.98) | 23.93(14.54,34.53) | 9.40(3.16,18.90) | 1.01(0.92,1.09) |
| Mauritius | 6.89(3.92,10.55) | 7.75(1.18,21.50) | 27.75(16.25,42.15) | 11.33(3.81,23.25) | 0.94(0.81,1.08) |
| Mexico | 2470.78(1491.19,3653.43) | 50.18(28.77,76.64) | 6960.17(4272.60,10280.87) | 44.92(26.75,67.64) | -0.22(-0.29,-0.16) |
| Micronesia (Federated States of) | 0.19(0.10,0.30) | 3.24(0.00,72.81) | 0.40(0.22,0.62) | 4.56(0.00,55.75) | 1.18(1.08,1.28) |
| Monaco | 1.12(0.71,1.64) | 12.14(0.05,71.90) | 1.61(1.06,2.30) | 12.90(0.25,61.58) | 0.38(0.20,0.56) |
| Mongolia | 8.67(5.80,12.21) | 7.17(1.72,17.56) | 23.05(14.21,33.87) | 9.28(3.14,19.05) | 1.12(0.95,1.29) |
| Montenegro | 6.67(3.46,10.69) | 8.63(1.07,24.96) | 14.60(7.95,22.83) | 10.96(2.57,25.76) | 0.96(0.79,1.12) |
| Morocco | 41.24(21.65,65.48) | 2.43(0.80,4.91) | 149.75(82.36,233.61) | 3.55(1.55,6.29) | 1.20(1.03,1.36) |
| Mozambique | 73.47(46.67,104.45) | 10.96(5.11,18.88) | 155.08(96.61,223.41) | 12.82(6.47,21.05) | 0.39(0.35,0.44) |
| Myanmar | 143.88(88.22,212.97) | 5.29(2.60,8.96) | 547.90(337.78,800.78) | 9.32(5.15,14.60) | 1.74(1.64,1.84) |
| Namibia | 9.24(5.73,13.38) | 11.88(2.62,29.19) | 24.34(14.86,35.89) | 15.61(5.32,31.88) | 0.90(0.82,0.97) |
| Nauru | 0.02(0.01,0.03) | 3.71(0.00,752.02) | 0.03(0.02,0.05) | 4.85(0.00,596.20) | 0.94(0.74,1.14) |
| Nepal | 218.91(144.16,308.89) | 20.86(11.58,32.90) | 997.62(644.27,1389.78) | 35.03(20.91,51.43) | 1.56(1.48,1.64) |
| Netherlands | 2036.35(1337.61,2828.67) | 78.86(49.06,113.66) | 3764.31(2501.33,5189.34) | 81.95(52.34,116.09) | 0.39(0.23,0.54) |
| New Zealand | 396.16(238.37,595.86) | 76.77(40.51,125.12) | 811.88(501.07,1185.79) | 73.46(41.45,113.57) | 0.01(-0.06,0.08) |
| Nicaragua | 47.28(35.87,60.96) | 27.08(14.37,44.85) | 218.23(162.43,287.98) | 37.70(23.91,55.83) | 1.13(1.10,1.17) |
| Niger | 16.51(9.99,24.31) | 5.36(1.55,11.71) | 56.08(34.43,80.81) | 6.03(2.57,10.80) | 0.53(0.44,0.63) |
| Nigeria | 249.95(143.10,379.40) | 4.84(2.34,8.13) | 558.45(328.78,838.27) | 5.65(2.98,9.08) | 0.56(0.43,0.68) |
| Niue | 0.01(0.00,0.01) | 3.21(0.00,1334.35) | 0.01(0.01,0.02) | 4.41(0.00,1324.84) | 0.92(0.87,0.97) |
| North Macedonia | 11.33(6.16,17.80) | 4.92(1.00,12.24) | 32.16(17.71,50.32) | 7.25(2.35,14.93) | 1.40(1.34,1.45) |
| Northern Mariana Islands | 0.06(0.03,0.10) | 4.31(0.00,266.11) | 0.31(0.17,0.48) | 4.97(0.00,75.37) | 0.45(0.40,0.51) |
| Norway | 702.34(425.26,1043.96) | 78.28(43.00,123.63) | 929.34(573.24,1357.69) | 72.49(41.13,111.69) | 0.05(-0.09,0.19) |
| Oman | 1.86(0.98,3.00) | 2.63(0.03,12.39) | 8.49(4.64,13.18) | 4.31(0.72,11.40) | 1.56(1.41,1.72) |
| Pakistan | 1164.36(706.25,1722.38) | 17.71(9.96,27.46) | 2327.00(1431.56,3390.64) | 17.31(10.11,26.09) | 0.03(-0.10,0.15) |
| Palau | 0.05(0.03,0.07) | 3.96(0.00,322.69) | 0.14(0.08,0.23) | 5.24(0.00,150.90) | 0.90(0.85,0.95) |
| Palestine | 3.48(1.87,5.51) | 3.36(0.20,11.93) | 14.79(8.21,23.17) | 5.27(1.28,12.38) | 1.30(1.23,1.37) |
| Panama | 23.64(15.63,32.61) | 13.35(5.01,25.91) | 92.05(60.80,130.07) | 16.81(8.49,28.20) | 0.66(0.61,0.70) |
| Papua New Guinea | 5.16(2.74,8.19) | 2.59(0.26,8.04) | 15.17(8.49,23.23) | 2.90(0.72,6.65) | 0.36(0.26,0.47) |
| Paraguay | 17.29(9.24,27.88) | 6.55(1.62,15.28) | 65.81(34.72,108.15) | 9.24(3.39,18.33) | 0.97(0.91,1.03) |
| Peru | 206.98(133.55,295.06) | 14.92(8.06,23.84) | 922.82(550.64,1386.74) | 22.67(12.42,35.90) | 1.42(1.39,1.45) |
| Philippines | 432.51(262.40,632.52) | 13.17(7.05,20.83) | 1288.70(807.36,1849.49) | 13.34(7.79,20.03) | -0.01(-0.05,0.03) |
| Poland | 545.22(275.57,905.51) | 9.52(4.26,16.88) | 1024.14(550.04,1635.15) | 10.44(5.15,17.50) | 0.48(0.24,0.72) |
| Portugal | 372.03(243.14,516.28) | 19.92(11.44,30.14) | 848.13(558.90,1182.16) | 27.12(16.42,40.02) | 1.04(0.94,1.13) |
| Puerto Rico | 83.96(57.16,117.00) | 18.09(9.33,30.22) | 222.34(145.75,318.79) | 23.61(13.07,37.79) | 0.88(0.83,0.94) |
| Qatar | 0.35(0.19,0.56) | 4.13(0.00,55.77) | 5.91(3.14,9.59) | 7.48(0.86,22.60) | 1.90(1.77,2.02) |
| Republic of Korea | 676.83(453.91,926.24) | 20.26(12.37,29.58) | 4046.25(2748.10,5413.85) | 32.22(21.07,44.27) | 1.70(1.50,1.91) |
| Republic of Moldova | 18.48(9.48,29.82) | 3.29(0.79,7.60) | 35.72(18.66,58.09) | 4.42(1.38,9.29) | 0.82(0.60,1.04) |
| Romania | 172.07(93.80,272.94) | 4.74(2.09,8.47) | 347.73(193.49,544.24) | 6.95(3.34,11.83) | 1.32(1.25,1.39) |
| Russian Federation | 716.67(340.93,1204.66) | 3.04(1.30,5.40) | 1163.03(575.75,1925.31) | 3.52(1.60,6.09) | 0.53(0.16,0.91) |
| Rwanda | 33.90(21.42,49.45) | 10.46(4.11,20.13) | 101.72(62.33,147.72) | 13.96(6.56,23.82) | 1.09(0.99,1.19) |
| Saint Kitts and Nevis | 1.09(0.83,1.40) | 20.65(0.20,118.55) | 1.91(1.30,2.68) | 21.31(0.76,92.61) | -0.08(-0.41,0.25) |
| Saint Lucia | 2.55(2.07,3.14) | 22.56(2.34,79.40) | 8.36(6.71,10.35) | 27.72(8.72,62.43) | 0.60(0.42,0.78) |
| Saint Vincent and the Grenadines | 1.02(0.75,1.36) | 11.06(0.07,66.86) | 2.26(1.67,3.00) | 12.18(0.79,47.25) | 0.33(0.20,0.46) |
| Samoa | 0.35(0.19,0.56) | 3.45(0.00,47.09) | 0.74(0.41,1.16) | 4.37(0.00,34.31) | 0.65(0.59,0.70) |
| San Marino | 0.57(0.36,0.84) | 12.36(0.00,115.20) | 1.10(0.70,1.62) | 11.99(0.05,71.85) | 0.06(-0.14,0.27) |
| Sao Tome and Principe | 0.51(0.31,0.75) | 6.33(0.00,63.28) | 1.07(0.66,1.55) | 8.67(0.03,52.80) | 0.87(0.77,0.97) |
| Saudi Arabia | 16.20(8.52,26.10) | 2.62(0.61,6.18) | 76.75(41.95,119.32) | 4.30(1.69,7.99) | 1.78(1.57,2.00) |
| Senegal | 24.36(14.65,35.49) | 6.47(2.16,13.09) | 72.07(44.31,104.21) | 8.04(3.60,14.09) | 0.56(0.51,0.61) |
| Serbia | 146.88(85.43,225.76) | 9.86(4.59,17.27) | 247.53(136.27,389.36) | 11.02(5.09,19.15) | 0.62(0.47,0.78) |
| Seychelles | 0.26(0.15,0.40) | 3.61(0.00,61.85) | 0.71(0.41,1.07) | 4.95(0.00,39.91) | 0.98(0.90,1.05) |
| Sierra Leone | 14.13(8.50,20.80) | 5.80(1.55,13.08) | 27.84(16.94,39.91) | 6.65(2.36,12.99) | 0.45(0.37,0.53) |
| Singapore | 27.59(17.48,40.11) | 10.80(4.02,21.38) | 157.51(99.56,226.13) | 14.07(7.23,23.01) | 1.14(1.03,1.25) |
| Slovakia | 38.97(21.21,61.26) | 4.95(1.67,9.99) | 89.76(50.64,140.63) | 6.97(2.93,12.89) | 1.34(1.22,1.46) |
| Slovenia | 58.09(29.74,88.98) | 18.41(6.35,34.71) | 134.19(74.47,205.10) | 23.10(10.08,40.49) | 0.86(0.77,0.95) |
| Solomon Islands | 0.41(0.22,0.65) | 2.76(0.00,33.07) | 1.19(0.66,1.82) | 3.29(0.01,19.27) | 0.47(0.34,0.59) |
| Somalia | 22.22(13.93,32.42) | 9.95(3.40,20.44) | 67.56(42.37,97.36) | 10.48(4.74,18.42) | 0.20(0.13,0.28) |
| South Africa | 457.91(265.12,705.01) | 18.82(9.63,31.20) | 1078.41(632.30,1644.05) | 19.28(10.44,30.85) | 0.19(0.05,0.33) |
| South Sudan | 29.67(18.79,42.65) | 9.75(3.71,18.90) | 44.44(27.88,63.75) | 11.17(4.65,20.47) | 0.45(0.40,0.49) |
| Spain | 1946.09(1414.61,2494.92) | 26.65(18.38,35.53) | 4041.92(3253.97,4946.84) | 33.31(25.90,41.91) | 0.61(0.52,0.70) |
| Sri Lanka | 45.05(26.15,68.83) | 3.62(1.37,7.00) | 178.19(104.48,266.30) | 4.98(2.39,8.40) | 1.20(1.02,1.38) |
| Sudan | 23.48(12.32,37.64) | 2.19(0.60,4.82) | 65.06(35.60,100.71) | 3.08(1.18,5.80) | 1.16(0.97,1.36) |
| Suriname | 2.43(1.75,3.25) | 8.07(0.56,30.31) | 7.58(5.44,10.04) | 9.68(2.40,23.58) | 0.84(0.76,0.92) |
| Sweden | 1442.67(967.84,2031.18) | 73.65(46.35,108.31) | 1827.97(1246.32,2498.80) | 67.58(43.55,96.07) | -0.26(-0.28,-0.24) |
| Switzerland | 759.60(500.65,1062.56) | 57.35(34.56,85.20) | 1367.87(898.17,1896.17) | 60.40(37.11,87.58) | 0.24(0.11,0.37) |
| Syrian Arab Republic | 17.53(9.18,27.92) | 2.93(0.71,6.75) | 71.64(39.09,112.51) | 4.49(1.74,8.49) | 1.42(1.35,1.48) |
| Taiwan (Province of China) | 486.12(308.62,685.36) | 24.22(13.71,36.81) | 1554.54(1187.85,1934.89) | 27.24(19.65,35.45) | 0.47(0.29,0.66) |
| Tajikistan | 31.38(19.95,45.71) | 9.67(3.75,18.81) | 86.75(55.91,126.78) | 13.15(6.40,22.87) | 1.02(0.89,1.14) |
| Thailand | 244.31(139.50,370.20) | 6.10(2.93,10.24) | 1162.11(661.45,1799.06) | 8.16(4.30,13.23) | 0.98(0.91,1.06) |
| Timor-Leste | 1.44(0.86,2.13) | 5.29(0.05,27.29) | 9.47(6.02,13.61) | 8.80(2.06,21.39) | 1.64(1.56,1.72) |
| Togo | 8.77(5.15,12.70) | 6.58(1.28,16.40) | 32.65(20.02,46.69) | 8.01(3.00,15.24) | 0.65(0.59,0.71) |
| Tokelau | 0.01(0.00,0.01) | 3.00(0.00,1942.93) | 0.01(0.00,0.01) | 3.88(0.00,1924.54) | 0.88(0.79,0.97) |
| Tonga | 0.22(0.12,0.35) | 3.26(0.00,66.18) | 0.37(0.20,0.58) | 3.81(0.00,49.99) | 0.42(0.33,0.50) |
| Trinidad and Tobago | 25.38(19.70,32.48) | 24.49(11.56,44.13) | 71.32(54.28,92.26) | 27.61(15.80,43.80) | 0.34(0.32,0.37) |
| Tunisia | 17.05(8.98,27.29) | 2.80(0.67,6.50) | 67.49(37.05,106.94) | 3.98(1.54,7.62) | 1.17(1.01,1.34) |
| Turkey | 234.15(125.42,368.38) | 5.76(2.57,10.04) | 1079.00(576.81,1695.27) | 9.14(4.49,15.06) | 1.63(1.54,1.73) |
| Turkmenistan | 7.79(4.40,12.08) | 3.46(0.58,9.36) | 20.15(11.42,31.11) | 4.27(1.23,9.35) | 0.73(0.56,0.91) |
| Tuvalu | 0.03(0.01,0.04) | 3.08(0.00,450.57) | 0.05(0.03,0.08) | 4.00(0.00,292.48) | 0.85(0.80,0.90) |
| Uganda | 79.79(49.48,113.84) | 10.85(4.99,18.61) | 212.15(131.43,306.92) | 13.32(6.90,21.56) | 0.74(0.64,0.84) |
| Ukraine | 318.87(161.02,524.72) | 3.32(1.43,5.96) | 419.76(217.62,677.20) | 3.95(1.79,6.88) | 0.42(0.31,0.53) |
| United Arab Emirates | 1.19(0.63,1.86) | 3.47(0.01,20.41) | 17.53(9.43,27.84) | 5.17(1.30,11.88) | 1.50(1.29,1.71) |
| United Kingdom | 8051.59(5233.59,11353.22) | 67.55(42.72,97.01) | 12060.12(7961.88,16879.84) | 73.33(47.35,104.20) | 0.26(0.25,0.28) |
| United Republic of Tanzania | 149.65(94.06,219.19) | 11.85(6.02,19.81) | 399.85(248.00,579.59) | 14.20(7.75,22.33) | 0.54(0.51,0.57) |
| United States Virgin Islands | 1.38(1.00,1.83) | 14.06(0.25,71.01) | 4.84(3.54,6.33) | 19.19(3.42,53.56) | 0.75(0.55,0.94) |
| United States of America | 16684.84(10763.77,23434.70) | 39.78(25.18,56.59) | 37428.62(24611.57,51634.20) | 47.65(30.95,66.31) | 0.72(0.67,0.77) |
| Uruguay | 139.08(101.54,182.79) | 26.86(15.98,40.81) | 226.74(160.25,305.28) | 31.87(19.17,48.01) | 0.64(0.51,0.76) |
| Uzbekistan | 104.93(58.30,164.43) | 7.61(3.21,13.90) | 284.15(156.92,454.52) | 9.28(4.36,16.28) | 0.77(0.61,0.92) |
| Vanuatu | 0.21(0.11,0.33) | 3.06(0.00,62.73) | 0.70(0.39,1.10) | 3.65(0.00,29.73) | 0.54(0.46,0.61) |
| Venezuela (Bolivarian Republic of) | 265.94(181.59,373.28) | 23.71(13.92,36.84) | 1106.41(723.84,1591.52) | 29.02(17.63,43.85) | 0.65(0.57,0.73) |
| Viet Nam | 377.42(221.26,569.17) | 7.56(3.87,12.38) | 1875.44(1111.99,2791.08) | 15.45(8.63,23.86) | 2.07(1.94,2.20) |
| Yemen | 12.40(6.45,19.82) | 2.24(0.45,5.55) | 44.14(24.24,68.05) | 2.82(1.00,5.52) | 0.91(0.77,1.05) |
| Zambia | 24.23(15.15,34.86) | 7.84(2.75,15.70) | 70.39(43.57,102.94) | 9.78(4.39,17.34) | 0.79(0.64,0.94) |
| Zimbabwe | 34.17(22.45,48.10) | 7.25(3.00,13.52) | 54.64(35.27,78.00) | 7.06(3.18,12.58) | -0.37(-0.52, -0.23) |

**Abbreviations: ASR, age-standardised rate; CI: confidence interval; UI: uncertainly interval; EAPC, estimated annual percentage change.**

**Table S3. Prevalence cases and age-standardised rates of elderly-onset rheumatoid arthritis across 204 countries and territories in 1990 and 2021, and their estimated annual percentage changes from 1990 to 2021.**

| **Location** | **Number (95 % UI), 1990** | **ASRs (95 % UI),1990** | **Number (95 % UI), 2021** | **ASRs (95 % UI),2021** | **EAPCs (95% CI)** |
| --- | --- | --- | --- | --- | --- |
| Afghanistan | 826.87(711.09,969.10) | 98.01(78.20,122.33) | 1139.21(1000.80,1298.06) | 141.85(117.02,170.67) | 1.31(1.14,1.49) |
| Albania | 616.52(515.17,734.44) | 254.35(194.58,325.73) | 2645.32(2248.10,3102.12) | 441.52(359.87,536.31) | 2.05(1.95,2.14) |
| Algeria | 1721.00(1446.68,2053.63) | 116.16(92.68,144.74) | 7631.44(6470.49,8994.27) | 179.54(148.54,216.02) | 1.45(1.36,1.54) |
| American Samoa | 3.66(3.08,4.34) | 147.61(26.41,432.70) | 10.29(8.82,12.00) | 181.24(70.34,369.16) | 0.53(0.47,0.59) |
| Andorra | 29.08(24.56,34.09) | 392.94(213.90,643.39) | 82.19(69.93,96.33) | 425.47(282.15,608.76) | 0.36(0.31,0.41) |
| Angola | 1097.67(958.95,1252.47) | 269.52(220.79,325.04) | 4481.66(3926.36,5094.13) | 361.75(307.09,422.64) | 1.06(0.93,1.18) |
| Antigua and Barbuda | 24.61(21.56,28.05) | 357.29(195.17,588.29) | 61.15(53.37,69.93) | 456.92(299.08,660.26) | 0.67(0.58,0.76) |
| Argentina | 16972.42(14726.07,19502.80) | 407.54(347.92,474.92) | 41694.41(37124.79,46731.32) | 580.05(511.24,656.05) | 1.03(0.92,1.13) |
| Armenia | 597.47(501.40,713.73) | 178.06(136.63,228.90) | 1636.77(1412.15,1894.23) | 271.41(222.11,328.58) | 1.38(1.32,1.44) |
| Australia | 33985.06(29858.50,38373.49) | 1320.19(1146.77,1505.65) | 86175.29(75770.19,97563.08) | 1470.98(1284.17,1675.84) | 0.36(0.20,0.53) |
| Austria | 14225.83(12624.06,16083.69) | 913.45(796.52,1048.83) | 19880.82(17531.40,22513.57) | 871.84(757.47,1000.27) | -0.06(-0.22,0.09) |
| Azerbaijan | 843.37(700.63,1018.88) | 146.65(112.98,188.39) | 2378.39(2001.70,2833.37) | 189.58(152.64,234.32) | 1.08(0.73,1.43) |
| Bahamas | 59.79(52.16,68.00) | 340.65(222.03,491.14) | 210.08(184.21,239.95) | 426.16(321.67,552.39) | 0.73(0.64,0.83) |
| Bahrain | 57.30(50.29,65.29) | 322.58(210.35,468.29) | 595.40(529.81,666.93) | 600.69(489.98,725.92) | 1.96(1.82,2.10) |
| Bangladesh | 11948.34(10131.27,13981.54) | 224.14(186.37,266.66) | 62256.83(53585.97,72566.32) | 368.20(314.24,432.30) | 1.80(1.60,2.00) |
| Barbados | 216.18(190.79,243.61) | 565.34(430.64,722.28) | 513.84(457.74,580.71) | 738.21(598.75,904.98) | 0.66(0.52,0.79) |
| Belarus | 3539.61(2967.27,4176.15) | 207.83(168.01,252.76) | 7415.64(6376.68,8542.18) | 336.98(282.70,396.49) | 1.52(1.37,1.68) |
| Belgium | 16928.21(14791.79,19296.53) | 831.12(714.57,960.85) | 26659.89(23444.80,30465.28) | 895.20(777.19,1034.53) | 0.29(0.22,0.37) |
| Belize | 35.68(31.25,40.65) | 313.19(186.69,484.63) | 138.47(122.17,156.95) | 401.65(294.33,532.33) | 0.86(0.80,0.93) |
| Benin | 336.06(281.49,399.47) | 143.27(106.39,187.85) | 1033.77(877.65,1212.14) | 182.41(144.79,226.27) | 0.80(0.73,0.88) |
| Bermuda | 31.55(27.18,36.71) | 404.08(229.76,648.75) | 109.13(94.88,125.68) | 602.94(424.07,826.91) | 1.37(1.25,1.49) |
| Bhutan | 113.03(96.18,133.50) | 428.95(295.70,600.21) | 538.01(461.66,628.19) | 742.67(580.47,937.69) | 1.94(1.84,2.04) |
| Bolivia (Plurinational State of) | 2005.21(1761.23,2279.32) | 539.04(451.59,638.40) | 10411.56(9238.38,11784.75) | 935.55(813.29,1078.24) | 1.82(1.77,1.88) |
| Bosnia and Herzegovina | 2413.35(2089.75,2785.23) | 496.56(411.74,594.76) | 6351.14(5592.60,7272.05) | 749.87(643.12,878.57) | 1.67(1.52,1.83) |
| Botswana | 237.03(208.19,269.07) | 362.53(276.63,463.77) | 816.90(723.61,920.58) | 492.86(405.34,592.48) | 1.09(0.92,1.27) |
| Brazil | 33297.00(28594.96,38622.38) | 316.83(268.94,371.19) | 97995.89(85131.24,113027.76) | 310.21(267.68,359.89) | 0.10(0.03,0.17) |
| Brunei Darussalam | 66.20(57.96,75.81) | 617.53(410.52,885.38) | 330.49(291.67,375.02) | 825.03(646.95,1035.92) | 0.94(0.85,1.04) |
| Bulgaria | 4029.84(3436.53,4753.25) | 239.26(197.27,290.36) | 5984.13(5161.47,6947.12) | 310.35(260.43,368.87) | 0.86(0.71,1.01) |
| Burkina Faso | 723.32(603.08,865.37) | 141.77(108.96,181.30) | 1771.47(1489.53,2092.61) | 169.79(135.61,209.35) | 0.66(0.59,0.73) |
| Burundi | 627.41(544.41,718.80) | 235.23(187.32,289.93) | 1397.88(1214.25,1616.59) | 259.72(213.09,315.36) | 0.42(0.35,0.49) |
| Cabo Verde | 41.60(35.07,49.12) | 141.44(83.08,220.67) | 114.04(96.22,134.54) | 212.92(145.56,297.41) | 1.45(1.38,1.50) |
| Cambodia | 688.68(586.22,804.29) | 135.91(106.52,170.09) | 3534.35(3015.13,4104.01) | 241.01(198.33,288.55) | 1.89(1.84,1.93) |
| Cameroon | 847.11(709.92,1000.59) | 167.91(130.56,211.02) | 2834.15(2406.27,3324.83) | 206.50(168.39,250.63) | 0.67(0.51,0.84) |
| Canada | 38624.52(36496.97,40757.71) | 912.37(853.29,972.15) | 115759.05(109583.21,122430.06) | 1204.27(1133.28,1280.83) | 0.86(0.74,0.97) |
| Central African Republic | 362.38(320.41,409.09) | 294.95(233.01,366.85) | 676.52(598.58,765.34) | 303.87(247.75,369.01) | 0.15(0.07,0.22) |
| Chad | 419.63(350.24,496.60) | 125.76(94.26,162.51) | 871.02(732.72,1025.18) | 138.14(107.94,172.85) | 0.36(0.23,0.50) |
| Chile | 8719.72(7660.19,9916.25) | 711.68(611.28,825.42) | 43275.41(38260.85,48892.85) | 1303.19(1140.67,1485.46) | 1.98(1.90,2.06) |
| China | 644812.35(544798.67,765069.94) | 643.85(542.55,765.65) | 2012064.12(1718981.45,2358487.75) | 747.94(638.04,877.84) | 0.53(0.50,0.56) |
| Colombia | 8980.55(7947.07,10157.88) | 442.85(383.31,510.74) | 46899.31(42013.68,52351.36) | 672.04(596.29,756.62) | 1.35(1.21,1.49) |
| Comoros | 57.93(49.58,67.55) | 257.31(163.26,380.69) | 179.23(154.74,207.74) | 321.21(235.34,426.51) | 0.85(0.73,0.97) |
| Congo | 446.97(391.92,508.59) | 364.50(288.74,452.41) | 1319.21(1170.97,1490.25) | 469.57(393.27,558.08) | 0.96(0.84,1.07) |
| Cook Islands | 1.60(1.29,1.97) | 109.16(4.54,489.17) | 5.12(4.25,6.21) | 150.46(35.76,392.17) | 1.08(1.01,1.15) |
| Costa Rica | 1442.90(1286.27,1625.70) | 691.22(582.97,817.60) | 8090.81(7262.37,9054.14) | 1155.11(1013.13,1319.55) | 1.56(1.48,1.63) |
| Côte d'Ivoire | 631.62(522.50,763.89) | 151.80(115.04,197.09) | 2289.75(1936.71,2692.09) | 193.40(156.37,236.13) | 0.81(0.68,0.94) |
| Croatia | 4513.08(3960.17,5142.63) | 571.27(485.79,669.00) | 10961.90(9723.29,12379.00) | 918.74(798.81,1055.95) | 1.48(1.42,1.55) |
| Cuba | 4960.17(4345.87,5685.13) | 386.89(328.97,455.12) | 13562.48(11979.84,15347.18) | 557.94(484.05,641.43) | 1.13(1.06,1.19) |
| Cyprus | 1342.42(1161.33,1552.84) | 1252.00(1021.71,1522.12) | 4335.31(3760.61,4956.72) | 1589.88(1335.39,1869.09) | 1.12(0.92,1.32) |
| Czechia | 5945.81(5136.84,6856.97) | 323.48(271.88,381.98) | 12804.16(11221.06,14627.95) | 455.21(391.58,528.55) | 1.08(1.01,1.15) |
| Democratic People's Republic of Korea | 9713.45(8499.15,11093.88) | 535.44(458.60,623.02) | 28731.73(25558.13,32445.85) | 721.62(634.07,823.82) | 1.00(0.94,1.05) |
| Democratic Republic of the Congo | 4634.16(4027.33,5308.72) | 263.73(222.17,310.36) | 12533.46(10929.55,14423.40) | 320.15(273.97,374.48) | 0.59(0.45,0.74) |
| Denmark | 12851.49(11409.39,14425.72) | 1223.05(1065.97,1395.45) | 25329.53(22472.90,28516.42) | 1659.36(1453.04,1889.95) | 1.05(1.03,1.08) |
| Djibouti | 33.34(28.37,39.34) | 245.42(139.19,395.34) | 209.17(179.13,242.32) | 313.04(230.26,411.31) | 0.83(0.68,0.98) |
| Dominica | 25.31(22.18,29.03) | 328.04(180.47,540.15) | 40.48(35.42,46.15) | 375.26(229.23,570.39) | 0.28(0.11,0.46) |
| Dominican Republic | 984.87(844.05,1147.40) | 227.21(181.81,280.48) | 3805.36(3277.69,4432.42) | 314.05(261.32,376.73) | 0.94(0.80,1.09) |
| Ecuador | 3907.66(3449.91,4405.41) | 634.44(541.59,736.70) | 21965.69(19585.70,24654.80) | 1085.68(954.53,1233.90) | 1.65(1.53,1.77) |
| Egypt | 4064.69(3462.87,4779.94) | 135.13(111.32,163.48) | 16495.10(14303.39,19087.85) | 224.39(191.40,263.37) | 1.50(1.40,1.61) |
| El Salvador | 853.64(737.10,987.14) | 240.88(193.25,296.48) | 3027.86(2630.08,3482.24) | 394.52(329.72,469.05) | 1.67(1.55,1.79) |
| Equatorial Guinea | 58.35(51.09,67.02) | 272.59(177.76,397.57) | 274.66(239.71,314.91) | 522.67(400.23,669.25) | 2.52(2.34,2.71) |
| Eritrea | 259.14(225.54,298.35) | 245.10(186.38,316.08) | 856.77(748.13,982.99) | 302.90(245.88,369.95) | 0.65(0.61,0.68) |
| Estonia | 2073.82(1907.70,2253.82) | 775.72(681.92,878.59) | 3410.75(3155.94,3695.70) | 963.52(860.70,1078.23) | 1.01(0.86,1.16) |
| Eswatini | 136.34(119.69,154.46) | 439.56(319.84,583.00) | 322.03(286.39,361.95) | 513.61(405.40,639.92) | 0.50(0.30,0.70) |
| Ethiopia | 5933.83(4979.94,7084.71) | 269.81(220.19,329.73) | 11996.89(10193.76,14191.65) | 250.13(208.43,300.79) | -0.08(-0.18,0.01) |
| Fiji | 32.84(26.64,40.67) | 88.83(47.35,149.41) | 98.22(81.66,119.15) | 106.77(70.57,154.98) | 0.63(0.47,0.79) |
| Finland | 14165.05(12655.22,15898.30) | 1515.25(1330.26,1727.30) | 31292.24(27900.07,34981.26) | 1901.17(1675.25,2147.69) | 0.90(0.84,0.97) |
| France | 76852.86(66718.42,88391.56) | 708.75(610.63,820.55) | 151984.15(132155.04,174944.91) | 853.65(738.28,987.23) | 0.70(0.55,0.85) |
| Gabon | 267.36(233.35,305.04) | 383.71(293.32,489.79) | 661.88(585.39,746.37) | 552.28(449.69,669.11) | 1.21(1.11,1.29) |
| Gambia | 57.26(47.43,68.72) | 148.00(90.22,224.92) | 204.18(172.52,240.64) | 188.26(136.22,251.76) | 0.85(0.76,0.93) |
| Georgia | 1532.07(1281.14,1846.62) | 188.55(149.15,237.87) | 1797.45(1533.42,2097.64) | 220.94(179.17,269.11) | 0.41(0.27,0.55) |
| Germany | 107487.24(93410.56,123213.90) | 659.89(569.80,760.68) | 181255.42(157997.22,206224.63) | 737.05(639.31,842.21) | 0.32(0.18,0.47) |
| Ghana | 943.65(764.01,1148.41) | 136.28(102.65,175.73) | 3634.95(2991.25,4361.04) | 193.61(153.67,239.29) | 1.10(0.98,1.23) |
| Greece | 10531.42(9066.31,12231.15) | 526.16(443.68,622.00) | 17616.39(15394.66,20126.00) | 591.35(508.64,684.99) | 0.35(0.32,0.39) |
| Greenland | 15.60(13.65,17.86) | 436.52(207.06,791.13) | 66.11(58.52,74.98) | 744.10(500.89,1058.03) | 1.90(1.86,1.94) |
| Grenada | 18.43(15.69,21.57) | 194.88(94.26,346.68) | 40.05(34.40,46.52) | 288.36(171.94,446.06) | 0.92(0.72,1.12) |
| Guam | 11.72(9.72,14.18) | 133.21(52.33,269.60) | 47.70(40.51,56.05) | 172.35(104.79,262.95) | 0.78(0.65,0.92) |
| Guatemala | 1163.64(1014.97,1328.01) | 293.28(240.31,353.20) | 7607.21(6800.20,8535.22) | 569.93(497.43,653.17) | 2.05(1.91,2.20) |
| Guinea | 554.83(462.47,659.75) | 136.32(103.51,174.95) | 1018.52(864.96,1204.45) | 158.12(125.48,197.85) | 0.38(0.29,0.47) |
| Guinea-Bissau | 61.00(50.82,72.50) | 136.70(84.75,204.43) | 128.63(109.27,150.83) | 169.39(118.18,232.97) | 0.71(0.60,0.81) |
| Guyana | 68.00(57.50,80.80) | 154.72(99.22,228.56) | 166.43(143.09,194.33) | 212.97(154.33,286.19) | 0.93(0.85,1.01) |
| Haiti | 736.26(647.46,843.10) | 197.07(160.21,241.43) | 2032.38(1789.18,2300.85) | 252.98(212.51,298.34) | 0.82(0.78,0.86) |
| Honduras | 1545.70(1374.99,1729.18) | 657.18(554.11,770.68) | 8090.52(7276.32,9018.80) | 1075.70(945.34,1224.13) | 1.46(1.38,1.53) |
| Hungary | 11197.28(10110.12,12412.67) | 569.31(504.06,642.31) | 18477.74(16694.63,20474.59) | 706.84(628.98,794.03) | 0.70(0.53,0.87) |
| Iceland | 284.08(246.52,325.99) | 769.45(586.94,984.20) | 675.45(580.90,777.61) | 890.44(704.78,1099.77) | 0.49(0.39,0.58) |
| India | 296018.95(243076.43,361451.55) | 573.51(469.07,702.57) | 1312132.26(1095429.60,1574167.89) | 904.18(753.44,1086.44) | 1.61(1.53,1.70) |
| Indonesia | 10943.06(8913.07,13367.20) | 100.44(80.12,124.78) | 36965.73(30652.81,44349.22) | 131.10(107.50,158.76) | 0.73(0.67,0.80) |
| Iran (Islamic Republic of) | 4338.36(3662.45,5142.99) | 140.06(114.44,170.64) | 17972.63(15447.51,21041.35) | 195.05(165.01,231.46) | 1.10(1.03,1.17) |
| Iraq | 1322.53(1115.45,1565.68) | 146.32(116.27,182.01) | 6018.31(5184.00,6993.14) | 228.43(191.44,271.72) | 1.61(1.43,1.78) |
| Ireland | 10389.70(9248.09,11660.33) | 1932.62(1685.38,2208.71) | 24025.99(21224.44,26966.82) | 2364.72(2060.97,2686.03) | 0.89(0.74,1.05) |
| Israel | 3098.62(2633.31,3607.07) | 486.87(398.10,585.56) | 10444.62(8976.15,12095.40) | 658.38(554.17,776.15) | 1.05(1.02,1.09) |
| Italy | 110657.82(92573.72,132208.20) | 936.48(778.40,1124.91) | 165823.42(140312.90,196635.61) | 919.83(774.26,1095.58) | -0.02(-0.04,0.01) |
| Jamaica | 605.51(525.78,701.48) | 263.55(209.70,328.78) | 1396.23(1222.67,1594.78) | 356.88(295.24,428.14) | 0.91(0.80,1.02) |
| Japan | 287917.74(241803.35,343586.17) | 1319.78(1103.98,1580.23) | 473300.59(405532.47,553459.87) | 1068.23(912.47,1252.45) | -0.51(-0.62,-0.40) |
| Jordan | 204.12(174.57,240.65) | 145.57(106.72,194.73) | 1873.34(1608.10,2190.99) | 230.49(188.31,281.11) | 1.77(1.64,1.90) |
| Kazakhstan | 2694.48(2257.48,3214.77) | 174.84(140.50,215.94) | 5441.13(4618.17,6376.82) | 245.30(202.24,294.63) | 1.38(1.07,1.70) |
| Kenya | 2554.85(2132.81,3072.87) | 273.04(218.37,340.23) | 7805.40(6600.01,9266.25) | 301.43(248.77,365.20) | 0.23(0.17,0.29) |
| Kiribati | 3.51(2.92,4.20) | 85.31(14.17,255.62) | 8.32(7.07,9.86) | 101.60(34.87,222.36) | 0.54(0.46,0.63) |
| Kuwait | 148.75(127.00,174.54) | 254.72(181.29,346.66) | 1073.77(935.66,1221.99) | 376.59(307.46,453.29) | 1.53(1.29,1.77) |
| Kyrgyzstan | 2371.27(2091.63,2680.48) | 640.56(541.06,752.02) | 5456.49(4776.78,6199.34) | 962.74(819.08,1121.38) | 1.52(1.33,1.72) |
| Lao People's Democratic Republic | 318.76(273.32,370.18) | 133.40(101.23,171.54) | 1136.69(972.84,1318.73) | 217.61(174.72,266.46) | 1.65(1.57,1.74) |
| Latvia | 2623.79(2428.00,2846.33) | 560.83(498.54,631.16) | 3493.34(3224.57,3782.59) | 664.91(592.75,743.29) | 0.56(0.46,0.65) |
| Lebanon | 522.43(447.92,615.10) | 197.80(154.25,252.04) | 2086.64(1796.22,2408.21) | 283.92(233.23,341.02) | 1.30(1.15,1.46) |
| Lesotho | 365.52(320.80,413.05) | 364.26(285.67,453.31) | 559.10(497.01,628.23) | 436.43(354.61,530.30) | 0.41(0.27,0.55) |
| Liberia | 197.68(164.37,235.53) | 141.44(100.32,191.47) | 399.40(337.99,468.49) | 185.03(140.33,237.61) | 1.07(0.94,1.21) |
| Libya | 288.88(244.78,345.24) | 133.48(99.38,177.28) | 1058.40(907.25,1230.37) | 192.06(154.09,236.09) | 1.38(1.28,1.47) |
| Lithuania | 3859.93(3561.79,4186.98) | 661.19(590.25,739.28) | 5972.96(5528.77,6462.17) | 786.91(709.31,872.38) | 0.41(0.34,0.47) |
| Luxembourg | 481.48(418.99,554.59) | 675.01(532.49,844.98) | 1036.56(902.03,1189.54) | 779.32(634.64,946.65) | 0.56(0.44,0.68) |
| Madagascar | 1183.51(1011.44,1380.94) | 202.53(162.58,249.11) | 2934.01(2553.02,3401.24) | 248.14(207.62,297.48) | 0.63(0.59,0.68) |
| Malawi | 1123.97(967.08,1312.80) | 253.70(204.74,312.79) | 2641.09(2288.39,3043.27) | 321.06(266.90,383.33) | 0.81(0.72,0.90) |
| Malaysia | 1101.45(895.82,1341.22) | 104.82(79.76,134.66) | 5152.10(4306.28,6173.08) | 146.33(118.68,179.75) | 1.14(1.10,1.17) |
| Maldives | 19.29(16.72,22.15) | 197.02(98.94,342.04) | 121.46(105.81,139.27) | 339.91(242.37,460.09) | 2.02(1.76,2.28) |
| Mali | 579.92(479.42,694.83) | 126.93(95.75,163.83) | 1512.01(1276.51,1777.93) | 152.70(121.94,188.11) | 0.65(0.57,0.73) |
| Malta | 331.56(287.06,379.25) | 605.23(465.12,765.58) | 928.73(808.77,1063.34) | 711.20(577.38,864.74) | 0.53(0.45,0.62) |
| Marshall Islands | 2.25(1.94,2.62) | 122.41(12.22,444.71) | 6.28(5.43,7.19) | 162.60(48.40,380.46) | 0.89(0.78,1.01) |
| Mauritania | 186.80(157.83,220.04) | 161.47(115.97,217.07) | 591.92(502.15,692.30) | 232.47(180.34,292.92) | 1.14(1.03,1.25) |
| Mauritius | 177.59(147.12,214.89) | 199.86(139.89,276.42) | 747.78(633.26,887.78) | 305.32(238.81,387.13) | 1.10(0.97,1.23) |
| Mexico | 62058.60(52613.25,72769.06) | 1260.31(1059.38,1488.60) | 206231.85(179087.21,238234.53) | 1330.96(1150.43,1543.68) | 0.16(0.12,0.21) |
| Micronesia (Federated States of) | 7.58(6.50,8.82) | 128.43(42.47,285.61) | 16.86(14.71,19.30) | 191.86(93.08,341.79) | 1.30(1.28,1.32) |
| Monaco | 35.70(30.05,42.60) | 385.87(219.21,621.11) | 51.53(43.53,60.45) | 412.52(252.71,622.27) | 0.38(0.21,0.55) |
| Mongolia | 266.91(230.55,307.36) | 220.86(166.94,284.41) | 886.92(770.23,1016.44) | 356.86(288.41,434.91) | 1.91(1.69,2.12) |
| Montenegro | 278.36(235.56,326.71) | 360.27(267.18,471.27) | 610.68(521.04,712.38) | 458.51(358.33,575.63) | 1.06(0.86,1.26) |
| Morocco | 1932.38(1639.01,2285.68) | 113.78(91.89,140.21) | 7510.14(6482.94,8724.56) | 177.82(149.78,210.95) | 1.44(1.32,1.55) |
| Mozambique | 1583.37(1360.49,1848.34) | 236.24(192.34,288.64) | 3549.99(3067.96,4084.40) | 293.35(244.63,348.02) | 0.58(0.52,0.64) |
| Myanmar | 3783.38(3202.11,4448.11) | 139.18(113.75,168.51) | 13915.93(11932.48,16188.61) | 236.72(199.35,279.65) | 1.75(1.63,1.86) |
| Namibia | 266.42(233.54,304.90) | 342.34(262.83,438.33) | 717.35(631.87,816.48) | 460.06(374.25,560.82) | 1.03(0.88,1.17) |
| Nauru | 0.72(0.62,0.84) | 144.70(0.42,1060.32) | 1.29(1.12,1.48) | 203.74(6.32,1005.27) | 1.06(0.82,1.30) |
| Nepal | 3770.41(3207.74,4433.60) | 359.26(295.16,435.07) | 17827.69(15322.89,20782.87) | 625.91(529.48,739.65) | 1.78(1.75,1.80) |
| Netherlands | 42033.09(36961.62,47708.05) | 1627.88(1416.91,1864.32) | 73427.67(64006.80,83839.01) | 1598.52(1382.65,1837.57) | 0.17(-0.01,0.35) |
| New Zealand | 9354.18(7776.86,11179.51) | 1812.70(1473.73,2206.96) | 18249.93(15413.63,21715.92) | 1651.24(1372.68,1991.15) | -0.25(-0.29,-0.22) |
| Nicaragua | 875.44(768.80,994.33) | 501.33(409.69,605.94) | 4488.55(3966.55,5115.68) | 775.33(664.00,908.21) | 1.52(1.49,1.55) |
| Niger | 374.44(308.52,448.83) | 121.55(89.29,159.83) | 1344.93(1131.54,1593.65) | 144.62(114.69,179.99) | 0.72(0.61,0.83) |
| Nigeria | 7019.73(5770.00,8476.03) | 136.01(108.93,167.77) | 15999.57(13389.63,19048.58) | 161.90(133.20,195.50) | 0.65(0.45,0.85) |
| Niue | 0.36(0.31,0.42) | 129.42(0.00,1626.57) | 0.52(0.44,0.60) | 183.05(0.06,1724.39) | 1.09(1.07,1.11) |
| North Macedonia | 519.71(434.44,624.00) | 225.55(171.23,292.92) | 1491.42(1257.33,1755.09) | 335.99(267.81,414.34) | 1.45(1.40,1.50) |
| Northern Mariana Islands | 2.49(2.11,2.95) | 169.96(19.01,593.67) | 12.11(10.33,14.13) | 197.15(81.77,384.99) | 0.36(0.27,0.46) |
| Norway | 18403.82(15363.93,22068.44) | 2051.10(1685.33,2492.19) | 21221.89(17928.74,25304.70) | 1655.38(1378.11,1998.33) | -0.62(-0.69,-0.54) |
| Oman | 81.59(67.66,98.25) | 115.25(74.16,169.09) | 408.35(343.94,482.97) | 207.18(156.54,267.90) | 2.02(1.88,2.16) |
| Pakistan | 28424.38(24215.95,33352.81) | 432.31(363.68,512.74) | 54595.15(46548.44,64091.40) | 406.20(343.20,480.57) | 0.09(-0.08,0.25) |
| Palau | 1.87(1.60,2.19) | 157.44(11.10,632.21) | 5.92(5.05,6.84) | 214.59(59.95,515.15) | 0.92(0.87,0.97) |
| Palestine | 163.77(139.91,191.78) | 157.89(113.46,213.00) | 660.13(565.59,769.65) | 235.40(185.41,294.55) | 1.20(1.14,1.25) |
| Panama | 687.30(604.44,780.18) | 388.12(314.66,472.61) | 2948.23(2602.60,3330.28) | 538.32(457.12,629.09) | 1.01(0.97,1.04) |
| Papua New Guinea | 180.80(150.86,217.61) | 90.73(64.11,124.72) | 542.79(458.95,641.86) | 103.81(79.93,132.63) | 0.37(0.27,0.47) |
| Paraguay | 1056.08(924.89,1202.78) | 400.16(328.22,482.24) | 5056.55(4559.71,5625.72) | 710.14(621.91,811.00) | 1.56(1.42,1.70) |
| Peru | 8365.62(7421.16,9397.67) | 603.08(522.89,691.32) | 43857.68(39168.75,49091.05) | 1077.29(952.61,1216.56) | 2.07(1.96,2.17) |
| Philippines | 9345.98(7799.69,11151.89) | 284.65(232.31,346.01) | 25389.75(21313.88,30136.66) | 262.77(217.64,315.44) | -0.14(-0.19,-0.08) |
| Poland | 46639.03(40773.06,53455.96) | 814.50(705.16,941.49) | 70600.07(61930.24,80284.12) | 719.98(626.60,824.42) | -0.32(-0.38,-0.26) |
| Portugal | 10715.60(9509.45,12114.57) | 573.83(499.06,660.41) | 25725.86(22815.97,28891.06) | 822.63(720.15,934.56) | 1.21(1.07,1.34) |
| Puerto Rico | 1829.82(1586.35,2115.92) | 394.34(325.25,475.85) | 6052.92(5292.99,6908.00) | 642.85(547.10,751.17) | 1.64(1.55,1.72) |
| Qatar | 14.43(11.98,17.44) | 168.08(72.10,323.45) | 246.68(205.86,293.06) | 312.23(226.18,415.92) | 1.92(1.77,2.06) |
| Republic of Korea | 17957.96(15818.53,20323.88) | 537.65(466.25,616.91) | 102908.20(90674.02,116125.09) | 819.44(717.33,930.02) | 1.58(1.36,1.80) |
| Republic of Moldova | 1183.49(1014.16,1386.01) | 211.06(169.90,260.54) | 2462.12(2143.68,2820.56) | 304.63(254.12,362.10) | 1.14(0.92,1.35) |
| Romania | 8050.60(6663.95,9647.32) | 221.86(179.26,271.22) | 17311.65(14798.35,20281.89) | 346.09(291.09,411.09) | 1.54(1.48,1.60) |
| Russian Federation | 92871.71(81946.17,105090.61) | 393.78(345.08,448.29) | 144550.46(129040.36,161409.43) | 437.24(388.20,490.62) | 0.32(0.25,0.40) |
| Rwanda | 834.04(728.09,960.45) | 257.20(208.51,315.52) | 2611.46(2283.44,2995.78) | 358.41(300.66,426.14) | 1.38(1.21,1.54) |
| Saint Kitts and Nevis | 18.97(16.72,21.55) | 359.77(183.74,621.36) | 46.02(40.28,52.48) | 514.41(322.07,768.36) | 1.10(0.83,1.37) |
| Saint Lucia | 37.67(33.08,42.88) | 333.47(201.67,511.50) | 139.91(123.34,157.91) | 463.70(339.81,611.63) | 1.08(0.91,1.25) |
| Saint Vincent and the Grenadines | 19.21(16.34,22.55) | 207.68(101.63,367.12) | 47.02(40.26,54.83) | 253.21(155.08,384.48) | 0.66(0.61,0.70) |
| Samoa | 13.99(11.95,16.51) | 137.67(60.63,261.83) | 30.31(26.22,34.99) | 178.07(100.84,285.92) | 0.71(0.65,0.77) |
| San Marino | 17.50(14.51,20.87) | 381.35(174.98,696.11) | 35.57(29.80,42.39) | 386.75(218.30,622.03) | 0.17(0.02,0.33) |
| Sao Tome and Principe | 12.90(10.80,15.37) | 159.19(66.04,310.84) | 26.94(22.73,31.57) | 218.74(116.62,362.75) | 1.08(0.96,1.19) |
| Saudi Arabia | 705.60(588.70,845.45) | 114.15(87.70,146.31) | 3576.66(3018.76,4246.83) | 200.19(162.99,244.97) | 1.88(1.74,2.02) |
| Senegal | 562.82(469.50,668.03) | 149.55(113.72,191.49) | 1719.81(1446.82,2032.20) | 191.92(153.24,236.86) | 0.73(0.67,0.79) |
| Serbia | 5338.49(4770.83,5969.74) | 358.49(311.35,411.18) | 11844.92(10328.43,13493.36) | 527.51(451.14,611.14) | 1.31(1.23,1.40) |
| Seychelles | 8.65(7.18,10.42) | 119.43(40.44,261.46) | 23.55(19.84,27.88) | 165.01(84.72,282.52) | 0.99(0.94,1.04) |
| Sierra Leone | 335.24(278.13,398.48) | 137.63(101.15,180.47) | 686.08(578.35,806.90) | 163.96(127.18,206.61) | 0.59(0.47,0.72) |
| Singapore | 808.67(687.49,948.28) | 316.65(249.45,395.72) | 4720.19(3993.47,5519.75) | 421.71(345.80,506.32) | 1.10(1.00,1.20) |
| Slovakia | 2010.03(1725.54,2337.85) | 255.20(208.86,309.10) | 4778.82(4178.28,5474.76) | 371.31(314.88,436.80) | 1.25(1.20,1.31) |
| Slovenia | 2617.74(2324.94,2944.05) | 829.75(707.29,967.51) | 6806.05(6069.39,7574.32) | 1171.76(1018.81,1333.74) | 1.30(1.18,1.41) |
| Solomon Islands | 16.73(14.39,19.43) | 111.75(53.08,201.78) | 53.96(47.01,61.70) | 149.72(95.84,219.58) | 0.82(0.76,0.88) |
| Somalia | 574.89(501.08,658.34) | 257.35(205.10,318.11) | 1727.88(1512.24,1974.97) | 268.04(222.91,320.18) | 0.16(0.12,0.20) |
| South Africa | 17876.56(15599.25,20527.62) | 734.82(631.18,855.41) | 38071.21(33313.18,43763.99) | 680.65(589.21,789.79) | -0.08(-0.23,0.07) |
| South Sudan | 671.15(576.53,783.60) | 220.55(174.31,276.19) | 1024.65(885.41,1186.58) | 257.57(208.15,315.74) | 0.57(0.51,0.63) |
| Spain | 48727.76(44105.45,53852.87) | 667.28(598.35,743.71) | 100932.46(93217.50,109867.52) | 831.68(763.19,910.68) | 0.66(0.58,0.74) |
| Sri Lanka | 1315.68(1072.46,1615.44) | 105.67(81.06,136.23) | 5374.92(4452.24,6463.92) | 150.36(120.92,185.29) | 1.29(1.13,1.45) |
| Sudan | 1016.91(859.08,1206.25) | 94.66(74.71,118.80) | 3049.61(2613.88,3539.79) | 144.45(119.11,173.28) | 1.44(1.25,1.62) |
| Suriname | 50.31(42.34,59.43) | 166.75(101.28,253.89) | 167.53(143.21,197.17) | 214.16(154.33,289.81) | 0.97(0.88,1.05) |
| Sweden | 30218.27(25556.03,35776.47) | 1542.76(1288.78,1845.55) | 37087.16(31635.77,43584.56) | 1371.07(1156.68,1626.47) | -0.40(-0.42,-0.38) |
| Switzerland | 15045.88(13056.82,17455.60) | 1136.04(969.02,1337.68) | 27451.93(23858.51,31597.76) | 1212.11(1040.12,1410.64) | 0.27(0.18,0.37) |
| Syrian Arab Republic | 779.66(656.34,934.56) | 130.44(101.56,166.70) | 3331.98(2840.12,3938.21) | 209.02(171.68,254.89) | 1.60(1.54,1.67) |
| Taiwan (Province of China) | 11777.47(10242.67,13456.64) | 586.89(500.57,682.00) | 36644.01(35140.97,38350.99) | 642.15(609.39,678.83) | 0.69(0.45,0.93) |
| Tajikistan | 933.43(811.32,1067.47) | 287.67(233.12,349.32) | 2477.90(2162.37,2839.59) | 375.71(314.19,446.68) | 0.80(0.69,0.91) |
| Thailand | 8054.57(6881.33,9413.95) | 201.19(167.85,239.94) | 41936.56(36074.71,48739.72) | 294.50(250.73,345.33) | 1.30(1.23,1.38) |
| Timor-Leste | 35.89(30.29,42.52) | 131.67(75.14,210.46) | 225.58(192.85,263.23) | 209.74(154.89,276.17) | 1.57(1.51,1.62) |
| Togo | 198.33(164.81,237.33) | 148.81(105.50,202.23) | 778.89(660.32,917.38) | 191.03(149.83,240.05) | 0.86(0.73,0.98) |
| Tokelau | 0.21(0.18,0.25) | 112.38(0.00,2202.52) | 0.32(0.27,0.37) | 163.95(0.00,2292.57) | 1.23(1.18,1.28) |
| Tonga | 8.41(7.09,10.03) | 127.04(43.36,278.31) | 15.37(13.19,18.02) | 159.53(73.24,295.49) | 0.64(0.58,0.69) |
| Trinidad and Tobago | 473.45(420.04,532.90) | 456.82(367.45,559.75) | 1580.48(1401.16,1771.60) | 611.91(514.45,718.61) | 1.06(0.99,1.12) |
| Tunisia | 786.19(664.06,934.35) | 128.99(100.82,163.45) | 3365.54(2873.94,3924.62) | 198.39(163.27,238.69) | 1.48(1.35,1.60) |
| Turkey | 13244.96(11782.78,14992.73) | 325.94(284.75,374.90) | 65796.73(58790.03,73776.82) | 557.30(493.94,629.42) | 1.91(1.83,1.99) |
| Turkmenistan | 330.82(277.94,394.69) | 147.07(109.46,193.66) | 936.80(807.22,1094.04) | 198.41(159.37,245.86) | 1.06(0.87,1.25) |
| Tuvalu | 0.95(0.81,1.12) | 113.80(1.18,690.16) | 2.10(1.82,2.45) | 159.53(14.25,600.44) | 1.05(1.03,1.07) |
| Uganda | 1789.39(1541.35,2086.25) | 243.43(199.35,296.26) | 4989.30(4322.00,5732.19) | 313.35(263.41,369.45) | 0.93(0.81,1.05) |
| Ukraine | 17326.88(14455.43,20859.74) | 180.52(148.16,220.29) | 23997.81(20365.90,28262.50) | 226.06(189.22,269.36) | 0.60(0.54,0.65) |
| United Arab Emirates | 59.80(51.19,69.81) | 174.43(111.24,257.34) | 775.33(668.15,902.28) | 228.68(182.41,284.07) | 0.99(0.83,1.16) |
| United Kingdom | 184798.79(159330.81,216144.03) | 1550.34(1330.12,1820.96) | 275624.47(239458.81,318921.03) | 1675.91(1450.18,1945.91) | 0.25(0.23,0.28) |
| United Republic of Tanzania | 3281.80(2810.03,3834.62) | 259.90(214.38,313.44) | 9238.00(7992.57,10674.33) | 328.11(277.69,386.39) | 0.70(0.65,0.74) |
| United States of America | 398296.94(348715.67,456394.54) | 949.51(828.56,1091.17) | 854511.26(750581.41,972079.80) | 1087.96(953.48,1240.12) | 0.56(0.52,0.61) |
| United States Virgin Islands | 26.03(22.04,30.62) | 265.74(141.06,444.65) | 90.70(78.23,105.64) | 359.67(245.32,506.83) | 0.85(0.80,0.90) |
| Uruguay | 2575.71(2249.47,2951.37) | 497.50(416.71,591.00) | 5042.55(4437.43,5694.93) | 708.84(605.56,821.61) | 1.15(1.07,1.22) |
| Uzbekistan | 7223.28(6509.81,7981.15) | 523.98(460.82,591.80) | 20113.14(18283.67,22193.91) | 657.11(588.71,734.70) | 0.88(0.69,1.07) |
| Vanuatu | 7.62(6.44,8.99) | 109.83(35.59,246.25) | 26.85(23.03,31.02) | 139.06(75.65,228.06) | 0.72(0.66,0.77) |
| Venezuela (Bolivarian Republic of) | 7497.34(6755.52,8377.45) | 668.52(588.09,763.17) | 36633.52(33120.65,40598.86) | 960.89(859.42,1075.31) | 1.18(1.04,1.33) |
| Viet Nam | 10691.41(9070.89,12619.39) | 214.27(178.07,257.36) | 48977.80(41928.30,56970.95) | 403.53(342.15,473.25) | 1.97(1.91,2.02) |
| Yemen | 530.14(448.35,632.35) | 95.85(73.73,123.60) | 1972.45(1686.01,2307.52) | 126.20(102.78,153.78) | 1.06(0.93,1.20) |
| Zambia | 699.89(613.60,799.05) | 226.46(183.14,277.11) | 2185.67(1928.33,2484.14) | 303.58(256.01,358.87) | 1.03(0.84,1.22) |
| Zimbabwe | 1115.92(970.01,1277.37) | 236.62(192.94,286.12) | 1857.09(1636.53,2111.45) | 240.00(201.37,284.76) | -0.28(-0.47,-0.09) |

**Abbreviations: ASR, age-standardised rate; CI: confidence interval; UI: uncertainly interval; EAPC, estimated annual percentage change.**

**Table S4. Mortality cases and age-standardised rates of elderly-onset rheumatoid arthritis** **across 204 countries and territories in 1990 and 2021, and their estimated annual percentage changes from 1990 to 2021.**

| **Location** | **Number (95 % UI), 1990** | **ASRs (95 % UI),1990** | **Number (95 % UI), 2021** | **ASRs (95 % UI),2021** | **EAPCs (95% CI)** |
| --- | --- | --- | --- | --- | --- |
| Afghanistan | 8.29(3.52,16.77) | 0.98(0.10,3.19) | 14.53(6.54,27.82) | 1.81(0.31,5.01) | 2.34(2.15,2.52) |
| Albania | 5.26(3.41,7.60) | 2.17(0.33,6.28) | 6.60(3.80,10.55) | 1.10(0.17,3.19) | -2.03(-2.33,-1.74) |
| Algeria | 3.99(1.74,7.38) | 0.27(0.01,1.01) | 17.63(8.90,30.47) | 0.41(0.10,1.02) | 1.82(1.49,2.15) |
| American Samoa | 0.00(0.00,0.00) | 0.02(0.00,148.75) | 0.00(0.00,0.00) | 0.01(0.00,64.98) | -4.57(-6.76,-2.32) |
| Andorra | 0.04(0.02,0.06) | 0.51(0.00,51.59) | 0.06(0.03,0.10) | 0.30(0.00,20.16) | -1.38(-1.61,-1.15) |
| Angola | 1.33(0.34,11.64) | 0.33(0.00,5.04) | 2.61(0.58,26.63) | 0.21(0.00,3.14) | -1.69(-1.80,-1.59) |
| Antigua and Barbuda | 0.22(0.18,0.27) | 3.22(0.00,61.62) | 0.38(0.31,0.47) | 2.87(0.00,34.57) | -0.69(-1.01,-0.37) |
| Argentina | 88.91(73.94,106.10) | 2.13(1.39,3.08) | 162.08(130.77,198.22) | 2.25(1.52,3.17) | 1.00(0.59,1.41) |
| Armenia | 0.10(0.09,0.12) | 0.03(0.00,1.17) | 4.28(3.54,5.06) | 0.71(0.14,1.95) | 8.99(6.93,11.09) |
| Australia | 170.06(142.46,198.87) | 6.61(4.66,8.88) | 231.44(183.25,282.64) | 3.95(2.69,5.42) | -1.75(-1.98,-1.52) |
| Austria | 68.81(56.32,83.21) | 4.42(2.73,6.62) | 45.72(35.50,56.43) | 2.00(1.09,3.21) | -2.36(-2.72,-2.00) |
| Azerbaijan | 0.10(0.06,0.16) | 0.02(0.00,0.70) | 0.69(0.19,1.08) | 0.06(0.00,0.46) | 3.80(2.50,5.11) |
| Bahamas | 0.51(0.39,0.64) | 2.89(0.00,28.13) | 1.31(0.99,1.70) | 2.66(0.05,13.68) | -0.53(-0.82,-0.24) |
| Bahrain | 0.04(0.02,0.07) | 0.20(0.00,21.54) | 1.04(0.59,1.67) | 1.05(0.00,6.75) | 7.50(6.13,8.88) |
| Bangladesh | 314.53(169.26,536.79) | 5.90(2.71,10.96) | 696.32(370.86,1202.07) | 4.12(1.98,7.52) | -1.29(-1.43,-1.15) |
| Barbados | 2.09(1.67,2.61) | 5.47(0.39,21.36) | 3.98(2.97,5.18) | 5.72(0.87,17.12) | -0.14(-0.57,0.29) |
| Belarus | 1.56(1.30,1.84) | 0.09(0.00,0.41) | 2.45(1.83,3.17) | 0.11(0.01,0.41) | -0.29(-0.63,0.05) |
| Belgium | 71.87(58.58,85.98) | 3.53(2.19,5.21) | 68.21(52.11,84.54) | 2.29(1.31,3.51) | -1.78(-2.06,-1.49) |
| Belize | 0.25(0.19,0.32) | 2.18(0.00,38.09) | 0.89(0.69,1.12) | 2.59(0.01,16.76) | 0.14(-0.40,0.68) |
| Benin | 0.15(0.05,0.31) | 0.06(0.00,1.84) | 0.18(0.07,0.36) | 0.03(0.00,0.78) | -2.44(-2.65,-2.22) |
| Bermuda | 0.22(0.17,0.27) | 2.76(0.00,54.31) | 0.54(0.39,0.72) | 2.98(0.00,28.05) | -0.24(-0.65,0.16) |
| Bhutan | 1.50(0.74,2.48) | 5.71(0.02,30.28) | 4.20(2.05,7.22) | 5.80(0.35,20.31) | 0.12(0.10,0.15) |
| Bolivia (Plurinational State of) | 22.53(14.35,34.10) | 6.06(2.13,12.80) | 59.61(35.07,93.76) | 5.36(2.20,10.31) | -0.26(-0.33,-0.20) |
| Bosnia and Herzegovina | 9.66(6.06,14.20) | 1.99(0.46,4.88) | 12.20(7.47,18.82) | 1.44(0.37,3.48) | -1.02(-1.17,-0.87) |
| Botswana | 1.92(0.92,3.10) | 2.94(0.03,13.64) | 3.49(1.03,5.88) | 2.11(0.02,7.78) | -0.93(-1.25,-0.61) |
| Brazil | 152.57(138.06,166.09) | 1.45(1.10,1.84) | 459.01(398.91,510.63) | 1.45(1.14,1.76) | 0.29(0.07,0.50) |
| Brunei Darussalam | 0.84(0.52,1.27) | 7.84(0.01,56.34) | 1.80(1.07,2.83) | 4.50(0.08,21.23) | -1.62(-1.75,-1.50) |
| Bulgaria | 12.36(10.41,14.64) | 0.73(0.30,1.44) | 9.24(7.19,11.71) | 0.48(0.15,1.07) | -2.10(-2.53,-1.67) |
| Burkina Faso | 0.42(0.13,0.94) | 0.08(0.00,1.07) | 0.44(0.15,0.91) | 0.04(0.00,0.52) | -2.36(-2.58,-2.14) |
| Burundi | 0.72(0.20,5.77) | 0.27(0.00,4.78) | 0.70(0.17,6.60) | 0.13(0.00,2.58) | -2.74(-2.91,-2.57) |
| Cabo Verde | 0.02(0.00,0.03) | 0.05(0.00,12.78) | 0.00(0.00,0.01) | 0.01(0.00,6.93) | -4.79(-5.16,-4.42) |
| Cambodia | 9.66(4.95,14.91) | 1.91(0.31,4.86) | 27.70(13.54,43.05) | 1.89(0.50,3.95) | 0.01(-0.05,0.06) |
| Cameroon | 0.32(0.10,0.63) | 0.06(0.00,0.98) | 0.41(0.17,0.90) | 0.03(0.00,0.39) | -2.54(-2.69,-2.39) |
| Canada | 190.90(157.70,226.23) | 4.51(3.17,6.09) | 282.08(221.84,344.87) | 2.93(2.01,3.99) | -1.78(-2.23,-1.33) |
| Central African Republic | 0.50(0.14,4.36) | 0.41(0.00,8.75) | 0.58(0.13,5.74) | 0.26(0.00,5.71) | -1.52(-1.62,-1.42) |
| Chad | 0.23(0.08,0.49) | 0.07(0.00,1.40) | 0.25(0.08,0.55) | 0.04(0.00,0.76) | -2.09(-2.35,-1.83) |
| Chile | 67.53(55.90,79.82) | 5.51(3.45,8.11) | 127.91(102.37,156.13) | 3.85(2.51,5.50) | -0.66(-1.05,-0.26) |
| China | 3393.01(2695.71,4297.10) | 3.39(2.59,4.42) | 8900.18(6127.97,11093.58) | 3.31(2.22,4.20) | 0.25(-0.16,0.66) |
| Colombia | 94.47(80.26,109.69) | 4.66(3.14,6.52) | 234.43(178.43,297.63) | 3.36(2.20,4.78) | -0.78(-1.35,-0.22) |
| Comoros | 0.05(0.01,0.36) | 0.21(0.00,19.57) | 0.08(0.02,0.81) | 0.15(0.00,9.39) | -1.25(-1.39,-1.10) |
| Congo | 0.53(0.16,4.82) | 0.43(0.00,9.30) | 0.64(0.17,6.59) | 0.23(0.00,4.94) | -2.41(-2.59,-2.23) |
| Cook Islands | 0.00(0.00,0.00) | 0.00(0.00,251.31) | 0.00(0.00,0.00) | 0.00(0.00,108.37) | -1.80(-2.06,-1.54) |
| Costa Rica | 10.51(8.67,12.48) | 5.03(1.87,10.34) | 35.80(27.53,44.02) | 5.11(2.60,8.44) | -0.04(-0.20,0.12) |
| Côte d'Ivoire | 0.16(0.06,0.35) | 0.04(0.00,1.05) | 0.32(0.12,0.65) | 0.03(0.00,0.42) | -1.26(-1.54,-0.97) |
| Croatia | 20.94(17.75,24.35) | 2.65(1.33,4.57) | 26.39(21.06,32.33) | 2.21(1.09,3.82) | -0.43(-0.96,0.10) |
| Cuba | 34.18(27.70,40.86) | 2.67(1.43,4.33) | 81.02(63.58,100.82) | 3.33(2.01,5.04) | 0.32(-0.02,0.66) |
| Cyprus | 11.51(6.77,18.49) | 10.73(2.49,27.09) | 12.51(7.62,18.94) | 4.59(1.18,10.85) | -3.02(-3.17,-2.86) |
| Czechia | 28.05(23.79,32.77) | 1.53(0.83,2.51) | 29.57(23.81,36.53) | 1.05(0.54,1.79) | -1.31(-2.40,-0.20) |
| Democratic People's Republic of Korea | 70.71(43.10,111.06) | 3.90(1.72,7.37) | 179.63(111.46,278.01) | 4.51(2.30,7.85) | 0.91(0.72,1.11) |
| Democratic Republic of the Congo | 5.69(1.47,50.45) | 0.32(0.01,3.78) | 9.21(2.00,94.27) | 0.24(0.01,2.95) | -1.12(-1.29,-0.94) |
| Denmark | 50.02(40.92,60.21) | 4.76(2.79,7.37) | 69.97(55.34,85.66) | 4.58(2.73,6.93) | -0.71(-1.21,-0.21) |
| Djibouti | 0.02(0.01,0.18) | 0.17(0.00,29.84) | 0.08(0.02,0.67) | 0.12(0.00,7.46) | -1.45(-1.63,-1.26) |
| Dominica | 0.27(0.17,0.40) | 3.49(0.00,58.10) | 0.33(0.20,0.50) | 3.05(0.00,43.31) | -0.56(-0.86,-0.26) |
| Dominican Republic | 15.55(8.36,23.25) | 3.59(0.85,8.03) | 40.61(23.04,63.99) | 3.35(1.21,6.74) | 0.21(-0.14,0.56) |
| Ecuador | 42.08(36.06,48.89) | 6.83(4.10,10.50) | 97.67(75.40,123.75) | 4.83(2.93,7.29) | -1.01(-1.43,-0.58) |
| Egypt | 15.49(8.49,24.24) | 0.51(0.13,1.20) | 24.75(15.18,37.40) | 0.34(0.12,0.70) | -1.06(-1.19,-0.94) |
| El Salvador | 6.12(3.64,9.75) | 1.73(0.26,5.10) | 12.78(7.69,20.07) | 1.67(0.42,4.04) | 0.02(-0.12,0.16) |
| Equatorial Guinea | 0.09(0.02,0.82) | 0.42(0.00,24.56) | 0.11(0.03,1.08) | 0.20(0.00,10.86) | -2.55(-2.71,-2.39) |
| Eritrea | 0.21(0.06,1.73) | 0.20(0.00,6.43) | 0.45(0.11,4.50) | 0.16(0.00,3.88) | -0.88(-0.92,-0.83) |
| Estonia | 10.90(8.90,13.03) | 4.08(1.51,8.33) | 14.48(11.54,17.84) | 4.09(1.66,7.98) | -0.78(-1.49,-0.06) |
| Eswatini | 1.38(0.49,2.22) | 4.46(0.00,24.42) | 2.23(0.52,4.13) | 3.56(0.00,16.64) | -0.83(-1.17,-0.49) |
| Ethiopia | 5.86(1.94,40.63) | 0.27(0.01,2.51) | 6.07(1.93,53.75) | 0.13(0.00,1.46) | -2.94(-3.14,-2.74) |
| Fiji | 0.00(0.00,0.00) | 0.00(0.00,9.99) | 0.00(0.00,0.00) | 0.00(0.00,4.02) | -4.48(-5.00,-3.95) |
| Finland | 125.51(104.69,147.88) | 13.43(9.16,18.58) | 104.77(82.87,126.33) | 6.37(4.01,9.14) | -3.08(-3.33,-2.82) |
| France | 456.44(371.81,552.37) | 4.21(3.09,5.54) | 414.14(313.63,515.50) | 2.33(1.57,3.16) | -1.81(-2.10,-1.52) |
| Gabon | 0.30(0.08,2.65) | 0.43(0.00,11.82) | 0.26(0.07,2.67) | 0.22(0.00,6.90) | -2.31(-2.61,-2.02) |
| Gambia | 0.02(0.01,0.05) | 0.06(0.00,9.78) | 0.04(0.01,0.08) | 0.04(0.00,3.56) | -1.08(-1.25,-0.92) |
| Georgia | 0.15(0.12,0.19) | 0.02(0.00,0.50) | 2.68(2.16,3.20) | 0.33(0.04,1.11) | 8.22(5.88,10.61) |
| Germany | 648.74(520.46,787.03) | 3.98(2.93,5.18) | 546.90(413.52,682.16) | 2.22(1.52,2.99) | -1.66(-2.58,-0.73) |
| Ghana | 0.24(0.03,0.54) | 0.04(0.00,0.69) | 5.41(1.68,11.90) | 0.29(0.01,1.11) | 10.83(8.27,13.45) |
| Greece | 23.02(19.17,27.25) | 1.15(0.58,1.98) | 44.10(34.78,53.90) | 1.48(0.81,2.36) | 0.97(0.18,1.77) |
| Greenland | 0.10(0.06,0.16) | 2.73(0.00,112.15) | 0.18(0.10,0.33) | 2.03(0.00,49.02) | -0.30(-0.53,-0.07) |
| Grenada | 0.16(0.12,0.22) | 1.71(0.00,43.66) | 0.18(0.13,0.24) | 1.30(0.00,30.01) | -0.46(-0.76,-0.16) |
| Guam | 0.00(0.00,0.00) | 0.01(0.00,41.95) | 0.00(0.00,0.00) | 0.00(0.00,13.33) | -5.86(-8.59,-3.06) |
| Guatemala | 11.68(9.96,13.54) | 2.94(1.20,5.78) | 48.59(39.30,59.22) | 3.64(2.10,5.72) | 1.26(0.81,1.72) |
| Guinea | 0.22(0.08,0.46) | 0.05(0.00,1.13) | 0.23(0.09,0.49) | 0.04(0.00,0.72) | -1.68(-2.14,-1.21) |
| Guinea-Bissau | 0.03(0.01,0.07) | 0.07(0.00,8.59) | 0.03(0.01,0.06) | 0.04(0.00,5.02) | -2.65(-2.86,-2.44) |
| Guyana | 0.05(0.04,0.07) | 0.12(0.00,8.73) | 0.77(0.55,1.02) | 0.98(0.00,7.18) | 5.12(2.54,7.77) |
| Haiti | 12.16(6.03,22.39) | 3.25(0.59,9.04) | 22.18(10.15,43.84) | 2.76(0.61,7.33) | -0.39(-0.48,-0.30) |
| Honduras | 17.07(9.01,30.41) | 7.26(1.75,18.41) | 69.53(41.33,106.53) | 9.24(3.95,17.12) | 0.78(0.57,0.98) |
| Hungary | 76.71(64.15,90.80) | 3.90(2.51,5.67) | 56.52(43.29,70.29) | 2.16(1.20,3.40) | -2.45(-2.99,-1.91) |
| Iceland | 1.35(1.09,1.64) | 3.67(0.10,17.98) | 1.51(1.15,1.91) | 1.99(0.06,9.33) | -1.55(-1.76,-1.34) |
| India | 2635.20(1748.05,3786.20) | 5.11(3.23,7.57) | 7253.29(5145.42,10786.31) | 5.00(3.45,7.57) | 0.12(-0.02,0.26) |
| Indonesia | 63.79(26.35,97.06) | 0.59(0.16,1.09) | 209.83(79.37,304.72) | 0.74(0.22,1.21) | 0.93(0.64,1.22) |
| Iran (Islamic Republic of) | 7.56(4.56,11.45) | 0.24(0.04,0.65) | 28.88(19.69,39.48) | 0.31(0.13,0.58) | 1.73(1.40,2.06) |
| Iraq | 2.13(0.99,3.91) | 0.24(0.00,1.12) | 3.97(1.98,7.12) | 0.15(0.01,0.55) | -1.81(-1.94,-1.68) |
| Ireland | 56.88(48.69,66.60) | 10.58(6.69,15.74) | 53.35(42.13,64.70) | 5.25(2.99,8.12) | -2.10(-2.36,-1.83) |
| Israel | 10.77(8.72,13.17) | 1.69(0.62,3.53) | 21.16(16.15,26.35) | 1.33(0.58,2.43) | -1.38(-1.74,-1.01) |
| Italy | 348.85(309.75,378.43) | 2.95(2.34,3.54) | 572.13(461.47,647.82) | 3.17(2.33,3.88) | -0.07(-0.29,0.15) |
| Jamaica | 4.72(3.58,5.97) | 2.05(0.38,5.66) | 9.99(7.00,13.45) | 2.55(0.72,5.83) | 0.73(0.42,1.05) |
| Japan | 1370.66(1232.49,1451.54) | 6.28(5.34,7.00) | 2157.91(1708.62,2434.85) | 4.87(3.68,5.72) | -1.36(-2.00,-0.73) |
| Jordan | 0.35(0.17,0.65) | 0.25(0.00,3.53) | 1.91(1.01,3.36) | 0.23(0.00,1.15) | 0.81(0.34,1.28) |
| Kazakhstan | 0.61(0.46,0.81) | 0.04(0.00,0.34) | 21.56(16.53,27.70) | 0.97(0.43,1.81) | 8.20(4.69,11.84) |
| Kenya | 1.63(0.57,12.82) | 0.17(0.00,2.35) | 3.32(1.09,30.06) | 0.13(0.00,1.66) | -0.85(-0.95,-0.75) |
| Kiribati | 0.00(0.00,0.00) | 0.00(0.00,89.58) | 0.00(0.00,0.00) | 0.00(0.00,45.05) | -1.28(-1.41,-1.14) |
| Kuwait | 0.13(0.09,0.16) | 0.22(0.00,6.88) | 2.49(1.78,3.34) | 0.87(0.06,3.25) | 3.79(2.47,5.14) |
| Kyrgyzstan | 4.83(3.52,6.92) | 1.31(0.23,3.87) | 13.28(9.58,18.64) | 2.34(0.80,5.16) | 4.34(3.02,5.67) |
| Lao People's Democratic Republic | 4.46(2.21,7.15) | 1.87(0.13,6.12) | 8.34(3.98,13.19) | 1.60(0.21,4.30) | -0.59(-0.77,-0.40) |
| Latvia | 14.02(11.37,16.85) | 3.00(1.23,5.78) | 17.21(13.70,21.50) | 3.28(1.41,6.23) | -0.32(-0.98,0.35) |
| Lebanon | 1.49(0.78,2.62) | 0.56(0.00,3.10) | 2.40(1.32,3.97) | 0.33(0.01,1.39) | -1.26(-1.47,-1.04) |
| Lesotho | 3.19(1.45,5.15) | 3.18(0.10,11.84) | 4.55(1.32,7.57) | 3.55(0.06,11.86) | 0.57(0.16,0.98) |
| Liberia | 0.09(0.03,0.19) | 0.06(0.00,2.91) | 0.07(0.03,0.16) | 0.03(0.00,1.86) | -2.52(-2.72,-2.32) |
| Libya | 0.65(0.31,1.22) | 0.30(0.00,2.75) | 4.04(1.80,7.37) | 0.73(0.03,2.71) | 3.99(3.68,4.30) |
| Lithuania | 21.27(17.46,25.41) | 3.64(1.76,6.41) | 34.64(27.69,42.40) | 4.56(2.42,7.54) | 0.06(-0.53,0.66) |
| Luxembourg | 2.29(1.88,2.75) | 3.21(0.29,11.77) | 2.19(1.69,2.73) | 1.65(0.12,6.28) | -1.73(-1.98,-1.47) |
| Madagascar | 1.04(0.28,8.14) | 0.18(0.00,2.73) | 1.14(0.27,10.77) | 0.10(0.00,1.64) | -2.12(-2.20,-2.03) |
| Malawi | 0.99(0.29,8.22) | 0.22(0.00,3.62) | 1.35(0.35,13.90) | 0.16(0.00,2.84) | -1.43(-1.73,-1.13) |
| Malaysia | 5.80(3.00,10.46) | 0.55(0.06,1.81) | 12.54(6.74,21.83) | 0.36(0.08,0.94) | -1.69(-1.94,-1.44) |
| Maldives | 0.16(0.10,0.25) | 1.59(0.00,42.81) | 0.40(0.25,0.60) | 1.13(0.00,13.61) | -1.00(-1.19,-0.81) |
| Mali | 0.30(0.10,0.66) | 0.07(0.00,1.09) | 0.42(0.13,0.87) | 0.04(0.00,0.54) | -1.16(-1.43,-0.88) |
| Malta | 1.64(1.34,1.96) | 2.99(0.14,13.07) | 2.34(1.79,2.92) | 1.79(0.14,6.62) | -2.10(-2.40,-1.81) |
| Marshall Islands | 0.00(0.00,0.00) | 0.01(0.00,200.50) | 0.00(0.00,0.00) | 0.00(0.00,95.60) | -3.61(-3.95,-3.26) |
| Mauritania | 0.10(0.03,0.22) | 0.09(0.00,3.58) | 0.09(0.03,0.20) | 0.04(0.00,1.61) | -3.32(-3.58,-3.06) |
| Mauritius | 0.09(0.07,0.12) | 0.11(0.00,4.43) | 4.78(3.78,5.92) | 1.95(0.40,5.28) | 6.00(3.26,8.81) |
| Mexico | 606.41(564.99,640.40) | 12.32(10.55,14.05) | 1186.00(1016.37,1350.12) | 7.65(6.16,9.19) | -1.38(-1.58,-1.18) |
| Micronesia (Federated States of) | 0.00(0.00,0.00) | 0.01(0.00,62.60) | 0.00(0.00,0.00) | 0.00(0.00,41.99) | -3.86(-4.23,-3.48) |
| Monaco | 0.02(0.01,0.03) | 0.21(0.00,40.65) | 0.02(0.01,0.04) | 0.17(0.00,30.16) | -0.66(-0.81,-0.50) |
| Mongolia | 1.23(0.64,3.61) | 1.02(0.00,8.01) | 4.48(2.54,7.09) | 1.80(0.17,5.85) | 2.40(1.90,2.91) |
| Montenegro | 0.76(0.51,1.06) | 0.98(0.00,7.35) | 1.37(0.88,1.95) | 1.03(0.01,5.36) | 0.23(0.13,0.32) |
| Morocco | 7.30(2.62,18.38) | 0.43(0.03,1.70) | 32.22(11.55,72.30) | 0.76(0.14,2.15) | 2.59(2.18,2.99) |
| Mozambique | 1.53(0.40,12.76) | 0.23(0.00,3.27) | 2.09(0.50,20.52) | 0.17(0.00,2.60) | -0.76(-0.88,-0.63) |
| Myanmar | 44.61(21.85,69.70) | 1.64(0.50,3.24) | 89.75(45.09,140.47) | 1.53(0.56,2.82) | -0.28(-0.54,-0.01) |
| Namibia | 2.33(1.13,3.89) | 2.99(0.05,12.95) | 4.02(1.18,7.42) | 2.58(0.03,9.61) | -0.79(-1.06,-0.52) |
| Nauru | 0.00(0.00,0.00) | 0.01(0.00,739.93) | 0.00(0.00,0.00) | 0.00(0.00,580.51) | -2.39(-2.85,-1.93) |
| Nepal | 52.28(27.21,90.04) | 4.98(1.71,10.54) | 145.27(77.14,250.96) | 5.10(2.14,9.97) | 0.16(-0.10,0.43) |
| Netherlands | 254.02(210.71,298.01) | 9.84(7.10,12.93) | 222.99(174.56,269.77) | 4.85(3.26,6.62) | -2.27(-2.96,-1.58) |
| New Zealand | 38.70(32.40,45.51) | 7.50(4.30,11.78) | 44.80(35.02,54.95) | 4.05(2.21,6.47) | -2.12(-2.68,-1.56) |
| Nicaragua | 6.93(4.36,10.44) | 3.97(0.73,10.86) | 19.53(12.76,28.89) | 3.37(1.17,7.17) | -0.29(-0.62,0.03) |
| Niger | 0.18(0.06,0.40) | 0.06(0.00,1.46) | 0.35(0.11,0.76) | 0.04(0.00,0.55) | -1.94(-2.28,-1.59) |
| Nigeria | 2.71(1.24,4.86) | 0.05(0.00,0.22) | 2.61(1.29,5.36) | 0.03(0.00,0.12) | -2.36(-2.58,-2.14) |
| Niue | 0.00(0.00,0.00) | 0.01(0.00,1323.70) | 0.00(0.00,0.00) | 0.00(0.00,1310.34) | -3.33(-3.43,-3.24) |
| North Macedonia | 1.16(0.70,1.77) | 0.50(0.00,2.98) | 1.22(0.69,2.03) | 0.28(0.00,1.64) | -2.16(-2.30,-2.03) |
| Northern Mariana Islands | 0.00(0.00,0.00) | 0.01(0.00,252.00) | 0.00(0.00,0.00) | 0.02(0.00,60.15) | 1.87(-0.06,3.83) |
| Norway | 101.70(90.46,110.54) | 11.33(8.11,14.84) | 51.83(42.84,59.02) | 4.04(2.42,5.94) | -3.99(-4.41,-3.57) |
| Oman | 0.17(0.09,0.33) | 0.24(0.00,6.14) | 0.48(0.24,0.86) | 0.25(0.00,2.71) | 1.02(0.60,1.45) |
| Pakistan | 445.86(258.51,684.10) | 6.78(3.47,11.21) | 930.49(533.59,1437.95) | 6.92(3.64,11.27) | -0.23(-0.50,0.05) |
| Palau | 0.00(0.00,0.00) | 0.03(0.00,309.90) | 0.00(0.00,0.00) | 0.02(0.00,133.80) | -1.57(-1.65,-1.49) |
| Palestine | 0.58(0.31,0.94) | 0.56(0.00,5.28) | 0.97(0.55,1.55) | 0.35(0.00,2.32) | -1.46(-1.59,-1.33) |
| Panama | 5.09(4.17,6.09) | 2.88(0.66,7.45) | 16.48(11.94,20.96) | 3.01(1.12,5.85) | -0.10(-0.30,0.09) |
| Papua New Guinea | 0.01(0.00,0.05) | 0.01(0.00,1.90) | 0.02(0.01,0.05) | 0.00(0.00,0.73) | -2.71(-2.96,-2.46) |
| Paraguay | 5.08(3.33,7.66) | 1.93(0.29,5.80) | 25.14(14.93,37.91) | 3.53(1.17,7.31) | 2.60(2.26,2.93) |
| Peru | 69.90(41.80,99.06) | 5.04(2.17,8.69) | 129.91(81.49,194.57) | 3.19(1.59,5.50) | -2.04(-2.48,-1.61) |
| Philippines | 69.24(39.12,88.73) | 2.11(0.85,3.33) | 174.58(113.28,214.83) | 1.81(0.97,2.54) | -0.69(-0.95,-0.43) |
| Poland | 378.11(353.72,398.66) | 6.60(5.55,7.68) | 243.30(214.37,269.36) | 2.48(1.90,3.10) | -3.55(-3.91,-3.19) |
| Portugal | 64.81(54.30,75.95) | 3.47(2.19,5.09) | 87.13(67.99,106.78) | 2.79(1.69,4.13) | -0.55(-1.04,-0.06) |
| Puerto Rico | 17.84(14.90,20.83) | 3.85(1.79,6.87) | 28.47(21.46,35.97) | 3.02(1.42,5.29) | -0.91(-1.12,-0.70) |
| Qatar | 0.02(0.01,0.04) | 0.26(0.00,43.91) | 0.13(0.07,0.24) | 0.17(0.00,5.29) | -0.76(-1.42,-0.08) |
| Republic of Korea | 142.47(90.25,226.22) | 4.27(2.17,7.72) | 185.13(112.54,300.76) | 1.47(0.74,2.68) | -3.87(-4.10,-3.64) |
| Republic of Moldova | 4.07(3.32,4.94) | 0.73(0.14,2.06) | 9.02(7.22,11.07) | 1.12(0.37,2.45) | 0.06(-0.55,0.67) |
| Romania | 0.64(0.47,0.85) | 0.02(0.00,0.15) | 0.78(0.59,1.01) | 0.02(0.00,0.11) | -0.74(-0.92,-0.55) |
| Russian Federation | 505.01(482.61,524.48) | 2.14(1.87,2.42) | 877.85(784.43,966.95) | 2.66(2.21,3.12) | 0.06(-0.77,0.89) |
| Rwanda | 0.90(0.27,7.32) | 0.28(0.00,4.58) | 1.01(0.25,10.12) | 0.14(0.00,2.55) | -3.09(-3.43,-2.75) |
| Saint Kitts and Nevis | 0.24(0.18,0.30) | 4.49(0.00,81.36) | 0.36(0.26,0.47) | 4.05(0.00,51.61) | -0.15(-0.90,0.61) |
| Saint Lucia | 0.44(0.36,0.52) | 3.86(0.00,41.75) | 1.31(1.02,1.63) | 4.36(0.09,21.99) | -0.23(-0.62,0.15) |
| Saint Vincent and the Grenadines | 0.17(0.14,0.21) | 1.87(0.00,44.47) | 0.24(0.17,0.31) | 1.27(0.00,23.22) | -0.81(-1.38,-0.24) |
| Samoa | 0.00(0.00,0.00) | 0.01(0.00,36.34) | 0.00(0.00,0.00) | 0.00(0.00,21.69) | -2.84(-3.09,-2.59) |
| San Marino | 0.06(0.04,0.10) | 1.34(0.00,84.82) | 0.06(0.03,0.11) | 0.67(0.00,42.62) | -1.47(-1.80,-1.13) |
| Sao Tome and Principe | 0.00(0.00,0.01) | 0.05(0.00,45.73) | 0.00(0.00,0.01) | 0.03(0.00,30.07) | -1.68(-2.06,-1.30) |
| Saudi Arabia | 2.08(0.90,4.15) | 0.34(0.00,1.69) | 5.86(3.10,10.36) | 0.33(0.04,1.06) | 0.04(-0.31,0.39) |
| Senegal | 0.23(0.08,0.48) | 0.06(0.00,1.23) | 0.32(0.11,0.70) | 0.04(0.00,0.56) | -1.84(-1.97,-1.71) |
| Serbia | 26.64(16.60,40.92) | 1.79(0.64,3.73) | 31.48(19.24,48.68) | 1.40(0.52,2.87) | -0.87(-1.01,-0.72) |
| Seychelles | 0.02(0.01,0.03) | 0.27(0.00,51.82) | 0.03(0.02,0.04) | 0.19(0.00,26.45) | -0.71(-1.03,-0.38) |
| Sierra Leone | 0.14(0.05,0.28) | 0.06(0.00,1.75) | 0.13(0.05,0.29) | 0.03(0.00,1.02) | -2.34(-2.66,-2.03) |
| Singapore | 2.91(2.30,3.60) | 1.14(0.13,3.78) | 3.62(2.78,4.61) | 0.32(0.05,0.99) | -4.80(-5.34,-4.26) |
| Slovakia | 12.19(7.96,17.23) | 1.55(0.44,3.49) | 12.62(7.72,19.36) | 0.98(0.25,2.34) | -1.70(-1.78,-1.62) |
| Slovenia | 16.27(13.40,19.45) | 5.16(2.29,9.58) | 15.92(12.13,19.87) | 2.74(1.08,5.29) | -2.27(-2.69,-1.85) |
| Solomon Islands | 0.00(0.00,0.00) | 0.01(0.00,24.69) | 0.00(0.00,0.00) | 0.00(0.00,10.26) | -1.92(-2.15,-1.70) |
| Somalia | 0.75(0.21,5.12) | 0.34(0.00,5.30) | 1.42(0.37,11.83) | 0.22(0.00,3.22) | -1.49(-1.55,-1.44) |
| South Africa | 120.95(82.10,160.85) | 4.97(2.68,7.72) | 216.27(169.64,280.33) | 3.87(2.59,5.63) | -1.46(-2.04,-0.88) |
| South Sudan | 0.65(0.20,4.47) | 0.21(0.00,3.59) | 0.59(0.17,5.07) | 0.15(0.00,2.96) | -1.57(-1.74,-1.41) |
| Spain | 216.11(174.42,264.49) | 2.96(2.05,4.09) | 252.43(190.10,315.41) | 2.08(1.35,2.90) | -1.09(-1.28,-0.89) |
| Sri Lanka | 2.74(1.78,4.12) | 0.22(0.01,0.84) | 7.10(3.83,11.47) | 0.20(0.03,0.57) | 0.23(-0.26,0.71) |
| Sudan | 4.74(2.04,10.33) | 0.44(0.02,1.75) | 12.33(5.94,22.79) | 0.58(0.10,1.62) | 1.26(1.03,1.50) |
| Suriname | 0.32(0.18,0.55) | 1.07(0.00,15.79) | 0.62(0.32,1.08) | 0.79(0.00,7.30) | -0.64(-0.84,-0.44) |
| Sweden | 136.77(115.27,157.93) | 6.98(4.86,9.42) | 110.94(86.08,135.68) | 4.10(2.55,5.93) | -2.07(-2.47,-1.67) |
| Switzerland | 68.44(53.82,83.90) | 5.17(3.05,7.84) | 57.86(42.23,73.12) | 2.55(1.35,4.06) | -2.37(-2.68,-2.07) |
| Syrian Arab Republic | 2.02(1.12,3.25) | 0.34(0.01,1.53) | 7.13(4.16,10.65) | 0.45(0.07,1.21) | 1.39(1.14,1.65) |
| Taiwan (Province of China) | 65.87(57.30,75.21) | 3.28(2.16,4.70) | 140.00(110.30,173.35) | 2.45(1.59,3.53) | 0.56(0.00,1.11) |
| Tajikistan | 0.56(0.28,1.31) | 0.17(0.00,1.88) | 3.09(1.20,5.36) | 0.47(0.01,1.85) | 4.16(3.14,5.18) |
| Thailand | 65.06(35.91,113.12) | 1.63(0.63,3.40) | 191.45(109.69,319.24) | 1.34(0.63,2.50) | -0.91(-1.08,-0.74) |
| Timor-Leste | 0.35(0.18,0.56) | 1.27(0.00,17.57) | 1.55(0.71,2.50) | 1.44(0.00,7.44) | 0.58(0.38,0.79) |
| Togo | 0.06(0.02,0.12) | 0.05(0.00,2.96) | 0.12(0.04,0.26) | 0.03(0.00,1.03) | -1.88(-2.08,-1.69) |
| Tokelau | 0.00(0.00,0.00) | 0.01(0.00,1933.06) | 0.00(0.00,0.00) | 0.00(0.00,1912.00) | -3.46(-3.78,-3.14) |
| Tonga | 0.00(0.00,0.00) | 0.01(0.00,55.78) | 0.00(0.00,0.00) | 0.00(0.00,38.32) | -2.25(-2.54,-1.97) |
| Trinidad and Tobago | 5.82(4.75,6.93) | 5.62(1.43,13.83) | 11.32(8.37,14.59) | 4.38(1.43,9.38) | -1.33(-1.58,-1.08) |
| Tunisia | 1.34(0.66,2.41) | 0.22(0.00,1.29) | 6.34(2.77,11.86) | 0.37(0.03,1.23) | 2.25(1.85,2.65) |
| Turkey | 109.31(62.67,178.99) | 2.69(1.18,5.10) | 193.47(117.82,295.36) | 1.64(0.83,2.80) | -1.31(-1.54,-1.08) |
| Turkmenistan | 0.01(0.01,0.02) | 0.01(0.00,1.66) | 2.14(1.53,3.11) | 0.45(0.02,1.89) | 10.97(9.21,12.75) |
| Tuvalu | 0.00(0.00,0.00) | 0.01(0.00,440.62) | 0.00(0.00,0.00) | 0.00(0.00,279.64) | -3.39(-3.62,-3.16) |
| Uganda | 1.39(0.41,9.65) | 0.19(0.00,2.44) | 2.24(0.55,22.71) | 0.14(0.00,2.15) | -1.33(-1.54,-1.11) |
| Ukraine | 75.36(45.36,96.40) | 0.79(0.35,1.23) | 80.75(55.42,113.68) | 0.76(0.39,1.29) | -0.51(-0.84,-0.18) |
| United Arab Emirates | 0.35(0.19,0.63) | 1.03(0.00,14.33) | 1.67(0.96,2.63) | 0.49(0.01,2.42) | -0.90(-1.68,-0.12) |
| United Kingdom | 1435.99(1327.38,1502.15) | 12.05(10.54,13.26) | 939.21(818.30,1014.33) | 5.71(4.64,6.56) | -1.70(-2.20,-1.20) |
| United Republic of Tanzania | 2.66(0.76,21.21) | 0.21(0.00,2.56) | 3.80(0.88,38.80) | 0.14(0.00,1.89) | -1.61(-1.70,-1.53) |
| United States Virgin Islands | 0.13(0.08,0.21) | 1.35(0.00,42.12) | 0.23(0.12,0.39) | 0.90(0.00,17.72) | -1.60(-1.92,-1.28) |
| United States of America | 1539.33(1389.05,1635.68) | 3.67(3.14,4.09) | 2071.84(1763.42,2308.30) | 2.64(2.14,3.06) | -1.37(-2.05,-0.68) |
| Uruguay | 19.25(15.98,22.83) | 3.72(1.76,6.63) | 38.32(30.87,45.97) | 5.39(2.95,8.62) | 1.45(1.10,1.79) |
| Uzbekistan | 2.13(1.55,4.34) | 0.15(0.01,0.78) | 39.50(31.86,47.97) | 1.29(0.71,2.08) | 4.79(3.15,6.45) |
| Vanuatu | 0.00(0.00,0.00) | 0.01(0.00,53.24) | 0.00(0.00,0.00) | 0.00(0.00,19.13) | -2.33(-2.56,-2.09) |
| Venezuela (Bolivarian Republic of) | 68.30(57.28,80.01) | 6.09(3.87,8.88) | 196.76(141.28,263.19) | 5.16(3.12,7.79) | -1.01(-1.24,-0.78) |
| Viet Nam | 76.34(37.21,122.76) | 1.53(0.53,2.94) | 172.49(86.05,280.45) | 1.42(0.57,2.60) | 0.18(-0.15,0.51) |
| Yemen | 2.17(0.98,4.29) | 0.39(0.00,1.93) | 9.73(4.63,18.29) | 0.62(0.09,1.84) | 1.79(1.60,1.97) |
| Zambia | 0.69(0.20,5.37) | 0.22(0.00,3.94) | 0.97(0.27,8.90) | 0.14(0.00,2.35) | -1.99(-2.24,-1.75) |
| Zimbabwe | 10.87(5.23,16.49) | 2.31(0.37,5.64) | 19.04(8.03,29.38) | 2.46(0.45,5.44) | 0.39(-0.06,0.85) |

**Abbreviations: ASR, age-standardised rate; CI: confidence interval; UI: uncertainly interval; EAPC, estimated annual percentage change.**

**Table S5. DALYs and age-standardised rates of elderly-onset rheumatoid arthritis across 204 countries and territories in 1990 and 2021, and their estimated annual percentage changes from 1990 to 2021.**

| **Location** | **Number (95 % UI), 1990** | **ASRs (95 % UI),1990** | **Number (95 % UI), 2021** | **ASRs (95 % UI),2021** | **EAPCs (95% CI)** |
| --- | --- | --- | --- | --- | --- |
| Afghanistan | 290.17(174.22,474.43) | 34.39(17.70,61.53) | 435.30(258.71,709.11) | 54.20(28.41,95.04) | 1.74(1.62,1.86) |
| Albania | 179.50(129.55,243.76) | 74.05(44.64,114.01) | 449.97(313.28,619.64) | 75.10(46.66,111.89) | 0.27(0.11,0.43) |
| Algeria | 305.63(190.75,454.56) | 20.63(11.11,33.64) | 1312.68(856.17,1881.44) | 30.88(18.82,46.31) | 1.45(1.37,1.51) |
| American Samoa | 0.49(0.28,0.77) | 19.77(0.00,208.01) | 1.31(0.77,1.99) | 23.00(0.13,127.04) | 0.37(0.31,0.42) |
| Andorra | 4.42(2.96,6.23) | 59.67(8.12,180.80) | 11.38(7.48,16.32) | 58.93(16.14,136.54) | 0.06(0.00,0.12) |
| Angola | 167.86(102.52,390.10) | 41.22(20.54,105.78) | 621.21(385.37,1106.82) | 50.14(28.08,94.76) | 0.70(0.59,0.80) |
| Antigua and Barbuda | 6.01(4.65,7.69) | 87.29(20.81,222.85) | 13.30(10.10,17.18) | 99.37(36.37,205.01) | 0.16(-0.01,0.34) |
| Argentina | 3873.23(3018.77,4892.75) | 93.00(69.92,120.82) | 8190.34(6284.71,10556.15) | 113.94(85.28,149.69) | 0.91(0.73,1.08) |
| Armenia | 79.25(46.32,123.64) | 23.62(10.12,43.94) | 272.43(185.75,386.30) | 45.17(26.53,70.77) | 2.62(2.17,3.07) |
| Australia | 7251.96(5759.42,9048.79) | 281.71(217.99,358.83) | 14300.62(10684.21,18662.62) | 244.11(178.93,323.17) | -0.47(-0.53,-0.40) |
| Austria | 2944.01(2283.12,3687.03) | 189.04(140.65,244.51) | 3202.72(2342.52,4247.07) | 140.45(98.61,191.94) | -0.83(-0.91,-0.76) |
| Azerbaijan | 112.86(64.57,178.33) | 19.62(8.66,35.91) | 318.97(192.61,483.32) | 25.42(13.26,42.12) | 1.08(0.81,1.36) |
| Bahamas | 15.76(12.02,19.96) | 89.81(35.42,175.70) | 46.39(35.15,60.24) | 94.10(49.71,157.22) | 0.06(-0.07,0.19) |
| Bahrain | 8.11(5.26,11.49) | 45.66(9.95,114.32) | 95.96(69.01,130.53) | 96.81(54.17,156.32) | 2.80(2.52,3.08) |
| Bangladesh | 7123.79(4431.00,10866.34) | 133.63(80.69,207.71) | 19631.84(12944.94,28721.11) | 116.11(75.25,171.84) | -0.45(-0.54,-0.36) |
| Barbados | 55.59(43.62,69.65) | 145.37(82.75,230.27) | 118.17(89.95,151.10) | 169.77(103.91,254.57) | 0.36(0.12,0.59) |
| Belarus | 485.54(295.36,743.58) | 28.51(15.42,46.91) | 989.90(625.83,1445.51) | 44.98(26.25,69.16) | 1.36(1.20,1.52) |
| Belgium | 3309.43(2526.65,4251.83) | 162.48(119.26,215.12) | 4385.80(3192.01,5783.30) | 147.27(103.50,199.26) | -0.48(-0.57,-0.40) |
| Belize | 8.18(6.20,10.67) | 71.78(20.37,169.01) | 31.29(23.94,40.29) | 90.76(44.46,158.97) | 0.56(0.34,0.79) |
| Benin | 45.60(27.58,71.21) | 19.44(7.79,38.28) | 134.33(78.59,203.75) | 23.70(10.97,41.24) | 0.66(0.60,0.72) |
| Bermuda | 7.10(5.35,9.27) | 90.92(23.36,223.36) | 20.15(14.75,26.80) | 111.32(45.35,215.76) | 0.55(0.45,0.64) |
| Bhutan | 42.90(27.02,61.51) | 162.81(67.59,299.53) | 137.21(90.56,194.64) | 189.40(100.59,309.19) | 0.51(0.47,0.56) |
| Bolivia (Plurinational State of) | 625.07(458.92,840.10) | 168.03(112.34,241.63) | 2281.38(1637.35,3012.01) | 205.00(140.09,280.49) | 0.66(0.62,0.70) |
| Bosnia and Herzegovina | 498.54(363.89,664.30) | 102.58(67.38,147.49) | 1017.91(731.34,1384.62) | 120.18(80.20,172.32) | 0.68(0.61,0.76) |
| Botswana | 69.57(46.39,96.15) | 106.40(52.02,179.55) | 168.77(111.64,232.82) | 101.82(55.44,159.72) | -0.11(-0.23,0.01) |
| Brazil | 7147.36(5706.88,8864.31) | 68.01(52.90,86.12) | 20984.43(16675.42,26155.36) | 66.43(51.99,83.81) | 0.12(0.03,0.21) |
| Brunei Darussalam | 24.46(17.28,33.40) | 228.21(94.39,436.62) | 77.61(54.92,106.25) | 193.74(103.26,320.72) | -0.45(-0.50,-0.40) |
| Bulgaria | 764.51(555.73,1040.14) | 45.39(30.31,65.63) | 922.42(635.94,1286.35) | 47.84(30.47,70.46) | -0.01(-0.24,0.23) |
| Burkina Faso | 100.27(57.85,153.55) | 19.65(8.61,35.25) | 237.07(139.72,369.76) | 22.72(11.26,39.24) | 0.53(0.46,0.60) |
| Burundi | 93.58(55.64,191.06) | 35.08(15.74,82.54) | 193.38(118.48,317.56) | 35.93(18.23,65.86) | 0.11(0.05,0.16) |
| Cabo Verde | 5.65(3.16,8.92) | 19.22(2.33,57.73) | 14.70(8.63,22.36) | 27.45(7.22,63.00) | 1.28(1.21,1.35) |
| Cambodia | 269.44(168.29,380.14) | 53.17(28.39,82.96) | 930.76(619.29,1284.43) | 63.47(38.97,92.51) | 0.54(0.51,0.58) |
| Cameroon | 113.85(66.08,176.22) | 22.57(10.13,40.49) | 370.26(217.33,564.75) | 26.98(13.80,44.69) | 0.54(0.39,0.70) |
| Canada | 8278.12(6542.44,10270.15) | 195.54(150.82,247.33) | 19064.90(14194.53,24932.44) | 198.34(145.25,262.62) | -0.14(-0.31,0.04) |
| Central African Republic | 56.28(34.92,140.60) | 45.81(19.79,135.00) | 97.27(59.79,200.89) | 43.69(20.48,103.61) | -0.13(-0.18,-0.07) |
| Chad | 57.45(32.91,89.09) | 17.22(6.78,32.85) | 116.70(68.85,179.17) | 18.51(8.49,32.89) | 0.25(0.13,0.38) |
| Chile | 2313.01(1860.47,2830.59) | 188.78(145.02,239.69) | 7437.29(5557.91,9560.77) | 223.97(163.00,293.74) | 0.73(0.57,0.90) |
| China | 148349.96(117765.03,188399.78) | 148.13(116.92,188.97) | 403327.42(309168.09,513645.16) | 149.93(114.52,191.46) | 0.20(0.03,0.37) |
| Colombia | 2810.03(2297.23,3388.68) | 138.57(108.69,172.82) | 9898.31(7584.54,12647.27) | 141.84(106.25,184.41) | 0.24(-0.03,0.51) |
| Comoros | 8.30(5.08,14.32) | 36.86(7.40,106.11) | 24.21(15.01,39.63) | 43.39(15.06,96.85) | 0.64(0.54,0.74) |
| Congo | 67.94(42.77,157.89) | 55.41(25.22,150.48) | 179.61(116.50,305.23) | 63.93(34.28,121.54) | 0.54(0.44,0.64) |
| Cook Islands | 0.21(0.12,0.34) | 14.41(0.00,297.75) | 0.66(0.38,1.02) | 19.27(0.00,164.46) | 0.98(0.91,1.05) |
| Costa Rica | 355.68(285.25,441.76) | 170.39(121.25,232.31) | 1580.57(1213.28,2006.02) | 225.66(163.61,299.21) | 0.79(0.72,0.87) |
| Côte d'Ivoire | 83.79(50.14,127.89) | 20.14(8.95,36.55) | 296.48(176.30,455.06) | 25.04(12.77,42.13) | 0.72(0.61,0.83) |
| Croatia | 978.75(763.32,1239.61) | 123.89(89.89,165.89) | 1817.59(1335.53,2411.75) | 152.34(106.01,210.36) | 0.60(0.45,0.75) |
| Cuba | 1178.62(904.14,1487.57) | 91.93(66.00,122.08) | 2901.36(2211.73,3757.95) | 119.36(87.23,159.62) | 0.67(0.53,0.80) |
| Cyprus | 360.46(259.30,488.15) | 336.18(213.29,497.51) | 746.43(533.31,996.68) | 273.74(179.33,388.93) | -0.60(-0.67,-0.53) |
| Czechia | 1292.39(994.09,1670.47) | 70.31(50.77,95.35) | 2122.30(1536.43,2846.15) | 75.45(51.93,104.97) | 0.29(0.03,0.55) |
| Democratic People's Republic of Korea | 2596.44(1878.95,3491.13) | 143.13(98.94,198.94) | 6841.78(4937.08,9160.70) | 171.84(120.56,234.84) | 0.77(0.67,0.87) |
| Democratic Republic of the Congo | 704.87(425.49,1683.57) | 40.11(21.97,100.50) | 1769.77(1103.63,3518.11) | 45.21(26.55,92.88) | 0.33(0.21,0.45) |
| Denmark | 2478.02(1916.06,3134.87) | 235.83(174.27,308.97) | 4230.24(3155.31,5464.33) | 277.13(199.56,367.59) | 0.35(0.20,0.50) |
| Djibouti | 4.74(2.87,8.18) | 34.88(4.15,117.82) | 28.39(17.94,44.29) | 42.49(15.90,88.89) | 0.65(0.52,0.78) |
| Dominica | 7.02(5.00,9.52) | 91.00(21.04,230.17) | 9.68(6.91,13.07) | 89.73(25.59,206.90) | -0.20(-0.40,-0.01) |
| Dominican Republic | 318.17(207.86,442.44) | 73.40(41.66,112.04) | 954.37(643.63,1343.25) | 78.76(49.09,116.95) | 0.46(0.27,0.64) |
| Ecuador | 1123.31(921.33,1349.39) | 182.38(140.08,231.09) | 4181.39(3193.11,5339.83) | 206.67(152.40,271.10) | 0.34(0.15,0.53) |
| Egypt | 847.21(571.67,1173.70) | 28.16(17.48,41.32) | 2645.75(1748.15,3690.14) | 35.99(22.68,51.84) | 0.77(0.68,0.86) |
| El Salvador | 208.91(146.41,288.72) | 58.95(34.89,91.43) | 575.52(400.72,794.55) | 74.99(47.23,110.98) | 0.84(0.80,0.87) |
| Equatorial Guinea | 9.14(5.47,23.89) | 42.68(8.84,166.22) | 36.59(24.07,58.64) | 69.62(29.36,144.05) | 1.92(1.78,2.06) |
| Eritrea | 38.00(22.53,73.35) | 35.94(13.43,87.18) | 117.66(71.83,199.64) | 41.60(19.86,81.08) | 0.45(0.42,0.48) |
| Estonia | 493.94(390.81,615.26) | 184.76(132.05,249.07) | 703.29(553.67,879.63) | 198.68(143.65,265.47) | 0.07(-0.17,0.31) |
| Eswatini | 44.42(27.05,61.53) | 143.22(57.50,254.53) | 85.43(48.76,125.63) | 136.26(57.48,238.63) | -0.22(-0.41,-0.04) |
| Ethiopia | 867.89(567.42,1566.01) | 39.46(23.72,74.82) | 1622.51(1057.12,2557.02) | 33.83(20.73,55.42) | -0.40(-0.48,-0.32) |
| Fiji | 4.47(2.53,7.00) | 12.09(1.15,39.02) | 12.93(7.24,20.64) | 14.05(3.22,34.41) | 0.50(0.36,0.63) |
| Finland | 3844.54(3128.91,4684.49) | 411.25(323.08,515.66) | 5510.61(4159.98,7077.46) | 334.80(245.12,440.13) | -0.82(-0.92,-0.73) |
| France | 16828.17(13155.88,21324.59) | 155.19(119.26,199.32) | 24921.21(18076.16,33201.61) | 139.98(100.05,188.50) | -0.25(-0.38,-0.11) |
| Gabon | 39.60(24.89,81.23) | 56.84(23.10,144.86) | 88.47(56.56,140.22) | 73.82(35.70,138.05) | 0.83(0.76,0.90) |
| Gambia | 7.80(4.51,11.98) | 20.16(3.50,54.13) | 26.66(15.57,40.57) | 24.58(8.13,50.83) | 0.71(0.63,0.79) |
| Georgia | 199.76(112.92,310.89) | 24.58(11.45,42.76) | 269.11(176.86,391.56) | 33.08(18.65,53.14) | 1.30(1.00,1.60) |
| Germany | 23410.18(17984.44,29856.75) | 143.72(108.80,185.39) | 30826.57(22684.33,40762.17) | 125.35(91.05,167.37) | -0.36(-0.50,-0.22) |
| Ghana | 126.13(71.94,197.79) | 18.22(8.13,32.84) | 537.13(308.51,851.42) | 28.61(14.65,48.50) | 1.82(1.57,2.08) |
| Greece | 1732.30(1232.21,2311.87) | 86.55(58.17,120.31) | 2899.13(2067.62,3878.09) | 97.32(66.45,134.34) | 0.38(0.22,0.54) |
| Greenland | 4.03(2.86,5.63) | 112.70(15.71,351.17) | 12.29(8.70,16.66) | 138.33(44.09,301.62) | 1.00(0.89,1.11) |
| Grenada | 4.53(3.27,6.12) | 47.95(7.81,139.77) | 7.69(5.54,10.32) | 55.34(13.92,135.40) | 0.45(0.28,0.62) |
| Guam | 1.57(0.91,2.46) | 17.86(0.19,90.36) | 6.24(3.59,9.43) | 22.55(3.22,63.79) | 0.72(0.58,0.87) |
| Guatemala | 361.35(297.02,442.16) | 91.07(66.59,122.33) | 1768.96(1403.40,2216.50) | 132.53(99.71,173.12) | 1.39(1.17,1.60) |
| Guinea | 75.29(43.86,116.94) | 18.50(7.83,34.43) | 135.08(80.18,210.98) | 20.97(9.87,37.48) | 0.31(0.23,0.39) |
| Guinea-Bissau | 8.45(4.87,13.20) | 18.93(3.48,50.39) | 17.06(9.72,26.41) | 22.46(6.06,50.80) | 0.54(0.44,0.65) |
| Guyana | 9.60(5.83,14.67) | 21.84(4.79,55.37) | 33.83(24.33,45.78) | 43.29(20.02,78.19) | 1.74(1.09,2.39) |
| Haiti | 320.51(196.97,519.98) | 85.79(45.62,151.67) | 665.39(408.52,1071.98) | 82.82(46.04,141.67) | -0.04(-0.10,0.02) |
| Honduras | 500.12(339.28,736.39) | 212.64(129.31,336.54) | 2285.06(1654.63,3039.15) | 303.82(209.52,418.71) | 1.16(1.05,1.27) |
| Hungary | 2886.48(2359.35,3545.80) | 146.76(115.17,186.31) | 3363.02(2540.86,4341.11) | 128.65(93.45,171.08) | -0.65(-0.81,-0.49) |
| Iceland | 58.51(45.00,75.05) | 158.48(88.89,254.79) | 109.67(79.29,146.03) | 144.58(82.80,226.39) | -0.16(-0.22,-0.09) |
| India | 87430.56(64860.15,112049.74) | 169.39(124.70,218.36) | 290806.29(225058.67,374648.25) | 200.39(154.45,258.99) | 0.64(0.57,0.71) |
| Indonesia | 2633.72(1750.63,3613.96) | 24.17(15.32,34.27) | 8588.14(5660.80,11490.45) | 30.46(19.56,41.50) | 0.72(0.64,0.81) |
| Iran (Islamic Republic of) | 725.47(515.02,986.02) | 23.42(15.22,33.88) | 2847.97(2081.90,3831.44) | 30.91(21.63,42.92) | 1.10(1.02,1.18) |
| Iraq | 215.39(138.72,311.58) | 23.83(12.90,38.52) | 844.81(550.93,1224.76) | 32.06(19.20,49.16) | 1.03(0.88,1.19) |
| Ireland | 2312.16(1851.41,2835.80) | 430.09(328.88,547.28) | 3874.12(2874.36,5021.28) | 381.30(272.66,508.07) | -0.18(-0.26,-0.11) |
| Israel | 582.44(432.00,778.48) | 91.51(61.63,131.22) | 1649.63(1166.11,2222.49) | 103.98(69.35,146.04) | 0.26(0.16,0.35) |
| Italy | 20105.14(15459.28,25670.54) | 170.15(128.78,219.92) | 28876.30(21939.50,37154.37) | 160.18(120.09,208.20) | -0.28(-0.34,-0.22) |
| Jamaica | 145.06(108.98,188.37) | 63.14(38.95,94.57) | 317.24(238.95,411.81) | 81.09(53.58,115.93) | 0.77(0.59,0.94) |
| Japan | 61402.33(49166.66,76291.70) | 281.46(223.39,352.20) | 90167.56(69799.28,114110.86) | 203.51(156.37,259.05) | -1.11(-1.24,-0.98) |
| Jordan | 33.80(21.90,50.72) | 24.10(9.77,47.60) | 274.14(177.76,393.26) | 33.73(18.77,53.41) | 1.50(1.33,1.67) |
| Kazakhstan | 360.34(208.38,563.38) | 23.38(11.75,39.70) | 1075.01(769.03,1461.10) | 48.46(32.26,69.34) | 2.33(1.60,3.05) |
| Kenya | 352.64(239.02,558.15) | 37.69(22.41,64.81) | 1048.90(712.04,1613.15) | 40.51(25.51,65.41) | 0.18(0.13,0.24) |
| Kiribati | 0.48(0.28,0.75) | 11.67(0.00,124.46) | 1.12(0.64,1.73) | 13.71(0.03,82.89) | 0.49(0.41,0.58) |
| Kuwait | 21.75(14.43,30.96) | 37.25(13.65,75.26) | 178.48(126.83,241.76) | 62.60(37.08,96.18) | 1.82(1.66,1.99) |
| Kyrgyzstan | 383.75(271.44,519.48) | 103.66(64.86,152.93) | 924.79(682.97,1239.82) | 163.17(111.63,231.28) | 2.32(1.89,2.76) |
| Lao People's Democratic Republic | 127.12(79.46,184.24) | 53.20(26.35,89.08) | 287.22(185.08,403.76) | 54.99(30.51,85.21) | 0.02(-0.03,0.07) |
| Latvia | 636.90(497.34,786.46) | 136.13(97.17,180.27) | 774.86(607.10,982.89) | 147.49(106.54,199.15) | -0.06(-0.38,0.26) |
| Lebanon | 98.13(65.61,135.52) | 37.15(19.20,60.71) | 301.63(192.82,437.81) | 41.04(22.66,65.42) | 0.49(0.35,0.64) |
| Lesotho | 106.98(71.65,151.90) | 106.61(55.84,177.46) | 161.21(93.95,227.73) | 125.84(59.26,202.42) | 0.65(0.44,0.86) |
| Liberia | 26.74(16.05,40.90) | 19.13(6.57,39.72) | 51.25(29.95,79.92) | 23.74(9.36,46.09) | 0.90(0.77,1.03) |
| Libya | 51.23(32.74,75.43) | 23.67(10.40,43.66) | 215.63(142.40,307.78) | 39.13(21.77,62.45) | 2.05(1.91,2.19) |
| Lithuania | 930.64(740.09,1149.95) | 159.42(117.81,208.70) | 1420.69(1119.57,1748.18) | 187.17(138.98,241.37) | 0.05(-0.24,0.34) |
| Luxembourg | 99.35(76.49,126.17) | 139.28(84.56,210.57) | 164.64(118.83,221.38) | 123.78(74.00,189.87) | -0.20(-0.28,-0.12) |
| Madagascar | 171.02(102.21,310.97) | 29.27(14.27,59.47) | 397.66(245.73,634.35) | 33.63(18.26,57.99) | 0.43(0.39,0.48) |
| Malawi | 162.14(101.35,305.23) | 36.60(18.64,77.07) | 361.84(228.97,613.01) | 43.99(24.35,80.66) | 0.59(0.53,0.65) |
| Malaysia | 249.43(163.01,371.65) | 23.74(13.22,39.15) | 885.91(564.16,1283.70) | 25.16(14.73,38.51) | 0.12(0.04,0.19) |
| Maldives | 5.77(4.10,7.98) | 58.90(11.64,160.74) | 22.05(15.02,30.58) | 61.70(23.54,121.76) | 0.22(0.13,0.31) |
| Mali | 79.87(46.53,122.52) | 17.48(7.47,32.01) | 201.95(117.69,308.37) | 20.40(9.84,34.82) | 0.54(0.46,0.63) |
| Malta | 71.20(54.58,89.63) | 129.97(74.96,201.20) | 155.94(113.83,208.40) | 119.42(71.89,182.79) | -0.34(-0.42,-0.25) |
| Marshall Islands | 0.30(0.17,0.47) | 16.13(0.00,250.72) | 0.81(0.48,1.26) | 21.05(0.01,155.98) | 0.81(0.71,0.92) |
| Mauritania | 25.94(15.09,39.86) | 22.42(7.31,46.94) | 76.95(46.17,115.51) | 30.22(13.28,54.43) | 0.93(0.82,1.05) |
| Mauritius | 24.65(15.13,36.64) | 27.74(9.56,56.92) | 176.26(136.06,225.57) | 71.97(46.61,104.94) | 2.14(1.28,3.00) |
| Mexico | 17364.26(14758.73,20445.81) | 352.64(294.91,420.95) | 46566.68(37941.35,56986.64) | 300.53(242.40,370.81) | -0.48(-0.57,-0.39) |
| Micronesia (Federated States of) | 1.00(0.58,1.54) | 16.91(0.02,110.01) | 2.19(1.30,3.35) | 24.94(0.77,105.70) | 1.23(1.20,1.25) |
| Monaco | 4.90(3.14,7.16) | 52.92(7.36,158.21) | 6.92(4.45,10.13) | 55.37(10.59,148.55) | 0.30(0.15,0.46) |
| Mongolia | 55.32(36.44,98.70) | 45.77(21.17,99.46) | 190.62(131.53,259.33) | 76.70(44.27,117.85) | 2.13(1.90,2.36) |
| Montenegro | 50.17(35.66,68.16) | 64.94(32.27,111.81) | 102.31(72.60,139.52) | 76.81(42.70,123.65) | 0.71(0.60,0.83) |
| Morocco | 404.93(246.13,647.98) | 23.84(12.74,41.21) | 1601.24(949.41,2513.33) | 37.91(21.07,61.88) | 1.78(1.66,1.91) |
| Mozambique | 227.71(135.72,441.16) | 33.97(16.99,72.26) | 487.54(306.52,833.84) | 40.29(22.57,73.74) | 0.47(0.43,0.51) |
| Myanmar | 1320.67(832.58,1857.52) | 48.58(28.58,71.51) | 3244.19(2156.27,4511.93) | 55.19(35.15,79.02) | 0.27(0.17,0.36) |
| Namibia | 81.90(53.89,116.69) | 105.24(52.01,179.74) | 167.61(106.62,244.24) | 107.49(56.01,177.56) | -0.13(-0.24,-0.01) |
| Nauru | 0.10(0.06,0.15) | 19.13(0.00,800.81) | 0.16(0.10,0.25) | 25.93(0.00,661.71) | 0.95(0.71,1.19) |
| Nepal | 1460.27(967.24,2175.65) | 139.14(86.44,216.20) | 4788.58(3262.82,6793.54) | 168.12(110.66,244.25) | 0.65(0.46,0.84) |
| Netherlands | 9470.06(7536.77,11652.25) | 366.76(285.33,459.54) | 12495.87(9393.31,16212.32) | 272.03(200.38,358.42) | -0.72(-0.85,-0.58) |
| New Zealand | 1856.11(1444.67,2340.98) | 359.69(265.70,472.40) | 3005.79(2183.28,3980.64) | 271.96(189.34,371.53) | -0.91(-1.07,-0.74) |
| Nicaragua | 225.99(166.99,303.29) | 129.42(81.68,194.37) | 891.74(665.54,1157.37) | 154.03(106.39,211.78) | 0.70(0.58,0.81) |
| Niger | 51.69(30.23,82.64) | 16.78(6.63,33.27) | 179.63(103.83,276.78) | 19.32(9.12,33.48) | 0.60(0.50,0.70) |
| Nigeria | 946.34(642.38,1346.66) | 18.34(11.50,27.52) | 2101.68(1408.39,2983.32) | 21.27(13.52,31.29) | 0.56(0.37,0.75) |
| Niue | 0.05(0.03,0.07) | 17.02(0.00,1379.13) | 0.07(0.04,0.10) | 23.17(0.00,1383.54) | 0.99(0.97,1.01) |
| North Macedonia | 89.46(60.74,129.99) | 38.83(20.15,66.99) | 210.57(140.54,307.12) | 47.44(26.64,77.38) | 0.71(0.64,0.79) |
| Northern Mariana Islands | 0.33(0.18,0.51) | 22.25(0.00,320.30) | 1.57(0.94,2.43) | 25.62(0.31,128.48) | 0.37(0.25,0.50) |
| Norway | 3968.05(3191.95,4899.14) | 442.24(343.51,561.51) | 3426.82(2547.15,4508.09) | 267.30(191.04,362.06) | -1.77(-1.89,-1.65) |
| Oman | 14.37(9.25,21.44) | 20.30(6.05,46.10) | 62.21(40.10,89.78) | 31.56(14.54,56.00) | 1.73(1.61,1.84) |
| Pakistan | 11561.82(7964.08,15829.80) | 175.84(118.48,244.54) | 23659.46(15717.48,33239.55) | 176.03(115.12,249.99) | -0.12(-0.27,0.04) |
| Palau | 0.25(0.15,0.38) | 21.12(0.00,373.95) | 0.75(0.45,1.11) | 27.34(0.01,209.10) | 0.78(0.72,0.83) |
| Palestine | 32.78(21.62,46.76) | 31.60(13.00,59.99) | 102.30(68.57,148.73) | 36.48(19.01,62.28) | 0.43(0.39,0.47) |
| Panama | 173.16(137.43,217.04) | 97.79(65.18,140.00) | 622.31(463.53,798.48) | 113.63(77.11,156.27) | 0.32(0.22,0.42) |
| Papua New Guinea | 25.00(15.04,38.02) | 12.54(4.23,26.19) | 72.36(40.24,114.16) | 13.84(5.50,26.22) | 0.25(0.14,0.35) |
| Paraguay | 228.21(166.29,310.30) | 86.47(53.79,131.41) | 1121.80(812.80,1483.52) | 157.55(106.44,219.22) | 2.03(1.87,2.19) |
| Peru | 1986.34(1394.94,2637.95) | 143.20(95.35,197.57) | 7460.42(5336.13,10006.50) | 183.25(127.58,250.66) | 0.69(0.52,0.86) |
| Philippines | 2301.60(1646.91,2935.65) | 70.10(47.77,92.70) | 6046.83(4577.28,7614.44) | 62.58(46.01,80.60) | -0.40(-0.51,-0.29) |
| Poland | 13639.65(11759.32,15869.46) | 238.20(201.67,281.49) | 13426.67(10659.50,16738.94) | 136.93(106.65,173.31) | -1.96(-2.17,-1.74) |
| Portugal | 2518.48(1979.98,3130.80) | 134.87(101.41,173.64) | 4545.04(3375.35,5899.45) | 145.34(104.32,193.52) | 0.37(0.27,0.48) |
| Puerto Rico | 453.38(346.57,579.61) | 97.71(67.03,135.51) | 1128.39(828.54,1478.66) | 119.84(82.10,165.25) | 0.56(0.44,0.68) |
| Qatar | 2.34(1.50,3.37) | 27.24(1.25,108.62) | 33.71(21.65,49.11) | 42.67(17.10,82.15) | 1.45(1.33,1.57) |
| Republic of Korea | 4965.69(3689.50,6728.16) | 148.67(106.93,206.31) | 16182.83(11385.86,21727.19) | 128.86(89.01,175.33) | -0.46(-0.55,-0.38) |
| Republic of Moldova | 243.93(176.30,331.88) | 43.50(26.97,65.91) | 514.76(382.67,678.97) | 63.69(42.72,90.57) | 0.66(0.30,1.02) |
| Romania | 1043.46(630.31,1593.66) | 28.76(16.04,46.13) | 2224.01(1389.13,3350.72) | 44.46(26.33,69.29) | 1.52(1.47,1.58) |
| Russian Federation | 23094.87(19361.53,27682.61) | 97.92(80.94,118.77) | 35884.61(30207.98,43086.73) | 108.55(90.35,131.57) | 0.02(-0.37,0.40) |
| Rwanda | 124.84(76.20,254.64) | 38.50(18.52,88.79) | 352.97(220.65,561.11) | 48.44(26.42,83.65) | 0.91(0.78,1.04) |
| Saint Kitts and Nevis | 6.13(4.77,7.62) | 116.20(28.34,289.35) | 11.46(8.69,14.55) | 128.15(43.65,270.25) | 0.35(-0.08,0.79) |
| Saint Lucia | 10.92(8.63,13.58) | 96.65(34.22,203.30) | 34.17(26.28,43.35) | 113.26(57.03,193.31) | 0.17(0.01,0.32) |
| Saint Vincent and the Grenadines | 4.94(3.74,6.40) | 53.37(10.39,147.09) | 9.15(6.53,12.38) | 49.29(13.59,115.48) | -0.09(-0.33,0.14) |
| Samoa | 1.84(1.07,2.84) | 18.08(0.33,83.83) | 3.93(2.34,5.92) | 23.08(2.09,76.11) | 0.65(0.59,0.71) |
| San Marino | 3.22(2.23,4.50) | 70.25(6.94,238.72) | 5.44(3.65,7.77) | 59.13(9.94,168.05) | -0.32(-0.48,-0.15) |
| Sao Tome and Principe | 1.74(1.02,2.69) | 21.41(0.33,102.37) | 3.50(2.05,5.42) | 28.39(2.10,99.52) | 0.97(0.87,1.07) |
| Saudi Arabia | 133.77(85.52,197.50) | 21.64(11.06,36.73) | 588.62(384.88,841.21) | 32.95(19.44,50.38) | 1.38(1.34,1.42) |
| Senegal | 76.81(44.71,117.37) | 20.41(8.66,37.37) | 224.37(132.93,348.02) | 25.04(12.42,43.14) | 0.58(0.52,0.64) |
| Serbia | 1199.48(868.73,1619.38) | 80.55(54.52,114.17) | 2064.31(1481.96,2762.89) | 91.93(62.68,127.72) | 0.33(0.26,0.40) |
| Seychelles | 1.47(0.94,2.17) | 20.27(0.27,103.54) | 3.43(2.07,5.12) | 24.06(1.84,82.94) | 0.60(0.56,0.65) |
| Sierra Leone | 45.78(26.80,71.19) | 18.79(7.24,36.85) | 90.30(52.58,139.41) | 21.58(9.40,39.33) | 0.46(0.34,0.59) |
| Singapore | 160.94(119.14,216.05) | 63.02(38.65,96.66) | 677.89(448.99,976.22) | 60.56(36.49,92.86) | -0.04(-0.12,0.04) |
| Slovakia | 492.39(357.16,655.90) | 62.52(40.76,89.90) | 838.88(599.98,1145.65) | 65.18(42.96,94.32) | 0.07(-0.02,0.16) |
| Slovenia | 634.49(510.84,780.49) | 201.12(148.18,265.37) | 1113.08(821.94,1467.96) | 191.63(132.00,266.00) | -0.23(-0.41,-0.05) |
| Solomon Islands | 2.23(1.30,3.52) | 14.92(0.45,63.74) | 7.10(4.17,10.73) | 19.71(3.26,53.63) | 0.73(0.66,0.80) |
| Somalia | 88.43(54.14,172.81) | 39.59(18.21,89.79) | 250.09(153.03,474.79) | 38.80(20.13,80.59) | -0.06(-0.09,-0.02) |
| South Africa | 4393.26(3360.26,5547.25) | 180.58(133.49,234.10) | 8719.51(6850.66,10950.59) | 155.89(119.60,199.48) | -0.79(-1.06,-0.52) |
| South Sudan | 96.16(59.09,173.20) | 31.60(14.79,66.06) | 138.76(85.46,230.25) | 34.88(17.17,65.86) | 0.36(0.29,0.43) |
| Spain | 10047.20(7834.33,12633.32) | 137.59(104.92,176.04) | 16360.09(11935.34,21792.10) | 134.81(96.59,181.97) | -0.12(-0.17,-0.06) |
| Sri Lanka | 224.01(147.40,326.89) | 17.99(10.00,29.26) | 809.13(491.27,1214.55) | 22.63(12.55,35.94) | 0.94(0.85,1.04) |
| Sudan | 233.54(143.76,364.98) | 21.74(11.28,37.64) | 644.79(432.17,935.58) | 30.54(18.59,47.25) | 1.31(1.21,1.41) |
| Suriname | 11.21(7.35,16.45) | 37.17(10.05,87.97) | 30.08(20.05,44.16) | 38.46(15.66,75.75) | 0.27(0.17,0.36) |
| Sweden | 6065.76(4750.00,7648.10) | 309.68(235.66,399.31) | 6199.54(4593.09,8130.21) | 229.19(164.93,307.17) | -1.14(-1.24,-1.03) |
| Switzerland | 2966.01(2279.99,3786.28) | 223.95(165.16,295.14) | 4238.30(3063.90,5632.25) | 187.14(130.54,255.27) | -0.56(-0.62,-0.50) |
| Syrian Arab Republic | 141.36(94.33,200.80) | 23.65(12.76,38.58) | 565.63(382.87,786.11) | 35.48(21.67,52.89) | 1.44(1.36,1.53) |
| Taiwan (Province of China) | 2754.06(2171.99,3451.55) | 137.24(103.73,177.83) | 6880.47(5237.86,8878.26) | 120.57(89.32,158.85) | 0.36(0.06,0.66) |
| Tajikistan | 128.48(80.71,190.43) | 39.60(19.74,67.64) | 372.52(249.19,527.29) | 56.48(33.24,87.07) | 1.20(1.12,1.27) |
| Thailand | 2299.75(1567.44,3408.60) | 57.44(37.24,88.05) | 8657.99(6018.26,12153.52) | 60.80(41.20,86.88) | 0.10(0.03,0.16) |
| Timor-Leste | 10.78(6.89,15.50) | 39.54(10.07,93.02) | 54.85(35.24,77.77) | 51.00(22.85,90.27) | 0.94(0.87,1.00) |
| Togo | 26.69(15.63,41.68) | 20.02(6.66,42.32) | 102.30(59.60,157.43) | 25.09(11.14,45.14) | 0.77(0.65,0.90) |
| Tokelau | 0.03(0.02,0.04) | 14.96(0.00,1981.49) | 0.04(0.02,0.06) | 21.02(0.00,1978.29) | 1.11(1.06,1.17) |
| Tonga | 1.11(0.64,1.74) | 16.79(0.04,102.81) | 1.98(1.15,3.06) | 20.59(0.46,91.97) | 0.55(0.50,0.60) |
| Trinidad and Tobago | 150.80(123.53,183.35) | 145.50(99.10,204.45) | 368.35(284.68,465.28) | 142.61(97.79,197.28) | -0.28(-0.40,-0.17) |
| Tunisia | 130.70(83.05,189.62) | 21.44(10.85,35.87) | 550.86(363.70,795.51) | 32.47(19.29,50.27) | 1.51(1.44,1.59) |
| Turkey | 3807.10(2681.81,5274.46) | 93.69(63.52,133.35) | 11828.73(8455.09,15793.71) | 100.19(70.10,135.88) | 0.32(0.19,0.46) |
| Turkmenistan | 43.62(24.31,69.42) | 19.39(6.95,39.03) | 156.83(107.08,223.16) | 33.22(18.59,53.89) | 1.90(1.56,2.24) |
| Tuvalu | 0.13(0.07,0.20) | 15.16(0.00,489.10) | 0.27(0.16,0.41) | 20.56(0.00,341.75) | 0.97(0.95,0.99) |
| Uganda | 253.12(154.32,431.92) | 34.44(17.81,64.57) | 675.80(408.93,1096.60) | 42.44(23.25,73.07) | 0.74(0.64,0.83) |
| Ukraine | 3981.68(2826.70,5306.30) | 41.48(28.37,56.79) | 4913.21(3518.39,6641.36) | 46.28(32.06,64.09) | 0.08(-0.06,0.22) |
| United Arab Emirates | 15.37(10.41,22.18) | 44.84(14.82,97.79) | 135.73(89.56,194.91) | 40.03(21.23,66.15) | 0.23(-0.06,0.52) |
| United Kingdom | 47682.38(39993.06,56717.73) | 400.02(332.23,479.76) | 48592.94(37610.29,61630.94) | 295.46(226.38,377.71) | -0.67(-0.89,-0.44) |
| United Republic of Tanzania | 467.88(281.66,834.41) | 37.05(19.78,70.72) | 1241.90(778.46,1955.21) | 44.11(25.74,72.59) | 0.49(0.44,0.54) |
| United States Virgin Islands | 5.40(3.73,7.58) | 55.13(9.74,155.23) | 14.43(9.64,20.82) | 57.21(18.03,126.44) | -0.04(-0.15,0.07) |
| United States of America | 77421.27(61655.59,95962.75) | 184.57(145.82,230.22) | 138962.66(106042.00,177227.27) | 176.93(134.20,226.70) | -0.18(-0.38,0.02) |
| Uruguay | 649.80(519.81,805.42) | 125.51(91.95,166.69) | 1201.49(957.52,1490.35) | 168.89(126.21,220.41) | 1.05(0.86,1.25) |
| Uzbekistan | 953.32(621.24,1378.83) | 69.15(41.59,105.44) | 3291.45(2367.81,4475.25) | 107.53(74.27,150.56) | 1.77(1.43,2.11) |
| Vanuatu | 1.01(0.58,1.58) | 14.62(0.02,94.50) | 3.51(2.06,5.42) | 18.16(1.35,63.52) | 0.66(0.60,0.71) |
| Venezuela (Bolivarian Republic of) | 2164.82(1771.46,2616.40) | 193.03(150.69,242.41) | 8035.25(6208.11,10187.64) | 210.76(158.81,272.46) | 0.08(-0.03,0.18) |
| Viet Nam | 2631.35(1744.71,3712.94) | 52.74(33.34,76.84) | 8994.28(6118.96,12555.44) | 74.10(49.16,105.27) | 1.15(1.05,1.25) |
| Yemen | 116.19(75.50,177.49) | 21.01(10.75,37.18) | 459.54(302.19,675.45) | 29.40(17.22,46.60) | 1.31(1.19,1.42) |
| Zambia | 103.08(62.60,189.73) | 33.35(15.55,70.78) | 297.53(185.18,478.31) | 41.33(22.15,72.66) | 0.69(0.53,0.85) |
| Zimbabwe | 355.36(229.58,488.34) | 75.35(42.59,113.15) | 627.44(386.45,859.68) | 81.09(45.09,118.78) | 0.28(0.05,0.50) |

**Abbreviations: ASR, age-standardised rate; CI: confidence interval; DALY=disability-adjusted life-years; UI: uncertainly interval; EAPC, estimated annual percentage change.**

**Table S6. Age-standardised incidence rates of elderly-onset rheumatoid arthritis with frontier analysis across 204 countries and territories in 1990 and 2021.**

| **Location** | **Incidence, 1990** | | | | | **Incidence, 2021** | | | | |
| --- | --- | --- | --- | --- | --- | --- | --- | --- | --- | --- |
|  | **SDI** | **ASRs** | **Frontier** | **Effective difference** | **Effective difference rank (ASRs rank)** | **SDI** | **ASRs** | **Frontier** | **Effective difference** | **Effective difference rank (ASRs rank)** |
| Afghanistan | 0.174 | 2.21(0.55,4.98) | 2.207 | 0.000 | 1(3) | 0.337 | 2.66(0.75,5.79) | 2.090 | 0.571 | 3(3) |
| Albania | 0.558 | 6.66(1.67,15.22) | 2.057 | 4.602 | 87(86) | 0.707 | 11.87(4.67,21.88) | 2.057 | 9.813 | 107(107) |
| Algeria | 0.460 | 2.81(0.94,5.64) | 2.059 | 0.751 | 19(15) | 0.660 | 4.20(1.88,7.44) | 2.058 | 2.146 | 24(22) |
| American Samoa | 0.614 | 3.50(0.00,160.18) | 2.057 | 1.443 | 42(37) | 0.724 | 4.30(0.00,78.48) | 2.057 | 2.243 | 29(27) |
| Andorra | 0.761 | 13.04(0.03,84.25) | 2.058 | 10.982 | 133(133) | 0.869 | 13.49(0.78,50.97) | 2.058 | 11.433 | 120(120) |
| Angola | 0.271 | 9.91(3.86,18.81) | 2.091 | 7.816 | 111(108) | 0.454 | 12.53(6.09,21.23) | 2.058 | 10.470 | 111(111) |
| Antigua and Barbuda | 0.612 | 22.93(0.89,104.13) | 2.057 | 20.876 | 166(166) | 0.750 | 25.47(3.39,80.46) | 2.058 | 23.414 | 157(157) |
| Argentina | 0.587 | 15.44(9.24,22.97) | 2.057 | 13.383 | 145(145) | 0.723 | 16.14(9.59,24.14) | 2.058 | 14.081 | 136(136) |
| Armenia | 0.544 | 4.26(1.04,10.05) | 2.057 | 2.205 | 54(49) | 0.702 | 5.69(1.92,11.53) | 2.057 | 3.637 | 55(53) |
| Australia | 0.726 | 64.84(40.97,92.81) | 2.057 | 62.778 | 197(197) | 0.844 | 72.24(46.80,102.49) | 2.060 | 70.182 | 196(196) |
| Austria | 0.750 | 32.14(18.79,48.74) | 2.058 | 30.085 | 183(183) | 0.854 | 30.83(18.15,45.29) | 2.057 | 28.768 | 170(170) |
| Azerbaijan | 0.596 | 3.49(1.02,7.64) | 2.059 | 1.433 | 41(36) | 0.695 | 4.29(1.68,8.14) | 2.057 | 2.236 | 27(25) |
| Bahamas | 0.694 | 17.73(2.00,58.53) | 2.058 | 15.675 | 150(150) | 0.805 | 19.25(5.43,44.58) | 2.058 | 17.189 | 147(147) |
| Bahrain | 0.585 | 5.56(0.00,37.03) | 2.059 | 3.500 | 71(69) | 0.753 | 10.02(1.78,26.76) | 2.058 | 7.958 | 95(94) |
| Bangladesh | 0.229 | 10.32(6.14,15.27) | 2.091 | 8.234 | 116(114) | 0.492 | 15.95(10.14,22.97) | 2.058 | 13.889 | 135(135) |
| Barbados | 0.654 | 34.26(13.10,69.72) | 2.062 | 32.199 | 184(184) | 0.747 | 36.35(15.67,67.37) | 2.058 | 34.287 | 182(182) |
| Belarus | 0.622 | 3.67(1.28,7.15) | 2.057 | 1.609 | 46(41) | 0.784 | 5.51(2.19,10.34) | 2.057 | 3.452 | 49(47) |
| Belgium | 0.737 | 35.76(21.92,52.64) | 2.058 | 33.699 | 187(187) | 0.854 | 36.88(22.58,53.44) | 2.060 | 34.821 | 183(183) |
| Belize | 0.424 | 15.60(0.65,67.76) | 2.090 | 13.510 | 146(146) | 0.610 | 16.50(3.36,43.94) | 2.058 | 14.442 | 137(137) |
| Benin | 0.219 | 6.02(1.64,13.46) | 2.092 | 3.929 | 78(76) | 0.373 | 7.16(2.79,13.39) | 2.090 | 5.074 | 67(66) |
| Bermuda | 0.696 | 22.66(0.90,99.01) | 2.057 | 20.598 | 165(165) | 0.821 | 32.70(7.55,84.63) | 2.058 | 30.646 | 174(174) |
| Bhutan | 0.215 | 24.95(4.65,66.70) | 2.092 | 22.860 | 173(173) | 0.473 | 41.57(16.11,79.96) | 2.058 | 39.513 | 187(187) |
| Bolivia (Plurinational State of) | 0.424 | 24.55(12.81,40.87) | 2.092 | 22.459 | 172(172) | 0.599 | 36.05(20.22,55.54) | 2.058 | 33.997 | 181(181) |
| Bosnia and Herzegovina | 0.541 | 11.07(4.10,21.96) | 2.058 | 9.015 | 121(121) | 0.723 | 14.40(6.27,26.07) | 2.058 | 12.341 | 128(128) |
| Botswana | 0.418 | 12.15(2.38,31.38) | 2.092 | 10.058 | 129(130) | 0.643 | 15.06(5.09,30.53) | 2.057 | 13.003 | 131(131) |
| Brazil | 0.500 | 5.46(2.54,9.45) | 2.058 | 3.403 | 70(66) | 0.653 | 5.59(2.84,9.25) | 2.058 | 3.534 | 51(49) |
| Brunei Darussalam | 0.666 | 26.49(1.86,94.77) | 2.058 | 24.431 | 176(176) | 0.810 | 35.84(11.28,76.90) | 2.057 | 33.785 | 180(180) |
| Bulgaria | 0.633 | 4.70(1.88,8.72) | 2.058 | 2.639 | 58(53) | 0.768 | 5.87(2.58,10.63) | 2.061 | 3.812 | 56(54) |
| Burkina Faso | 0.130 | 6.28(2.28,12.20) | 5.179 | 1.104 | 28(79) | 0.285 | 6.92(3.09,12.10) | 2.091 | 4.827 | 62(61) |
| Burundi | 0.206 | 10.03(3.66,19.52) | 2.091 | 7.942 | 113(111) | 0.289 | 10.58(4.66,18.88) | 2.091 | 8.488 | 97(97) |
| Cabo Verde | 0.277 | 4.94(0.05,25.48) | 2.091 | 2.848 | 61(57) | 0.534 | 7.80(0.79,24.71) | 2.058 | 5.739 | 74(73) |
| Cambodia | 0.289 | 5.20(1.85,10.41) | 2.092 | 3.110 | 65(60) | 0.474 | 9.76(4.83,16.10) | 2.057 | 7.702 | 92(91) |
| Cameroon | 0.303 | 6.95(2.56,13.51) | 2.092 | 4.854 | 90(89) | 0.480 | 8.16(3.91,13.74) | 2.058 | 6.103 | 77(76) |
| Canada | 0.782 | 39.21(28.31,51.03) | 2.058 | 37.157 | 189(189) | 0.873 | 52.25(37.88,66.58) | 2.061 | 50.193 | 192(192) |
| Central African Republic | 0.217 | 9.36(2.22,22.29) | 2.090 | 7.273 | 102(99) | 0.309 | 9.49(2.98,19.80) | 2.091 | 7.398 | 90(89) |
| Chad | 0.115 | 5.45(1.65,11.65) | 5.177 | 0.277 | 8(65) | 0.240 | 5.96(2.34,11.29) | 2.092 | 3.866 | 57(55) |
| Chile | 0.586 | 28.86(15.78,46.41) | 2.057 | 26.803 | 181(181) | 0.772 | 47.12(27.05,72.97) | 2.059 | 45.065 | 189(189) |
| China | 0.459 | 23.42(13.20,36.33) | 2.059 | 21.363 | 167(167) | 0.722 | 30.07(18.35,44.31) | 2.057 | 28.018 | 169(169) |
| Colombia | 0.481 | 13.33(7.22,21.41) | 2.058 | 11.277 | 137(137) | 0.655 | 16.81(9.79,25.61) | 2.057 | 14.748 | 138(138) |
| Comoros | 0.270 | 11.71(0.66,44.12) | 2.093 | 9.616 | 125(125) | 0.476 | 14.37(2.84,36.49) | 2.057 | 12.317 | 127(127) |
| Congo | 0.421 | 11.37(2.92,25.88) | 2.091 | 9.284 | 124(124) | 0.583 | 13.87(5.28,26.73) | 2.058 | 11.807 | 122(122) |
| Cook Islands | 0.565 | 2.77(0.00,260.52) | 2.057 | 0.718 | 16(12) | 0.779 | 3.49(0.00,119.89) | 2.057 | 1.436 | 10(8) |
| Costa Rica | 0.534 | 21.90(9.24,40.76) | 2.057 | 19.845 | 163(163) | 0.700 | 29.60(15.01,48.98) | 2.058 | 27.543 | 166(166) |
| Côte d'Ivoire | 0.279 | 6.84(2.39,13.65) | 2.092 | 4.751 | 88(87) | 0.426 | 8.23(3.81,14.06) | 2.087 | 6.143 | 79(78) |
| Croatia | 0.669 | 12.23(4.87,23.08) | 2.058 | 10.175 | 131(131) | 0.798 | 17.14(7.41,30.11) | 2.058 | 15.078 | 141(141) |
| Cuba | 0.558 | 13.93(7.48,22.48) | 2.057 | 11.875 | 141(141) | 0.669 | 17.18(9.65,26.81) | 2.061 | 15.120 | 142(142) |
| Cyprus | 0.648 | 66.14(30.87,114.02) | 2.058 | 64.080 | 198(198) | 0.836 | 80.46(42.34,127.21) | 2.057 | 78.408 | 201(201) |
| Czechia | 0.682 | 5.41(2.20,9.95) | 2.058 | 3.348 | 68(64) | 0.828 | 7.29(3.29,12.79) | 2.059 | 5.233 | 69(68) |
| Democratic People's Republic of Korea | 0.498 | 17.97(9.46,28.89) | 2.057 | 15.915 | 151(151) | 0.570 | 24.29(14.29,37.11) | 2.058 | 22.227 | 156(156) |
| Democratic Republic of the Congo | 0.290 | 10.06(5.06,16.67) | 2.090 | 7.973 | 114(112) | 0.383 | 11.55(6.25,18.39) | 2.091 | 9.460 | 106(106) |
| Denmark | 0.801 | 62.61(40.22,88.59) | 2.057 | 60.557 | 195(195) | 0.896 | 85.19(55.20,119.69) | 2.061 | 83.125 | 203(203) |
| Djibouti | 0.338 | 11.19(0.13,55.73) | 2.092 | 9.099 | 122(122) | 0.488 | 13.38(2.78,33.39) | 2.060 | 11.316 | 119(119) |
| Dominica | 0.564 | 21.59(0.95,94.91) | 2.057 | 19.530 | 162(162) | 0.747 | 21.86(1.87,80.96) | 2.058 | 19.803 | 150(150) |
| Dominican Republic | 0.443 | 18.67(11.82,27.42) | 2.060 | 16.611 | 154(154) | 0.619 | 22.78(16.23,30.91) | 2.058 | 20.725 | 152(152) |
| Ecuador | 0.518 | 35.14(22.28,50.96) | 2.057 | 33.087 | 186(186) | 0.661 | 49.69(32.14,70.61) | 2.059 | 47.632 | 191(191) |
| Egypt | 0.417 | 2.81(1.08,5.30) | 2.091 | 0.719 | 17(14) | 0.607 | 4.38(2.02,7.49) | 2.062 | 2.315 | 32(30) |
| El Salvador | 0.373 | 9.52(4.32,17.10) | 2.091 | 7.433 | 103(102) | 0.564 | 13.87(7.57,22.16) | 2.058 | 11.809 | 123(123) |
| Equatorial Guinea | 0.269 | 10.20(0.34,42.13) | 2.091 | 8.106 | 115(113) | 0.658 | 17.33(3.45,44.44) | 2.058 | 15.269 | 144(144) |
| Eritrea | 0.216 | 9.95(2.43,23.61) | 2.091 | 7.855 | 112(109) | 0.404 | 12.57(4.84,23.93) | 2.091 | 10.480 | 112(112) |
| Estonia | 0.675 | 5.68(1.15,14.06) | 2.057 | 3.623 | 73(71) | 0.845 | 6.51(1.60,15.33) | 2.058 | 4.449 | 59(58) |
| Eswatini | 0.399 | 13.42(1.42,42.84) | 2.090 | 11.327 | 139(139) | 0.585 | 14.99(3.21,37.46) | 2.057 | 12.929 | 130(130) |
| Ethiopia | 0.148 | 10.81(5.22,18.20) | 5.183 | 5.630 | 95(117) | 0.359 | 11.26(6.11,17.62) | 2.090 | 9.172 | 103(104) |
| Fiji | 0.535 | 2.33(0.00,16.87) | 2.059 | 0.271 | 7(5) | 0.675 | 2.59(0.07,10.75) | 2.057 | 0.529 | 2(2) |
| Finland | 0.756 | 63.31(41.48,89.44) | 2.057 | 61.256 | 196(196) | 0.860 | 73.81(48.19,103.01) | 2.057 | 71.756 | 200(200) |
| France | 0.731 | 34.86(22.45,48.97) | 2.058 | 32.807 | 185(185) | 0.838 | 41.49(26.55,57.73) | 2.057 | 39.433 | 186(186) |
| Gabon | 0.455 | 13.18(2.75,33.11) | 2.057 | 11.123 | 136(136) | 0.635 | 16.99(5.19,36.26) | 2.062 | 14.932 | 140(140) |
| Gambia | 0.239 | 6.34(0.27,25.01) | 2.090 | 4.249 | 82(81) | 0.410 | 7.62(1.50,19.37) | 2.090 | 5.527 | 72(71) |
| Georgia | 0.740 | 4.45(1.56,8.99) | 2.057 | 2.398 | 56(51) | 0.847 | 4.95(1.79,9.74) | 2.057 | 2.894 | 41(39) |
| Germany | 0.817 | 25.81(16.50,37.17) | 2.059 | 23.753 | 175(175) | 0.903 | 28.55(18.95,40.67) | 2.057 | 26.489 | 163(163) |
| Ghana | 0.373 | 5.73(2.07,11.25) | 2.090 | 3.643 | 74(72) | 0.565 | 7.66(3.59,13.05) | 2.057 | 5.604 | 73(72) |
| Greece | 0.674 | 16.23(9.10,25.46) | 2.061 | 14.171 | 147(147) | 0.792 | 17.72(10.58,26.54) | 2.058 | 15.661 | 145(145) |
| Greenland | 0.732 | 17.27(0.00,149.89) | 2.058 | 15.214 | 148(148) | 0.826 | 29.99(1.78,111.39) | 2.058 | 27.933 | 167(167) |
| Grenada | 0.437 | 9.50(0.03,62.76) | 2.070 | 7.434 | 104(100) | 0.669 | 12.49(0.37,56.30) | 2.058 | 10.428 | 110(110) |
| Guam | 0.676 | 3.40(0.00,52.76) | 2.059 | 1.346 | 37(32) | 0.804 | 4.01(0.01,24.70) | 2.058 | 1.956 | 20(18) |
| Guatemala | 0.312 | 13.87(7.42,22.83) | 2.091 | 11.779 | 140(140) | 0.540 | 24.13(15.67,35.03) | 2.057 | 22.078 | 155(155) |
| Guinea | 0.178 | 5.93(2.00,11.96) | 2.092 | 3.837 | 77(75) | 0.336 | 6.56(2.61,12.17) | 2.090 | 4.468 | 60(59) |
| Guinea-Bissau | 0.208 | 6.15(0.34,22.73) | 2.090 | 4.057 | 80(78) | 0.353 | 7.34(1.10,20.70) | 2.090 | 5.245 | 70(69) |
| Guyana | 0.460 | 6.86(0.62,23.64) | 2.058 | 4.807 | 89(88) | 0.651 | 8.98(2.08,22.66) | 2.060 | 6.923 | 83(82) |
| Haiti | 0.310 | 9.63(4.63,16.91) | 2.092 | 7.534 | 106(104) | 0.448 | 11.06(6.18,17.90) | 2.058 | 8.997 | 101(101) |
| Honduras | 0.332 | 25.27(12.41,43.19) | 2.092 | 23.182 | 174(174) | 0.513 | 33.17(18.67,52.68) | 2.058 | 31.110 | 175(175) |
| Hungary | 0.649 | 9.62(4.06,17.23) | 2.059 | 7.558 | 107(103) | 0.791 | 11.24(5.05,18.98) | 2.062 | 9.181 | 104(103) |
| Iceland | 0.764 | 29.94(7.87,69.01) | 2.057 | 27.887 | 182(182) | 0.876 | 35.36(13.31,68.04) | 2.058 | 33.299 | 179(179) |
| India | 0.333 | 40.09(24.94,58.25) | 2.091 | 38.001 | 191(191) | 0.575 | 58.20(36.92,82.63) | 2.060 | 56.140 | 193(193) |
| Indonesia | 0.457 | 3.90(1.89,6.44) | 2.057 | 1.843 | 49(44) | 0.657 | 5.63(3.12,8.72) | 2.058 | 3.574 | 52(50) |
| Iran (Islamic Republic of) | 0.454 | 3.09(1.15,5.89) | 2.057 | 1.031 | 26(22) | 0.697 | 4.02(1.85,6.97) | 2.057 | 1.964 | 22(20) |
| Iraq | 0.412 | 2.97(0.86,6.40) | 2.091 | 0.879 | 21(17) | 0.663 | 4.80(2.06,8.70) | 2.058 | 2.741 | 38(36) |
| Ireland | 0.720 | 76.09(43.08,113.34) | 2.059 | 74.031 | 201(201) | 0.874 | 104.89(63.02,153.05) | 2.057 | ##### | 204(204) |
| Israel | 0.709 | 21.15(11.68,33.60) | 2.058 | 19.094 | 161(161) | 0.809 | 28.87(17.57,42.37) | 2.057 | 26.809 | 164(164) |
| Italy | 0.706 | 37.86(21.69,57.68) | 2.060 | 35.800 | 188(188) | 0.806 | 39.93(24.09,58.88) | 2.058 | 37.870 | 185(185) |
| Jamaica | 0.535 | 14.04(6.70,24.78) | 2.058 | 11.986 | 142(142) | 0.683 | 15.26(8.18,25.21) | 2.057 | 13.202 | 132(132) |
| Japan | 0.790 | 50.03(29.59,75.80) | 2.058 | 47.971 | 192(192) | 0.871 | 35.16(20.93,52.43) | 2.058 | 33.099 | 178(178) |
| Jordan | 0.539 | 3.12(0.25,10.27) | 2.057 | 1.062 | 27(23) | 0.725 | 4.63(1.55,9.37) | 2.061 | 2.566 | 37(35) |
| Kazakhstan | 0.589 | 4.09(1.64,7.74) | 2.057 | 2.031 | 52(47) | 0.725 | 5.50(2.45,9.84) | 2.057 | 3.443 | 48(46) |
| Kenya | 0.334 | 13.17(6.19,22.62) | 2.091 | 11.076 | 134(134) | 0.524 | 14.53(7.69,23.27) | 2.059 | 12.475 | 129(129) |
| Kiribati | 0.410 | 2.16(0.00,96.56) | 2.092 | 0.068 | 4(1) | 0.527 | 2.50(0.00,52.88) | 2.062 | 0.437 | 1(1) |
| Kuwait | 0.665 | 3.97(0.09,16.90) | 2.057 | 1.914 | 51(46) | 0.847 | 5.58(1.33,13.14) | 2.058 | 3.520 | 50(48) |
| Kyrgyzstan | 0.519 | 19.18(8.51,34.12) | 2.059 | 17.120 | 156(156) | 0.604 | 30.00(14.46,51.74) | 2.058 | 27.939 | 168(168) |
| Lao People's Democratic Republic | 0.264 | 5.07(1.30,11.56) | 2.091 | 2.982 | 64(59) | 0.489 | 8.66(3.58,16.00) | 2.058 | 6.606 | 80(79) |
| Latvia | 0.680 | 3.62(0.79,8.76) | 2.057 | 1.566 | 45(40) | 0.831 | 4.25(1.08,9.72) | 2.058 | 2.192 | 25(23) |
| Lebanon | 0.537 | 3.19(0.50,8.62) | 2.058 | 1.135 | 29(24) | 0.745 | 4.02(1.23,8.49) | 2.059 | 1.956 | 21(19) |
| Lesotho | 0.339 | 11.87(3.13,27.31) | 2.092 | 9.783 | 127(127) | 0.510 | 13.20(4.06,28.51) | 2.062 | 11.137 | 116(116) |
| Liberia | 0.235 | 5.64(1.02,14.69) | 2.091 | 3.553 | 72(70) | 0.352 | 7.08(1.98,15.70) | 2.091 | 4.989 | 65(65) |
| Libya | 0.528 | 2.73(0.31,8.11) | 2.067 | 0.660 | 14(10) | 0.726 | 3.64(0.98,8.20) | 2.058 | 1.577 | 13(11) |
| Lithuania | 0.669 | 4.67(1.21,10.47) | 2.057 | 2.617 | 57(52) | 0.856 | 5.64(1.75,11.88) | 2.058 | 3.581 | 53(51) |
| Luxembourg | 0.781 | 27.23(9.71,55.56) | 2.060 | 25.168 | 180(180) | 0.884 | 31.81(14.00,57.27) | 2.059 | 29.752 | 171(171) |
| Madagascar | 0.280 | 9.13(4.03,16.30) | 2.091 | 7.038 | 101(98) | 0.400 | 10.65(5.36,17.53) | 2.091 | 8.558 | 98(98) |
| Malawi | 0.204 | 11.30(4.88,20.55) | 2.090 | 9.212 | 123(123) | 0.385 | 13.71(6.71,23.38) | 2.091 | 11.620 | 121(121) |
| Malaysia | 0.546 | 3.80(1.41,7.46) | 2.057 | 1.747 | 48(43) | 0.743 | 5.08(2.46,8.74) | 2.057 | 3.026 | 44(42) |
| Maldives | 0.332 | 5.47(0.00,53.58) | 2.091 | 3.376 | 69(67) | 0.651 | 9.94(0.78,34.00) | 2.057 | 7.880 | 94(93) |
| Mali | 0.127 | 5.49(1.84,11.21) | 5.182 | 0.312 | 9(68) | 0.269 | 6.15(2.68,10.98) | 2.091 | 4.062 | 58(57) |
| Malta | 0.657 | 23.68(6.90,52.31) | 2.057 | 21.619 | 168(168) | 0.802 | 26.79(11.33,49.19) | 2.057 | 24.730 | 158(158) |
| Marshall Islands | 0.431 | 3.24(0.00,210.87) | 2.075 | 1.167 | 31(27) | 0.574 | 4.19(0.00,108.62) | 2.057 | 2.136 | 23(21) |
| Mauritania | 0.336 | 6.57(1.20,16.98) | 2.091 | 4.477 | 84(84) | 0.499 | 9.40(3.16,18.90) | 2.057 | 7.341 | 89(88) |
| Mauritius | 0.545 | 7.75(1.18,21.50) | 2.062 | 5.690 | 96(94) | 0.718 | 11.33(3.81,23.25) | 2.057 | 9.274 | 105(105) |
| Mexico | 0.505 | 50.18(28.77,76.64) | 2.058 | 48.120 | 193(193) | 0.665 | 44.92(26.75,67.64) | 2.063 | 42.856 | 188(188) |
| Micronesia (Federated States of) | 0.463 | 3.24(0.00,72.81) | 2.057 | 1.184 | 32(26) | 0.588 | 4.56(0.00,55.75) | 2.058 | 2.507 | 36(34) |
| Monaco | 0.845 | 12.14(0.05,71.90) | 2.057 | 10.081 | 130(129) | 0.908 | 12.90(0.25,61.58) | 2.057 | 10.840 | 114(114) |
| Mongolia | 0.467 | 7.17(1.72,17.56) | 2.057 | 5.117 | 91(90) | 0.618 | 9.28(3.14,19.05) | 2.057 | 7.219 | 86(85) |
| Montenegro | 0.674 | 8.63(1.07,24.96) | 2.058 | 6.570 | 100(97) | 0.796 | 10.96(2.57,25.76) | 2.058 | 8.907 | 99(99) |
| Morocco | 0.358 | 2.43(0.80,4.91) | 2.093 | 0.335 | 10(6) | 0.563 | 3.55(1.55,6.29) | 2.058 | 1.488 | 12(10) |
| Mozambique | 0.173 | 10.96(5.11,18.88) | 5.191 | 5.770 | 98(119) | 0.326 | 12.82(6.47,21.05) | 2.091 | 10.724 | 113(113) |
| Myanmar | 0.319 | 5.29(2.60,8.96) | 2.090 | 3.203 | 67(62) | 0.534 | 9.32(5.15,14.60) | 2.057 | 7.263 | 88(87) |
| Namibia | 0.450 | 11.88(2.62,29.19) | 2.058 | 9.820 | 128(128) | 0.618 | 15.61(5.32,31.88) | 2.057 | 13.552 | 134(134) |
| Nauru | 0.539 | 3.71(0.00,752.02) | 2.059 | 1.646 | 47(42) | 0.625 | 4.85(0.00,596.20) | 2.058 | 2.796 | 39(37) |
| Nepal | 0.200 | 20.86(11.58,32.90) | 2.092 | 18.767 | 160(160) | 0.433 | 35.03(20.91,51.43) | 2.070 | 32.955 | 177(177) |
| Netherlands | 0.795 | 78.86(49.06,113.66) | 2.057 | 76.808 | 204(204) | 0.888 | 81.95(52.34,116.09) | 2.059 | 79.890 | 202(202) |
| New Zealand | 0.752 | 76.77(40.51,125.12) | 2.063 | 74.707 | 202(202) | 0.849 | 73.46(41.45,113.57) | 2.059 | 71.399 | 199(199) |
| Nicaragua | 0.346 | 27.08(14.37,44.85) | 2.092 | 24.983 | 179(179) | 0.524 | 37.70(23.91,55.83) | 2.058 | 35.638 | 184(184) |
| Niger | 0.081 | 5.36(1.55,11.71) | 5.361 | 0.000 | 2(63) | 0.168 | 6.03(2.57,10.80) | 5.179 | 0.852 | 6(56) |
| Nigeria | 0.306 | 4.84(2.34,8.13) | 2.092 | 2.751 | 60(55) | 0.503 | 5.65(2.98,9.08) | 2.058 | 3.593 | 54(52) |
| Niue | 0.588 | 3.21(0.00,1334.35) | 2.058 | 1.155 | 30(25) | 0.726 | 4.41(0.00,1324.84) | 2.057 | 2.355 | 33(31) |
| North Macedonia | 0.609 | 4.92(1.00,12.24) | 2.057 | 2.861 | 62(56) | 0.751 | 7.25(2.35,14.93) | 2.057 | 5.188 | 68(67) |
| Northern Mariana Islands | 0.709 | 4.31(0.00,266.11) | 2.058 | 2.249 | 55(50) | 0.772 | 4.97(0.00,75.37) | 2.058 | 2.914 | 42(40) |
| Norway | 0.796 | 78.28(43.00,123.63) | 2.057 | 76.218 | 203(203) | 0.916 | 72.49(41.13,111.69) | 2.057 | 70.435 | 197(197) |
| Oman | 0.429 | 2.63(0.03,12.39) | 2.075 | 0.557 | 12(9) | 0.773 | 4.31(0.72,11.40) | 2.058 | 2.250 | 30(28) |
| Pakistan | 0.310 | 17.71(9.96,27.46) | 2.091 | 15.617 | 149(149) | 0.504 | 17.31(10.11,26.09) | 2.058 | 15.256 | 143(143) |
| Palau | 0.663 | 3.96(0.00,322.69) | 2.058 | 1.904 | 50(45) | 0.754 | 5.24(0.00,150.90) | 2.057 | 3.188 | 46(44) |
| Palestine | 0.402 | 3.36(0.20,11.93) | 2.090 | 1.266 | 36(31) | 0.631 | 5.27(1.28,12.38) | 2.058 | 3.216 | 47(45) |
| Panama | 0.546 | 13.35(5.01,25.91) | 2.058 | 11.294 | 138(138) | 0.709 | 16.81(8.49,28.20) | 2.058 | 14.749 | 139(139) |
| Papua New Guinea | 0.311 | 2.59(0.26,8.04) | 2.093 | 0.494 | 11(7) | 0.418 | 2.90(0.72,6.65) | 2.091 | 0.810 | 5(5) |
| Paraguay | 0.470 | 6.55(1.62,15.28) | 2.057 | 4.496 | 86(83) | 0.636 | 9.24(3.39,18.33) | 2.060 | 7.182 | 85(84) |
| Peru | 0.510 | 14.92(8.06,23.84) | 2.057 | 12.864 | 144(144) | 0.662 | 22.67(12.42,35.90) | 2.061 | 20.607 | 151(151) |
| Philippines | 0.510 | 13.17(7.05,20.83) | 2.059 | 11.114 | 135(135) | 0.651 | 13.34(7.79,20.03) | 2.058 | 11.280 | 118(118) |
| Poland | 0.627 | 9.52(4.26,16.88) | 2.057 | 7.465 | 105(101) | 0.812 | 10.44(5.15,17.50) | 2.057 | 8.387 | 96(95) |
| Portugal | 0.600 | 19.92(11.44,30.14) | 2.057 | 17.866 | 157(157) | 0.744 | 27.12(16.42,40.02) | 2.058 | 25.063 | 159(159) |
| Puerto Rico | 0.659 | 18.09(9.33,30.22) | 2.058 | 16.036 | 152(152) | 0.826 | 23.61(13.07,37.79) | 2.057 | 21.557 | 154(154) |
| Qatar | 0.651 | 4.13(0.00,55.77) | 2.058 | 2.067 | 53(48) | 0.847 | 7.48(0.86,22.60) | 2.057 | 5.420 | 71(70) |
| Republic of Korea | 0.692 | 20.26(12.37,29.58) | 2.057 | 18.207 | 158(158) | 0.887 | 32.22(21.07,44.27) | 2.058 | 30.162 | 173(173) |
| Republic of Moldova | 0.604 | 3.29(0.79,7.60) | 2.060 | 1.235 | 34(29) | 0.732 | 4.42(1.38,9.29) | 2.059 | 2.361 | 34(32) |
| Romania | 0.619 | 4.74(2.09,8.47) | 2.059 | 2.683 | 59(54) | 0.768 | 6.95(3.34,11.83) | 2.057 | 4.895 | 63(62) |
| Russian Federation | 0.672 | 3.04(1.30,5.40) | 2.058 | 0.981 | 24(19) | 0.809 | 3.52(1.60,6.09) | 2.060 | 1.458 | 11(9) |
| Rwanda | 0.275 | 10.46(4.11,20.13) | 2.090 | 8.365 | 117(115) | 0.436 | 13.96(6.56,23.82) | 2.077 | 11.883 | 124(124) |
| Saint Kitts and Nevis | 0.581 | 20.65(0.20,118.55) | 2.062 | 18.591 | 159(159) | 0.755 | 21.31(0.76,92.61) | 2.058 | 19.249 | 149(149) |
| Saint Lucia | 0.496 | 22.56(2.34,79.40) | 2.057 | 20.501 | 164(164) | 0.673 | 27.72(8.72,62.43) | 2.059 | 25.662 | 162(162) |
| Saint Vincent and the Grenadines | 0.476 | 11.06(0.07,66.86) | 2.057 | 9.000 | 120(120) | 0.637 | 12.18(0.79,47.25) | 2.058 | 10.117 | 109(109) |
| Samoa | 0.487 | 3.45(0.00,47.09) | 2.058 | 1.396 | 38(33) | 0.593 | 4.37(0.00,34.31) | 2.058 | 2.309 | 31(29) |
| San Marino | 0.813 | 12.36(0.00,115.20) | 2.057 | 10.306 | 132(132) | 0.888 | 11.99(0.05,71.85) | 2.058 | 9.928 | 108(108) |
| Sao Tome and Principe | 0.310 | 6.33(0.00,63.28) | 2.090 | 4.235 | 81(80) | 0.505 | 8.67(0.03,52.80) | 2.057 | 6.613 | 81(80) |
| Saudi Arabia | 0.539 | 2.62(0.61,6.18) | 2.057 | 0.563 | 13(8) | 0.815 | 4.30(1.69,7.99) | 2.058 | 2.238 | 28(26) |
| Senegal | 0.238 | 6.47(2.16,13.09) | 2.091 | 4.383 | 83(82) | 0.408 | 8.04(3.60,14.09) | 2.091 | 5.952 | 76(75) |
| Serbia | 0.631 | 9.86(4.59,17.27) | 2.057 | 7.806 | 110(107) | 0.792 | 11.02(5.09,19.15) | 2.058 | 8.966 | 100(100) |
| Seychelles | 0.576 | 3.61(0.00,61.85) | 2.058 | 1.549 | 43(38) | 0.730 | 4.95(0.00,39.91) | 2.058 | 2.890 | 40(38) |
| Sierra Leone | 0.212 | 5.80(1.55,13.08) | 2.091 | 3.712 | 76(74) | 0.359 | 6.65(2.36,12.99) | 2.090 | 4.563 | 61(60) |
| Singapore | 0.686 | 10.80(4.02,21.38) | 2.057 | 8.746 | 118(116) | 0.856 | 14.07(7.23,23.01) | 2.057 | 12.015 | 125(125) |
| Slovakia | 0.654 | 4.95(1.67,9.99) | 2.057 | 2.891 | 63(58) | 0.811 | 6.97(2.93,12.89) | 2.057 | 4.917 | 64(63) |
| Slovenia | 0.727 | 18.41(6.35,34.71) | 2.057 | 16.357 | 153(153) | 0.842 | 23.10(10.08,40.49) | 2.058 | 21.044 | 153(153) |
| Solomon Islands | 0.301 | 2.76(0.00,33.07) | 2.091 | 0.671 | 15(11) | 0.429 | 3.29(0.01,19.27) | 2.076 | 1.213 | 9(7) |
| Somalia | 0.049 | 9.95(3.40,20.44) | 9.946 | 0.000 | 3(110) | 0.078 | 10.48(4.74,18.42) | 9.550 | 0.930 | 7(96) |
| South Africa | 0.542 | 18.82(9.63,31.20) | 2.058 | 16.765 | 155(155) | 0.680 | 19.28(10.44,30.85) | 2.057 | 17.223 | 148(148) |
| South Sudan | 0.207 | 9.75(3.71,18.90) | 2.090 | 7.660 | 109(106) | 0.278 | 11.17(4.65,20.47) | 2.091 | 9.080 | 102(102) |
| Spain | 0.637 | 26.65(18.38,35.53) | 2.058 | 24.591 | 177(177) | 0.769 | 33.31(25.90,41.91) | 2.058 | 31.247 | 176(176) |
| Sri Lanka | 0.523 | 3.62(1.37,7.00) | 2.059 | 1.560 | 44(39) | 0.702 | 4.98(2.39,8.40) | 2.058 | 2.927 | 43(41) |
| Sudan | 0.292 | 2.19(0.60,4.82) | 2.090 | 0.096 | 5(2) | 0.542 | 3.08(1.18,5.80) | 2.057 | 1.025 | 8(6) |
| Suriname | 0.502 | 8.07(0.56,30.31) | 2.057 | 6.009 | 99(96) | 0.634 | 9.68(2.40,23.58) | 2.057 | 7.628 | 91(90) |
| Sweden | 0.786 | 73.65(46.35,108.31) | 2.060 | 71.594 | 200(200) | 0.887 | 67.58(43.55,96.07) | 2.058 | 65.520 | 195(195) |
| Switzerland | 0.863 | 57.35(34.56,85.20) | 2.057 | 55.297 | 194(194) | 0.933 | 60.40(37.11,87.58) | 2.058 | 58.339 | 194(194) |
| Syrian Arab Republic | 0.430 | 2.93(0.71,6.75) | 2.075 | 0.858 | 20(16) | 0.623 | 4.49(1.74,8.49) | 2.057 | 2.437 | 35(33) |
| Taiwan (Province of China) | 0.668 | 24.22(13.71,36.81) | 2.059 | 22.165 | 170(170) | 0.875 | 27.24(19.65,35.45) | 2.058 | 25.184 | 160(160) |
| Tajikistan | 0.466 | 9.67(3.75,18.81) | 2.057 | 7.613 | 108(105) | 0.542 | 13.15(6.40,22.87) | 2.058 | 11.095 | 115(115) |
| Thailand | 0.507 | 6.10(2.93,10.24) | 2.058 | 4.045 | 79(77) | 0.683 | 8.16(4.30,13.23) | 2.057 | 6.104 | 78(77) |
| Timor-Leste | 0.262 | 5.29(0.05,27.29) | 2.092 | 3.195 | 66(61) | 0.445 | 8.80(2.06,21.39) | 2.058 | 6.746 | 82(81) |
| Togo | 0.270 | 6.58(1.28,16.40) | 2.090 | 4.491 | 85(85) | 0.409 | 8.01(3.00,15.24) | 2.090 | 5.917 | 75(74) |
| Tokelau | 0.522 | 3.00(0.00,1942.93) | 2.058 | 0.943 | 22(18) | 0.686 | 3.88(0.00,1924.54) | 2.058 | 1.825 | 16(14) |
| Tonga | 0.492 | 3.26(0.00,66.18) | 2.058 | 1.197 | 33(28) | 0.626 | 3.81(0.00,49.99) | 2.057 | 1.756 | 15(13) |
| Trinidad and Tobago | 0.624 | 24.49(11.56,44.13) | 2.058 | 22.433 | 171(171) | 0.769 | 27.61(15.80,43.80) | 2.058 | 25.555 | 161(161) |
| Tunisia | 0.471 | 2.80(0.67,6.50) | 2.057 | 0.741 | 18(13) | 0.682 | 3.98(1.54,7.62) | 2.060 | 1.918 | 18(16) |
| Turkey | 0.462 | 5.76(2.57,10.04) | 2.058 | 3.704 | 75(73) | 0.713 | 9.14(4.49,15.06) | 2.057 | 7.082 | 84(83) |
| Turkmenistan | 0.563 | 3.46(0.58,9.36) | 2.064 | 1.399 | 39(34) | 0.682 | 4.27(1.23,9.35) | 2.057 | 2.210 | 26(24) |
| Tuvalu | 0.406 | 3.08(0.00,450.57) | 2.090 | 0.989 | 25(21) | 0.577 | 4.00(0.00,292.48) | 2.058 | 1.942 | 19(17) |
| Uganda | 0.187 | 10.85(4.99,18.61) | 2.091 | 8.764 | 119(118) | 0.423 | 13.32(6.90,21.56) | 2.091 | 11.233 | 117(117) |
| Ukraine | 0.647 | 3.32(1.43,5.96) | 2.057 | 1.265 | 35(30) | 0.761 | 3.95(1.79,6.88) | 2.057 | 1.897 | 17(15) |
| United Arab Emirates | 0.644 | 3.47(0.01,20.41) | 2.057 | 1.417 | 40(35) | 0.849 | 5.17(1.30,11.88) | 2.057 | 3.114 | 45(43) |
| United Kingdom | 0.744 | 67.55(42.72,97.01) | 2.057 | 65.491 | 199(199) | 0.859 | 73.33(47.35,104.20) | 2.059 | 71.271 | 198(198) |
| United Republic of Tanzania | 0.259 | 11.85(6.02,19.81) | 2.092 | 9.759 | 126(126) | 0.447 | 14.20(7.75,22.33) | 2.065 | 12.137 | 126(126) |
| United States Virgin Islands | 0.655 | 14.06(0.25,71.01) | 2.058 | 12.004 | 143(143) | 0.822 | 19.19(3.42,53.56) | 2.057 | 17.135 | 146(146) |
| United States of America | 0.764 | 39.78(25.18,56.59) | 2.057 | 37.718 | 190(190) | 0.862 | 47.65(30.95,66.31) | 2.057 | 45.597 | 190(190) |
| Uruguay | 0.582 | 26.86(15.98,40.81) | 2.057 | 24.806 | 178(178) | 0.719 | 31.87(19.17,48.01) | 2.057 | 29.816 | 172(172) |
| Uzbekistan | 0.500 | 7.61(3.21,13.90) | 2.059 | 5.553 | 94(93) | 0.663 | 9.28(4.36,16.28) | 2.057 | 7.226 | 87(86) |
| Vanuatu | 0.353 | 3.06(0.00,62.73) | 2.092 | 0.967 | 23(20) | 0.473 | 3.65(0.00,29.73) | 2.059 | 1.590 | 14(12) |
| Venezuela (Bolivarian Republic of) | 0.517 | 23.71(13.92,36.84) | 2.057 | 21.656 | 169(169) | 0.597 | 29.02(17.63,43.85) | 2.058 | 26.963 | 165(165) |
| Viet Nam | 0.408 | 7.56(3.87,12.38) | 2.091 | 5.473 | 93(92) | 0.628 | 15.45(8.63,23.86) | 2.057 | 13.394 | 133(133) |
| Yemen | 0.216 | 2.24(0.45,5.55) | 2.092 | 0.151 | 6(4) | 0.450 | 2.82(1.00,5.52) | 2.062 | 0.762 | 4(4) |
| Zambia | 0.304 | 7.84(2.75,15.70) | 2.092 | 5.747 | 97(95) | 0.506 | 9.78(4.39,17.34) | 2.058 | 7.719 | 93(92) |
| Zimbabwe | 0.399 | 7.25(3.00,13.52) | 2.091 | 5.154 | 92(91) | 0.474 | 7.06(3.18,12.58) | 2.060 | 5.001 | 66(64) |

**Abbreviations: ASR, age-standardised rate;** **SDI: sociodemographic index**

**Table S7. Age-standardised prevalence rates of elderly-onset rheumatoid arthritis with frontier analysis across 204 countries and territories in 1990 and 2021.**

| **Location** | **Prevalence, 1990** | | | | | **Prevalence, 2021** | | | | |
| --- | --- | --- | --- | --- | --- | --- | --- | --- | --- | --- |
|  | **SDI** | **ASRs** | **Frontier** | **Effective difference** | **Effective difference rank (ASRs rank)** | **SDI** | **ASRs** | **Frontier** | **Effective difference** | **Effective difference rank (ASRs rank)** |
| Afghanistan | 0.174 | 98.01(78.20,122.33) | 98.008 | 0.000 | 1(6) | 0.337 | 141.85(117.02,170.67) | 90.744 | 51.110 | 9(8) |
| Albania | 0.558 | 254.35(194.58,325.73) | 84.421 | 169.932 | 104(98) | 0.707 | 441.52(359.87,536.31) | 84.412 | 357.105 | 124(124) |
| Algeria | 0.460 | 116.16(92.68,144.74) | 84.414 | 31.750 | 23(18) | 0.660 | 179.54(148.54,216.02) | 84.414 | 95.125 | 32(29) |
| American Samoa | 0.614 | 147.61(26.41,432.70) | 84.417 | 63.190 | 57(52) | 0.724 | 181.24(70.34,369.16) | 84.414 | 96.828 | 33(30) |
| Andorra | 0.761 | 392.94(213.90,643.39) | 84.412 | 308.530 | 139(139) | 0.869 | 425.47(282.15,608.76) | 84.414 | 341.055 | 120(120) |
| Angola | 0.271 | 269.52(220.79,325.04) | 94.217 | 175.301 | 107(108) | 0.454 | 361.75(307.09,422.64) | 84.421 | 277.334 | 107(107) |
| Antigua and Barbuda | 0.612 | 357.29(195.17,588.29) | 84.415 | 272.877 | 127(126) | 0.750 | 456.92(299.08,660.26) | 84.412 | 372.503 | 126(126) |
| Argentina | 0.587 | 407.54(347.92,474.92) | 84.415 | 323.129 | 144(144) | 0.723 | 580.05(511.24,656.05) | 84.413 | 495.640 | 141(141) |
| Armenia | 0.544 | 178.06(136.63,228.90) | 84.418 | 93.638 | 69(68) | 0.702 | 271.41(222.11,328.58) | 84.412 | 187.000 | 78(78) |
| Australia | 0.726 | 1320.19(1146.77,1505.65) | 84.412 | 1235.777 | 197(197) | 0.844 | 1470.98(1284.17,1675.84) | 84.412 | 1386.563 | 196(196) |
| Austria | 0.750 | 913.45(796.52,1048.83) | 84.412 | 829.038 | 189(189) | 0.854 | 871.84(757.47,1000.27) | 84.412 | 787.426 | 174(174) |
| Azerbaijan | 0.596 | 146.65(112.98,188.39) | 84.415 | 62.238 | 55(50) | 0.695 | 189.58(152.64,234.32) | 84.412 | 105.167 | 38(35) |
| Bahamas | 0.694 | 340.65(222.03,491.14) | 84.414 | 256.237 | 124(124) | 0.805 | 426.16(321.67,552.39) | 84.412 | 341.749 | 121(121) |
| Bahrain | 0.585 | 322.58(210.35,468.29) | 84.412 | 238.172 | 119(119) | 0.753 | 600.69(489.98,725.92) | 84.414 | 516.281 | 143(143) |
| Bangladesh | 0.229 | 224.14(186.37,266.66) | 96.254 | 127.882 | 85(85) | 0.492 | 368.20(314.24,432.30) | 84.412 | 283.786 | 108(108) |
| Barbados | 0.654 | 565.34(430.64,722.28) | 84.412 | 480.933 | 161(161) | 0.747 | 738.21(598.75,904.98) | 84.412 | 653.796 | 161(161) |
| Belarus | 0.622 | 207.83(168.01,252.76) | 84.417 | 123.414 | 81(79) | 0.784 | 336.98(282.70,396.49) | 84.413 | 252.566 | 100(100) |
| Belgium | 0.737 | 831.12(714.57,960.85) | 84.412 | 746.704 | 187(187) | 0.854 | 895.20(777.19,1034.53) | 84.413 | 810.782 | 176(176) |
| Belize | 0.424 | 313.19(186.69,484.63) | 84.474 | 228.712 | 116(116) | 0.610 | 401.65(294.33,532.33) | 84.412 | 317.243 | 115(115) |
| Benin | 0.219 | 143.27(106.39,187.85) | 96.601 | 46.671 | 40(46) | 0.373 | 182.41(144.79,226.27) | 90.119 | 92.291 | 28(31) |
| Bermuda | 0.696 | 404.08(229.76,648.75) | 84.412 | 319.669 | 143(143) | 0.821 | 602.94(424.07,826.91) | 84.414 | 518.521 | 144(144) |
| Bhutan | 0.215 | 428.95(295.70,600.21) | 97.136 | 331.814 | 145(145) | 0.473 | 742.67(580.47,937.69) | 84.414 | 658.257 | 162(162) |
| Bolivia (Plurinational State of) | 0.424 | 539.04(451.59,638.40) | 84.492 | 454.544 | 159(159) | 0.599 | 935.55(813.29,1078.24) | 84.414 | 851.136 | 180(180) |
| Bosnia and Herzegovina | 0.541 | 496.56(411.74,594.76) | 84.415 | 412.146 | 153(152) | 0.723 | 749.87(643.12,878.57) | 84.412 | 665.461 | 165(165) |
| Botswana | 0.418 | 362.53(276.63,463.77) | 85.230 | 277.301 | 132(131) | 0.643 | 492.86(405.34,592.48) | 84.413 | 408.445 | 131(131) |
| Brazil | 0.500 | 316.83(268.94,371.19) | 84.412 | 232.418 | 118(118) | 0.653 | 310.21(267.68,359.89) | 84.415 | 225.795 | 89(89) |
| Brunei Darussalam | 0.666 | 617.53(410.52,885.38) | 84.413 | 533.116 | 169(169) | 0.810 | 825.03(646.95,1035.92) | 84.414 | 740.615 | 171(171) |
| Bulgaria | 0.633 | 239.26(197.27,290.36) | 84.412 | 154.853 | 98(92) | 0.768 | 310.35(260.43,368.87) | 84.412 | 225.938 | 90(90) |
| Burkina Faso | 0.130 | 141.77(108.96,181.30) | #### | 24.207 | 18(45) | 0.285 | 169.79(135.61,209.35) | 93.870 | 75.920 | 21(25) |
| Burundi | 0.206 | 235.23(187.32,289.93) | 97.138 | 138.089 | 89(89) | 0.289 | 259.72(213.09,315.36) | 93.948 | 165.770 | 74(75) |
| Cabo Verde | 0.277 | 141.44(83.08,220.67) | 93.880 | 47.559 | 41(44) | 0.534 | 212.92(145.56,297.41) | 84.430 | 128.488 | 53(52) |
| Cambodia | 0.289 | 135.91(106.52,170.09) | 93.932 | 41.982 | 31(34) | 0.474 | 241.01(198.33,288.55) | 84.419 | 156.587 | 69(68) |
| Cameroon | 0.303 | 167.91(130.56,211.02) | 93.930 | 73.983 | 63(63) | 0.480 | 206.50(168.39,250.63) | 84.412 | 122.091 | 49(48) |
| Canada | 0.782 | 912.37(853.29,972.15) | 84.412 | 827.957 | 188(188) | 0.873 | 1204.27(1133.28,1280.83) | 84.412 | 1119.861 | 191(191) |
| Central African Republic | 0.217 | 294.95(233.01,366.85) | 96.929 | 198.021 | 112(115) | 0.309 | 303.87(247.75,369.01) | 93.941 | 209.927 | 82(86) |
| Chad | 0.115 | 125.76(94.26,162.51) | #### | 7.939 | 9(22) | 0.240 | 138.14(107.94,172.85) | 95.008 | 43.132 | 7(6) |
| Chile | 0.586 | 711.68(611.28,825.42) | 84.413 | 627.263 | 181(181) | 0.772 | 1303.19(1140.67,1485.46) | 84.412 | 1218.780 | 193(193) |
| China | 0.459 | 643.85(542.55,765.65) | 84.416 | 559.437 | 172(172) | 0.722 | 747.94(638.04,877.84) | 84.412 | 663.532 | 164(164) |
| Colombia | 0.481 | 442.85(383.31,510.74) | 84.433 | 358.413 | 149(149) | 0.655 | 672.04(596.29,756.62) | 84.412 | 587.628 | 152(152) |
| Comoros | 0.270 | 257.31(163.26,380.69) | 94.348 | 162.960 | 100(102) | 0.476 | 321.21(235.34,426.51) | 84.412 | 236.801 | 97(97) |
| Congo | 0.421 | 364.50(288.74,452.41) | 84.936 | 279.562 | 133(133) | 0.583 | 469.57(393.27,558.08) | 84.413 | 385.152 | 130(130) |
| Cook Islands | 0.565 | 109.16(4.54,489.17) | 84.412 | 24.749 | 19(10) | 0.779 | 150.46(35.76,392.17) | 84.415 | 66.048 | 16(14) |
| Costa Rica | 0.534 | 691.22(582.97,817.60) | 84.416 | 606.807 | 179(179) | 0.700 | 1155.11(1013.13,1319.55) | 84.412 | 1070.701 | 189(189) |
| Côte d'Ivoire | 0.279 | 151.80(115.04,197.09) | 93.871 | 57.931 | 52(56) | 0.426 | 193.40(156.37,236.13) | 84.420 | 108.977 | 41(40) |
| Croatia | 0.669 | 571.27(485.79,669.00) | 84.414 | 486.855 | 164(163) | 0.798 | 918.74(798.81,1055.95) | 84.414 | 834.324 | 178(178) |
| Cuba | 0.558 | 386.89(328.97,455.12) | 84.420 | 302.473 | 137(137) | 0.669 | 557.94(484.05,641.43) | 84.414 | 473.526 | 139(139) |
| Cyprus | 0.648 | 1252.00(1021.71,1522.12) | 84.412 | 1167.590 | 194(194) | 0.836 | 1589.88(1335.39,1869.09) | 84.412 | 1505.470 | 197(197) |
| Czechia | 0.682 | 323.48(271.88,381.98) | 84.414 | 239.063 | 120(120) | 0.828 | 455.21(391.58,528.55) | 84.413 | 370.802 | 125(125) |
| Democratic People's Republic of Korea | 0.498 | 535.44(458.60,623.02) | 84.445 | 450.996 | 157(157) | 0.570 | 721.62(634.07,823.82) | 84.421 | 637.202 | 159(159) |
| Democratic Republic of the Congo | 0.290 | 263.73(222.17,310.36) | 93.878 | 169.853 | 103(106) | 0.383 | 320.15(273.97,374.48) | 90.176 | 229.969 | 95(95) |
| Denmark | 0.801 | 1223.05(1065.97,1395.45) | 84.412 | 1138.634 | 193(193) | 0.896 | 1659.36(1453.04,1889.95) | 84.413 | 1574.949 | 201(201) |
| Djibouti | 0.338 | 245.42(139.19,395.34) | 90.747 | 154.673 | 97(96) | 0.488 | 313.04(230.26,411.31) | 84.414 | 228.628 | 92(92) |
| Dominica | 0.564 | 328.04(180.47,540.15) | 84.412 | 243.632 | 122(122) | 0.747 | 375.26(229.23,570.39) | 84.412 | 290.848 | 110(110) |
| Dominican Republic | 0.443 | 227.21(181.81,280.48) | 84.414 | 142.800 | 91(88) | 0.619 | 314.05(261.32,376.73) | 84.412 | 229.639 | 94(94) |
| Ecuador | 0.518 | 634.44(541.59,736.70) | 84.412 | 550.032 | 170(170) | 0.661 | 1085.68(954.53,1233.90) | 84.412 | 1001.264 | 187(187) |
| Egypt | 0.417 | 135.13(111.32,163.48) | 84.789 | 50.338 | 45(33) | 0.607 | 224.39(191.40,263.37) | 84.413 | 139.976 | 60(59) |
| El Salvador | 0.373 | 240.88(193.25,296.48) | 90.201 | 150.677 | 95(93) | 0.564 | 394.52(329.72,469.05) | 84.413 | 310.110 | 114(114) |
| Equatorial Guinea | 0.269 | 272.59(177.76,397.57) | 93.933 | 178.658 | 108(110) | 0.658 | 522.67(400.23,669.25) | 84.413 | 438.261 | 134(134) |
| Eritrea | 0.216 | 245.10(186.38,316.08) | 96.989 | 148.108 | 94(95) | 0.404 | 302.90(245.88,369.95) | 90.158 | 212.744 | 84(84) |
| Estonia | 0.675 | 775.72(681.92,878.59) | 84.412 | 691.312 | 184(184) | 0.845 | 963.52(860.70,1078.23) | 84.413 | 879.105 | 183(183) |
| Eswatini | 0.399 | 439.56(319.84,583.00) | 90.118 | 349.442 | 147(148) | 0.585 | 513.61(405.40,639.92) | 84.417 | 429.197 | 132(132) |
| Ethiopia | 0.148 | 269.81(220.19,329.73) | #### | 152.532 | 96(109) | 0.359 | 250.13(208.43,300.79) | 90.487 | 159.638 | 71(71) |
| Fiji | 0.535 | 88.83(47.35,149.41) | 84.413 | 4.419 | 8(2) | 0.675 | 106.77(70.57,154.98) | 84.412 | 22.355 | 4(3) |
| Finland | 0.756 | 1515.25(1330.26,1727.30) | 84.412 | 1430.838 | 198(198) | 0.860 | 1901.17(1675.25,2147.69) | 84.412 | 1816.763 | 203(203) |
| France | 0.731 | 708.75(610.63,820.55) | 84.413 | 624.335 | 180(180) | 0.838 | 853.65(738.28,987.23) | 84.414 | 769.237 | 173(173) |
| Gabon | 0.455 | 383.71(293.32,489.79) | 84.412 | 299.300 | 135(135) | 0.635 | 552.28(449.69,669.11) | 84.413 | 467.867 | 137(137) |
| Gambia | 0.239 | 148.00(90.22,224.92) | 94.589 | 53.408 | 47(53) | 0.410 | 188.26(136.22,251.76) | 90.163 | 98.099 | 34(34) |
| Georgia | 0.740 | 188.55(149.15,237.87) | 84.412 | 104.138 | 72(70) | 0.847 | 220.94(179.17,269.11) | 84.416 | 136.522 | 59(58) |
| Germany | 0.817 | 659.89(569.80,760.68) | 84.412 | 575.482 | 174(174) | 0.903 | 737.05(639.31,842.21) | 84.412 | 652.634 | 160(160) |
| Ghana | 0.373 | 136.28(102.65,175.73) | 90.120 | 46.163 | 38(36) | 0.565 | 193.61(153.67,239.29) | 84.438 | 109.175 | 42(41) |
| Greece | 0.674 | 526.16(443.68,622.00) | 84.412 | 441.743 | 156(156) | 0.792 | 591.35(508.64,684.99) | 84.412 | 506.934 | 142(142) |
| Greenland | 0.732 | 436.52(207.06,791.13) | 84.414 | 352.110 | 148(147) | 0.826 | 744.10(500.89,1058.03) | 84.415 | 659.689 | 163(163) |
| Grenada | 0.437 | 194.88(94.26,346.68) | 84.412 | 110.466 | 75(71) | 0.669 | 288.36(171.94,446.06) | 84.414 | 203.942 | 81(80) |
| Guam | 0.676 | 133.21(52.33,269.60) | 84.414 | 48.796 | 43(30) | 0.804 | 172.35(104.79,262.95) | 84.425 | 87.922 | 27(26) |
| Guatemala | 0.312 | 293.28(240.31,353.20) | 91.377 | 201.900 | 114(114) | 0.540 | 569.93(497.43,653.17) | 84.418 | 485.512 | 140(140) |
| Guinea | 0.178 | 136.32(103.51,174.95) | 97.156 | 39.161 | 28(37) | 0.336 | 158.12(125.48,197.85) | 90.731 | 67.390 | 17(16) |
| Guinea-Bissau | 0.208 | 136.70(84.75,204.43) | 97.172 | 39.523 | 29(38) | 0.353 | 169.39(118.18,232.97) | 90.739 | 78.648 | 24(24) |
| Guyana | 0.460 | 154.72(99.22,228.56) | 84.413 | 70.309 | 60(57) | 0.651 | 212.97(154.33,286.19) | 84.414 | 128.551 | 54(53) |
| Haiti | 0.310 | 197.07(160.21,241.43) | 93.883 | 103.186 | 71(73) | 0.448 | 252.98(212.51,298.34) | 84.420 | 168.558 | 75(72) |
| Honduras | 0.332 | 657.18(554.11,770.68) | 90.730 | 566.451 | 173(173) | 0.513 | 1075.70(945.34,1224.13) | 84.430 | 991.267 | 185(185) |
| Hungary | 0.649 | 569.31(504.06,642.31) | 84.414 | 484.896 | 163(162) | 0.791 | 706.84(628.98,794.03) | 84.412 | 622.430 | 154(154) |
| Iceland | 0.764 | 769.45(586.94,984.20) | 84.412 | 685.037 | 183(183) | 0.876 | 890.44(704.78,1099.77) | 84.412 | 806.027 | 175(175) |
| India | 0.333 | 573.51(469.07,702.57) | 90.856 | 482.658 | 162(164) | 0.575 | 904.18(753.44,1086.44) | 84.441 | 819.737 | 177(177) |
| Indonesia | 0.457 | 100.44(80.12,124.78) | 84.413 | 16.023 | 11(7) | 0.657 | 131.10(107.50,158.76) | 84.414 | 46.689 | 8(5) |
| Iran (Islamic Republic of) | 0.454 | 140.06(114.44,170.64) | 84.439 | 55.625 | 50(42) | 0.697 | 195.05(165.01,231.46) | 84.412 | 110.640 | 43(42) |
| Iraq | 0.412 | 146.32(116.27,182.01) | 88.951 | 57.365 | 51(49) | 0.663 | 228.43(191.44,271.72) | 84.412 | 144.013 | 62(61) |
| Ireland | 0.720 | 1932.62(1685.38,2208.71) | 84.413 | 1848.206 | 203(203) | 0.874 | 2364.72(2060.97,2686.03) | 84.412 | 2280.306 | 204(204) |
| Israel | 0.709 | 486.87(398.10,585.56) | 84.412 | 402.455 | 151(151) | 0.809 | 658.38(554.17,776.15) | 84.412 | 573.968 | 150(150) |
| Italy | 0.706 | 936.48(778.40,1124.91) | 84.413 | 852.069 | 190(190) | 0.806 | 919.83(774.26,1095.58) | 84.412 | 835.420 | 179(179) |
| Jamaica | 0.535 | 263.55(209.70,328.78) | 84.414 | 179.138 | 109(105) | 0.683 | 356.88(295.24,428.14) | 84.413 | 272.465 | 104(104) |
| Japan | 0.790 | 1319.78(1103.98,1580.23) | 84.412 | 1235.366 | 196(196) | 0.871 | 1068.23(912.47,1252.45) | 84.415 | 983.819 | 184(184) |
| Jordan | 0.539 | 145.57(106.72,194.73) | 84.418 | 61.154 | 54(48) | 0.725 | 230.49(188.31,281.11) | 84.412 | 146.082 | 64(63) |
| Kazakhstan | 0.589 | 174.84(140.50,215.94) | 84.412 | 90.429 | 68(67) | 0.725 | 245.30(202.24,294.63) | 84.412 | 160.890 | 72(69) |
| Kenya | 0.334 | 273.04(218.37,340.23) | 90.742 | 182.302 | 111(111) | 0.524 | 301.43(248.77,365.20) | 84.419 | 217.009 | 85(83) |
| Kiribati | 0.410 | 85.31(14.17,255.62) | 85.310 | 0.000 | 2(1) | 0.527 | 101.60(34.87,222.36) | 84.412 | 17.193 | 2(1) |
| Kuwait | 0.665 | 254.72(181.29,346.66) | 84.413 | 170.308 | 105(99) | 0.847 | 376.59(307.46,453.29) | 84.412 | 292.180 | 112(112) |
| Kyrgyzstan | 0.519 | 640.56(541.06,752.02) | 84.426 | 556.130 | 171(171) | 0.604 | 962.74(819.08,1121.38) | 84.412 | 878.325 | 182(182) |
| Lao People's Democratic Republic | 0.264 | 133.40(101.23,171.54) | 94.405 | 38.999 | 27(31) | 0.489 | 217.61(174.72,266.46) | 84.421 | 133.186 | 57(56) |
| Latvia | 0.680 | 560.83(498.54,631.16) | 84.415 | 476.411 | 160(160) | 0.831 | 664.91(592.75,743.29) | 84.415 | 580.499 | 151(151) |
| Lebanon | 0.537 | 197.80(154.25,252.04) | 84.421 | 113.381 | 76(74) | 0.745 | 283.92(233.23,341.02) | 84.414 | 199.502 | 79(79) |
| Lesotho | 0.339 | 364.26(285.67,453.31) | 90.767 | 273.495 | 128(132) | 0.510 | 436.43(354.61,530.30) | 84.413 | 352.022 | 122(122) |
| Liberia | 0.235 | 141.44(100.32,191.47) | 94.977 | 46.462 | 39(43) | 0.352 | 185.03(140.33,237.61) | 90.751 | 94.278 | 31(33) |
| Libya | 0.528 | 133.48(99.38,177.28) | 84.413 | 49.071 | 44(32) | 0.726 | 192.06(154.09,236.09) | 84.415 | 107.641 | 40(39) |
| Lithuania | 0.669 | 661.19(590.25,739.28) | 84.413 | 576.780 | 175(175) | 0.856 | 786.91(709.31,872.38) | 84.412 | 702.496 | 168(168) |
| Luxembourg | 0.781 | 675.01(532.49,844.98) | 84.413 | 590.599 | 178(178) | 0.884 | 779.32(634.64,946.65) | 84.414 | 694.906 | 167(167) |
| Madagascar | 0.280 | 202.53(162.58,249.11) | 93.914 | 108.614 | 74(77) | 0.400 | 248.14(207.62,297.48) | 90.125 | 158.011 | 70(70) |
| Malawi | 0.204 | 253.70(204.74,312.79) | 97.158 | 156.539 | 99(97) | 0.385 | 321.06(266.90,383.33) | 90.146 | 230.912 | 96(96) |
| Malaysia | 0.546 | 104.82(79.76,134.66) | 84.412 | 20.412 | 14(8) | 0.743 | 146.33(118.68,179.75) | 84.414 | 61.913 | 13(11) |
| Maldives | 0.332 | 197.02(98.94,342.04) | 90.736 | 106.280 | 73(72) | 0.651 | 339.91(242.37,460.09) | 84.412 | 255.497 | 101(101) |
| Mali | 0.127 | 126.93(95.75,163.83) | #### | 9.384 | 10(23) | 0.269 | 152.70(121.94,188.11) | 93.900 | 58.805 | 11(15) |
| Malta | 0.657 | 605.23(465.12,765.58) | 84.412 | 520.819 | 168(168) | 0.802 | 711.20(577.38,864.74) | 84.413 | 626.788 | 157(157) |
| Marshall Islands | 0.431 | 122.41(12.22,444.71) | 84.421 | 37.986 | 26(21) | 0.574 | 162.60(48.40,380.46) | 84.419 | 78.180 | 23(20) |
| Mauritania | 0.336 | 161.47(115.97,217.07) | 90.917 | 70.555 | 61(61) | 0.499 | 232.47(180.34,292.92) | 84.412 | 148.056 | 65(64) |
| Mauritius | 0.545 | 199.86(139.89,276.42) | 84.418 | 115.442 | 77(75) | 0.718 | 305.32(238.81,387.13) | 84.412 | 220.904 | 88(88) |
| Mexico | 0.505 | 1260.31(1059.38,1488.60) | 84.432 | 1175.881 | 195(195) | 0.665 | 1330.96(1150.43,1543.68) | 84.414 | 1246.542 | 194(194) |
| Micronesia (Federated States of) | 0.463 | 128.43(42.47,285.61) | 84.418 | 44.007 | 34(25) | 0.588 | 191.86(93.08,341.79) | 84.412 | 107.451 | 39(37) |
| Monaco | 0.845 | 385.87(219.21,621.11) | 84.412 | 301.456 | 136(136) | 0.908 | 412.52(252.71,622.27) | 84.414 | 328.102 | 118(118) |
| Mongolia | 0.467 | 220.86(166.94,284.41) | 84.412 | 136.447 | 87(83) | 0.618 | 356.86(288.41,434.91) | 84.412 | 272.449 | 103(103) |
| Montenegro | 0.674 | 360.27(267.18,471.27) | 84.412 | 275.859 | 131(130) | 0.796 | 458.51(358.33,575.63) | 84.413 | 374.094 | 127(127) |
| Morocco | 0.358 | 113.78(91.89,140.21) | 90.662 | 23.113 | 16(14) | 0.563 | 177.82(149.78,210.95) | 84.418 | 93.397 | 29(27) |
| Mozambique | 0.173 | 236.24(192.34,288.64) | #### | 119.194 | 79(90) | 0.326 | 293.35(244.63,348.02) | 90.736 | 202.614 | 80(81) |
| Myanmar | 0.319 | 139.18(113.75,168.51) | 90.733 | 48.447 | 42(41) | 0.534 | 236.72(199.35,279.65) | 84.412 | 152.306 | 67(66) |
| Namibia | 0.450 | 342.34(262.83,438.33) | 84.430 | 257.908 | 125(125) | 0.618 | 460.06(374.25,560.82) | 84.412 | 375.648 | 128(128) |
| Nauru | 0.539 | 144.70(0.42,1060.32) | 84.418 | 60.279 | 53(47) | 0.625 | 203.74(6.32,1005.27) | 84.412 | 119.333 | 48(47) |
| Nepal | 0.200 | 359.26(295.16,435.07) | 97.141 | 262.115 | 126(128) | 0.433 | 625.91(529.48,739.65) | 84.412 | 541.499 | 146(146) |
| Netherlands | 0.795 | 1627.88(1416.91,1864.32) | 84.412 | 1543.466 | 201(201) | 0.888 | 1598.52(1382.65,1837.57) | 84.416 | 1514.101 | 198(198) |
| New Zealand | 0.752 | 1812.70(1473.73,2206.96) | 84.412 | 1728.285 | 202(202) | 0.849 | 1651.24(1372.68,1991.15) | 84.412 | 1566.824 | 199(199) |
| Nicaragua | 0.346 | 501.33(409.69,605.94) | 90.870 | 410.461 | 152(154) | 0.524 | 775.33(664.00,908.21) | 84.438 | 690.890 | 166(166) |
| Niger | 0.081 | 121.55(89.29,159.83) | #### | 0.000 | 3(20) | 0.168 | 144.62(114.69,179.99) | ##### | 27.586 | 5(10) |
| Nigeria | 0.306 | 136.01(108.93,167.77) | 93.929 | 42.084 | 32(35) | 0.503 | 161.90(133.20,195.50) | 84.437 | 77.458 | 22(19) |
| Niue | 0.588 | 129.42(0.00,1626.57) | 84.415 | 45.007 | 36(27) | 0.726 | 183.05(0.06,1724.39) | 84.412 | 98.641 | 35(32) |
| North Macedonia | 0.609 | 225.55(171.23,292.92) | 84.412 | 141.142 | 90(86) | 0.751 | 335.99(267.81,414.34) | 84.412 | 251.583 | 99(99) |
| Northern Mariana Islands | 0.709 | 169.96(19.01,593.67) | 84.413 | 85.549 | 66(65) | 0.772 | 197.15(81.77,384.99) | 84.412 | 112.734 | 44(43) |
| Norway | 0.796 | 2051.10(1685.33,2492.19) | 84.414 | 1966.684 | 204(204) | 0.916 | 1655.38(1378.11,1998.33) | 84.414 | 1570.966 | 200(200) |
| Oman | 0.429 | 115.25(74.16,169.09) | 84.420 | 30.831 | 22(17) | 0.773 | 207.18(156.54,267.90) | 84.415 | 122.763 | 50(49) |
| Pakistan | 0.310 | 432.31(363.68,512.74) | 93.872 | 338.434 | 146(146) | 0.504 | 406.20(343.20,480.57) | 84.414 | 321.791 | 117(117) |
| Palau | 0.663 | 157.44(11.10,632.21) | 84.415 | 73.021 | 62(58) | 0.754 | 214.59(59.95,515.15) | 84.412 | 130.176 | 56(55) |
| Palestine | 0.402 | 157.89(113.46,213.00) | 90.132 | 67.761 | 59(59) | 0.631 | 235.40(185.41,294.55) | 84.412 | 150.988 | 66(65) |
| Panama | 0.546 | 388.12(314.66,472.61) | 84.415 | 303.707 | 138(138) | 0.709 | 538.32(457.12,629.09) | 84.413 | 453.903 | 136(136) |
| Papua New Guinea | 0.311 | 90.73(64.11,124.72) | 90.730 | 0.000 | 4(3) | 0.418 | 103.81(79.93,132.63) | 84.953 | 18.854 | 3(2) |
| Paraguay | 0.470 | 400.16(328.22,482.24) | 84.417 | 315.739 | 142(142) | 0.636 | 710.14(621.91,811.00) | 84.412 | 625.732 | 156(156) |
| Peru | 0.510 | 603.08(522.89,691.32) | 84.428 | 518.653 | 167(167) | 0.662 | 1077.29(952.61,1216.56) | 84.412 | 992.883 | 186(186) |
| Philippines | 0.510 | 284.65(232.31,346.01) | 84.422 | 200.223 | 113(112) | 0.651 | 262.77(217.64,315.44) | 84.412 | 178.362 | 77(76) |
| Poland | 0.627 | 814.50(705.16,941.49) | 84.412 | 730.085 | 185(185) | 0.812 | 719.98(626.60,824.42) | 84.414 | 635.566 | 158(158) |
| Portugal | 0.600 | 573.83(499.06,660.41) | 84.412 | 489.422 | 165(165) | 0.744 | 822.63(720.15,934.56) | 84.412 | 738.222 | 170(170) |
| Puerto Rico | 0.659 | 394.34(325.25,475.85) | 84.412 | 309.930 | 141(141) | 0.826 | 642.85(547.10,751.17) | 84.414 | 558.436 | 148(148) |
| Qatar | 0.651 | 168.08(72.10,323.45) | 84.413 | 83.665 | 65(64) | 0.847 | 312.23(226.18,415.92) | 84.412 | 227.818 | 91(91) |
| Republic of Korea | 0.692 | 537.65(466.25,616.91) | 84.413 | 453.238 | 158(158) | 0.887 | 819.44(717.33,930.02) | 84.414 | 735.028 | 169(169) |
| Republic of Moldova | 0.604 | 211.06(169.90,260.54) | 84.412 | 126.649 | 84(80) | 0.732 | 304.63(254.12,362.10) | 84.412 | 220.219 | 87(87) |
| Romania | 0.619 | 221.86(179.26,271.22) | 84.412 | 137.445 | 88(84) | 0.768 | 346.09(291.09,411.09) | 84.413 | 261.674 | 102(102) |
| Russian Federation | 0.672 | 393.78(345.08,448.29) | 84.413 | 309.364 | 140(140) | 0.809 | 437.24(388.20,490.62) | 84.412 | 352.830 | 123(123) |
| Rwanda | 0.275 | 257.20(208.51,315.52) | 93.907 | 163.293 | 101(101) | 0.436 | 358.41(300.66,426.14) | 84.432 | 273.974 | 105(105) |
| Saint Kitts and Nevis | 0.581 | 359.77(183.74,621.36) | 84.413 | 275.361 | 130(129) | 0.755 | 514.41(322.07,768.36) | 84.414 | 429.994 | 133(133) |
| Saint Lucia | 0.496 | 333.47(201.67,511.50) | 84.413 | 249.055 | 123(123) | 0.673 | 463.70(339.81,611.63) | 84.412 | 379.292 | 129(129) |
| Saint Vincent and the Grenadines | 0.476 | 207.68(101.63,367.12) | 84.419 | 123.264 | 80(78) | 0.637 | 253.21(155.08,384.48) | 84.412 | 168.799 | 76(73) |
| Samoa | 0.487 | 137.67(60.63,261.83) | 84.427 | 53.245 | 46(40) | 0.593 | 178.07(100.84,285.92) | 84.412 | 93.662 | 30(28) |
| San Marino | 0.813 | 381.35(174.98,696.11) | 84.414 | 296.936 | 134(134) | 0.888 | 386.75(218.30,622.03) | 84.413 | 302.341 | 113(113) |
| Sao Tome and Principe | 0.310 | 159.19(66.04,310.84) | 93.930 | 65.262 | 58(60) | 0.505 | 218.74(116.62,362.75) | 84.446 | 134.290 | 58(57) |
| Saudi Arabia | 0.539 | 114.15(87.70,146.31) | 84.430 | 29.720 | 21(16) | 0.815 | 200.19(162.99,244.97) | 84.416 | 115.778 | 47(46) |
| Senegal | 0.238 | 149.55(113.72,191.49) | 94.492 | 55.060 | 49(55) | 0.408 | 191.92(153.24,236.86) | 90.117 | 101.805 | 37(38) |
| Serbia | 0.631 | 358.49(311.35,411.18) | 84.413 | 274.079 | 129(127) | 0.792 | 527.51(451.14,611.14) | 84.412 | 443.093 | 135(135) |
| Seychelles | 0.576 | 119.43(40.44,261.46) | 84.418 | 35.015 | 24(19) | 0.730 | 165.01(84.72,282.52) | 84.412 | 80.596 | 26(23) |
| Sierra Leone | 0.212 | 137.63(101.15,180.47) | 97.166 | 40.461 | 30(39) | 0.359 | 163.96(127.18,206.61) | 90.656 | 73.308 | 18(22) |
| Singapore | 0.686 | 316.65(249.45,395.72) | 84.412 | 232.233 | 117(117) | 0.856 | 421.71(345.80,506.32) | 84.414 | 337.293 | 119(119) |
| Slovakia | 0.654 | 255.20(208.86,309.10) | 84.412 | 170.789 | 106(100) | 0.811 | 371.31(314.88,436.80) | 84.414 | 286.894 | 109(109) |
| Slovenia | 0.727 | 829.75(707.29,967.51) | 84.413 | 745.339 | 186(186) | 0.842 | 1171.76(1018.81,1333.74) | 84.412 | 1087.352 | 190(190) |
| Solomon Islands | 0.301 | 111.75(53.08,201.78) | 93.929 | 17.822 | 12(12) | 0.429 | 149.72(95.84,219.58) | 84.426 | 65.298 | 14(12) |
| Somalia | 0.049 | 257.35(205.10,318.11) | #### | 0.000 | 5(103) | 0.078 | 268.04(222.91,320.18) | ##### | 15.110 | 1(77) |
| South Africa | 0.542 | 734.82(631.18,855.41) | 84.416 | 650.399 | 182(182) | 0.680 | 680.65(589.21,789.79) | 84.412 | 596.238 | 153(153) |
| South Sudan | 0.207 | 220.55(174.31,276.19) | 97.132 | 123.423 | 82(82) | 0.278 | 257.57(208.15,315.74) | 93.916 | 163.652 | 73(74) |
| Spain | 0.637 | 667.28(598.35,743.71) | 84.412 | 582.863 | 176(176) | 0.769 | 831.68(763.19,910.68) | 84.414 | 747.271 | 172(172) |
| Sri Lanka | 0.523 | 105.67(81.06,136.23) | 84.430 | 21.239 | 15(9) | 0.702 | 150.36(120.92,185.29) | 84.412 | 65.948 | 15(13) |
| Sudan | 0.292 | 94.66(74.71,118.80) | 93.871 | 0.786 | 7(4) | 0.542 | 144.45(119.11,173.28) | 84.413 | 60.035 | 12(9) |
| Suriname | 0.502 | 166.75(101.28,253.89) | 84.415 | 82.339 | 64(62) | 0.634 | 214.16(154.33,289.81) | 84.417 | 129.747 | 55(54) |
| Sweden | 0.786 | 1542.76(1288.78,1845.55) | 84.413 | 1458.342 | 199(199) | 0.887 | 1371.07(1156.68,1626.47) | 84.412 | 1286.654 | 195(195) |
| Switzerland | 0.863 | 1136.04(969.02,1337.68) | 84.412 | 1051.628 | 192(192) | 0.933 | 1212.11(1040.12,1410.64) | 84.417 | 1127.695 | 192(192) |
| Syrian Arab Republic | 0.430 | 130.44(101.56,166.70) | 84.424 | 46.011 | 37(28) | 0.623 | 209.02(171.68,254.89) | 84.413 | 124.611 | 51(50) |
| Taiwan (Province of China) | 0.668 | 586.89(500.57,682.00) | 84.414 | 502.478 | 166(166) | 0.875 | 642.15(609.39,678.83) | 84.412 | 557.743 | 147(147) |
| Tajikistan | 0.466 | 287.67(233.12,349.32) | 84.429 | 203.240 | 115(113) | 0.542 | 375.71(314.19,446.68) | 84.422 | 291.283 | 111(111) |
| Thailand | 0.507 | 201.19(167.85,239.94) | 84.412 | 116.778 | 78(76) | 0.683 | 294.50(250.73,345.33) | 84.413 | 210.091 | 83(82) |
| Timor-Leste | 0.262 | 131.67(75.14,210.46) | 94.019 | 37.651 | 25(29) | 0.445 | 209.74(154.89,276.17) | 84.416 | 125.319 | 52(51) |
| Togo | 0.270 | 148.81(105.50,202.23) | 94.372 | 54.443 | 48(54) | 0.409 | 191.03(149.83,240.05) | 90.129 | 100.904 | 36(36) |
| Tokelau | 0.522 | 112.38(0.00,2202.52) | 84.424 | 27.957 | 20(13) | 0.686 | 163.95(0.00,2292.57) | 84.412 | 79.542 | 25(21) |
| Tonga | 0.492 | 127.04(43.36,278.31) | 84.434 | 42.602 | 33(24) | 0.626 | 159.53(73.24,295.49) | 84.412 | 75.115 | 19(17) |
| Trinidad and Tobago | 0.624 | 456.82(367.45,559.75) | 84.414 | 372.404 | 150(150) | 0.769 | 611.91(514.45,718.61) | 84.412 | 527.498 | 145(145) |
| Tunisia | 0.471 | 128.99(100.82,163.45) | 84.418 | 44.574 | 35(26) | 0.682 | 198.39(163.27,238.69) | 84.412 | 113.974 | 45(44) |
| Turkey | 0.462 | 325.94(284.75,374.90) | 84.414 | 241.526 | 121(121) | 0.713 | 557.30(493.94,629.42) | 84.413 | 472.886 | 138(138) |
| Turkmenistan | 0.563 | 147.07(109.46,193.66) | 84.441 | 62.629 | 56(51) | 0.682 | 198.41(159.37,245.86) | 84.414 | 113.994 | 46(45) |
| Tuvalu | 0.406 | 113.80(1.18,690.16) | 90.138 | 23.661 | 17(15) | 0.577 | 159.53(14.25,600.44) | 84.412 | 75.115 | 20(18) |
| Uganda | 0.187 | 243.43(199.35,296.26) | 97.127 | 146.302 | 92(94) | 0.423 | 313.35(263.41,369.45) | 84.499 | 228.849 | 93(93) |
| Ukraine | 0.647 | 180.52(148.16,220.29) | 84.412 | 96.106 | 70(69) | 0.761 | 226.06(189.22,269.36) | 84.413 | 141.651 | 61(60) |
| United Arab Emirates | 0.644 | 174.43(111.24,257.34) | 84.414 | 90.020 | 67(66) | 0.849 | 228.68(182.41,284.07) | 84.415 | 144.263 | 63(62) |
| United Kingdom | 0.744 | 1550.34(1330.12,1820.96) | 84.416 | 1465.922 | 200(200) | 0.859 | 1675.91(1450.18,1945.91) | 84.412 | 1591.493 | 202(202) |
| United Republic of Tanzania | 0.259 | 259.90(214.38,313.44) | 94.627 | 165.270 | 102(104) | 0.447 | 328.11(277.69,386.39) | 84.430 | 243.684 | 98(98) |
| United States Virgin Islands | 0.655 | 949.51(828.56,1091.17) | 84.416 | 181.321 | 110(107) | 0.822 | 1087.96(953.48,1240.12) | 84.412 | 275.260 | 106(106) |
| United States of America | 0.764 | 265.74(141.06,444.65) | 84.414 | 865.098 | 191(191) | 0.862 | 359.67(245.32,506.83) | 84.414 | 1003.551 | 188(188) |
| Uruguay | 0.582 | 497.50(416.71,591.00) | 84.421 | 413.079 | 154(153) | 0.719 | 708.84(605.56,821.61) | 84.413 | 624.424 | 155(155) |
| Uzbekistan | 0.500 | 523.98(460.82,591.80) | 84.419 | 439.561 | 155(155) | 0.663 | 657.11(588.71,734.70) | 84.412 | 572.701 | 149(149) |
| Vanuatu | 0.353 | 109.83(35.59,246.25) | 90.917 | 18.910 | 13(11) | 0.473 | 139.06(75.65,228.06) | 84.418 | 54.642 | 10(7) |
| Venezuela (Bolivarian Republic of) | 0.517 | 668.52(588.09,763.17) | 84.431 | 584.089 | 177(177) | 0.597 | 960.89(859.42,1075.31) | 84.413 | 876.481 | 181(181) |
| Viet Nam | 0.408 | 214.27(178.07,257.36) | 90.275 | 123.992 | 83(81) | 0.628 | 403.53(342.15,473.25) | 84.412 | 319.114 | 116(116) |
| Yemen | 0.216 | 95.85(73.73,123.60) | 95.855 | 0.000 | 6(5) | 0.450 | 126.20(102.78,153.78) | 84.420 | 41.776 | 6(4) |
| Zambia | 0.304 | 226.46(183.14,277.11) | 94.061 | 132.399 | 86(87) | 0.506 | 303.58(256.01,358.87) | 84.419 | 219.156 | 86(85) |
| Zimbabwe | 0.399 | 236.62(192.94,286.12) | 90.148 | 146.467 | 93(91) | 0.474 | 240.00(201.37,284.76) | 84.440 | 155.558 | 68(67) |

**Abbreviations: ASR, age-standardised rate;** **SDI: sociodemographic index.**

**Table S8. Age-standardised mortality rates of elderly-onset rheumatoid arthritis with frontier analysis across 204 countries and territories in 1990 and 2021.**

| **Location** | **Mortality, 1990** | | | | | **Mortality, 2021** | | | | |
| --- | --- | --- | --- | --- | --- | --- | --- | --- | --- | --- |
|  | **SDI** | **ASRs** | **Frontier** | **Effective difference** | **Effective difference rank (ASRs rank)** | **SDI** | **ASRs** | **Frontier** | **Effective difference** | **Effective difference rank (ASRs rank)** |
| Afghanistan | 0.174 | 0.98(0.10,3.19) | 0.035 | 0.947 | 98(99) | 0.337 | 1.81(0.31,5.01) | 0.006 | 1.803 | 124(126) |
| Albania | 0.558 | 2.17(0.33,6.28) | 0.002 | 2.167 | 129(129) | 0.707 | 1.10(0.17,3.19) | 0.000 | 1.102 | 103(103) |
| Algeria | 0.460 | 0.27(0.01,1.01) | 0.003 | 0.266 | 74(73) | 0.660 | 0.41(0.10,1.02) | 0.000 | 0.415 | 80(80) |
| American Samoa | 0.614 | 0.02(0.00,148.75) | 0.000 | 0.021 | 27(21) | 0.724 | 0.01(0.00,64.98) | 0.000 | 0.005 | 18(16) |
| Andorra | 0.761 | 0.51(0.00,51.59) | 0.000 | 0.514 | 90(89) | 0.869 | 0.30(0.00,20.16) | 0.000 | 0.305 | 70(70) |
| Angola | 0.271 | 0.33(0.00,5.04) | 0.035 | 0.293 | 77(77) | 0.454 | 0.21(0.00,3.14) | 0.003 | 0.208 | 61(60) |
| Antigua and Barbuda | 0.612 | 3.22(0.00,61.62) | 0.000 | 3.215 | 148(148) | 0.750 | 2.87(0.00,34.57) | 0.000 | 2.873 | 154(154) |
| Argentina | 0.587 | 2.13(1.39,3.08) | 0.000 | 2.135 | 127(127) | 0.723 | 2.25(1.52,3.17) | 0.000 | 2.255 | 137(137) |
| Armenia | 0.544 | 0.03(0.00,1.17) | 0.002 | 0.029 | 35(23) | 0.702 | 0.71(0.14,1.95) | 0.000 | 0.710 | 89(89) |
| Australia | 0.726 | 6.61(4.66,8.88) | 0.000 | 6.606 | 191(191) | 0.844 | 3.95(2.69,5.42) | 0.000 | 3.951 | 174(174) |
| Austria | 0.750 | 4.42(2.73,6.62) | 0.000 | 4.418 | 169(169) | 0.854 | 2.00(1.09,3.21) | 0.000 | 2.005 | 130(130) |
| Azerbaijan | 0.596 | 0.02(0.00,0.70) | 0.000 | 0.017 | 23(18) | 0.695 | 0.06(0.00,0.46) | 0.000 | 0.055 | 39(38) |
| Bahamas | 0.694 | 2.89(0.00,28.13) | 0.000 | 2.886 | 138(138) | 0.805 | 2.66(0.05,13.68) | 0.000 | 2.657 | 150(150) |
| Bahrain | 0.585 | 0.20(0.00,21.54) | 0.000 | 0.205 | 60(54) | 0.753 | 1.05(0.00,6.75) | 0.000 | 1.050 | 101(101) |
| Bangladesh | 0.229 | 5.90(2.71,10.96) | 0.034 | 5.866 | 186(186) | 0.492 | 4.12(1.98,7.52) | 0.003 | 4.115 | 180(180) |
| Barbados | 0.654 | 5.47(0.39,21.36) | 0.000 | 5.475 | 182(182) | 0.747 | 5.72(0.87,17.12) | 0.000 | 5.720 | 199(199) |
| Belarus | 0.622 | 0.09(0.00,0.41) | 0.000 | 0.091 | 45(44) | 0.784 | 0.11(0.01,0.41) | 0.000 | 0.111 | 42(40) |
| Belgium | 0.737 | 3.53(2.19,5.21) | 0.000 | 3.528 | 154(154) | 0.854 | 2.29(1.31,3.51) | 0.000 | 2.290 | 138(138) |
| Belize | 0.424 | 2.18(0.00,38.09) | 0.003 | 2.176 | 130(130) | 0.610 | 2.59(0.01,16.76) | 0.000 | 2.589 | 147(147) |
| Benin | 0.219 | 0.06(0.00,1.84) | 0.034 | 0.029 | 34(37) | 0.373 | 0.03(0.00,0.78) | 0.004 | 0.027 | 33(26) |
| Bermuda | 0.696 | 2.76(0.00,54.31) | 0.000 | 2.763 | 136(136) | 0.821 | 2.98(0.00,28.05) | 0.000 | 2.983 | 156(156) |
| Bhutan | 0.215 | 5.71(0.02,30.28) | 0.035 | 5.674 | 185(185) | 0.473 | 5.80(0.35,20.31) | 0.003 | 5.799 | 200(200) |
| Bolivia (Plurinational State of) | 0.424 | 6.06(2.13,12.80) | 0.003 | 6.053 | 187(187) | 0.599 | 5.36(2.20,10.31) | 0.000 | 5.356 | 196(196) |
| Bosnia and Herzegovina | 0.541 | 1.99(0.46,4.88) | 0.002 | 1.984 | 124(124) | 0.723 | 1.44(0.37,3.48) | 0.000 | 1.441 | 114(114) |
| Botswana | 0.418 | 2.94(0.03,13.64) | 0.003 | 2.939 | 140(139) | 0.643 | 2.11(0.02,7.78) | 0.000 | 2.108 | 133(133) |
| Brazil | 0.500 | 1.45(1.10,1.84) | 0.003 | 1.449 | 109(109) | 0.653 | 1.45(1.14,1.76) | 0.000 | 1.453 | 115(115) |
| Brunei Darussalam | 0.666 | 7.84(0.01,56.34) | 0.000 | 7.841 | 197(197) | 0.810 | 4.50(0.08,21.23) | 0.000 | 4.499 | 183(183) |
| Bulgaria | 0.633 | 0.73(0.30,1.44) | 0.000 | 0.734 | 96(96) | 0.768 | 0.48(0.15,1.07) | 0.000 | 0.479 | 84(84) |
| Burkina Faso | 0.130 | 0.08(0.00,1.07) | 0.036 | 0.045 | 43(42) | 0.285 | 0.04(0.00,0.52) | 0.035 | 0.007 | 21(37) |
| Burundi | 0.206 | 0.27(0.00,4.78) | 0.035 | 0.234 | 68(71) | 0.289 | 0.13(0.00,2.58) | 0.035 | 0.096 | 41(44) |
| Cabo Verde | 0.277 | 0.05(0.00,12.78) | 0.036 | 0.017 | 22(30) | 0.534 | 0.01(0.00,6.93) | 0.003 | 0.006 | 20(17) |
| Cambodia | 0.289 | 1.91(0.31,4.86) | 0.035 | 1.872 | 122(122) | 0.474 | 1.89(0.50,3.95) | 0.003 | 1.886 | 127(127) |
| Cameroon | 0.303 | 0.06(0.00,0.98) | 0.035 | 0.028 | 33(36) | 0.480 | 0.03(0.00,0.39) | 0.003 | 0.027 | 31(25) |
| Canada | 0.782 | 4.51(3.17,6.09) | 0.000 | 4.509 | 172(172) | 0.873 | 2.93(2.01,3.99) | 0.000 | 2.935 | 155(155) |
| Central African Republic | 0.217 | 0.41(0.00,8.75) | 0.035 | 0.372 | 82(82) | 0.309 | 0.26(0.00,5.71) | 0.007 | 0.256 | 67(67) |
| Chad | 0.115 | 0.07(0.00,1.40) | 0.046 | 0.021 | 28(40) | 0.240 | 0.04(0.00,0.76) | 0.034 | 0.005 | 19(35) |
| Chile | 0.586 | 5.51(3.45,8.11) | 0.000 | 5.511 | 183(183) | 0.772 | 3.85(2.51,5.50) | 0.000 | 3.852 | 172(172) |
| China | 0.459 | 3.39(2.59,4.42) | 0.003 | 3.385 | 151(151) | 0.722 | 3.31(2.22,4.20) | 0.000 | 3.308 | 163(163) |
| Colombia | 0.481 | 4.66(3.14,6.52) | 0.003 | 4.656 | 173(173) | 0.655 | 3.36(2.20,4.78) | 0.000 | 3.359 | 166(166) |
| Comoros | 0.270 | 0.21(0.00,19.57) | 0.034 | 0.171 | 55(55) | 0.476 | 0.15(0.00,9.39) | 0.003 | 0.147 | 51(50) |
| Congo | 0.421 | 0.43(0.00,9.30) | 0.003 | 0.432 | 87(86) | 0.583 | 0.23(0.00,4.94) | 0.000 | 0.227 | 63(63) |
| Cook Islands | 0.565 | 0.00(0.00,251.31) | 0.000 | 0.000 | 1(1) | 0.779 | 0.00(0.00,108.37) | 0.000 | 0.000 | 1(1) |
| Costa Rica | 0.534 | 5.03(1.87,10.34) | 0.003 | 5.031 | 177(177) | 0.700 | 5.11(2.60,8.44) | 0.000 | 5.111 | 193(193) |
| C么te d'Ivoire | 0.279 | 0.04(0.00,1.05) | 0.034 | 0.005 | 15(26) | 0.426 | 0.03(0.00,0.42) | 0.003 | 0.024 | 28(23) |
| Croatia | 0.669 | 2.65(1.33,4.57) | 0.000 | 2.651 | 132(132) | 0.798 | 2.21(1.09,3.82) | 0.000 | 2.212 | 135(135) |
| Cuba | 0.558 | 2.67(1.43,4.33) | 0.002 | 2.664 | 133(133) | 0.669 | 3.33(2.01,5.04) | 0.000 | 3.333 | 164(164) |
| Cyprus | 0.648 | 10.73(2.49,27.09) | 0.000 | 10.733 | 200(200) | 0.836 | 4.59(1.18,10.85) | 0.000 | 4.588 | 187(187) |
| Czechia | 0.682 | 1.53(0.83,2.51) | 0.000 | 1.526 | 110(110) | 0.828 | 1.05(0.54,1.79) | 0.000 | 1.051 | 102(102) |
| Democratic People's Republic of Korea | 0.498 | 3.90(1.72,7.37) | 0.003 | 3.895 | 162(162) | 0.570 | 4.51(2.30,7.85) | 0.000 | 4.511 | 184(184) |
| Democratic Republic of the Congo | 0.290 | 0.32(0.01,3.78) | 0.035 | 0.289 | 76(76) | 0.383 | 0.24(0.01,2.95) | 0.004 | 0.232 | 64(65) |
| Denmark | 0.801 | 4.76(2.79,7.37) | 0.000 | 4.760 | 174(174) | 0.896 | 4.58(2.73,6.93) | 0.000 | 4.584 | 186(186) |
| Djibouti | 0.338 | 0.17(0.00,29.84) | 0.006 | 0.159 | 51(48) | 0.488 | 0.12(0.00,7.46) | 0.003 | 0.112 | 43(41) |
| Dominica | 0.564 | 3.49(0.00,58.10) | 0.002 | 3.486 | 153(153) | 0.747 | 3.05(0.00,43.31) | 0.000 | 3.046 | 159(159) |
| Dominican Republic | 0.443 | 3.59(0.85,8.03) | 0.003 | 3.584 | 155(155) | 0.619 | 3.35(1.21,6.74) | 0.000 | 3.352 | 165(165) |
| Ecuador | 0.518 | 6.83(4.10,10.50) | 0.003 | 6.829 | 193(193) | 0.661 | 4.83(2.93,7.29) | 0.000 | 4.828 | 188(188) |
| Egypt | 0.417 | 0.51(0.13,1.20) | 0.003 | 0.512 | 89(90) | 0.607 | 0.34(0.12,0.70) | 0.000 | 0.337 | 76(76) |
| El Salvador | 0.373 | 1.73(0.26,5.10) | 0.004 | 1.722 | 118(118) | 0.564 | 1.67(0.42,4.04) | 0.002 | 1.663 | 122(122) |
| Equatorial Guinea | 0.269 | 0.42(0.00,24.56) | 0.034 | 0.382 | 83(83) | 0.658 | 0.20(0.00,10.86) | 0.000 | 0.201 | 60(59) |
| Eritrea | 0.216 | 0.20(0.00,6.43) | 0.034 | 0.167 | 52(53) | 0.404 | 0.16(0.00,3.88) | 0.003 | 0.155 | 53(52) |
| Estonia | 0.675 | 4.08(1.51,8.33) | 0.000 | 4.076 | 166(166) | 0.845 | 4.09(1.66,7.98) | 0.000 | 4.092 | 178(178) |
| Eswatini | 0.399 | 4.46(0.00,24.42) | 0.003 | 4.459 | 170(170) | 0.585 | 3.56(0.00,16.64) | 0.000 | 3.559 | 170(170) |
| Ethiopia | 0.148 | 0.27(0.01,2.51) | 0.036 | 0.230 | 66(70) | 0.359 | 0.13(0.00,1.46) | 0.005 | 0.121 | 45(42) |
| Fiji | 0.535 | 0.00(0.00,9.99) | 0.002 | 0.000 | 2(2) | 0.675 | 0.00(0.00,4.02) | 0.000 | 0.001 | 6(2) |
| Finland | 0.756 | 13.43(9.16,18.58) | 0.000 | 13.426 | 204(204) | 0.860 | 6.37(4.01,9.14) | 0.000 | 6.366 | 201(201) |
| France | 0.731 | 4.21(3.09,5.54) | 0.000 | 4.209 | 167(167) | 0.838 | 2.33(1.57,3.16) | 0.000 | 2.326 | 139(139) |
| Gabon | 0.455 | 0.43(0.00,11.82) | 0.003 | 0.428 | 86(85) | 0.635 | 0.22(0.00,6.90) | 0.000 | 0.220 | 62(62) |
| Gambia | 0.239 | 0.06(0.00,9.78) | 0.035 | 0.021 | 29(32) | 0.410 | 0.04(0.00,3.56) | 0.003 | 0.033 | 38(32) |
| Georgia | 0.740 | 0.02(0.00,0.50) | 0.000 | 0.019 | 25(20) | 0.847 | 0.33(0.04,1.11) | 0.000 | 0.329 | 75(75) |
| Germany | 0.817 | 3.98(2.93,5.18) | 0.000 | 3.983 | 165(165) | 0.903 | 2.22(1.52,2.99) | 0.000 | 2.224 | 136(136) |
| Ghana | 0.373 | 0.04(0.00,0.69) | 0.004 | 0.031 | 38(24) | 0.565 | 0.29(0.01,1.11) | 0.000 | 0.288 | 69(69) |
| Greece | 0.674 | 1.15(0.58,1.98) | 0.000 | 1.150 | 104(104) | 0.792 | 1.48(0.81,2.36) | 0.000 | 1.480 | 117(117) |
| Greenland | 0.732 | 2.73(0.00,112.15) | 0.000 | 2.728 | 135(135) | 0.826 | 2.03(0.00,49.02) | 0.000 | 2.026 | 131(131) |
| Grenada | 0.437 | 1.71(0.00,43.66) | 0.003 | 1.710 | 117(117) | 0.669 | 1.30(0.00,30.01) | 0.000 | 1.298 | 108(108) |
| Guam | 0.676 | 0.01(0.00,41.95) | 0.000 | 0.006 | 16(4) | 0.804 | 0.00(0.00,13.33) | 0.000 | 0.001 | 7(3) |
| Guatemala | 0.312 | 2.94(1.20,5.78) | 0.008 | 2.937 | 139(140) | 0.540 | 3.64(2.10,5.72) | 0.003 | 3.638 | 171(171) |
| Guinea | 0.178 | 0.05(0.00,1.13) | 0.035 | 0.019 | 26(31) | 0.336 | 0.04(0.00,0.72) | 0.006 | 0.029 | 34(30) |
| Guinea-Bissau | 0.208 | 0.07(0.00,8.59) | 0.036 | 0.039 | 39(41) | 0.353 | 0.04(0.00,5.02) | 0.006 | 0.031 | 35(33) |
| Guyana | 0.460 | 0.12(0.00,8.73) | 0.003 | 0.118 | 47(46) | 0.651 | 0.98(0.00,7.18) | 0.000 | 0.981 | 98(98) |
| Haiti | 0.310 | 3.25(0.59,9.04) | 0.007 | 3.247 | 149(149) | 0.448 | 2.76(0.61,7.33) | 0.003 | 2.758 | 152(152) |
| Honduras | 0.332 | 7.26(1.75,18.41) | 0.006 | 7.250 | 195(195) | 0.513 | 9.24(3.95,17.12) | 0.003 | 9.241 | 204(204) |
| Hungary | 0.649 | 3.90(2.51,5.67) | 0.000 | 3.900 | 163(163) | 0.791 | 2.16(1.20,3.40) | 0.000 | 2.162 | 134(134) |
| Iceland | 0.764 | 3.67(0.10,17.98) | 0.000 | 3.668 | 157(157) | 0.876 | 1.99(0.06,9.33) | 0.000 | 1.987 | 129(129) |
| India | 0.333 | 5.11(3.23,7.57) | 0.006 | 5.100 | 179(179) | 0.575 | 5.00(3.45,7.57) | 0.000 | 4.998 | 191(191) |
| Indonesia | 0.457 | 0.59(0.16,1.09) | 0.003 | 0.582 | 94(94) | 0.657 | 0.74(0.22,1.21) | 0.000 | 0.744 | 91(91) |
| Iran (Islamic Republic of) | 0.454 | 0.24(0.04,0.65) | 0.003 | 0.241 | 70(67) | 0.697 | 0.31(0.13,0.58) | 0.000 | 0.313 | 71(71) |
| Iraq | 0.412 | 0.24(0.00,1.12) | 0.003 | 0.232 | 67(65) | 0.663 | 0.15(0.01,0.55) | 0.000 | 0.151 | 52(51) |
| Ireland | 0.720 | 10.58(6.69,15.74) | 0.000 | 10.580 | 199(199) | 0.874 | 5.25(2.99,8.12) | 0.000 | 5.251 | 195(195) |
| Israel | 0.709 | 1.69(0.62,3.53) | 0.000 | 1.692 | 116(116) | 0.809 | 1.33(0.58,2.43) | 0.000 | 1.334 | 109(109) |
| Italy | 0.706 | 2.95(2.34,3.54) | 0.000 | 2.952 | 141(141) | 0.806 | 3.17(2.33,3.88) | 0.000 | 3.174 | 160(160) |
| Jamaica | 0.535 | 2.05(0.38,5.66) | 0.003 | 2.050 | 125(125) | 0.683 | 2.55(0.72,5.83) | 0.000 | 2.552 | 144(144) |
| Japan | 0.790 | 6.28(5.34,7.00) | 0.000 | 6.283 | 189(189) | 0.871 | 4.87(3.68,5.72) | 0.000 | 4.870 | 190(190) |
| Jordan | 0.539 | 0.25(0.00,3.53) | 0.002 | 0.244 | 72(68) | 0.725 | 0.23(0.00,1.15) | 0.000 | 0.235 | 65(64) |
| Kazakhstan | 0.589 | 0.04(0.00,0.34) | 0.000 | 0.039 | 40(25) | 0.725 | 0.97(0.43,1.81) | 0.000 | 0.972 | 97(97) |
| Kenya | 0.334 | 0.17(0.00,2.35) | 0.006 | 0.168 | 53(50) | 0.524 | 0.13(0.00,1.66) | 0.003 | 0.125 | 46(43) |
| Kiribati | 0.410 | 0.00(0.00,89.58) | 0.003 | 0.001 | 8(3) | 0.527 | 0.00(0.00,45.05) | 0.003 | 0.000 | 2(6) |
| Kuwait | 0.665 | 0.22(0.00,6.88) | 0.000 | 0.218 | 65(59) | 0.847 | 0.87(0.06,3.25) | 0.000 | 0.872 | 95(95) |
| Kyrgyzstan | 0.519 | 1.31(0.23,3.87) | 0.003 | 1.302 | 106(106) | 0.604 | 2.34(0.80,5.16) | 0.000 | 2.344 | 140(140) |
| Lao People's Democratic Republic | 0.264 | 1.87(0.13,6.12) | 0.034 | 1.832 | 120(121) | 0.489 | 1.60(0.21,4.30) | 0.003 | 1.594 | 119(119) |
| Latvia | 0.680 | 3.00(1.23,5.78) | 0.000 | 2.997 | 145(145) | 0.831 | 3.28(1.41,6.23) | 0.000 | 3.275 | 162(162) |
| Lebanon | 0.537 | 0.56(0.00,3.10) | 0.003 | 0.561 | 93(93) | 0.745 | 0.33(0.01,1.39) | 0.000 | 0.326 | 73(73) |
| Lesotho | 0.339 | 3.18(0.10,11.84) | 0.006 | 3.178 | 146(146) | 0.510 | 3.55(0.06,11.86) | 0.003 | 3.548 | 169(169) |
| Liberia | 0.235 | 0.06(0.00,2.91) | 0.034 | 0.029 | 37(38) | 0.352 | 0.03(0.00,1.86) | 0.006 | 0.027 | 32(28) |
| Libya | 0.528 | 0.30(0.00,2.75) | 0.003 | 0.298 | 78(75) | 0.726 | 0.73(0.03,2.71) | 0.000 | 0.733 | 90(90) |
| Lithuania | 0.669 | 3.64(1.76,6.41) | 0.000 | 3.644 | 156(156) | 0.856 | 4.56(2.42,7.54) | 0.000 | 4.564 | 185(185) |
| Luxembourg | 0.781 | 3.21(0.29,11.77) | 0.000 | 3.213 | 147(147) | 0.884 | 1.65(0.12,6.28) | 0.000 | 1.647 | 121(121) |
| Madagascar | 0.280 | 0.18(0.00,2.73) | 0.035 | 0.143 | 48(51) | 0.400 | 0.10(0.00,1.64) | 0.003 | 0.093 | 40(39) |
| Malawi | 0.204 | 0.22(0.00,3.62) | 0.034 | 0.190 | 58(63) | 0.385 | 0.16(0.00,2.84) | 0.004 | 0.161 | 54(53) |
| Malaysia | 0.546 | 0.55(0.06,1.81) | 0.002 | 0.550 | 91(91) | 0.743 | 0.36(0.08,0.94) | 0.000 | 0.356 | 78(78) |
| Maldives | 0.332 | 1.59(0.00,42.81) | 0.006 | 1.581 | 113(113) | 0.651 | 1.13(0.00,13.61) | 0.000 | 1.132 | 105(105) |
| Mali | 0.127 | 0.07(0.00,1.09) | 0.040 | 0.026 | 32(39) | 0.269 | 0.04(0.00,0.54) | 0.034 | 0.008 | 22(36) |
| Malta | 0.657 | 2.99(0.14,13.07) | 0.000 | 2.992 | 144(143) | 0.802 | 1.79(0.14,6.62) | 0.000 | 1.790 | 123(123) |
| Marshall Islands | 0.431 | 0.01(0.00,200.50) | 0.003 | 0.004 | 11(13) | 0.574 | 0.00(0.00,95.60) | 0.000 | 0.003 | 12(7) |
| Mauritania | 0.336 | 0.09(0.00,3.58) | 0.006 | 0.080 | 44(43) | 0.499 | 0.04(0.00,1.61) | 0.003 | 0.032 | 36(29) |
| Mauritius | 0.545 | 0.11(0.00,4.43) | 0.002 | 0.105 | 46(45) | 0.718 | 1.95(0.40,5.28) | 0.000 | 1.953 | 128(128) |
| Mexico | 0.505 | 12.32(10.55,14.05) | 0.003 | 12.312 | 203(203) | 0.665 | 7.65(6.16,9.19) | 0.000 | 7.654 | 203(203) |
| Micronesia (Federated States of) | 0.463 | 0.01(0.00,62.60) | 0.003 | 0.007 | 19(16) | 0.588 | 0.00(0.00,41.99) | 0.000 | 0.003 | 15(10) |
| Monaco | 0.845 | 0.21(0.00,40.65) | 0.000 | 0.212 | 61(57) | 0.908 | 0.17(0.00,30.16) | 0.000 | 0.174 | 57(56) |
| Mongolia | 0.467 | 1.02(0.00,8.01) | 0.003 | 1.016 | 100(100) | 0.618 | 1.80(0.17,5.85) | 0.000 | 1.803 | 125(124) |
| Montenegro | 0.674 | 0.98(0.00,7.35) | 0.000 | 0.982 | 99(98) | 0.796 | 1.03(0.01,5.36) | 0.000 | 1.026 | 100(100) |
| Morocco | 0.358 | 0.43(0.03,1.70) | 0.006 | 0.424 | 85(84) | 0.563 | 0.76(0.14,2.15) | 0.002 | 0.761 | 93(93) |
| Mozambique | 0.173 | 0.23(0.00,3.27) | 0.035 | 0.193 | 59(64) | 0.326 | 0.17(0.00,2.60) | 0.006 | 0.167 | 55(55) |
| Myanmar | 0.319 | 1.64(0.50,3.24) | 0.006 | 1.635 | 115(115) | 0.534 | 1.53(0.56,2.82) | 0.003 | 1.524 | 118(118) |
| Namibia | 0.450 | 2.99(0.05,12.95) | 0.003 | 2.991 | 143(144) | 0.618 | 2.58(0.03,9.61) | 0.000 | 2.577 | 146(146) |
| Nauru | 0.539 | 0.01(0.00,739.93) | 0.002 | 0.004 | 13(9) | 0.625 | 0.00(0.00,580.51) | 0.000 | 0.004 | 16(13) |
| Nepal | 0.200 | 4.98(1.71,10.54) | 0.035 | 4.947 | 175(176) | 0.433 | 5.10(2.14,9.97) | 0.003 | 5.097 | 192(192) |
| Netherlands | 0.795 | 9.84(7.10,12.93) | 0.000 | 9.838 | 198(198) | 0.888 | 4.85(3.26,6.62) | 0.000 | 4.854 | 189(189) |
| New Zealand | 0.752 | 7.50(4.30,11.78) | 0.000 | 7.499 | 196(196) | 0.849 | 4.05(2.21,6.47) | 0.000 | 4.054 | 177(177) |
| Nicaragua | 0.346 | 3.97(0.73,10.86) | 0.006 | 3.960 | 164(164) | 0.524 | 3.37(1.17,7.17) | 0.003 | 3.371 | 167(167) |
| Niger | 0.081 | 0.06(0.00,1.46) | 0.058 | 0.000 | 3(34) | 0.168 | 0.04(0.00,0.55) | 0.036 | 0.002 | 9(34) |
| Nigeria | 0.306 | 0.05(0.00,0.22) | 0.007 | 0.045 | 42(29) | 0.503 | 0.03(0.00,0.12) | 0.003 | 0.023 | 27(22) |
| Niue | 0.588 | 0.01(0.00,1323.70) | 0.000 | 0.007 | 18(12) | 0.726 | 0.00(0.00,1310.34) | 0.000 | 0.002 | 10(4) |
| North Macedonia | 0.609 | 0.50(0.00,2.98) | 0.000 | 0.502 | 88(88) | 0.751 | 0.28(0.00,1.64) | 0.000 | 0.276 | 68(68) |
| Northern Mariana Islands | 0.709 | 0.01(0.00,252.00) | 0.000 | 0.013 | 21(17) | 0.772 | 0.02(0.00,60.15) | 0.000 | 0.022 | 25(20) |
| Norway | 0.796 | 11.33(8.11,14.84) | 0.000 | 11.335 | 201(201) | 0.916 | 4.04(2.42,5.94) | 0.000 | 4.043 | 175(175) |
| Oman | 0.429 | 0.24(0.00,6.14) | 0.003 | 0.240 | 69(66) | 0.773 | 0.25(0.00,2.71) | 0.000 | 0.245 | 66(66) |
| Pakistan | 0.310 | 6.78(3.47,11.21) | 0.009 | 6.773 | 192(192) | 0.504 | 6.92(3.64,11.27) | 0.003 | 6.920 | 202(202) |
| Palau | 0.663 | 0.03(0.00,309.90) | 0.000 | 0.029 | 36(22) | 0.754 | 0.02(0.00,133.80) | 0.000 | 0.018 | 24(19) |
| Palestine | 0.402 | 0.56(0.00,5.28) | 0.003 | 0.560 | 92(92) | 0.631 | 0.35(0.00,2.32) | 0.000 | 0.346 | 77(77) |
| Panama | 0.546 | 2.88(0.66,7.45) | 0.002 | 2.875 | 137(137) | 0.709 | 3.01(1.12,5.85) | 0.000 | 3.008 | 157(157) |
| Papua New Guinea | 0.311 | 0.01(0.00,1.90) | 0.006 | 0.000 | 4(5) | 0.418 | 0.00(0.00,0.73) | 0.003 | 0.000 | 3(8) |
| Paraguay | 0.470 | 1.93(0.29,5.80) | 0.003 | 1.924 | 123(123) | 0.636 | 3.53(1.17,7.31) | 0.000 | 3.530 | 168(168) |
| Peru | 0.510 | 5.04(2.17,8.69) | 0.003 | 5.036 | 178(178) | 0.662 | 3.19(1.59,5.50) | 0.000 | 3.191 | 161(161) |
| Philippines | 0.510 | 2.11(0.85,3.33) | 0.003 | 2.106 | 126(126) | 0.651 | 1.81(0.97,2.54) | 0.000 | 1.807 | 126(125) |
| Poland | 0.627 | 6.60(5.55,7.68) | 0.000 | 6.603 | 190(190) | 0.812 | 2.48(1.90,3.10) | 0.000 | 2.481 | 143(143) |
| Portugal | 0.600 | 3.47(2.19,5.09) | 0.000 | 3.471 | 152(152) | 0.744 | 2.79(1.69,4.13) | 0.000 | 2.786 | 153(153) |
| Puerto Rico | 0.659 | 3.85(1.79,6.87) | 0.000 | 3.845 | 160(160) | 0.826 | 3.02(1.42,5.29) | 0.000 | 3.024 | 158(158) |
| Qatar | 0.651 | 0.26(0.00,43.91) | 0.000 | 0.257 | 73(69) | 0.847 | 0.17(0.00,5.29) | 0.000 | 0.170 | 56(54) |
| Republic of Korea | 0.692 | 4.27(2.17,7.72) | 0.000 | 4.265 | 168(168) | 0.887 | 1.47(0.74,2.68) | 0.000 | 1.474 | 116(116) |
| Republic of Moldova | 0.604 | 0.73(0.14,2.06) | 0.000 | 0.726 | 95(95) | 0.732 | 1.12(0.37,2.45) | 0.000 | 1.116 | 104(104) |
| Romania | 0.619 | 0.02(0.00,0.15) | 0.000 | 0.018 | 24(19) | 0.768 | 0.02(0.00,0.11) | 0.000 | 0.015 | 23(18) |
| Russian Federation | 0.672 | 2.14(1.87,2.42) | 0.000 | 2.141 | 128(128) | 0.809 | 2.66(2.21,3.12) | 0.000 | 2.655 | 149(149) |
| Rwanda | 0.275 | 0.28(0.00,4.58) | 0.036 | 0.243 | 71(74) | 0.436 | 0.14(0.00,2.55) | 0.003 | 0.136 | 49(47) |
| Saint Kitts and Nevis | 0.581 | 4.49(0.00,81.36) | 0.000 | 4.494 | 171(171) | 0.755 | 4.05(0.00,51.61) | 0.000 | 4.049 | 176(176) |
| Saint Lucia | 0.496 | 3.86(0.00,41.75) | 0.003 | 3.857 | 161(161) | 0.673 | 4.36(0.09,21.99) | 0.000 | 4.358 | 181(181) |
| Saint Vincent and the Grenadines | 0.476 | 1.87(0.00,44.47) | 0.003 | 1.863 | 121(120) | 0.637 | 1.27(0.00,23.22) | 0.000 | 1.268 | 106(106) |
| Samoa | 0.487 | 0.01(0.00,36.34) | 0.003 | 0.004 | 9(10) | 0.593 | 0.00(0.00,21.69) | 0.000 | 0.003 | 13(9) |
| San Marino | 0.813 | 1.34(0.00,84.82) | 0.000 | 1.342 | 107(107) | 0.888 | 0.67(0.00,42.62) | 0.000 | 0.667 | 88(88) |
| Sao Tome and Principe | 0.310 | 0.05(0.00,45.73) | 0.007 | 0.045 | 41(28) | 0.505 | 0.03(0.00,30.07) | 0.003 | 0.023 | 26(21) |
| Saudi Arabia | 0.539 | 0.34(0.00,1.69) | 0.002 | 0.334 | 79(78) | 0.815 | 0.33(0.04,1.06) | 0.000 | 0.328 | 74(74) |
| Senegal | 0.238 | 0.06(0.00,1.23) | 0.034 | 0.026 | 31(35) | 0.408 | 0.04(0.00,0.56) | 0.003 | 0.033 | 37(31) |
| Serbia | 0.631 | 1.79(0.64,3.73) | 0.000 | 1.789 | 119(119) | 0.792 | 1.40(0.52,2.87) | 0.000 | 1.402 | 111(111) |
| Seychelles | 0.576 | 0.27(0.00,51.82) | 0.000 | 0.269 | 75(72) | 0.730 | 0.19(0.00,26.45) | 0.000 | 0.186 | 58(57) |
| Sierra Leone | 0.212 | 0.06(0.00,1.75) | 0.034 | 0.023 | 30(33) | 0.359 | 0.03(0.00,1.02) | 0.005 | 0.026 | 30(27) |
| Singapore | 0.686 | 1.14(0.13,3.78) | 0.000 | 1.141 | 103(103) | 0.856 | 0.32(0.05,0.99) | 0.000 | 0.324 | 72(72) |
| Slovakia | 0.654 | 1.55(0.44,3.49) | 0.000 | 1.548 | 112(112) | 0.811 | 0.98(0.25,2.34) | 0.000 | 0.981 | 99(99) |
| Slovenia | 0.727 | 5.16(2.29,9.58) | 0.000 | 5.158 | 180(180) | 0.842 | 2.74(1.08,5.29) | 0.000 | 2.740 | 151(151) |
| Solomon Islands | 0.301 | 0.01(0.00,24.69) | 0.007 | 0.000 | 5(8) | 0.429 | 0.00(0.00,10.26) | 0.003 | 0.001 | 8(14) |
| Somalia | 0.049 | 0.34(0.00,5.30) | 0.337 | 0.000 | 6(79) | 0.078 | 0.22(0.00,3.22) | 0.220 | 0.000 | 4(61) |
| South Africa | 0.542 | 4.97(2.68,7.72) | 0.002 | 4.969 | 176(175) | 0.680 | 3.87(2.59,5.63) | 0.000 | 3.866 | 173(173) |
| South Sudan | 0.207 | 0.21(0.00,3.59) | 0.034 | 0.180 | 57(58) | 0.278 | 0.15(0.00,2.96) | 0.035 | 0.113 | 44(49) |
| Spain | 0.637 | 2.96(2.05,4.09) | 0.000 | 2.959 | 142(142) | 0.769 | 2.08(1.35,2.90) | 0.000 | 2.080 | 132(132) |
| Sri Lanka | 0.523 | 0.22(0.01,0.84) | 0.003 | 0.217 | 64(61) | 0.702 | 0.20(0.03,0.57) | 0.000 | 0.199 | 59(58) |
| Sudan | 0.292 | 0.44(0.02,1.75) | 0.034 | 0.407 | 84(87) | 0.542 | 0.58(0.10,1.62) | 0.002 | 0.582 | 86(86) |
| Suriname | 0.502 | 1.07(0.00,15.79) | 0.003 | 1.064 | 102(102) | 0.634 | 0.79(0.00,7.30) | 0.000 | 0.789 | 94(94) |
| Sweden | 0.786 | 6.98(4.86,9.42) | 0.000 | 6.983 | 194(194) | 0.887 | 4.10(2.55,5.93) | 0.000 | 4.101 | 179(179) |
| Switzerland | 0.863 | 5.17(3.05,7.84) | 0.000 | 5.167 | 181(181) | 0.933 | 2.55(1.35,4.06) | 0.000 | 2.555 | 145(145) |
| Syrian Arab Republic | 0.430 | 0.34(0.01,1.53) | 0.003 | 0.335 | 80(80) | 0.623 | 0.45(0.07,1.21) | 0.000 | 0.447 | 81(81) |
| Taiwan (Province of China) | 0.668 | 3.28(2.16,4.70) | 0.000 | 3.282 | 150(150) | 0.875 | 2.45(1.59,3.53) | 0.000 | 2.453 | 141(141) |
| Tajikistan | 0.466 | 0.17(0.00,1.88) | 0.003 | 0.170 | 54(49) | 0.542 | 0.47(0.01,1.85) | 0.003 | 0.465 | 83(83) |
| Thailand | 0.507 | 1.63(0.63,3.40) | 0.003 | 1.622 | 114(114) | 0.683 | 1.34(0.63,2.50) | 0.000 | 1.344 | 110(110) |
| Timor-Leste | 0.262 | 1.27(0.00,17.57) | 0.034 | 1.239 | 105(105) | 0.445 | 1.44(0.00,7.44) | 0.003 | 1.435 | 113(113) |
| Togo | 0.270 | 0.05(0.00,2.96) | 0.035 | 0.012 | 20(27) | 0.409 | 0.03(0.00,1.03) | 0.003 | 0.026 | 29(24) |
| Tokelau | 0.522 | 0.01(0.00,1933.06) | 0.003 | 0.004 | 10(11) | 0.686 | 0.00(0.00,1912.00) | 0.000 | 0.003 | 11(5) |
| Tonga | 0.492 | 0.01(0.00,55.78) | 0.003 | 0.005 | 14(14) | 0.626 | 0.00(0.00,38.32) | 0.000 | 0.004 | 17(15) |
| Trinidad and Tobago | 0.624 | 5.62(1.43,13.83) | 0.000 | 5.620 | 184(184) | 0.769 | 4.38(1.43,9.38) | 0.000 | 4.382 | 182(182) |
| Tunisia | 0.471 | 0.22(0.00,1.29) | 0.003 | 0.217 | 63(60) | 0.682 | 0.37(0.03,1.23) | 0.000 | 0.373 | 79(79) |
| Turkey | 0.462 | 2.69(1.18,5.10) | 0.003 | 2.687 | 134(134) | 0.713 | 1.64(0.83,2.80) | 0.000 | 1.639 | 120(120) |
| Turkmenistan | 0.563 | 0.01(0.00,1.66) | 0.002 | 0.004 | 12(6) | 0.682 | 0.45(0.02,1.89) | 0.000 | 0.454 | 82(82) |
| Tuvalu | 0.406 | 0.01(0.00,440.62) | 0.003 | 0.006 | 17(15) | 0.577 | 0.00(0.00,279.64) | 0.000 | 0.003 | 14(12) |
| Uganda | 0.187 | 0.19(0.00,2.44) | 0.036 | 0.154 | 50(52) | 0.423 | 0.14(0.00,2.15) | 0.003 | 0.138 | 50(48) |
| Ukraine | 0.647 | 0.79(0.35,1.23) | 0.000 | 0.785 | 97(97) | 0.761 | 0.76(0.39,1.29) | 0.000 | 0.761 | 92(92) |
| United Arab Emirates | 0.644 | 1.03(0.00,14.33) | 0.000 | 1.032 | 101(101) | 0.849 | 0.49(0.01,2.42) | 0.000 | 0.492 | 85(85) |
| United Kingdom | 0.744 | 12.05(10.54,13.26) | 0.000 | 12.047 | 202(202) | 0.859 | 5.71(4.64,6.56) | 0.000 | 5.711 | 198(198) |
| United Republic of Tanzania | 0.259 | 0.21(0.00,2.56) | 0.034 | 0.176 | 56(56) | 0.447 | 0.14(0.00,1.89) | 0.003 | 0.132 | 47(45) |
| United States Virgin Islands | 0.655 | 1.35(0.00,42.12) | 0.000 | 1.351 | 108(108) | 0.822 | 0.90(0.00,17.72) | 0.000 | 0.895 | 96(96) |
| United States of America | 0.764 | 3.67(3.14,4.09) | 0.000 | 3.670 | 158(158) | 0.862 | 2.64(2.14,3.06) | 0.000 | 2.638 | 148(148) |
| Uruguay | 0.582 | 3.72(1.76,6.63) | 0.000 | 3.718 | 159(159) | 0.719 | 5.39(2.95,8.62) | 0.000 | 5.386 | 197(197) |
| Uzbekistan | 0.500 | 0.15(0.01,0.78) | 0.003 | 0.152 | 49(47) | 0.663 | 1.29(0.71,2.08) | 0.000 | 1.291 | 107(107) |
| Vanuatu | 0.353 | 0.01(0.00,53.24) | 0.006 | 0.001 | 7(7) | 0.473 | 0.00(0.00,19.13) | 0.003 | 0.001 | 5(11) |
| Venezuela (Bolivarian Republic of) | 0.517 | 6.09(3.87,8.88) | 0.003 | 6.087 | 188(188) | 0.597 | 5.16(3.12,7.79) | 0.000 | 5.161 | 194(194) |
| Viet Nam | 0.408 | 1.53(0.53,2.94) | 0.003 | 1.527 | 111(111) | 0.628 | 1.42(0.57,2.60) | 0.000 | 1.421 | 112(112) |
| Yemen | 0.216 | 0.39(0.00,1.93) | 0.035 | 0.357 | 81(81) | 0.450 | 0.62(0.09,1.84) | 0.003 | 0.620 | 87(87) |
| Zambia | 0.304 | 0.22(0.00,3.94) | 0.010 | 0.213 | 62(62) | 0.506 | 0.14(0.00,2.35) | 0.003 | 0.132 | 48(46) |
| Zimbabwe | 0.399 | 2.31(0.37,5.64) | 0.003 | 2.302 | 131(131) | 0.474 | 2.46(0.45,5.44) | 0.003 | 2.457 | 142(142) |

**Abbreviations: ASR, age-standardised rate;** **SDI: sociodemographic index.**

**Table S9. Age-standardised DALYs rates of elderly-onset rheumatoid arthritis with frontier analysis across 204 countries and territories in 1990 and 2021.**

| **Location** | **SDI** | **ASRs** | **Frontier** | **Effective difference** | **Effective difference rank (ASRs rank)** | **SDI** | **ASRs** | **Frontier** | **Effective difference** | **Effective difference rank (ASRs rank)** |
| --- | --- | --- | --- | --- | --- | --- | --- | --- | --- | --- |
| Afghanistan | 0.174 | 34.39(17.70,61.53) | 16.162 | 18.231 | 69(70) | 0.337 | 54.20(28.41,95.04) | 12.545 | 41.659 | 91(91) |
| Albania | 0.558 | 74.05(44.64,114.01) | 11.557 | 62.498 | 123(123) | 0.707 | 75.10(46.66,111.89) | 11.558 | 63.544 | 115(115) |
| Algeria | 0.460 | 20.63(11.11,33.64) | 11.561 | 9.068 | 43(38) | 0.660 | 30.88(18.82,46.31) | 11.545 | 19.337 | 47(45) |
| American Samoa | 0.614 | 19.77(0.00,208.01) | 11.557 | 8.214 | 40(31) | 0.724 | 23.00(0.13,127.04) | 11.527 | 11.477 | 23(21) |
| Andorra | 0.761 | 59.67(8.12,180.80) | 11.538 | 48.132 | 111(111) | 0.869 | 58.93(16.14,136.54) | 11.567 | 47.364 | 98(98) |
| Angola | 0.271 | 41.22(20.54,105.78) | 16.119 | 25.097 | 83(89) | 0.454 | 50.14(28.08,94.76) | 11.540 | 38.604 | 89(89) |
| Antigua and Barbuda | 0.612 | 87.29(20.81,222.85) | 11.563 | 75.731 | 129(129) | 0.750 | 99.37(36.37,205.01) | 11.551 | 87.817 | 129(129) |
| Argentina | 0.587 | 93.00(69.92,120.82) | 11.560 | 81.445 | 136(136) | 0.723 | 113.94(85.28,149.69) | 11.531 | 102.413 | 139(139) |
| Armenia | 0.544 | 23.62(10.12,43.94) | 11.573 | 12.046 | 53(51) | 0.702 | 45.17(26.53,70.77) | 11.571 | 33.603 | 82(81) |
| Australia | 0.726 | 281.71(217.99,358.83) | 11.526 | 270.185 | 195(195) | 0.844 | 244.11(178.93,323.17) | 11.532 | 232.573 | 194(194) |
| Austria | 0.750 | 189.04(140.65,244.51) | 11.549 | 177.488 | 185(185) | 0.854 | 140.45(98.61,191.94) | 11.526 | 128.924 | 158(158) |
| Azerbaijan | 0.596 | 19.62(8.66,35.91) | 11.570 | 8.055 | 39(29) | 0.695 | 25.42(13.26,42.12) | 11.533 | 13.891 | 34(33) |
| Bahamas | 0.694 | 89.81(35.42,175.70) | 11.573 | 78.236 | 131(130) | 0.805 | 94.10(49.71,157.22) | 11.545 | 82.551 | 126(126) |
| Bahrain | 0.585 | 45.66(9.95,114.32) | 11.539 | 34.120 | 96(95) | 0.753 | 96.81(54.17,156.32) | 11.541 | 85.270 | 127(127) |
| Bangladesh | 0.229 | 133.63(80.69,207.71) | 16.128 | 117.505 | 152(153) | 0.492 | 116.11(75.25,171.84) | 11.524 | 104.582 | 140(140) |
| Barbados | 0.654 | 145.37(82.75,230.27) | 11.560 | 133.813 | 165(165) | 0.747 | 169.77(103.91,254.57) | 11.532 | 158.236 | 174(174) |
| Belarus | 0.622 | 28.51(15.42,46.91) | 11.572 | 16.937 | 66(63) | 0.784 | 44.98(26.25,69.16) | 11.558 | 33.425 | 81(80) |
| Belgium | 0.737 | 162.48(119.26,215.12) | 11.543 | 150.939 | 174(173) | 0.854 | 147.27(103.50,199.26) | 11.550 | 135.718 | 163(163) |
| Belize | 0.424 | 71.78(20.37,169.01) | 11.575 | 60.203 | 121(121) | 0.610 | 90.76(44.46,158.97) | 11.532 | 79.225 | 124(124) |
| Benin | 0.219 | 19.44(7.79,38.28) | 16.151 | 3.289 | 17(28) | 0.373 | 23.70(10.97,41.24) | 12.152 | 11.552 | 25(24) |
| Bermuda | 0.696 | 90.92(23.36,223.36) | 11.571 | 79.350 | 132(131) | 0.821 | 111.32(45.35,215.76) | 11.538 | 99.778 | 136(136) |
| Bhutan | 0.215 | 162.81(67.59,299.53) | 16.139 | 146.672 | 171(174) | 0.473 | 189.40(100.59,309.19) | 11.524 | 177.877 | 181(181) |
| Bolivia (Plurinational State of) | 0.424 | 168.03(112.34,241.63) | 11.573 | 156.455 | 175(175) | 0.599 | 205.00(140.09,280.49) | 11.533 | 193.464 | 188(188) |
| Bosnia and Herzegovina | 0.541 | 102.58(67.38,147.49) | 11.540 | 91.038 | 142(142) | 0.723 | 120.18(80.20,172.32) | 11.542 | 108.641 | 144(144) |
| Botswana | 0.418 | 106.40(52.02,179.55) | 11.594 | 94.806 | 146(145) | 0.643 | 101.82(55.44,159.72) | 11.532 | 90.291 | 131(131) |
| Brazil | 0.500 | 68.01(52.90,86.12) | 11.530 | 56.480 | 116(116) | 0.653 | 66.43(51.99,83.81) | 11.548 | 54.879 | 109(109) |
| Brunei Darussalam | 0.666 | 228.21(94.39,436.62) | 11.524 | 216.684 | 191(191) | 0.810 | 193.74(103.26,320.72) | 11.524 | 182.212 | 183(183) |
| Bulgaria | 0.633 | 45.39(30.31,65.63) | 11.550 | 33.841 | 95(94) | 0.768 | 47.84(30.47,70.46) | 11.560 | 36.279 | 85(85) |
| Burkina Faso | 0.130 | 19.65(8.61,35.25) | 16.169 | 3.484 | 20(30) | 0.285 | 22.72(11.26,39.24) | 16.168 | 6.553 | 8(20) |
| Burundi | 0.206 | 35.08(15.74,82.54) | 16.129 | 18.955 | 72(73) | 0.289 | 35.93(18.23,65.86) | 16.147 | 19.781 | 49(58) |
| Cabo Verde | 0.277 | 19.22(2.33,57.73) | 16.177 | 3.039 | 16(26) | 0.534 | 27.45(7.22,63.00) | 11.545 | 15.906 | 39(38) |
| Cambodia | 0.289 | 53.17(28.39,82.96) | 16.150 | 37.025 | 100(102) | 0.474 | 63.47(38.97,92.51) | 11.577 | 51.892 | 105(105) |
| Cameroon | 0.303 | 22.57(10.13,40.49) | 16.149 | 6.418 | 33(48) | 0.480 | 26.98(13.80,44.69) | 11.532 | 15.446 | 37(36) |
| Canada | 0.782 | 195.54(150.82,247.33) | 11.536 | 184.006 | 187(187) | 0.873 | 198.34(145.25,262.62) | 11.559 | 186.778 | 184(184) |
| Central African Republic | 0.217 | 45.81(19.79,135.00) | 16.120 | 29.692 | 91(97) | 0.309 | 43.69(20.48,103.61) | 15.044 | 28.648 | 67(76) |
| Chad | 0.115 | 17.22(6.78,32.85) | 16.157 | 1.061 | 7(14) | 0.240 | 18.51(8.49,32.89) | 16.164 | 2.344 | 4(5) |
| Chile | 0.586 | 188.78(145.02,239.69) | 11.533 | 177.247 | 184(184) | 0.772 | 223.97(163.00,293.74) | 11.551 | 212.415 | 191(191) |
| China | 0.459 | 148.13(116.92,188.97) | 11.550 | 136.579 | 168(168) | 0.722 | 149.93(114.52,191.46) | 11.533 | 138.396 | 165(165) |
| Colombia | 0.481 | 138.57(108.69,172.82) | 11.547 | 127.020 | 159(158) | 0.655 | 141.84(106.25,184.41) | 11.529 | 130.308 | 159(159) |
| Comoros | 0.270 | 36.86(7.40,106.11) | 16.158 | 20.706 | 76(76) | 0.476 | 43.39(15.06,96.85) | 11.563 | 31.828 | 76(75) |
| Congo | 0.421 | 55.41(25.22,150.48) | 11.590 | 43.817 | 106(106) | 0.583 | 63.93(34.28,121.54) | 11.568 | 52.362 | 107(107) |
| Cook Islands | 0.565 | 14.41(0.00,297.75) | 11.560 | 2.848 | 13(4) | 0.779 | 19.27(0.00,164.46) | 11.564 | 7.707 | 10(6) |
| Costa Rica | 0.534 | 170.39(121.25,232.31) | 11.533 | 158.857 | 178(178) | 0.700 | 225.66(163.61,299.21) | 11.531 | 214.125 | 192(192) |
| Côte d'Ivoire | 0.279 | 20.14(8.95,36.55) | 16.162 | 3.976 | 22(33) | 0.426 | 25.04(12.77,42.13) | 11.533 | 13.509 | 32(30) |
| Croatia | 0.669 | 123.89(89.89,165.89) | 11.524 | 112.367 | 149(149) | 0.798 | 152.34(106.01,210.36) | 11.529 | 140.807 | 166(166) |
| Cuba | 0.558 | 91.93(66.00,122.08) | 11.554 | 80.379 | 135(135) | 0.669 | 119.36(87.23,159.62) | 11.561 | 107.797 | 141(141) |
| Cyprus | 0.648 | 336.18(213.29,497.51) | 11.548 | 324.630 | 197(197) | 0.836 | 273.74(179.33,388.93) | 11.566 | 262.172 | 198(198) |
| Czechia | 0.682 | 70.31(50.77,95.35) | 11.524 | 58.788 | 120(120) | 0.828 | 75.45(51.93,104.97) | 11.539 | 63.913 | 116(116) |
| Democratic People's Republic of Korea | 0.498 | 143.13(98.94,198.94) | 11.538 | 131.587 | 162(161) | 0.570 | 171.84(120.56,234.84) | 11.524 | 160.313 | 175(175) |
| Democratic Republic of the Congo | 0.290 | 40.11(21.97,100.50) | 16.116 | 23.998 | 82(88) | 0.383 | 45.21(26.55,92.88) | 12.154 | 33.052 | 80(82) |
| Denmark | 0.801 | 235.83(174.27,308.97) | 11.556 | 224.271 | 192(192) | 0.896 | 277.13(199.56,367.59) | 11.524 | 265.603 | 199(199) |
| Djibouti | 0.338 | 34.88(4.15,117.82) | 12.545 | 22.332 | 78(72) | 0.488 | 42.49(15.90,88.89) | 11.540 | 30.949 | 73(72) |
| Dominica | 0.564 | 91.00(21.04,230.17) | 11.524 | 79.478 | 133(132) | 0.747 | 89.73(25.59,206.90) | 11.573 | 78.155 | 123(123) |
| Dominican Republic | 0.443 | 73.40(41.66,112.04) | 11.545 | 61.858 | 122(122) | 0.619 | 78.76(49.09,116.95) | 11.564 | 67.199 | 119(119) |
| Ecuador | 0.518 | 182.38(140.08,231.09) | 11.552 | 170.828 | 181(181) | 0.661 | 206.67(152.40,271.10) | 11.539 | 195.131 | 189(189) |
| Egypt | 0.417 | 28.16(17.48,41.32) | 11.602 | 16.562 | 65(62) | 0.607 | 35.99(22.68,51.84) | 11.568 | 24.423 | 60(59) |
| El Salvador | 0.373 | 58.95(34.89,91.43) | 12.150 | 46.801 | 110(110) | 0.564 | 74.99(47.23,110.98) | 11.550 | 63.439 | 114(114) |
| Equatorial Guinea | 0.269 | 42.68(8.84,166.22) | 16.141 | 26.540 | 88(91) | 0.658 | 69.62(29.36,144.05) | 11.524 | 58.097 | 110(110) |
| Eritrea | 0.216 | 35.94(13.43,87.18) | 16.133 | 19.803 | 74(74) | 0.404 | 41.60(19.86,81.08) | 12.148 | 29.449 | 69(70) |
| Estonia | 0.675 | 184.76(132.05,249.07) | 11.553 | 173.209 | 183(183) | 0.845 | 198.68(143.65,265.47) | 11.544 | 187.132 | 185(185) |
| Eswatini | 0.399 | 143.22(57.50,254.53) | 12.149 | 131.073 | 161(163) | 0.585 | 136.26(57.48,238.63) | 11.532 | 124.728 | 154(154) |
| Ethiopia | 0.148 | 39.46(23.72,74.82) | 16.195 | 23.268 | 80(84) | 0.359 | 33.83(20.73,55.42) | 12.265 | 21.563 | 56(55) |
| Fiji | 0.535 | 12.09(1.15,39.02) | 11.524 | 0.568 | 6(2) | 0.675 | 14.05(3.22,34.41) | 11.536 | 2.516 | 5(3) |
| Finland | 0.756 | 411.25(323.08,515.66) | 11.553 | 399.702 | 202(202) | 0.860 | 334.80(245.12,440.13) | 11.558 | 323.241 | 203(203) |
| France | 0.731 | 155.19(119.26,199.32) | 11.539 | 143.653 | 170(170) | 0.838 | 139.98(100.05,188.50) | 11.543 | 128.432 | 157(157) |
| Gabon | 0.455 | 56.84(23.10,144.86) | 11.535 | 45.306 | 107(107) | 0.635 | 73.82(35.70,138.05) | 11.559 | 62.266 | 112(112) |
| Gambia | 0.239 | 20.16(3.50,54.13) | 16.119 | 4.045 | 23(34) | 0.410 | 24.58(8.13,50.83) | 12.149 | 12.431 | 27(27) |
| Georgia | 0.740 | 24.58(11.45,42.76) | 11.563 | 13.021 | 60(59) | 0.847 | 33.08(18.65,53.14) | 11.525 | 21.554 | 55(51) |
| Germany | 0.817 | 143.72(108.80,185.39) | 11.533 | 132.188 | 164(164) | 0.903 | 125.35(91.05,167.37) | 11.569 | 113.782 | 147(147) |
| Ghana | 0.373 | 18.22(8.13,32.84) | 12.148 | 6.067 | 31(19) | 0.565 | 28.61(14.65,48.50) | 11.554 | 17.056 | 41(40) |
| Greece | 0.674 | 86.55(58.17,120.31) | 11.559 | 74.988 | 128(128) | 0.792 | 97.32(66.45,134.34) | 11.547 | 85.770 | 128(128) |
| Greenland | 0.732 | 112.70(15.71,351.17) | 11.524 | 101.176 | 147(147) | 0.826 | 138.33(44.09,301.62) | 11.524 | 126.803 | 156(156) |
| Grenada | 0.437 | 47.95(7.81,139.77) | 11.550 | 36.398 | 99(98) | 0.669 | 55.34(13.92,135.40) | 11.556 | 43.781 | 94(94) |
| Guam | 0.676 | 17.86(0.19,90.36) | 11.569 | 6.286 | 32(16) | 0.804 | 22.55(3.22,63.79) | 11.534 | 11.011 | 20(18) |
| Guatemala | 0.312 | 91.07(66.59,122.33) | 14.662 | 76.410 | 130(133) | 0.540 | 132.53(99.71,173.12) | 11.564 | 120.966 | 152(152) |
| Guinea | 0.178 | 18.50(7.83,34.43) | 16.160 | 2.338 | 10(21) | 0.336 | 20.97(9.87,37.48) | 12.552 | 8.419 | 12(12) |
| Guinea-Bissau | 0.208 | 18.93(3.48,50.39) | 16.118 | 2.816 | 12(23) | 0.353 | 22.46(6.06,50.80) | 12.341 | 10.122 | 19(17) |
| Guyana | 0.460 | 21.84(4.79,55.37) | 11.533 | 10.308 | 48(45) | 0.651 | 43.29(20.02,78.19) | 11.577 | 31.710 | 75(74) |
| Haiti | 0.310 | 85.79(45.62,151.67) | 14.923 | 70.866 | 126(126) | 0.448 | 82.82(46.04,141.67) | 11.527 | 71.296 | 122(122) |
| Honduras | 0.332 | 212.64(129.31,336.54) | 12.549 | 200.087 | 189(189) | 0.513 | 303.82(209.52,418.71) | 11.552 | 292.265 | 202(202) |
| Hungary | 0.649 | 146.76(115.17,186.31) | 11.552 | 135.207 | 167(167) | 0.791 | 128.65(93.45,171.08) | 11.572 | 117.076 | 150(150) |
| Iceland | 0.764 | 158.48(88.89,254.79) | 11.573 | 146.908 | 172(171) | 0.876 | 144.58(82.80,226.39) | 11.528 | 133.052 | 161(161) |
| India | 0.333 | 169.39(124.70,218.36) | 12.547 | 156.843 | 176(176) | 0.575 | 200.39(154.45,258.99) | 11.550 | 188.842 | 186(186) |
| Indonesia | 0.457 | 24.17(15.32,34.27) | 11.568 | 12.604 | 59(58) | 0.657 | 30.46(19.56,41.50) | 11.566 | 18.893 | 45(43) |
| Iran (Islamic Republic of) | 0.454 | 23.42(15.22,33.88) | 11.552 | 11.870 | 52(50) | 0.697 | 30.91(21.63,42.92) | 11.567 | 19.342 | 48(46) |
| Iraq | 0.412 | 23.83(12.90,38.52) | 11.721 | 12.108 | 55(55) | 0.663 | 32.06(19.20,49.16) | 11.573 | 20.492 | 51(48) |
| Ireland | 0.720 | 430.09(328.88,547.28) | 11.541 | 418.550 | 203(203) | 0.874 | 381.30(272.66,508.07) | 11.561 | 369.743 | 204(204) |
| Israel | 0.709 | 91.51(61.63,131.22) | 11.545 | 79.970 | 134(134) | 0.809 | 103.98(69.35,146.04) | 11.552 | 92.433 | 132(132) |
| Italy | 0.706 | 170.15(128.78,219.92) | 11.581 | 158.566 | 177(177) | 0.806 | 160.18(120.09,208.20) | 11.525 | 148.654 | 170(170) |
| Jamaica | 0.535 | 63.14(38.95,94.57) | 11.551 | 51.589 | 114(114) | 0.683 | 81.09(53.58,115.93) | 11.577 | 69.510 | 120(121) |
| Japan | 0.790 | 281.46(223.39,352.20) | 11.538 | 269.922 | 194(194) | 0.871 | 203.51(156.37,259.05) | 11.527 | 191.980 | 187(187) |
| Jordan | 0.539 | 24.10(9.77,47.60) | 11.524 | 12.580 | 58(57) | 0.725 | 33.73(18.77,53.41) | 11.559 | 22.171 | 58(54) |
| Kazakhstan | 0.589 | 23.38(11.75,39.70) | 11.557 | 11.826 | 51(49) | 0.725 | 48.46(32.26,69.34) | 11.555 | 36.909 | 86(87) |
| Kenya | 0.334 | 37.69(22.41,64.81) | 12.551 | 25.137 | 84(81) | 0.524 | 40.51(25.51,65.41) | 11.549 | 28.958 | 68(67) |
| Kiribati | 0.410 | 11.67(0.00,124.46) | 11.669 | 0.000 | 1(1) | 0.527 | 13.71(0.03,82.89) | 11.577 | 2.134 | 2(1) |
| Kuwait | 0.665 | 37.25(13.65,75.26) | 11.532 | 25.716 | 87(80) | 0.847 | 62.60(37.08,96.18) | 11.545 | 51.050 | 103(104) |
| Kyrgyzstan | 0.519 | 103.66(64.86,152.93) | 11.561 | 92.102 | 143(143) | 0.604 | 163.17(111.63,231.28) | 11.541 | 151.628 | 171(171) |
| Lao People's Democratic Republic | 0.264 | 53.20(26.35,89.08) | 16.133 | 37.067 | 101(103) | 0.489 | 54.99(30.51,85.21) | 11.540 | 43.445 | 92(92) |
| Latvia | 0.680 | 136.13(97.17,180.27) | 11.525 | 124.609 | 156(155) | 0.831 | 147.49(106.54,199.15) | 11.555 | 135.931 | 164(164) |
| Lebanon | 0.537 | 37.15(19.20,60.71) | 11.569 | 25.583 | 85(78) | 0.745 | 41.04(22.66,65.42) | 11.542 | 29.499 | 70(68) |
| Lesotho | 0.339 | 106.61(55.84,177.46) | 12.550 | 94.061 | 145(146) | 0.510 | 125.84(59.26,202.42) | 11.576 | 114.269 | 148(148) |
| Liberia | 0.235 | 19.13(6.57,39.72) | 16.131 | 3.004 | 14(25) | 0.352 | 23.74(9.36,46.09) | 12.348 | 11.396 | 22(25) |
| Libya | 0.528 | 23.67(10.40,43.66) | 11.577 | 12.098 | 54(53) | 0.726 | 39.13(21.77,62.45) | 11.556 | 27.571 | 64(64) |
| Lithuania | 0.669 | 159.42(117.81,208.70) | 11.561 | 147.854 | 173(172) | 0.856 | 187.17(138.98,241.37) | 11.558 | 175.611 | 179(180) |
| Luxembourg | 0.781 | 139.28(84.56,210.57) | 11.550 | 127.733 | 160(160) | 0.884 | 123.78(74.00,189.87) | 11.539 | 112.243 | 146(146) |
| Madagascar | 0.280 | 29.27(14.27,59.47) | 16.160 | 13.106 | 61(65) | 0.400 | 33.63(18.26,57.99) | 12.151 | 21.480 | 54(53) |
| Malawi | 0.204 | 36.60(18.64,77.07) | 16.155 | 20.443 | 75(75) | 0.385 | 43.99(24.35,80.66) | 12.150 | 31.836 | 77(77) |
| Malaysia | 0.546 | 23.74(13.22,39.15) | 11.524 | 12.214 | 57(54) | 0.743 | 25.16(14.73,38.51) | 11.562 | 13.599 | 33(32) |
| Maldives | 0.332 | 58.90(11.64,160.74) | 12.550 | 46.352 | 109(109) | 0.651 | 61.70(23.54,121.76) | 11.532 | 50.166 | 102(102) |
| Mali | 0.127 | 17.48(7.47,32.01) | 16.184 | 1.298 | 8(15) | 0.269 | 20.40(9.84,34.82) | 16.126 | 4.270 | 7(9) |
| Malta | 0.657 | 129.97(74.96,201.20) | 11.547 | 118.427 | 153(152) | 0.802 | 119.42(71.89,182.79) | 11.580 | 107.836 | 142(142) |
| Marshall Islands | 0.431 | 16.13(0.00,250.72) | 11.561 | 4.571 | 25(9) | 0.574 | 21.05(0.01,155.98) | 11.524 | 9.524 | 17(14) |
| Mauritania | 0.336 | 22.42(7.31,46.94) | 12.550 | 9.872 | 45(47) | 0.499 | 30.22(13.28,54.43) | 11.561 | 18.661 | 43(42) |
| Mauritius | 0.545 | 27.74(9.56,56.92) | 11.561 | 16.182 | 64(61) | 0.718 | 71.97(46.61,104.94) | 11.569 | 60.397 | 111(111) |
| Mexico | 0.505 | 352.64(294.91,420.95) | 11.534 | 341.107 | 198(198) | 0.665 | 300.53(242.40,370.81) | 11.571 | 288.956 | 201(201) |
| Micronesia (Federated States of) | 0.463 | 16.91(0.02,110.01) | 11.535 | 5.378 | 28(12) | 0.588 | 24.94(0.77,105.70) | 11.545 | 13.395 | 31(28) |
| Monaco | 0.845 | 52.92(7.36,158.21) | 11.552 | 41.365 | 103(101) | 0.908 | 55.37(10.59,148.55) | 11.566 | 43.800 | 95(95) |
| Mongolia | 0.467 | 45.77(21.17,99.46) | 11.552 | 34.221 | 97(96) | 0.618 | 76.70(44.27,117.85) | 11.524 | 65.175 | 117(117) |
| Montenegro | 0.674 | 64.94(32.27,111.81) | 11.534 | 53.402 | 115(115) | 0.796 | 76.81(42.70,123.65) | 11.528 | 65.285 | 118(118) |
| Morocco | 0.358 | 23.84(12.74,41.21) | 12.280 | 11.561 | 50(56) | 0.563 | 37.91(21.07,61.88) | 11.534 | 26.378 | 62(61) |
| Mozambique | 0.173 | 33.97(16.99,72.26) | 16.139 | 17.835 | 68(69) | 0.326 | 40.29(22.57,73.74) | 12.546 | 27.741 | 65(66) |
| Myanmar | 0.319 | 48.58(28.58,71.51) | 12.582 | 36.001 | 98(99) | 0.534 | 55.19(35.15,79.02) | 11.573 | 43.613 | 93(93) |
| Namibia | 0.450 | 105.24(52.01,179.74) | 11.549 | 93.687 | 144(144) | 0.618 | 107.49(56.01,177.56) | 11.574 | 95.916 | 133(133) |
| Nauru | 0.539 | 19.13(0.00,800.81) | 11.529 | 7.606 | 37(24) | 0.625 | 25.93(0.00,661.71) | 11.547 | 14.385 | 36(35) |
| Nepal | 0.200 | 139.14(86.44,216.20) | 16.150 | 122.989 | 154(159) | 0.433 | 168.12(110.66,244.25) | 11.558 | 156.564 | 172(172) |
| Netherlands | 0.795 | 366.76(285.33,459.54) | 11.533 | 355.228 | 200(200) | 0.888 | 272.03(200.38,358.42) | 11.541 | 260.493 | 197(197) |
| New Zealand | 0.752 | 359.69(265.70,472.40) | 11.577 | 348.109 | 199(199) | 0.849 | 271.96(189.34,371.53) | 11.547 | 260.414 | 196(196) |
| Nicaragua | 0.346 | 129.42(81.68,194.37) | 12.539 | 116.879 | 151(151) | 0.524 | 154.03(106.39,211.78) | 11.561 | 142.473 | 167(167) |
| Niger | 0.081 | 16.78(6.63,33.27) | 16.779 | 0.000 | 2(10) | 0.168 | 19.32(9.12,33.48) | 16.133 | 3.183 | 6(7) |
| Nigeria | 0.306 | 18.34(11.50,27.52) | 14.968 | 3.368 | 18(20) | 0.503 | 21.27(13.52,31.29) | 11.525 | 9.741 | 18(15) |
| Niue | 0.588 | 17.02(0.00,1379.13) | 11.525 | 5.491 | 29(13) | 0.726 | 23.17(0.00,1383.54) | 11.541 | 11.631 | 26(23) |
| North Macedonia | 0.609 | 38.83(20.15,66.99) | 11.569 | 27.257 | 89(83) | 0.751 | 47.44(26.64,77.38) | 11.551 | 35.888 | 84(84) |
| Northern Mariana Islands | 0.709 | 22.25(0.00,320.30) | 11.545 | 10.706 | 49(46) | 0.772 | 25.62(0.31,128.48) | 11.566 | 14.057 | 35(34) |
| Norway | 0.796 | 442.24(343.51,561.51) | 11.525 | 430.713 | 204(204) | 0.916 | 267.30(191.04,362.06) | 11.525 | 255.779 | 195(195) |
| Oman | 0.429 | 20.30(6.05,46.10) | 11.532 | 8.766 | 42(36) | 0.773 | 31.56(14.54,56.00) | 11.525 | 20.038 | 50(47) |
| Pakistan | 0.310 | 175.84(118.48,244.54) | 14.999 | 160.844 | 179(179) | 0.504 | 176.03(115.12,249.99) | 11.540 | 164.493 | 176(176) |
| Palau | 0.663 | 21.12(0.00,373.95) | 11.528 | 9.594 | 44(40) | 0.754 | 27.34(0.01,209.10) | 11.571 | 15.766 | 38(37) |
| Palestine | 0.402 | 31.60(13.00,59.99) | 12.151 | 19.451 | 73(67) | 0.631 | 36.48(19.01,62.28) | 11.527 | 24.952 | 61(60) |
| Panama | 0.546 | 97.79(65.18,140.00) | 11.530 | 86.258 | 140(140) | 0.709 | 113.63(77.11,156.27) | 11.524 | 102.104 | 138(138) |
| Papua New Guinea | 0.311 | 12.54(4.23,26.19) | 12.545 | 0.000 | 3(3) | 0.418 | 13.84(5.50,26.22) | 11.613 | 2.225 | 3(2) |
| Paraguay | 0.470 | 86.47(53.79,131.41) | 11.565 | 74.906 | 127(127) | 0.636 | 157.55(106.44,219.22) | 11.578 | 145.967 | 169(169) |
| Peru | 0.510 | 143.20(95.35,197.57) | 11.527 | 131.669 | 163(162) | 0.662 | 183.25(127.58,250.66) | 11.561 | 171.693 | 178(178) |
| Philippines | 0.510 | 70.10(47.77,92.70) | 11.535 | 58.564 | 118(118) | 0.651 | 62.58(46.01,80.60) | 11.524 | 51.059 | 104(103) |
| Poland | 0.627 | 238.20(201.67,281.49) | 11.553 | 226.648 | 193(193) | 0.812 | 136.93(106.65,173.31) | 11.524 | 125.401 | 155(155) |
| Portugal | 0.600 | 134.87(101.41,173.64) | 11.537 | 123.331 | 155(154) | 0.744 | 145.34(104.32,193.52) | 11.526 | 133.811 | 162(162) |
| Puerto Rico | 0.659 | 97.71(67.03,135.51) | 11.561 | 86.148 | 139(139) | 0.826 | 119.84(82.10,165.25) | 11.565 | 108.276 | 143(143) |
| Qatar | 0.651 | 27.24(1.25,108.62) | 11.536 | 15.707 | 63(60) | 0.847 | 42.67(17.10,82.15) | 11.554 | 31.113 | 74(73) |
| Republic of Korea | 0.692 | 148.67(106.93,206.31) | 11.564 | 137.105 | 169(169) | 0.887 | 128.86(89.01,175.33) | 11.558 | 117.304 | 151(151) |
| Republic of Moldova | 0.604 | 43.50(26.97,65.91) | 11.541 | 31.961 | 93(92) | 0.732 | 63.69(42.72,90.57) | 11.552 | 52.139 | 106(106) |
| Romania | 0.619 | 28.76(16.04,46.13) | 11.536 | 17.220 | 67(64) | 0.768 | 44.46(26.33,69.29) | 11.556 | 32.905 | 79(79) |
| Russian Federation | 0.672 | 97.92(80.94,118.77) | 11.552 | 86.371 | 141(141) | 0.809 | 108.55(90.35,131.57) | 11.578 | 96.967 | 135(135) |
| Rwanda | 0.275 | 38.50(18.52,88.79) | 16.119 | 22.379 | 79(82) | 0.436 | 48.44(26.42,83.65) | 11.524 | 36.919 | 87(86) |
| Saint Kitts and Nevis | 0.581 | 116.20(28.34,289.35) | 11.556 | 104.649 | 148(148) | 0.755 | 128.15(43.65,270.25) | 11.524 | 116.628 | 149(149) |
| Saint Lucia | 0.496 | 96.65(34.22,203.30) | 11.569 | 85.083 | 138(138) | 0.673 | 113.26(57.03,193.31) | 11.524 | 101.740 | 137(137) |
| Saint Vincent and the Grenadines | 0.476 | 53.37(10.39,147.09) | 11.569 | 41.799 | 104(104) | 0.637 | 49.29(13.59,115.48) | 11.539 | 37.749 | 88(88) |
| Samoa | 0.487 | 18.08(0.33,83.83) | 11.542 | 6.540 | 36(18) | 0.593 | 23.08(2.09,76.11) | 11.560 | 11.523 | 24(22) |
| San Marino | 0.813 | 70.25(6.94,238.72) | 11.566 | 58.687 | 119(119) | 0.888 | 59.13(9.94,168.05) | 11.564 | 47.568 | 99(99) |
| Sao Tome and Principe | 0.310 | 21.41(0.33,102.37) | 14.965 | 6.444 | 35(41) | 0.505 | 28.39(2.10,99.52) | 11.533 | 16.860 | 40(39) |
| Saudi Arabia | 0.539 | 21.64(11.06,36.73) | 11.524 | 10.116 | 47(43) | 0.815 | 32.95(19.44,50.38) | 11.549 | 21.398 | 53(50) |
| Senegal | 0.238 | 20.41(8.66,37.37) | 16.135 | 4.274 | 24(37) | 0.408 | 25.04(12.42,43.14) | 12.148 | 12.891 | 29(29) |
| Serbia | 0.631 | 80.55(54.52,114.17) | 11.536 | 69.011 | 125(125) | 0.792 | 91.93(62.68,127.72) | 11.545 | 80.388 | 125(125) |
| Seychelles | 0.576 | 20.27(0.27,103.54) | 11.531 | 8.736 | 41(35) | 0.730 | 24.06(1.84,82.94) | 11.560 | 12.502 | 28(26) |
| Sierra Leone | 0.212 | 18.79(7.24,36.85) | 16.129 | 2.664 | 11(22) | 0.359 | 21.58(9.40,39.33) | 12.266 | 9.314 | 15(16) |
| Singapore | 0.686 | 63.02(38.65,96.66) | 11.561 | 51.456 | 113(113) | 0.856 | 60.56(36.49,92.86) | 11.562 | 49.001 | 100(100) |
| Slovakia | 0.654 | 62.52(40.76,89.90) | 11.532 | 50.984 | 112(112) | 0.811 | 65.18(42.96,94.32) | 11.533 | 53.647 | 108(108) |
| Slovenia | 0.727 | 201.12(148.18,265.37) | 11.553 | 189.564 | 188(188) | 0.842 | 191.63(132.00,266.00) | 11.530 | 180.103 | 182(182) |
| Solomon Islands | 0.301 | 14.92(0.45,63.74) | 14.921 | 0.000 | 4(6) | 0.429 | 19.71(3.26,53.63) | 11.524 | 8.185 | 11(8) |
| Somalia | 0.049 | 39.59(18.21,89.79) | 39.586 | 0.000 | 5(86) | 0.078 | 38.80(20.13,80.59) | 38.199 | 0.596 | 1(63) |
| South Africa | 0.542 | 180.58(133.49,234.10) | 11.546 | 169.038 | 180(180) | 0.680 | 155.89(119.60,199.48) | 11.551 | 144.339 | 168(168) |
| South Sudan | 0.207 | 31.60(14.79,66.06) | 16.135 | 15.465 | 62(66) | 0.278 | 34.88(17.17,65.86) | 16.141 | 18.738 | 44(56) |
| Spain | 0.637 | 137.59(104.92,176.04) | 11.530 | 126.056 | 158(157) | 0.769 | 134.81(96.59,181.97) | 11.564 | 123.243 | 153(153) |
| Sri Lanka | 0.523 | 17.99(10.00,29.26) | 11.552 | 6.440 | 34(17) | 0.702 | 22.63(12.55,35.94) | 11.542 | 11.093 | 21(19) |
| Sudan | 0.292 | 21.74(11.28,37.64) | 16.134 | 5.604 | 30(44) | 0.542 | 30.54(18.59,47.25) | 11.576 | 18.965 | 46(44) |
| Suriname | 0.502 | 37.17(10.05,87.97) | 11.556 | 25.609 | 86(79) | 0.634 | 38.46(15.66,75.75) | 11.558 | 26.898 | 63(62) |
| Sweden | 0.786 | 309.68(235.66,399.31) | 11.547 | 298.133 | 196(196) | 0.887 | 229.19(164.93,307.17) | 11.533 | 217.656 | 193(193) |
| Switzerland | 0.863 | 223.95(165.16,295.14) | 11.524 | 212.424 | 190(190) | 0.933 | 187.14(130.54,255.27) | 11.524 | 175.614 | 180(179) |
| Syrian Arab Republic | 0.430 | 23.65(12.76,38.58) | 11.530 | 12.119 | 56(52) | 0.623 | 35.48(21.67,52.89) | 11.525 | 23.959 | 59(57) |
| Taiwan (Province of China) | 0.668 | 137.24(103.73,177.83) | 11.541 | 125.698 | 157(156) | 0.875 | 120.57(89.32,158.85) | 11.532 | 109.043 | 145(145) |
| Tajikistan | 0.466 | 39.60(19.74,67.64) | 11.552 | 28.045 | 90(87) | 0.542 | 56.48(33.24,87.07) | 11.537 | 44.946 | 96(96) |
| Thailand | 0.507 | 57.44(37.24,88.05) | 11.524 | 45.920 | 108(108) | 0.683 | 60.80(41.20,86.88) | 11.552 | 49.249 | 101(101) |
| Timor-Leste | 0.262 | 39.54(10.07,93.02) | 16.134 | 23.403 | 81(85) | 0.445 | 51.00(22.85,90.27) | 11.545 | 39.452 | 90(90) |
| Togo | 0.270 | 20.02(6.66,42.32) | 16.147 | 3.876 | 21(32) | 0.409 | 25.09(11.14,45.14) | 12.148 | 12.942 | 30(31) |
| Tokelau | 0.522 | 14.96(0.00,1981.49) | 11.524 | 3.440 | 19(7) | 0.686 | 21.02(0.00,1978.29) | 11.527 | 9.491 | 16(13) |
| Tonga | 0.492 | 16.79(0.04,102.81) | 11.568 | 5.225 | 27(11) | 0.626 | 20.59(0.46,91.97) | 11.556 | 9.035 | 14(11) |
| Trinidad and Tobago | 0.624 | 145.50(99.10,204.45) | 11.576 | 133.926 | 166(166) | 0.769 | 142.61(97.79,197.28) | 11.529 | 131.085 | 160(160) |
| Tunisia | 0.471 | 21.44(10.85,35.87) | 11.535 | 9.909 | 46(42) | 0.682 | 32.47(19.29,50.27) | 11.554 | 20.917 | 52(49) |
| Turkey | 0.462 | 93.69(63.52,133.35) | 11.547 | 82.141 | 137(137) | 0.713 | 100.19(70.10,135.88) | 11.556 | 88.633 | 130(130) |
| Turkmenistan | 0.563 | 19.39(6.95,39.03) | 11.560 | 7.834 | 38(27) | 0.682 | 33.22(18.59,53.89) | 11.578 | 21.639 | 57(52) |
| Tuvalu | 0.406 | 15.16(0.00,489.10) | 12.148 | 3.015 | 15(8) | 0.577 | 20.56(0.00,341.75) | 11.553 | 9.005 | 13(10) |
| Uganda | 0.187 | 34.44(17.81,64.57) | 16.130 | 18.305 | 71(71) | 0.423 | 42.44(23.25,73.07) | 11.554 | 30.889 | 72(71) |
| Ukraine | 0.647 | 41.48(28.37,56.79) | 11.552 | 29.931 | 92(90) | 0.761 | 46.28(32.06,64.09) | 11.537 | 34.746 | 83(83) |
| United Arab Emirates | 0.644 | 44.84(14.82,97.79) | 11.556 | 33.287 | 94(93) | 0.849 | 40.03(21.23,66.15) | 11.553 | 28.481 | 66(65) |
| United Kingdom | 0.744 | 400.02(332.23,479.76) | 11.547 | 388.476 | 201(201) | 0.859 | 295.46(226.38,377.71) | 11.542 | 283.922 | 200(200) |
| United Republic of Tanzania | 0.259 | 37.05(19.78,70.72) | 16.169 | 20.884 | 77(77) | 0.447 | 44.11(25.74,72.59) | 11.550 | 32.560 | 78(78) |
| United States Virgin Islands | 0.655 | 55.13(9.74,155.23) | 11.563 | 43.564 | 105(105) | 0.822 | 57.21(18.03,126.44) | 11.536 | 45.672 | 97(97) |
| United States of America | 0.764 | 184.57(145.82,230.22) | 11.566 | 173.001 | 182(182) | 0.862 | 176.93(134.20,226.70) | 11.562 | 165.365 | 177(177) |
| Uruguay | 0.582 | 125.51(91.95,166.69) | 11.526 | 113.983 | 150(150) | 0.719 | 168.89(126.21,220.41) | 11.553 | 157.342 | 173(173) |
| Uzbekistan | 0.500 | 69.15(41.59,105.44) | 11.567 | 57.587 | 117(117) | 0.663 | 107.53(74.27,150.56) | 11.571 | 95.964 | 134(134) |
| Vanuatu | 0.353 | 14.62(0.02,94.50) | 12.318 | 2.301 | 9(5) | 0.473 | 18.16(1.35,63.52) | 11.575 | 6.586 | 9(4) |
| Venezuela (Bolivarian Republic of) | 0.517 | 193.03(150.69,242.41) | 11.538 | 181.494 | 186(186) | 0.597 | 210.76(158.81,272.46) | 11.537 | 199.226 | 190(190) |
| Viet Nam | 0.408 | 52.74(33.34,76.84) | 12.149 | 40.586 | 102(100) | 0.628 | 74.10(49.16,105.27) | 11.573 | 62.530 | 113(113) |
| Yemen | 0.216 | 21.01(10.75,37.18) | 16.147 | 4.861 | 26(39) | 0.450 | 29.40(17.22,46.60) | 11.547 | 17.854 | 42(41) |
| Zambia | 0.304 | 33.35(15.55,70.78) | 15.072 | 18.282 | 70(68) | 0.506 | 41.33(22.15,72.66) | 11.545 | 29.780 | 71(69) |
| Zimbabwe | 0.399 | 75.35(42.59,113.15) | 12.152 | 63.199 | 124(124) | 0.474 | 81.09(45.09,118.78) | 11.552 | 69.535 | 121(120) |

**Abbreviations: ASR, age-standardised rate; DALY=disability-adjusted life-years; SDI: sociodemographic index.**

**Table S10. Age-standardised mortality and DALYs rates attributable to smoking for elderly-onset rheumatoid arthritis globally and regionally in 1990 and 2021, along with their proportions.**

| **Location** | **Mortality** | | | | **DALYs** | | | |
| --- | --- | --- | --- | --- | --- | --- | --- | --- |
|  | **Number (95 % UI), 1990** | **ASRs,**  **proportion (%), 1990** | **Number (95 % UI), 2021** | **ASRs,**  **proportion (%), 2021** | **Number (95 % UI), 1990** | **ASRs,**  **proportion (%), 1990** | **Number (95 % UI), 2021** | **ASRs,**  **Proportion (%), 2021** |
| Global | 1405.5(1036.85,1776.44) | 0.29(7.85) | 1889.22(1223.29,2497.98) | 0.17(5.69) | 64931.67(47390.56,86772.7) | 13.35(9.07) | 100844.38(70615.61,137340.69) | 9.25(6.51) |
| **SDI regions** | | | | | | | | |
| Low SDI | 28.74(14.75,46.29) | 0.11(5.38) | 51.39(27.77,88.22) | 0.09(4.11) | 1041.32(668.61,1518.45) | 4.06(5.31) | 2114.34(1340.23,3215.38) | 3.73(4.09) |
| Low-middle SDI | 163.5(81.65,239.89) | 0.23(5.81) | 322.63(176.17,485.52) | 0.19(4.51) | 5490.99(3539.76,7708.06) | 7.88(6.20) | 11838.01(7815.9,16809.13) | 6.90(4.62) |
| Middle SDI | 323.13(197.35,433.88) | 0.27(8.28) | 638.43(326.25,901.88) | 0.19(6.36) | 13715.06(9678.83,18686.25) | 11.39(9.09) | 28935.04(19683.31,39998.58) | 8.72(6.52) |
| High-middle SDI | 221.48(161.44,295.52) | 0.18(7.36) | 402.85(262.87,560.26) | 0.16(6.52) | 12017.35(8583.42,16532.53) | 9.53(8.51) | 23400.9(16044.22,32335.11) | 9.11(7.47) |
| High SDI | 667.35(520.41,822.12) | 0.46(8.74) | 472.98(354.26,604.21) | 0.17(5.53) | 32607.97(23589.84,43123.97) | 22.66(10.36) | 34491.9(23577.92,48001.89) | 12.67(7.13) |
| **GBD regions** | | | | | | | | |
| East Asia | 349.48(210.67,486.77) | 0.34(9.90) | 776.39(383.72,1125.95) | 0.28(8.42) | 16340.93(11505.43,22590.89) | 15.72(10.63) | 36991.01(24810.81,52150.58) | 13.27(8.87) |
| South Asia | 385.7(187.4,572.61) | 0.30(5.59) | 755.97(407.16,1172.24) | 0.21(4.19) | 12780.16(8121.2,18292.45) | 9.89(5.94) | 28115.93(18150.11,40842.52) | 7.88(4.15) |
| Southeast Asia | 35.15(16.46,51.03) | 0.12(10.25) | 72.19(30.76,105.83) | 0.09(8.01) | 1225.01(772.17,1721.94) | 4.21(10.11) | 3011.47(1939.41,4201.38) | 3.78(7.77) |
| Central Asia | 0.45(0.31,0.66) | 0.01(4.63) | 3.34(2.38,4.45) | 0.03(3.64) | 99.96(62.73,149.57) | 1.76(4.31) | 308.46(205.92,444.84) | 3.12(4.49) |
| High-income Asia Pacific | 112.51(84.47,143.28) | 0.44(7.42) | 106.13(73.79,144.06) | 0.18(4.52) | 5731.79(4030.48,7803.29) | 22.55(8.61) | 5652.83(3707.64,8112.53) | 9.74(5.28) |
| Oceania | 0(0,0) | 0.00(4.93) | 0(0,0) | 0.00(4.84) | 3.29(1.85,5.43) | 1.01(7.58) | 8.43(4.7,13.83) | 1.05(6.88) |
| Australasia | 15.22(11.25,19.86) | 0.49(7.29) | 11.12(7.35,16.09) | 0.16(4.02) | 807.3(559.36,1112.15) | 26.12(8.86) | 903.34(563.74,1359.06) | 12.97(5.22) |
| Eastern Europe | 21.67(16.55,27.96) | 0.06(3.43) | 45.38(33.86,58.35) | 0.09(4.38) | 1243.23(886.6,1723.83) | 3.38(4.16) | 2355.79(1686.97,3195.02) | 4.88(5.21) |
| Western Europe | 362.16(279.82,449.06) | 0.48(8.71) | 192.4(143.23,243.97) | 0.16(5.22) | 16834.38(12121.62,22410.93) | 22.18(10.46) | 14772.07(10090.08,20700.77) | 12.55(7.12) |
| Central Europe | 48.89(37.43,61.85) | 0.25(8.17) | 26.8(19.85,34.98) | 0.09(5.91) | 2303.51(1702.08,3072.21) | 11.77(9.54) | 2349.88(1623.27,3307.64) | 7.85(7.80) |
| High-income North America | 169.83(129.63,213.31) | 0.37(9.81) | 153.22(109.41,206.71) | 0.17(6.51) | 9852.15(6980.67,13346.03) | 21.33(11.50) | 13051(8674.12,18629.93) | 14.80(8.26) |
| Andean Latin America | 3.92(2.63,5.47) | 0.17(2.92) | 5.54(3.45,8.51) | 0.08(1.93) | 122.04(83.92,168.98) | 5.14(3.27) | 342.19(217.8,499.24) | 4.75(2.46) |
| Central Latin America | 43.56(31.77,57.75) | 0.45(5.27) | 44.46(31.44,60.25) | 0.14(2.44) | 1384.88(992.02,1879.67) | 14.40(5.73) | 2025.35(1405.22,2843.48) | 6.54(2.80) |
| Caribbean | 4.75(3.24,6.78) | 0.15(4.81) | 7.45(4.87,10.98) | 0.11(3.52) | 168.53(110.32,243.86) | 5.26(5.99) | 316.17(202.56,468.44) | 4.71(4.55) |
| Tropical Latin America | 15.56(11,20.87) | 0.14(9.87) | 29.41(21.17,38.95) | 0.09(6.07) | 842.29(572.41,1194.18) | 7.82(11.42) | 1617.58(1117.49,2263.15) | 5.01(7.32) |
| Southern Latin America | 9.79(6.84,13.71) | 0.17(5.57) | 13.66(9.35,18.87) | 0.12(4.16) | 489.14(329.16,725.76) | 8.28(7.15) | 972.47(634.62,1404.81) | 8.67(5.78) |
| Eastern Sub-Saharan Africa | 0.59(0.18,2.47) | 0.01(3.07) | 0.5(0.17,2.84) | 0.00(1.97) | 123.67(80.53,186.61) | 1.47(4.04) | 229.83(146.56,341.66) | 1.25(3.17) |
| Southern Sub-Saharan Africa | 9.49(5.59,14.07) | 0.30(6.75) | 7.81(4.83,12.09) | 0.11(3.13) | 399.53(269.92,576.17) | 12.57(7.91) | 395.01(260.01,585.88) | 5.74(3.98) |
| Western Sub-Saharan Africa | 0.05(0.02,0.08) | 0.00(0.82) | 0.33(0.11,0.71) | 0.00(2.85) | 38.31(23.26,60.14) | 0.38(2.01) | 89.45(53.69,136.88) | 0.41(1.82) |
| North Africa and Middle East | 18.92(9.46,33.7) | 0.05(5.56) | 29.94(16.43,47.92) | 0.03(4.02) | 1005.47(654.1,1508.93) | 2.58(6.59) | 2646.55(1726.87,3778.1) | 2.52(5.14) |
| Central Sub-Saharan Africa | 0.11(0.03,0.44) | 0.00(1.35) | 0.12(0.04,0.63) | 0.00(0.92) | 28.91(17.77,45.97) | 1.16(2.76) | 70.8(42.09,111.62) | 1.21(2.53) |

**Abbreviations: ASR, age-standardised rate; DALY=disability-adjusted life-years; UI: uncertainly interval; GBD, global burden of disease; SDI: sociodemographic index.**

**Table S11. Age-standardised mortality rates and DALYs rates attributable to smoking for elderly-onset rheumatoid arthritis** **across 204 countries and territories in 1990 and 2021, along with their proportions.**

| **Location** | **Mortality** | | | | **DALYs** | | | |
| --- | --- | --- | --- | --- | --- | --- | --- | --- |
|  | **Number (95 % UI), 1990** | **ASRs,**  **proportion (%), 1990** | **Number (95 % UI), 2021** | **ASRs,**  **proportion (%), 2021** | **Number (95 % UI), 1990** | **ASRs,**  **proportion (%), 1990** | **Number (95 % UI), 2021** | **ASRs,**  **Proportion (%), 2021** |
| Afghanistan | 0.09(0.04,0.2) | 0.01(1.13) | 0.18(0.08,0.37) | 0.02(1.25) | 4.85(2.77,8.26) | 0.58(1.67) | 7.13(3.9,12.16) | 0.89(1.64) |
| Albania | 0.52(0.27,0.84) | 0.21(9.85) | 0.73(0.35,1.28) | 0.12(11.03) | 19.18(11.41,29.65) | 7.91(10.69) | 53.31(31.01,82.52) | 8.90(11.85) |
| Algeria | 0.11(0.04,0.23) | 0.01(2.70) | 0.53(0.22,1.17) | 0.01(3.03) | 14.04(7.82,23.11) | 0.95(4.59) | 50.11(27.94,81.03) | 1.18(3.82) |
| American Samoa | 0(0,0) | 0.00(3.58) | 0(0,0) | 0.00(3.24) | 0.04(0.02,0.06) | 1.43(7.24) | 0.09(0.04,0.16) | 1.55(6.74) |
| Andorra | 0(0,0) | 0.03(5.99) | 0(0,0.01) | 0.01(4.41) | 0.43(0.24,0.7) | 5.80(9.71) | 0.96(0.51,1.59) | 4.97(8.44) |
| Angola | 0.03(0.01,0.12) | 0.01(2.51) | 0.05(0.01,0.25) | 0.00(1.80) | 8.11(4.47,13.47) | 1.99(4.83) | 27.24(14.26,47.36) | 2.20(4.39) |
| Antigua and Barbuda | 0.01(0,0.01) | 0.08(2.42) | 0.01(0,0.01) | 0.06(2.07) | 0.18(0.11,0.27) | 2.55(2.93) | 0.37(0.22,0.59) | 2.79(2.81) |
| Argentina | 6.14(3.94,9.16) | 0.15(6.90) | 9.08(5.76,13.32) | 0.13(5.60) | 325.18(199.03,504.66) | 7.81(8.40) | 616.5(377.43,944.39) | 8.58(7.53) |
| Armenia | 0(0,0.01) | 0.00(4.18) | 0.17(0.12,0.25) | 0.03(4.07) | 5.91(3.06,10.29) | 1.76(7.46) | 17.47(9.79,28.83) | 2.90(6.41) |
| Australia | 11.86(8.57,15.78) | 0.46(6.98) | 8.63(5.34,13.04) | 0.15(3.73) | 617.11(422.2,858.64) | 23.97(8.51) | 698.89(418.21,1101.66) | 11.93(4.89) |
| Austria | 3.21(2.12,4.59) | 0.21(4.66) | 2.42(1.59,3.48) | 0.11(5.30) | 182.76(116.66,270.46) | 11.74(6.21) | 267.89(165.35,408.78) | 11.75(8.36) |
| Azerbaijan | 0(0,0.01) | 0.00(4.98) | 0.05(0.01,0.08) | 0.00(6.53) | 7.22(3.74,12.18) | 1.26(6.40) | 26.1(12.4,45.81) | 2.08(8.18) |
| Bahamas | 0.01(0.01,0.02) | 0.07(2.35) | 0.03(0.02,0.04) | 0.05(2.03) | 0.45(0.27,0.68) | 2.54(2.82) | 1.22(0.71,1.92) | 2.48(2.63) |
| Bahrain | 0(0,0) | 0.01(4.88) | 0.05(0.02,0.09) | 0.05(4.72) | 0.49(0.26,0.83) | 2.76(6.05) | 4.76(2.63,8.01) | 4.80(4.96) |
| Bangladesh | 20.75(8.03,39.25) | 0.39(6.60) | 35.8(13.66,69.36) | 0.21(5.14) | 486.05(231.26,848.79) | 9.12(6.82) | 1015.1(529.02,1722.32) | 6.00(5.17) |
| Barbados | 0.04(0.03,0.06) | 0.10(1.91) | 0.05(0.03,0.08) | 0.07(1.23) | 1.04(0.65,1.56) | 2.72(1.87) | 1.54(0.93,2.41) | 2.21(1.30) |
| Belarus | 0.08(0.06,0.12) | 0.00(5.46) | 0.17(0.11,0.24) | 0.01(6.78) | 30.9(15.94,51.8) | 1.81(6.36) | 59.37(32.64,96.26) | 2.70(6.00) |
| Belgium | 6.32(4.42,8.53) | 0.31(8.80) | 3.92(2.63,5.52) | 0.13(5.75) | 367.05(248.85,518.03) | 18.02(11.09) | 344.98(217.88,516.76) | 11.58(7.87) |
| Belize | 0.01(0,0.01) | 0.05(2.31) | 0.02(0.01,0.02) | 0.04(1.73) | 0.25(0.15,0.4) | 2.22(3.09) | 0.82(0.48,1.31) | 2.38(2.62) |
| Benin | 0(0,0) | 0.00(0.99) | 0(0,0) | 0.00(0.98) | 1.27(0.61,2.31) | 0.54(2.78) | 2.48(1.16,4.57) | 0.44(1.85) |
| Bermuda | 0.01(0,0.01) | 0.07(2.36) | 0.01(0.01,0.02) | 0.06(2.17) | 0.25(0.14,0.42) | 3.22(3.55) | 0.72(0.4,1.16) | 3.95(3.55) |
| Bhutan | 0.04(0.02,0.08) | 0.16(2.84) | 0.1(0.04,0.2) | 0.14(2.37) | 1.31(0.65,2.36) | 4.98(3.06) | 3.58(1.89,6.24) | 4.95(2.61) |
| Bolivia (Plurinational State of) | 0.63(0.32,1.05) | 0.17(2.79) | 1.12(0.57,1.94) | 0.10(1.88) | 20.9(12.72,32.06) | 5.62(3.34) | 56.53(34.53,85.29) | 5.08(2.48) |
| Bosnia and Herzegovina | 0.78(0.43,1.29) | 0.16(8.08) | 0.96(0.49,1.69) | 0.11(7.88) | 48.56(29.04,76.49) | 9.99(9.74) | 100.59(60.37,159.46) | 11.88(9.88) |
| Botswana | 0.14(0.07,0.25) | 0.22(7.36) | 0.15(0.06,0.3) | 0.09(4.35) | 5.89(3.43,9.34) | 9.01(8.46) | 10.12(5.95,16.36) | 6.10(5.99) |
| Brazil | 15.03(10.54,20.31) | 0.14(9.85) | 27.6(19.91,36.77) | 0.09(6.01) | 818.38(554.87,1165.51) | 7.79(11.45) | 1534.86(1058.05,2160.86) | 4.86(7.31) |
| Brunei Darussalam | 0.05(0.03,0.09) | 0.49(6.27) | 0.07(0.03,0.13) | 0.17(3.85) | 1.88(1.08,3.12) | 17.57(7.70) | 3.8(2.08,6.43) | 9.48(4.89) |
| Bulgaria | 0.8(0.52,1.16) | 0.05(6.46) | 0.43(0.27,0.62) | 0.02(4.65) | 69.71(41.15,110.56) | 4.14(9.12) | 65.12(38.46,101.92) | 3.38(7.06) |
| Burkina Faso | 0(0,0) | 0.00(0.53) | 0(0,0.01) | 0.00(0.79) | 1.5(0.72,2.62) | 0.29(1.50) | 3.19(1.51,5.55) | 0.31(1.34) |
| Burundi | 0.02(0,0.06) | 0.01(2.69) | 0.01(0,0.03) | 0.00(1.09) | 3.41(1.78,6.01) | 1.28(3.64) | 4.14(1.93,7.48) | 0.77(2.14) |
| Cabo Verde | 0(0,0) | 0.00(0.65) | 0(0,0) | 0.00(0.43) | 0.13(0.06,0.24) | 0.44(2.31) | 0.22(0.11,0.38) | 0.41(1.49) |
| Cambodia | 1.04(0.37,1.86) | 0.20(10.73) | 2.85(0.97,5.24) | 0.19(10.31) | 29.2(14.69,47.48) | 5.76(10.84) | 93.05(49.85,151.83) | 6.35(10.00) |
| Cameroon | 0(0,0.01) | 0.00(0.87) | 0(0,0.01) | 0.00(1.22) | 2.67(1.26,4.89) | 0.53(2.34) | 6.73(2.82,12.45) | 0.49(1.82) |
| Canada | 20.09(14.27,26.81) | 0.47(10.53) | 17.51(11.35,25.92) | 0.18(6.21) | 1017.47(712.48,1399.63) | 24.03(12.29) | 1528.92(924.33,2363.7) | 15.91(8.02) |
| Central African Republic | 0.01(0,0.04) | 0.01(1.86) | 0.01(0,0.03) | 0.00(1.13) | 2.06(1.11,3.52) | 1.67(3.65) | 2.65(1.41,4.54) | 1.19(2.72) |
| Chad | 0(0,0.01) | 0.00(1.54) | 0(0,0.01) | 0.00(1.98) | 1.57(0.75,2.89) | 0.47(2.73) | 2.73(1.29,5.06) | 0.43(2.34) |
| Chile | 2.77(1.73,4.32) | 0.23(4.11) | 3.14(1.87,4.89) | 0.09(2.45) | 125.41(75.92,198.15) | 10.24(5.42) | 289.86(164.29,469.19) | 8.73(3.90) |
| China | 339.8(202.39,474.92) | 0.34(10.01) | 762.98(374.81,1109.3) | 0.28(8.57) | 15944.9(11200.12,22099.04) | 15.92(10.75) | 36356.87(24352.92,51359.48) | 13.51(9.01) |
| Colombia | 3.54(2.32,5.3) | 0.17(3.75) | 5.05(3.08,7.84) | 0.07(2.16) | 132.37(81.22,206.01) | 6.53(4.71) | 291.45(173.88,464.55) | 4.18(2.94) |
| Comoros | 0(0,0) | 0.01(3.37) | 0(0,0.01) | 0.00(1.81) | 0.35(0.17,0.63) | 1.56(4.23) | 0.66(0.33,1.14) | 1.19(2.74) |
| Congo | 0.01(0,0.02) | 0.01(1.40) | 0.01(0,0.03) | 0.00(1.30) | 2.02(1.11,3.35) | 1.65(2.98) | 6.47(3.41,11.18) | 2.30(3.60) |
| Cook Islands | 0(0,0) | 0.00(5.11) | 0(0,0) | 0.00(5.50) | 0.02(0.01,0.03) | 1.12(7.81) | 0.04(0.02,0.08) | 1.27(6.59) |
| Costa Rica | 0.5(0.33,0.72) | 0.24(4.74) | 0.97(0.61,1.45) | 0.14(2.72) | 18.32(11.76,26.74) | 8.78(5.15) | 52.75(32.17,81.39) | 7.53(3.34) |
| Côte d'Ivoire | 0(0,0.01) | 0.14(8.98) | 0.01(0,0.02) | 0.07(6.71) | 2.82(1.4,5.01) | 7.46(10.61) | 7.61(3.72,13.31) | 6.79(9.00) |
| Croatia | 1.95(1.24,2.94) | 0.00(1.36) | 1.7(1.05,2.59) | 0.00(2.01) | 101.09(62.65,159.91) | 0.68(3.36) | 155.16(91.54,240.81) | 0.64(2.57) |
| Cuba | 2.03(1.27,3.13) | 0.25(9.31) | 3.06(1.79,4.92) | 0.14(6.43) | 92.54(54.67,145.85) | 12.80(10.33) | 161.63(90.58,262.44) | 13.00(8.54) |
| Cyprus | 0.62(0.33,1.11) | 0.16(5.93) | 0.66(0.36,1.11) | 0.13(3.77) | 26.2(15.67,42.73) | 7.22(7.85) | 54.15(33.03,82.22) | 6.65(5.57) |
| Czechia | 2.52(1.67,3.68) | 0.58(5.43) | 1.98(1.29,2.89) | 0.24(5.30) | 137.12(85.59,210.49) | 24.43(7.27) | 190.97(117.09,285.9) | 19.86(7.25) |
| Democratic People's Republic of Korea | 4.05(2.08,7.08) | 0.22(5.73) | 8.26(4.16,14.25) | 0.21(4.60) | 167.98(102.7,258.33) | 9.26(6.47) | 375.69(226.61,571.06) | 9.44(5.49) |
| Democratic Republic of the Congo | 0.06(0.02,0.24) | 0.00(1.04) | 0.06(0.02,0.28) | 0.00(0.63) | 15.58(8.11,27.58) | 0.89(2.21) | 31.08(15.86,53.16) | 0.79(1.76) |
| Denmark | 7.49(5.47,9.77) | 0.71(14.97) | 6.02(4.11,8.43) | 0.39(8.61) | 410.37(283.75,562.9) | 39.05(16.56) | 456.26(295.02,665.83) | 29.89(10.79) |
| Djibouti | 0(0,0) | 0.01(5.15) | 0(0,0.01) | 0.00(3.63) | 0.31(0.15,0.52) | 2.29(6.55) | 1.54(0.78,2.57) | 2.30(5.42) |
| Dominica | 0.01(0,0.01) | 0.08(2.33) | 0.01(0,0.01) | 0.06(1.88) | 0.19(0.12,0.31) | 2.52(2.77) | 0.24(0.14,0.39) | 2.25(2.51) |
| Dominican Republic | 1.24(0.57,2.16) | 0.29(8.00) | 2.54(1.18,4.48) | 0.21(6.24) | 27.85(15.36,45.57) | 6.42(8.75) | 65.95(36.75,109.51) | 5.44(6.91) |
| Ecuador | 1.7(1.16,2.38) | 0.28(4.04) | 1.59(1,2.39) | 0.08(1.62) | 49.84(33.18,72.6) | 8.09(4.44) | 87.77(54.34,133.06) | 4.34(2.10) |
| Egypt | 0.6(0.24,1.12) | 0.02(3.84) | 1.15(0.51,2.19) | 0.02(4.66) | 42.64(25.13,68.5) | 1.42(5.03) | 139.57(78.25,227.6) | 1.90(5.28) |
| El Salvador | 0.11(0.06,0.21) | 0.03(1.85) | 0.23(0.12,0.41) | 0.03(1.79) | 4.96(2.91,8.07) | 1.40(2.37) | 13.82(7.92,22.76) | 1.80(2.40) |
| Equatorial Guinea | 0(0,0) | 0.01(1.60) | 0(0,0) | 0.00(0.98) | 0.29(0.14,0.52) | 1.33(3.12) | 0.93(0.48,1.57) | 1.77(2.55) |
| Eritrea | 0(0,0.01) | 0.00(1.42) | 0(0,0.01) | 0.00(0.69) | 0.82(0.4,1.49) | 0.77(2.15) | 1.69(0.84,2.99) | 0.60(1.44) |
| Estonia | 0.58(0.39,0.85) | 0.22(5.33) | 0.95(0.64,1.34) | 0.27(6.53) | 30.81(19.86,45.78) | 11.53(6.24) | 55(36.63,79.44) | 15.54(7.82) |
| Eswatini | 0.05(0.02,0.11) | 0.17(3.86) | 0.06(0.01,0.13) | 0.09(2.53) | 1.86(0.97,3.19) | 5.99(4.18) | 2.36(1.14,4.38) | 3.76(2.76) |
| Ethiopia | 0.08(0.02,0.21) | 0.00(1.45) | 0.04(0.01,0.12) | 0.00(0.72) | 16.32(8.92,27.43) | 0.74(1.88) | 20.98(11.8,34.38) | 0.44(1.29) |
| Fiji | 0(0,0) | 0.00(3.80) | 0(0,0) | 0.00(2.64) | 0.39(0.2,0.68) | 1.05(8.64) | 0.92(0.46,1.6) | 1.00(7.08) |
| Finland | 5.17(3.3,7.7) | 0.55(4.12) | 3.88(2.58,5.58) | 0.24(3.70) | 218.77(140.18,324.12) | 23.40(5.69) | 300.89(188.39,446.31) | 18.28(5.46) |
| France | 27.55(19.46,37.68) | 0.25(6.03) | 17.17(11.29,24.88) | 0.10(4.15) | 1283.06(869.81,1825.59) | 11.83(7.62) | 1554.39(970.04,2349.28) | 8.73(6.24) |
| Gabon | 0(0,0.01) | 0.00(0.99) | 0(0,0.01) | 0.00(0.98) | 0.86(0.46,1.44) | 1.24(2.18) | 2.42(1.29,3.98) | 2.02(2.73) |
| Gambia | 0(0,0) | 0.00(1.35) | 0(0,0) | 0.00(1.74) | 0.34(0.17,0.6) | 0.87(4.31) | 0.63(0.29,1.13) | 0.58(2.36) |
| Georgia | 0.01(0,0.01) | 0.00(4.18) | 0.12(0.08,0.17) | 0.01(4.50) | 12.68(5.93,22.22) | 1.56(6.35) | 18.55(10.16,31.76) | 2.28(6.89) |
| Germany | 42.82(29.09,59.07) | 0.26(6.60) | 28.09(18.64,39.66) | 0.11(5.14) | 2090.72(1401.76,2994.39) | 12.84(8.93) | 2269.92(1429.37,3392.85) | 9.23(7.36) |
| Ghana | 0(0,0.01) | 0.00(1.45) | 0.25(0.07,0.57) | 0.01(4.61) | 2.74(1.33,5.01) | 0.40(2.17) | 14.16(6.41,26.72) | 0.75(2.64) |
| Greece | 1.99(1.41,2.67) | 0.10(8.65) | 2.96(1.98,4.21) | 0.10(6.70) | 212.75(137.12,314.39) | 10.63(12.28) | 276.2(166.23,421.78) | 9.27(9.53) |
| Greenland | 0.01(0.01,0.03) | 0.41(14.98) | 0.02(0.01,0.05) | 0.26(13.05) | 0.69(0.42,1.07) | 19.24(17.07) | 1.82(1.15,2.74) | 20.50(14.82) |
| Grenada | 0(0,0) | 0.02(1.21) | 0(0,0) | 0.01(1.01) | 0.1(0.05,0.16) | 1.01(2.10) | 0.15(0.08,0.25) | 1.06(1.91) |
| Guam | 0(0,0) | 0.00(3.15) | 0(0,0) | 0.00(3.93) | 0.1(0.05,0.17) | 1.13(6.32) | 0.37(0.19,0.61) | 1.33(5.90) |
| Guatemala | 0.43(0.28,0.63) | 0.11(3.70) | 1.11(0.69,1.68) | 0.08(2.29) | 13.41(8.72,20.09) | 3.38(3.71) | 43.64(26.99,66.34) | 3.27(2.47) |
| Guinea | 0(0,0.01) | 0.00(1.40) | 0(0,0.01) | 0.00(2.13) | 2.23(1.12,4.02) | 0.55(2.96) | 3.72(1.78,6.75) | 0.58(2.75) |
| Guinea-Bissau | 0(0,0) | 0.00(0.53) | 0(0,0) | 0.00(0.88) | 0.12(0.06,0.21) | 0.27(1.40) | 0.27(0.13,0.5) | 0.36(1.60) |
| Guyana | 0(0,0) | 0.00(1.74) | 0.01(0.01,0.02) | 0.02(1.69) | 0.33(0.17,0.56) | 0.74(3.40) | 0.86(0.5,1.39) | 1.10(2.54) |
| Haiti | 0.19(0.08,0.38) | 0.05(1.57) | 0.27(0.11,0.56) | 0.03(1.22) | 5.95(3.19,10.55) | 1.59(1.86) | 9.51(5.03,17.03) | 1.18(1.43) |
| Honduras | 0.69(0.29,1.4) | 0.29(4.04) | 2.36(1.11,4.04) | 0.31(3.39) | 21.65(11.87,37.52) | 9.20(4.33) | 83.89(48.52,131.03) | 11.15(3.67) |
| Hungary | 5.2(3.52,7.38) | 0.26(6.77) | 2.67(1.7,3.96) | 0.10(4.73) | 249.36(163.51,359.95) | 12.68(8.64) | 225.37(140.77,338.46) | 8.62(6.70) |
| Iceland | 0.12(0.08,0.19) | 0.33(8.98) | 0.08(0.05,0.12) | 0.11(5.33) | 6.38(3.88,9.93) | 17.28(10.90) | 7.84(4.6,12.3) | 10.33(7.15) |
| India | 140.6(66.15,217.15) | 0.27(5.34) | 300.26(157.72,479.31) | 0.21(4.14) | 4819.53(2996.12,6980.34) | 9.34(5.51) | 11568.09(7282.87,17211.03) | 7.97(3.98) |
| Indonesia | 7.94(2.66,12.93) | 0.07(12.45) | 27.88(8.7,45.25) | 0.10(13.29) | 280.12(159.49,419.57) | 2.57(10.64) | 1008.57(566.47,1508.85) | 3.58(11.74) |
| Iran (Islamic Republic of) | 0.18(0.08,0.34) | 0.01(2.40) | 0.78(0.42,1.31) | 0.01(2.72) | 31.14(17.75,50.56) | 1.01(4.29) | 114.08(65.89,183.56) | 1.24(4.01) |
| Iraq | 0.1(0.04,0.21) | 0.01(4.86) | 0.15(0.07,0.29) | 0.01(3.85) | 17.96(9.73,29.17) | 1.99(8.34) | 53.86(30.17,84.99) | 2.04(6.37) |
| Ireland | 7.51(5.57,9.79) | 1.40(13.21) | 3.44(2.19,5.06) | 0.34(6.45) | 334.68(236.13,454.05) | 62.26(14.47) | 314.27(196.77,471.73) | 30.93(8.11) |
| Israel | 0.79(0.52,1.17) | 0.12(7.32) | 0.97(0.62,1.44) | 0.06(4.60) | 51.7(31.53,80.79) | 8.12(8.88) | 98.7(59.5,155.19) | 6.22(5.98) |
| Italy | 26.03(19.29,33.63) | 0.22(7.46) | 23.16(15.9,31.89) | 0.13(4.05) | 1850.08(1253.87,2617.31) | 15.66(9.20) | 1695.73(1082.52,2516.72) | 9.41(5.87) |
| Jamaica | 0.15(0.09,0.23) | 0.07(3.22) | 0.22(0.13,0.36) | 0.06(2.22) | 6.16(3.69,9.68) | 2.68(4.24) | 9.96(5.75,15.86) | 2.54(3.14) |
| Japan | 99.91(74.71,127.82) | 0.46(7.29) | 95.18(65.66,130.61) | 0.21(4.41) | 5283.68(3669.16,7259.98) | 24.22(8.61) | 4701.05(3074.41,6850.84) | 10.61(5.21) |
| Jordan | 0.02(0.01,0.03) | 0.01(4.57) | 0.09(0.04,0.16) | 0.01(4.54) | 2.9(1.55,4.86) | 2.07(8.57) | 20.83(11.29,33.45) | 2.56(7.60) |
| Kazakhstan | 0.02(0.02,0.04) | 0.00(4.05) | 0.86(0.56,1.25) | 0.04(3.99) | 18.8(10.19,32.02) | 1.22(5.22) | 52.05(30.86,82.96) | 2.35(4.84) |
| Kenya | 0.06(0.02,0.21) | 0.01(3.39) | 0.06(0.02,0.24) | 0.00(1.78) | 16.61(9.69,26.32) | 1.78(4.71) | 30.33(17.97,48.51) | 1.17(2.89) |
| Kiribati | 0(0,0) | 0.00(8.72) | 0(0,0) | 0.00(9.26) | 0.06(0.03,0.11) | 1.57(13.45) | 0.17(0.09,0.27) | 2.03(14.82) |
| Kuwait | 0.01(0,0.01) | 0.01(4.08) | 0.11(0.06,0.17) | 0.04(4.42) | 1.22(0.66,2.1) | 2.10(5.63) | 7.96(4.39,13.3) | 2.79(4.46) |
| Kyrgyzstan | 0.28(0.18,0.41) | 0.07(5.70) | 0.96(0.6,1.42) | 0.17(7.19) | 17.37(10.77,26.45) | 4.69(4.53) | 58.24(37,88.64) | 10.28(6.30) |
| Lao People's Democratic Republic | 0.4(0.14,0.78) | 0.17(9.06) | 0.69(0.23,1.29) | 0.13(8.29) | 11.73(5.7,19.82) | 4.91(9.23) | 23.79(13,39.05) | 4.55(8.28) |
| Latvia | 0.65(0.41,1.01) | 0.14(4.64) | 0.77(0.52,1.09) | 0.15(4.46) | 34.33(21.01,53.31) | 7.34(5.39) | 41.79(28.05,60.48) | 7.95(5.39) |
| Lebanon | 0.15(0.07,0.27) | 0.06(10.01) | 0.26(0.12,0.49) | 0.04(11.00) | 10.79(6.17,17.47) | 4.08(10.99) | 37.39(20.98,59.84) | 5.09(12.39) |
| Lesotho | 0.16(0.07,0.31) | 0.16(5.14) | 0.22(0.08,0.45) | 0.17(4.88) | 5.94(3.37,9.99) | 5.92(5.55) | 8.91(4.7,15.1) | 6.96(5.53) |
| Liberia | 0(0,0) | 0.00(0.71) | 0(0,0) | 0.00(0.87) | 0.6(0.29,1.08) | 0.43(2.25) | 0.94(0.45,1.71) | 0.43(1.83) |
| Libya | 0.01(0,0.02) | 0.00(1.52) | 0.08(0.03,0.19) | 0.01(2.04) | 2.1(1.07,3.7) | 0.97(4.10) | 7.43(4.12,12.51) | 1.35(3.45) |
| Lithuania | 0.66(0.45,0.96) | 0.11(3.12) | 1.37(0.91,1.97) | 0.18(3.96) | 34.9(22.39,51.17) | 5.98(3.75) | 64.56(42.39,94.37) | 8.51(4.54) |
| Luxembourg | 0.15(0.09,0.23) | 0.21(6.66) | 0.12(0.08,0.18) | 0.09(5.57) | 8.91(5.4,13.52) | 12.50(8.97) | 13.32(7.81,20.53) | 10.01(8.09) |
| Madagascar | 0.03(0.01,0.09) | 0.00(2.76) | 0.01(0,0.04) | 0.00(0.99) | 5.49(2.69,9.87) | 0.94(3.21) | 5.92(2.91,10.69) | 0.50(1.49) |
| Malawi | 0.04(0.01,0.14) | 0.01(4.14) | 0.04(0.01,0.17) | 0.01(3.06) | 8.51(4.57,14.71) | 1.92(5.25) | 17.86(9.75,29.8) | 2.17(4.94) |
| Malaysia | 0.17(0.07,0.34) | 0.02(2.92) | 0.31(0.14,0.6) | 0.01(2.47) | 13.43(7.5,21.94) | 1.28(5.38) | 37.52(19.5,63.61) | 1.07(4.24) |
| Maldives | 0.01(0.01,0.02) | 0.14(8.81) | 0.02(0.01,0.04) | 0.06(5.53) | 0.51(0.3,0.82) | 5.23(8.88) | 1.29(0.72,2.1) | 3.61(5.86) |
| Mali | 0(0,0.01) | 0.00(0.94) | 0.01(0,0.03) | 0.00(2.97) | 1.54(0.74,2.8) | 0.34(1.93) | 6.07(3.01,10.68) | 0.61(3.01) |
| Malta | 0.11(0.07,0.17) | 0.20(6.60) | 0.09(0.06,0.14) | 0.07(4.04) | 6.07(3.73,9.27) | 11.08(8.52) | 9.19(5.35,14.24) | 7.04(5.89) |
| Marshall Islands | 0(0,0) | 0.00(2.35) | 0(0,0) | 0.00(2.96) | 0.01(0.01,0.02) | 0.72(4.44) | 0.04(0.02,0.07) | 1.03(4.87) |
| Mauritania | 0(0,0) | 0.00(1.43) | 0(0,0) | 0.00(1.43) | 0.46(0.22,0.81) | 0.40(1.76) | 1.03(0.46,1.96) | 0.40(1.34) |
| Mauritius | 0.01(0,0.01) | 0.01(6.60) | 0.18(0.12,0.26) | 0.07(3.82) | 1.56(0.87,2.56) | 1.76(6.34) | 8.34(5.04,12.55) | 3.41(4.73) |
| Mexico | 34.36(24.36,46.53) | 0.70(5.67) | 28.02(18.61,40.04) | 0.18(2.36) | 1049.75(733.62,1466.5) | 21.32(6.05) | 1206.34(803.93,1763.94) | 7.79(2.59) |
| Micronesia (Federated States of) | 0(0,0) | 0.00(4.76) | 0(0,0) | 0.00(5.63) | 0.09(0.05,0.15) | 1.49(8.84) | 0.21(0.11,0.36) | 2.37(9.49) |
| Monaco | 0(0,0) | 0.02(7.51) | 0(0,0) | 0.01(6.09) | 0.5(0.27,0.85) | 5.41(10.22) | 0.59(0.31,0.99) | 4.72(8.53) |
| Mongolia | 0.07(0.03,0.19) | 0.06(5.87) | 0.2(0.09,0.36) | 0.08(4.46) | 3.86(2.19,6.82) | 3.19(6.98) | 12.86(7.58,19.99) | 5.17(6.75) |
| Montenegro | 0.1(0.05,0.15) | 0.12(12.61) | 0.16(0.09,0.25) | 0.12(11.42) | 7.58(4.67,11.21) | 9.81(15.10) | 14.54(8.97,21.61) | 10.92(14.21) |
| Morocco | 0.07(0.03,0.16) | 0.00(0.99) | 0.36(0.15,0.75) | 0.01(1.12) | 10.83(5.95,18.07) | 0.64(2.68) | 32.99(18.59,54.15) | 0.78(2.06) |
| Mozambique | 0.04(0.01,0.18) | 0.01(2.86) | 0.04(0.01,0.2) | 0.00(1.99) | 9.13(4.73,15.56) | 1.36(4.01) | 16.37(8.99,27.72) | 1.35(3.36) |
| Myanmar | 5.69(2.34,9.75) | 0.21(12.75) | 5.4(2.09,10.05) | 0.09(6.02) | 173.06(95.21,276.1) | 6.37(13.10) | 200.87(109.59,330.85) | 3.42(6.19) |
| Namibia | 0.17(0.08,0.32) | 0.22(7.49) | 0.2(0.06,0.42) | 0.13(4.99) | 6.55(3.67,10.71) | 8.41(7.99) | 9.25(4.72,15.81) | 5.93(5.52) |
| Nauru | 0(0,0) | 0.00(5.24) | 0(0,0) | 0.00(4.46) | 0.01(0,0.01) | 1.64(8.57) | 0.01(0.01,0.02) | 1.80(6.95) |
| Nepal | 6.29(2.92,11) | 0.60(12.03) | 11.29(5.17,21.31) | 0.40(7.77) | 185.6(106.72,291.45) | 17.68(12.71) | 386.85(219.05,632.55) | 13.58(8.08) |
| Netherlands | 23.27(16.51,30.99) | 0.90(9.16) | 14.94(9.86,21.03) | 0.33(6.70) | 1077.9(749.11,1487.12) | 41.75(11.38) | 1080.8(688.78,1608.94) | 23.53(8.65) |
| New Zealand | 3.36(2.41,4.47) | 0.65(8.67) | 2.49(1.65,3.54) | 0.23(5.56) | 190.2(126.96,268.5) | 36.86(10.25) | 204.46(127.79,302.76) | 18.50(6.80) |
| Nicaragua | 0.22(0.11,0.37) | 0.13(3.19) | 0.51(0.26,0.89) | 0.09(2.60) | 7.76(4.63,12.01) | 4.44(3.43) | 24.62(14.75,38.89) | 4.25(2.76) |
| Niger | 0(0,0) | 0.00(0.60) | 0(0,0.01) | 0.00(1.16) | 0.59(0.27,1.07) | 0.19(1.14) | 1.81(0.83,3.2) | 0.19(1.01) |
| Nigeria | 0.02(0.01,0.03) | 0.00(0.60) | 0.02(0.01,0.05) | 0.00(0.89) | 14.78(8.22,24.22) | 0.29(1.56) | 27.74(15.76,45.16) | 0.28(1.32) |
| Niue | 0(0,0) | 0.00(2.55) | 0(0,0) | 0.00(3.08) | 0(0,0) | 0.99(5.84) | 0(0,0.01) | 1.46(6.31) |
| North Macedonia | 0.1(0.05,0.18) | 0.04(8.93) | 0.09(0.04,0.16) | 0.02(7.21) | 10.67(5.89,17.5) | 4.63(11.93) | 21.69(11.93,35.27) | 4.89(10.30) |
| Northern Mariana Islands | 0(0,0) | 0.00(10.60) | 0(0,0) | 0.00(10.37) | 0.03(0.01,0.05) | 1.80(8.10) | 0.12(0.06,0.2) | 1.88(7.33) |
| Norway | 8.21(5.46,11.73) | 0.91(8.07) | 1.98(1.19,3.13) | 0.15(3.81) | 388.96(251.14,570.42) | 43.35(9.80) | 175.94(99.91,286.61) | 13.72(5.13) |
| Oman | 0(0,0.01) | 0.01(2.35) | 0.01(0,0.02) | 0.01(2.23) | 0.51(0.28,0.86) | 0.72(3.55) | 1.78(0.93,3.03) | 0.90(2.86) |
| Pakistan | 25.17(11.93,43.63) | 0.38(5.64) | 30.53(15.65,52.64) | 0.23(3.28) | 897.59(558.06,1394.55) | 13.65(7.76) | 1084.33(650.98,1669.33) | 8.07(4.58) |
| Palau | 0(0,0) | 0.00(3.09) | 0(0,0) | 0.00(2.96) | 0.02(0.01,0.03) | 1.27(6.03) | 0.04(0.02,0.07) | 1.62(5.91) |
| Palestine | 0.03(0.01,0.06) | 0.03(5.24) | 0.05(0.02,0.1) | 0.02(5.57) | 1.93(1.09,3.12) | 1.86(5.88) | 5.98(3.23,9.92) | 2.13(5.85) |
| Panama | 0.2(0.13,0.3) | 0.11(3.98) | 0.37(0.21,0.6) | 0.07(2.24) | 7.19(4.43,11.18) | 4.06(4.15) | 16.5(9.39,26.89) | 3.01(2.65) |
| Papua New Guinea | 0(0,0) | 0.00(5.25) | 0(0,0) | 0.00(4.68) | 1.73(0.85,3.15) | 0.87(6.94) | 4.72(2.16,8.69) | 0.90(6.52) |
| Paraguay | 0.53(0.29,0.87) | 0.20(10.36) | 1.81(0.9,3.13) | 0.25(7.21) | 23.91(14.77,36.4) | 9.06(10.48) | 82.72(49.72,129.35) | 11.62(7.37) |
| Peru | 1.59(0.79,2.66) | 0.11(2.28) | 2.83(1.43,5.1) | 0.07(2.18) | 51.3(30.17,80.25) | 3.70(2.58) | 197.9(117.08,303.57) | 4.86(2.65) |
| Philippines | 7.27(3.23,10.91) | 0.22(10.50) | 11.93(6.06,17.6) | 0.12(6.83) | 263.71(164.07,383.08) | 8.03(11.46) | 454.72(295.84,654.79) | 4.71(7.52) |
| Poland | 32.91(24.64,42.82) | 0.57(8.70) | 14.58(10.41,19.98) | 0.15(5.99) | 1361.61(986.93,1827.82) | 23.78(9.98) | 1066.21(710.34,1538.16) | 10.87(7.94) |
| Portugal | 3.08(2.2,4.2) | 0.16(4.75) | 1.77(1.17,2.55) | 0.06(2.04) | 150.15(101.25,214.54) | 8.04(5.96) | 145.68(89.77,219.63) | 4.66(3.21) |
| Puerto Rico | 0.66(0.42,0.97) | 0.14(3.70) | 0.68(0.41,1.05) | 0.07(2.37) | 20.1(12.2,30.72) | 4.33(4.43) | 39.37(23.16,63.44) | 4.18(3.49) |
| Qatar | 0(0,0) | 0.01(2.01) | 0(0,0.01) | 0.00(2.72) | 0.1(0.05,0.18) | 1.22(4.49) | 1.4(0.64,2.4) | 1.77(4.15) |
| Republic of Korea | 12.45(6.5,20.73) | 0.37(8.74) | 10.78(5.49,19.34) | 0.09(5.82) | 438.15(271.75,666.32) | 13.12(8.82) | 922.83(559.53,1419.9) | 7.35(5.70) |
| Republic of Moldova | 0.17(0.11,0.24) | 0.03(4.06) | 0.53(0.35,0.74) | 0.07(5.85) | 11.17(7.03,17.4) | 1.99(4.58) | 27.62(17.76,41.77) | 3.42(5.37) |
| Romania | 0.03(0.02,0.05) | 0.00(4.64) | 0.03(0.02,0.04) | 0.00(3.47) | 82.92(43.16,144.35) | 2.29(7.95) | 125.04(67.49,205.37) | 2.50(5.62) |
| Russian Federation | 16.85(12.44,22.34) | 0.07(3.34) | 38.67(28.59,50.81) | 0.12(4.40) | 911.09(645.19,1277.8) | 3.86(3.94) | 1904.11(1349.97,2605.15) | 5.76(5.31) |
| Rwanda | 0.07(0.02,0.4) | 0.02(7.37) | 0.07(0.02,0.56) | 0.01(7.21) | 10.6(5.58,19.46) | 3.27(8.49) | 31.61(16.43,54.35) | 4.34(8.96) |
| Saint Kitts and Nevis | 0(0,0.01) | 0.08(1.87) | 0(0,0.01) | 0.05(1.28) | 0.13(0.08,0.21) | 2.52(2.16) | 0.19(0.11,0.32) | 2.17(1.69) |
| Saint Lucia | 0.01(0.01,0.02) | 0.11(2.77) | 0.02(0.01,0.03) | 0.07(1.71) | 0.37(0.23,0.55) | 3.27(3.38) | 0.79(0.48,1.21) | 2.63(2.32) |
| Saint Vincent and the Grenadines | 0(0,0) | 0.04(1.93) | 0(0,0) | 0.01(1.15) | 0.12(0.07,0.18) | 1.29(2.41) | 0.21(0.12,0.35) | 1.15(2.34) |
| Samoa | 0(0,0) | 0.00(4.41) | 0(0,0) | 0.00(4.00) | 0.18(0.09,0.3) | 1.72(9.53) | 0.32(0.17,0.53) | 1.86(8.06) |
| San Marino | 0(0,0.01) | 0.08(6.26) | 0(0,0.01) | 0.03(4.48) | 0.3(0.17,0.48) | 6.58(9.37) | 0.39(0.22,0.64) | 4.28(7.24) |
| Sao Tome and Principe | 0(0,0) | 0.00(0.54) | 0(0,0) | 0.00(0.87) | 0.03(0.02,0.05) | 0.38(1.75) | 0.05(0.02,0.1) | 0.44(1.53) |
| Saudi Arabia | 0.03(0.01,0.06) | 0.00(1.35) | 0.14(0.06,0.28) | 0.01(2.44) | 3.7(2.02,6.21) | 0.60(2.77) | 21.29(10.93,36.99) | 1.19(3.62) |
| Senegal | 0(0,0) | 0.00(0.67) | 0(0,0.01) | 0.00(0.98) | 2.16(1,3.76) | 0.57(2.81) | 4.29(1.89,7.74) | 0.48(1.91) |
| Serbia | 1.74(0.94,3.03) | 0.12(6.54) | 1.87(1.02,3.16) | 0.08(5.95) | 103.73(61.03,164.71) | 6.97(8.65) | 165.76(100.74,262.23) | 7.38(8.03) |
| Seychelles | 0(0,0) | 0.02(8.50) | 0(0,0) | 0.01(8.03) | 0.12(0.07,0.19) | 1.60(7.90) | 0.24(0.12,0.41) | 1.70(7.06) |
| Sierra Leone | 0(0,0) | 0.00(1.02) | 0(0,0) | 0.00(1.29) | 1.38(0.69,2.38) | 0.57(3.01) | 2.09(1.05,3.68) | 0.50(2.32) |
| Singapore | 0.11(0.07,0.18) | 0.04(3.82) | 0.1(0.06,0.16) | 0.01(2.78) | 8.08(4.48,13.21) | 3.16(5.02) | 25.15(13.29,43.14) | 2.25(3.71) |
| Slovakia | 0.52(0.29,0.86) | 0.07(4.30) | 0.47(0.25,0.79) | 0.04(3.69) | 28.77(17.02,47.44) | 3.65(5.84) | 48.98(29.32,77.25) | 3.81(5.84) |
| Slovenia | 0.94(0.6,1.42) | 0.30(5.79) | 0.75(0.48,1.09) | 0.13(4.70) | 46.36(28.04,71.69) | 14.70(7.31) | 82.94(51.16,123.57) | 14.28(7.45) |
| Solomon Islands | 0(0,0) | 0.00(4.95) | 0(0,0) | 0.00(4.96) | 0.22(0.12,0.39) | 1.49(10.00) | 0.68(0.37,1.14) | 1.90(9.62) |
| Somalia | 0.02(0.01,0.09) | 0.01(3.22) | 0.03(0.01,0.11) | 0.00(1.98) | 3.65(1.81,6.49) | 1.63(4.13) | 7.66(3.82,13.64) | 1.19(3.06) |
| South Africa | 8.23(4.77,12.55) | 0.34(6.80) | 6.08(3.6,9.83) | 0.11(2.81) | 351.32(235.3,513.48) | 14.44(8.00) | 324.04(206.75,497.92) | 5.79(3.72) |
| South Sudan | 0.02(0.01,0.06) | 0.01(3.19) | 0.01(0,0.05) | 0.00(2.05) | 4.13(1.98,7.45) | 1.36(4.30) | 4.5(2.12,8.07) | 1.13(3.24) |
| Spain | 14.03(9.95,19.23) | 0.19(6.49) | 9.22(6.06,13.06) | 0.08(3.65) | 770.17(521.36,1100.74) | 10.55(7.67) | 889.28(554.63,1321.07) | 7.33(5.44) |
| Sri Lanka | 0.14(0.07,0.25) | 0.01(4.99) | 0.15(0.07,0.28) | 0.00(2.15) | 13.68(7.24,23.22) | 1.10(6.11) | 24.28(12.55,41.81) | 0.68(3.00) |
| Sudan | 0.09(0.04,0.21) | 0.01(1.94) | 0.26(0.11,0.54) | 0.01(2.12) | 8.05(4.32,13.66) | 0.75(3.45) | 19.95(10.9,34.11) | 0.94(3.09) |
| Suriname | 0.02(0.01,0.03) | 0.06(5.61) | 0.02(0.01,0.05) | 0.03(3.79) | 0.78(0.46,1.29) | 2.59(6.97) | 1.42(0.78,2.36) | 1.81(4.72) |
| Sweden | 11.07(7.64,15.31) | 0.57(8.10) | 7.23(4.7,10.24) | 0.27(6.52) | 562.9(374.42,815.31) | 28.74(9.28) | 537.61(339.46,810.4) | 19.87(8.67) |
| Switzerland | 4.54(2.99,6.53) | 0.34(6.64) | 3.18(2.03,4.76) | 0.14(5.50) | 239.2(155.69,349.78) | 18.06(8.06) | 306.75(183.46,472.26) | 13.54(7.24) |
| Syrian Arab Republic | 0.17(0.09,0.3) | 0.03(8.50) | 0.44(0.19,0.74) | 0.03(6.11) | 11.41(6.87,18.09) | 1.91(8.07) | 31.39(17.76,49.79) | 1.97(5.55) |
| Taiwan (Province of China) | 5.62(3.9,7.77) | 0.28(8.53) | 5.16(3.21,7.54) | 0.09(3.68) | 228.04(151.31,331.72) | 11.36(8.28) | 258.44(157.77,398.08) | 4.53(3.76) |
| Tajikistan | 0.03(0.01,0.09) | 0.01(5.58) | 0.08(0.02,0.19) | 0.01(2.72) | 8.24(4.23,14.65) | 2.54(6.41) | 14.41(7.68,24.36) | 2.18(3.87) |
| Thailand | 5.17(2.39,9.99) | 0.13(7.95) | 8.73(3.95,17.26) | 0.06(4.56) | 187.92(107.11,312.95) | 4.69(8.17) | 425.58(245.6,693.74) | 2.99(4.92) |
| Timor-Leste | 0.02(0.01,0.05) | 0.09(6.86) | 0.09(0.03,0.19) | 0.09(6.09) | 0.78(0.4,1.32) | 2.86(7.23) | 3.26(1.6,5.58) | 3.03(5.95) |
| Togo | 0(0,0) | 0.00(2.55) | 0(0,0.01) | 0.00(2.36) | 1.4(0.69,2.47) | 1.05(5.25) | 3.67(1.76,6.71) | 0.90(3.59) |
| Tokelau | 0(0,0) | 0.00(3.29) | 0(0,0) | 0.00(2.97) | 0(0,0) | 1.04(6.96) | 0(0,0) | 1.21(5.73) |
| Tonga | 0(0,0) | 0.00(4.38) | 0(0,0) | 0.00(3.50) | 0.11(0.06,0.19) | 1.69(10.04) | 0.16(0.08,0.28) | 1.69(8.21) |
| Trinidad and Tobago | 0.19(0.12,0.29) | 0.19(3.34) | 0.25(0.15,0.39) | 0.10(2.19) | 5.84(3.65,8.81) | 5.63(3.87) | 10.01(6.12,15.78) | 3.88(2.72) |
| Tunisia | 0.05(0.02,0.11) | 0.01(3.96) | 0.31(0.12,0.7) | 0.02(4.90) | 9.47(5.2,15.64) | 1.55(7.25) | 33.01(18.58,55.13) | 1.95(5.99) |
| Turkey | 7.62(3.54,14.19) | 0.19(6.97) | 9.43(4.52,16.59) | 0.08(4.87) | 320.1(188.29,512.71) | 7.88(8.41) | 695.98(419.51,1047.99) | 5.89(5.88) |
| Turkmenistan | 0(0,0) | 0.00(5.77) | 0.07(0.04,0.12) | 0.02(3.39) | 2.39(1.19,4.11) | 1.06(5.48) | 6.1(3.23,10.09) | 1.29(3.89) |
| Tuvalu | 0(0,0) | 0.00(4.27) | 0(0,0) | 0.00(4.07) | 0.01(0,0.02) | 1.17(7.69) | 0.02(0.01,0.03) | 1.50(7.28) |
| Uganda | 0.04(0.01,0.17) | 0.00(2.53) | 0.04(0.01,0.3) | 0.00(1.83) | 8.08(4.16,14) | 1.10(3.19) | 18.48(9.8,31.76) | 1.16(2.73) |
| Ukraine | 2.68(1.74,3.96) | 0.03(3.55) | 2.94(1.82,4.54) | 0.03(3.64) | 190.03(120.55,286.92) | 1.98(4.77) | 203.33(128.89,300.75) | 1.92(4.14) |
| United Arab Emirates | 0.02(0.01,0.03) | 0.05(5.13) | 0.07(0.03,0.13) | 0.02(4.28) | 0.74(0.42,1.21) | 2.17(4.84) | 6.97(3.8,12.06) | 2.05(5.13) |
| United Kingdom | 167.76(130.29,207.97) | 1.41(11.68) | 60.89(43.51,82.08) | 0.37(6.48) | 6580.52(4837.96,8661.96) | 55.21(13.80) | 3957.35(2650.45,5712.1) | 24.06(8.14) |
| United Republic of Tanzania | 0.13(0.03,0.52) | 0.01(4.72) | 0.1(0.03,0.61) | 0.00(2.69) | 29.32(15.65,50.04) | 2.32(6.27) | 54.77(29.94,89.56) | 1.95(4.41) |
| United States of America | 0(0,0.01) | 0.36(9.73) | 0.01(0,0.01) | 0.17(6.55) | 0.21(0.12,0.35) | 21.06(11.41) | 0.51(0.28,0.86) | 14.67(8.29) |
| United States Virgin Islands | 149.72(113.96,188.77) | 0.04(3.00) | 135.68(95.09,185.59) | 0.02(2.55) | 8833.77(6234.4,11932.99) | 2.14(3.87) | 11520.05(7609.76,16657.74) | 2.02(3.52) |
| Uruguay | 0.88(0.57,1.26) | 0.17(4.56) | 1.44(0.96,2.06) | 0.20(3.77) | 38.52(24.73,57.93) | 7.44(5.93) | 66.05(42.82,98.54) | 9.29(5.50) |
| Uzbekistan | 0.03(0.02,0.05) | 0.00(1.43) | 0.83(0.5,1.24) | 0.03(2.10) | 23.5(12.8,39.31) | 1.70(2.47) | 102.67(59.08,167.94) | 3.35(3.12) |
| Vanuatu | 0(0,0) | 0.00(2.11) | 0(0,0) | 0.00(1.69) | 0.06(0.03,0.11) | 0.92(6.30) | 0.15(0.08,0.26) | 0.76(4.20) |
| Venezuela (Bolivarian Republic of) | 3.5(2.22,5.21) | 0.31(5.13) | 5.84(3.32,9.4) | 0.15(2.97) | 129.47(79.65,199.34) | 11.54(5.98) | 292.33(169.71,466.44) | 7.67(3.64) |
| Viet Nam | 7.23(2.63,13.16) | 0.14(9.48) | 13.84(4.47,27.18) | 0.11(8.02) | 247.42(134.09,389.72) | 4.96(9.40) | 725.76(417.13,1139.41) | 5.98(8.07) |
| Yemen | 0.1(0.04,0.22) | 0.02(4.61) | 0.48(0.21,0.94) | 0.03(4.94) | 7.48(4.29,12.8) | 1.35(6.44) | 28.2(16.4,45.74) | 1.80(6.14) |
| Zambia | 0.04(0.01,0.23) | 0.01(5.14) | 0.03(0.01,0.22) | 0.00(3.18) | 6.86(3.62,12.32) | 2.22(6.66) | 13.13(6.86,22.73) | 1.82(4.41) |
| Zimbabwe | 0.73(0.32,1.35) | 0.15(6.71) | 1.1(0.49,2.05) | 0.14(5.76) | 27.97(15.84,46.23) | 5.93(7.87) | 40.33(22.19,66.69) | 5.21(6.43) |

**Abbreviations: ASR, age-standardised rate; DALY=disability-adjusted life-years; UI: uncertainly interval.**

**Table S12. Projected number of incident cases and age-standardised incidence rates of elderly-onset rheumatoid arthritis worldwide from 2022 to 2050 based on the BAPC model, by sex.**

| **Year** | **Both** | | **Female** | | **Male** | |
| --- | --- | --- | --- | --- | --- | --- |
|  | **Number (95 % UI)** | **ASRs (95 % UI)** | **Number (95 % UI)** | **ASRs (95 % UI)** | **Number (95 % UI)** | **ASRs (95 % UI)** |
| 2022 | 340576.22(336854.96,344297.47) | 30.2(29.87,30.53) | 225974.5(223295.39,228653.61) | 37.28(36.84,37.72) | 116800.67(115517.73,118083.61) | 22.4(22.15,22.64) |
| 2023 | 351895.74(346458.94,357332.54) | 30.29(29.82,30.76) | 233348.02(229488.38,237207.67) | 37.36(36.75,37.98) | 120777.63(118876.04,122679.21) | 22.48(22.13,22.84) |
| 2024 | 364137.94(356383.77,371892.11) | 30.38(29.74,31.03) | 241334.94(235877.32,246792.56) | 37.45(36.61,38.3) | 125055.79(122335,127776.59) | 22.57(22.08,23.06) |
| 2025 | 376946.46(366334.45,387558.47) | 30.48(29.62,31.33) | 249702.1(242254.38,257149.82) | 37.55(36.43,38.67) | 129509.91(125775.69,133244.12) | 22.65(21.99,23.3) |
| 2026 | 389924.13(375892.02,403956.23) | 30.57(29.47,31.67) | 258191.22(248383.64,267998.81) | 37.65(36.22,39.08) | 134003.31(129072.58,138934.04) | 22.72(21.88,23.56) |
| 2027 | 402719.91(384720.23,420719.59) | 30.65(29.28,32.02) | 266582.59(254008.74,279156.44) | 37.74(35.96,39.52) | 138406.73(132089.25,144724.2) | 22.78(21.74,23.82) |
| 2028 | 415626(393045.71,438206.28) | 30.74(29.07,32.41) | 275049.5(259347.79,290751.2) | 37.84(35.68,40) | 142827.26(134949.96,150704.56) | 22.85(21.59,24.11) |
| 2029 | 428937.21(401250.2,456624.22) | 30.83(28.84,32.82) | 283772.67(264477.31,303068.03) | 37.94(35.36,40.52) | 147375.2(137723.83,157026.58) | 22.9(21.4,24.4) |
| 2030 | 442402.03(409067.94,475736.12) | 30.92(28.59,33.25) | 292608.17(269310.42,315905.91) | 38.06(35.03,41.09) | 151949.91(140303.2,163596.63) | 22.96(21.2,24.72) |
| 2031 | 455739.96(416066.31,495413.6) | 31.02(28.32,33.72) | 301393.97(273676.93,329111) | 38.17(34.66,41.68) | 156445.09(142646.47,170243.71) | 23.02(20.99,25.05) |
| 2032 | 468698.55(421987.51,515409.59) | 31.11(28.01,34.21) | 309988.58(277353.09,342624.07) | 38.28(34.25,42.31) | 160758.18(144587.92,176928.44) | 23.06(20.74,25.38) |
| 2033 | 481407.82(427091.31,535724.32) | 31.2(27.68,34.72) | 318464.58(280559.14,356370.02) | 38.4(33.83,42.97) | 164937.63(146168.85,183706.41) | 23.11(20.48,25.74) |
| 2034 | 494104.59(431580.09,556629.09) | 31.29(27.33,35.25) | 326951.78(283236.54,370667.02) | 38.52(33.37,43.67) | 169084.53(147547.71,190621.36) | 23.16(20.21,26.11) |
| 2035 | 506770.04(435420.81,578119.27) | 31.39(26.97,35.81) | 335432.89(285436.29,385429.48) | 38.64(32.88,44.4) | 173196.24(148644.96,197747.52) | 23.21(19.92,26.5) |
| 2036 | 519353.35(438385.27,600321.43) | 31.49(26.58,36.4) | 343878.04(287116.83,400639.25) | 38.77(32.37,45.17) | 177254.87(149436.38,205073.36) | 23.26(19.61,26.91) |
| 2037 | 531821.78(440587.5,623056.06) | 31.59(26.17,37.01) | 352271.44(288251.23,416291.65) | 38.9(31.83,45.97) | 181246.86(149980.5,212513.21) | 23.3(19.28,27.32) |
| 2038 | 544193.9(441867.93,646519.87) | 31.7(25.74,37.66) | 360621.6(288840.57,432402.62) | 39.04(31.27,46.81) | 185177.78(150204.01,220151.55) | 23.35(18.94,27.76) |
| 2039 | 556556.78(442454.11,670659.45) | 31.8(25.28,38.32) | 368977.81(288821.35,449134.27) | 39.17(30.66,47.68) | 189084.27(150213.13,227955.42) | 23.4(18.59,28.21) |
| 2040 | 569041.75(442433.48,695650.02) | 31.91(24.81,39.01) | 377417.4(288425.83,466408.98) | 39.31(30.04,48.58) | 193018.66(149964.27,236073.04) | 23.45(18.22,28.68) |
| 2041 | 581759.95(441683.21,721836.69) | 32.02(24.31,39.73) | 386007.9(287525.38,484490.42) | 39.46(29.39,49.52) | 197022.37(149477.79,244566.94) | 23.5(17.83,29.17) |
| 2042 | 594835.41(440438.49,749232.33) | 32.13(23.79,40.47) | 394829.43(286241.94,503416.92) | 39.6(28.71,50.49) | 201140.59(148859.23,253421.96) | 23.55(17.43,29.67) |
| 2043 | 608548.23(438871.11,778225.35) | 32.24(23.25,41.23) | 404054.2(284652.39,523456) | 39.75(28,51.49) | 205473.16(148084.75,262861.58) | 23.59(17,30.18) |
| 2044 | 622882.64(436919.94,808845.34) | 32.36(22.7,42.02) | 413673.66(282717.83,544629.48) | 39.9(27.27,52.53) | 210013.86(147218.69,272809.02) | 23.65(16.58,30.72) |
| 2045 | 637554.53(434114.02,840995.04) | 32.47(22.11,42.83) | 423512.35(280286.01,566738.69) | 40.05(26.5,53.59) | 214657.96(146082.93,283232.99) | 23.7(16.13,31.27) |
| 2046 | 652270.26(430485.96,874054.56) | 32.59(21.51,43.67) | 433392.57(277216.29,589568.84) | 40.2(25.71,54.68) | 219299.58(144683.7,293915.46) | 23.75(15.67,31.83) |
| 2047 | 666744.12(425743.5,907744.74) | 32.7(20.88,44.52) | 443136.75(273383.75,612889.76) | 40.35(24.89,55.81) | 223833.04(142854.4,304811.68) | 23.8(15.19,32.41) |
| 2048 | 681289.31(420083.05,942495.56) | 32.82(20.24,45.4) | 452960.77(268919.07,637002.47) | 40.5(24.05,56.96) | 228360(140656.28,316063.72) | 23.85(14.69,33.01) |
| 2049 | 696141.69(413625.86,978657.53) | 32.94(19.57,46.31) | 463010.54(263888.46,662132.62) | 40.66(23.17,58.14) | 232965.95(138326.94,327604.95) | 23.9(14.19,33.61) |
| 2050 | 710903.01(406110.17,1015695.85) | 33.06(18.88,47.23) | 473003.86(258113.05,687894.67) | 40.81(22.27,59.35) | 237527.14(135562.79,339491.5) | 23.95(13.67,34.24) |

**Abbreviations: ASR, age-standardised rate; UI: uncertainly interval; BAPC: Bayesian age-period-cohort.**

**Table S13. Projected number of prevalent cases and age-standardised prevalence rates of elderly-onset rheumatoid arthritis worldwide from 2022 to 2050 based on the BAPC model, by sex.**

| **Year** | **Both** | | **Female** | | **Male** | |
| --- | --- | --- | --- | --- | --- | --- |
|  | **Number (95 % UI)** | **ASRs (95 % UI)** | **Number (95 % UI)** | **ASRs (95 % UI)** | **Number (95 % UI)** | **ASRs (95 % UI)** |
| 2022 | 8201253.71(8136188.09,8266319.33) | 727.28(721.51,733.05) | 5873708.42(5824490.37,5922926.48) | 969.05(960.93,977.17) | 2332077.41(2311842.43,2352312.39) | 447.17(443.29,451.05) |
| 2023 | 8476606.11(8373794.82,8579417.4) | 729.67(720.82,738.52) | 6067844.88(5991026.7,6144663.05) | 971.57(959.27,983.87) | 2410962.41(2379376.73,2442548.09) | 448.83(442.95,454.71) |
| 2024 | 8773112.41(8619706.9,8926517.92) | 732.02(719.22,744.82) | 6276593.19(6162543.8,6390642.57) | 974.1(956.4,991.8) | 2495787.84(2449185.19,2542390.48) | 450.39(441.98,458.8) |
| 2025 | 9082250.42(8867041.85,9297458.98) | 734.32(716.92,751.72) | 6493998.28(6335069.25,6652927.31) | 976.58(952.68,1000.48) | 2584091.83(2518900.26,2649283.39) | 451.88(440.48,463.28) |
| 2026 | 9395347.57(9107051.6,9683643.54) | 736.52(713.92,759.12) | 6713929.19(6501317.19,6926541.2) | 978.93(947.93,1009.93) | 2673440.97(2586740.26,2760141.67) | 453.28(438.58,467.98) |
| 2027 | 9704345.06(9332526.79,10076163.32) | 738.62(710.32,766.92) | 6930835.88(6656047.79,7205623.97) | 981.15(942.25,1020.05) | 2761454.39(2649683.72,2873225.07) | 454.6(436.2,473) |
| 2028 | 10014976.17(9547145.1,10482807.25) | 740.69(706.09,775.29) | 7148386.43(6803094.35,7493678.52) | 983.37(935.87,1030.87) | 2849836.99(2709796.17,2989877.81) | 455.84(433.44,478.24) |
| 2029 | 10333694.54(9757693.38,10909695.7) | 742.73(701.33,784.13) | 7370954.71(6946905.52,7795003.9) | 985.58(928.88,1042.28) | 2940526.77(2768732.3,3112321.24) | 457.01(430.31,483.71) |
| 2030 | 10654509.42(9959214.66,11349804.17) | 744.73(696.13,793.33) | 7594893.24(7083573.05,8106213.42) | 987.76(921.26,1054.26) | 3031572.13(2824445.91,3238698.35) | 458.12(426.82,489.42) |
| 2031 | 10971440.4(10145640.86,11797239.93) | 746.66(690.46,802.86) | 7816485.07(7208447.48,8424522.66) | 989.86(912.86,1066.86) | 3121101.08(2875037.02,3367165.15) | 459.16(422.96,495.36) |
| 2032 | 11278792.91(10308408.77,12249177.05) | 748.52(684.12,812.92) | 8032271.48(7318825.62,8745717.34) | 991.87(903.77,1079.97) | 3207238.01(2918682.5,3495793.52) | 460.15(418.75,501.55) |
| 2033 | 11578615.79(10452165.51,12705066.07) | 750.36(677.36,823.36) | 8243581.24(7414969.99,9072192.49) | 993.87(893.97,1093.77) | 3290524.25(2956539.85,3624508.65) | 461.09(414.29,507.89) |
| 2034 | 11876241.28(10581542.05,13170940.51) | 752.18(670.18,834.18) | 8453586.46(7501397.28,9405775.64) | 995.9(883.72,1108.07) | 3372764.92(2989482.41,3756047.43) | 461.98(409.48,514.48) |
| 2035 | 12171420.57(10694394.58,13648446.56) | 754(662.5,845.5) | 8662074.26(7576329.61,9747818.9) | 997.94(872.85,1123.03) | 3453958.06(3017407.89,3890508.23) | 462.85(404.35,521.35) |
| 2036 | 12463537.18(10792103.67,14134970.69) | 755.8(654.45,857.16) | 8868584.37(7639475.35,10097693.4) | 999.96(861.37,1138.55) | 3534007.36(3040895.97,4027118.74) | 463.69(398.99,528.39) |
| 2037 | 12752138.37(10872144.96,14632131.79) | 757.57(645.89,869.26) | 9072871.96(7690310.66,10455433.25) | 1001.95(849.27,1154.63) | 3612762.75(3058212.72,4167312.77) | 464.5(393.2,535.8) |
| 2038 | 13036909.09(10934745.62,15139072.57) | 759.34(636.9,881.78) | 9274806.14(7728708.41,10820903.86) | 1003.96(836.6,1171.32) | 3690012.56(3071428.89,4308596.22) | 465.29(387.29,543.29) |
| 2039 | 13319766.49(10981421.26,15658111.71) | 761.11(627.5,894.73) | 9475589.29(7755590.79,11195587.78) | 1006(823.39,1188.61) | 3766367.33(3078647.1,4454087.56) | 466.06(380.96,551.16) |
| 2040 | 13604076.23(11014666.1,16193486.36) | 762.9(617.69,908.11) | 9677378.92(7772516.63,11582241.22) | 1008.07(809.64,1206.49) | 3842924.14(3082269.23,4603579.06) | 466.82(374.42,559.22) |
| 2041 | 13892885.31(11036426.31,16749344.32) | 764.68(607.46,921.9) | 9882114.87(7780671.1,11983558.64) | 1010.14(795.33,1224.95) | 3920693.31(3083003.19,4758383.43) | 467.57(367.67,567.47) |
| 2042 | 14189383.91(11048541.5,17330226.31) | 766.46(596.8,936.12) | 10091917.8(7781239.44,12402596.16) | 1012.22(780.46,1243.98) | 4000648.67(3080630.76,4920666.57) | 468.31(360.62,576.01) |
| 2043 | 14499885.94(11055264.81,17944507.06) | 768.25(585.74,950.75) | 10311024(7777025.47,12845022.54) | 1014.31(765.04,1263.58) | 4084631.25(3076666.89,5092595.6) | 469.04(353.3,584.79) |
| 2044 | 14823908.75(11055297.65,18592519.85) | 770.04(574.28,965.81) | 10539148.72(7767172.21,13311125.23) | 1016.42(749.09,1283.76) | 4172455.42(3070723.56,5274187.27) | 469.77(345.73,593.81) |
| 2045 | 15154794.39(11042800.15,19266788.64) | 771.85(562.42,981.27) | 10771917.37(7747841.73,13795993.02) | 1018.56(732.61,1304.5) | 4262086.63(3061049.8,5463123.46) | 470.49(337.91,603.07) |
| 2046 | 15485793.16(11012248.4,19959337.93) | 773.66(550.16,997.16) | 11004866.4(7715391.02,14294341.78) | 1020.7(715.6,1325.8) | 4351494.62(3046021.49,5656967.76) | 471.21(329.85,612.58) |
| 2047 | 15810194.45(10958607.65,20661781.25) | 775.48(537.51,1013.44) | 11233561.82(7666519.3,14800604.35) | 1022.86(698.07,1347.66) | 4438650.68(3024160.81,5853140.54) | 471.94(321.54,622.33) |
| 2048 | 16135380.16(10886976.26,21383784.06) | 777.3(524.47,1030.14) | 11463449.87(7604907.42,15321992.32) | 1025.03(680.01,1370.05) | 4525521.54(2996829.42,6054213.66) | 472.66(313,632.32) |
| 2049 | 16466902.98(10800703.43,22133102.53) | 779.14(511.04,1047.23) | 11698197.89(7532670.06,15863725.73) | 1027.21(661.44,1392.98) | 4613786.99(2965056.61,6262517.37) | 473.38(304.22,642.54) |
| 2050 | 16795385.14(10693190.61,22897579.68) | 780.98(497.23,1064.72) | 11930737.41(7444914.38,16416560.44) | 1029.41(642.36,1416.45) | 4701005.93(2927164.64,6474847.21) | 474.1(295.2,652.99) |

**Abbreviations: ASR, age-standardised rate; UI: uncertainly interval; BAPC: Bayesian age-period-cohort.**

**Table S14.** **Projected number of deaths and age-standardised mortality rates of elderly-onset rheumatoid arthritis worldwide from 2022 to 2050 based on the BAPC model, by sex.**

| **Year** | **Both** | | **Female** | | **Male** | |
| --- | --- | --- | --- | --- | --- | --- |
|  | **Number (95 % UI)** | **ASRs (95 % UI)** | **Number (95 % UI)** | **ASRs (95 % UI)** | **Number (95 % UI)** | **ASRs (95 % UI)** |
| 2022 | 35521.09(34998.99,36043.19) | 3.15(3.1,3.2) | 22548.17(22196.61,22899.73) | 3.72(3.66,3.78) | 12725.09(12497.18,12952.99) | 2.44(2.4,2.48) |
| 2023 | 36129.17(35268.34,36989.99) | 3.11(3.04,3.18) | 22858.09(22294.76,23421.43) | 3.66(3.57,3.75) | 13053.27(12699.27,13407.26) | 2.43(2.36,2.5) |
| 2024 | 36793.35(35498.99,38087.71) | 3.07(2.96,3.18) | 23196.48(22365.28,24027.69) | 3.6(3.47,3.73) | 13354.62(12839.83,13869.41) | 2.41(2.32,2.5) |
| 2025 | 37475.97(35682.57,39269.38) | 3.03(2.88,3.17) | 23540.12(22396.36,24683.87) | 3.54(3.37,3.71) | 13724.54(13021.16,14427.92) | 2.4(2.28,2.52) |
| 2026 | 38141.81(35769.11,40514.51) | 2.99(2.8,3.18) | 23867.41(22372.27,25362.55) | 3.48(3.26,3.7) | 14037.26(13111.27,14963.24) | 2.38(2.22,2.54) |
| 2027 | 38627.06(35605.21,41648.9) | 2.94(2.71,3.17) | 24158.75(22265.61,26051.89) | 3.42(3.15,3.69) | 14396.55(13224.17,15568.93) | 2.37(2.18,2.56) |
| 2028 | 39211.28(35479.44,42943.11) | 2.9(2.62,3.18) | 24424.87(22105.96,26743.78) | 3.36(3.04,3.68) | 14691.78(13247.61,16135.95) | 2.35(2.12,2.58) |
| 2029 | 39791.38(35297.46,44285.31) | 2.86(2.54,3.18) | 24680.11(21905.47,27454.75) | 3.3(2.93,3.67) | 14991.8(13248.12,16735.48) | 2.33(2.06,2.6) |
| 2030 | 40201.2(34879.19,45523.21) | 2.81(2.44,3.18) | 24912.44(21644.61,28180.28) | 3.24(2.82,3.67) | 15286.31(13221.67,17350.96) | 2.31(2,2.62) |
| 2031 | 40702.22(34516.07,46888.37) | 2.77(2.35,3.19) | 25032.2(21241.83,28822.56) | 3.17(2.69,3.65) | 15565.93(13152.87,17978.99) | 2.29(1.94,2.64) |
| 2032 | 41135.85(34023.72,48247.98) | 2.73(2.26,3.2) | 25185.21(20852.7,29517.71) | 3.11(2.57,3.64) | 15821.76(13040.76,18602.77) | 2.27(1.87,2.67) |
| 2033 | 41354.61(33284.29,49424.93) | 2.68(2.16,3.2) | 25297.94(20404.24,30191.64) | 3.05(2.46,3.64) | 16056.94(12888.37,19225.51) | 2.25(1.81,2.69) |
| 2034 | 41683(32604.32,50761.68) | 2.64(2.06,3.22) | 25380.3(19896.8,30863.81) | 2.99(2.34,3.64) | 16280.38(12703.08,19857.68) | 2.23(1.74,2.72) |
| 2035 | 41808.71(31703.6,51913.83) | 2.59(1.96,3.22) | 25432.3(19347.64,31516.95) | 2.93(2.23,3.63) | 16491.9(12484.59,20499.2) | 2.21(1.67,2.75) |
| 2036 | 42050.63(30870.11,53231.15) | 2.55(1.87,3.23) | 25453.86(18748.94,32158.77) | 2.87(2.11,3.63) | 16614.88(12163.92,21065.84) | 2.18(1.6,2.76) |
| 2037 | 42250.56(29962.55,54538.57) | 2.51(1.78,3.24) | 25445.09(18101.33,32788.85) | 2.81(2,3.62) | 16799.83(11884.33,21715.34) | 2.16(1.53,2.79) |
| 2038 | 42235.22(28809.22,55661.21) | 2.46(1.68,3.24) | 25497.51(17506.44,33488.57) | 2.76(1.9,3.62) | 16971.4(11570.69,22372.11) | 2.14(1.46,2.82) |
| 2039 | 42350.99(27755.65,56946.33) | 2.42(1.59,3.25) | 25431.55(16775.4,34087.69) | 2.7(1.78,3.62) | 17132.4(11241.11,23023.68) | 2.12(1.39,2.85) |
| 2040 | 42440.52(26659.06,58221.97) | 2.38(1.49,3.27) | 25343.88(16012.72,34675.04) | 2.64(1.67,3.61) | 17205.29(10792.41,23618.17) | 2.09(1.31,2.87) |
| 2041 | 42513.56(25508.14,59518.99) | 2.34(1.4,3.28) | 25337.72(15359.16,35316.28) | 2.59(1.57,3.61) | 17357.54(10414.53,24300.56) | 2.07(1.24,2.9) |
| 2042 | 42394.36(24140.72,60648) | 2.29(1.3,3.28) | 25224.39(14456.66,35992.11) | 2.53(1.45,3.61) | 17512.55(10012.05,25013.05) | 2.05(1.17,2.93) |
| 2043 | 42466.47(22837.52,62095.41) | 2.25(1.21,3.29) | 25210.63(13723.53,36697.73) | 2.48(1.35,3.61) | 17590.99(9509.58,25672.39) | 2.02(1.09,2.95) |
| 2044 | 42544.26(21560.89,63527.63) | 2.21(1.12,3.3) | 25196.37(12961.1,37431.65) | 2.43(1.25,3.61) | 17763.84(9077.32,26450.35) | 2(1.02,2.98) |
| 2045 | 42606.78(20419.84,64793.72) | 2.17(1.04,3.3) | 25064.35(12056.27,38072.42) | 2.37(1.14,3.6) | 17845.81(8515.26,27176.37) | 1.97(0.94,3) |
| 2046 | 42634.66(19015.46,66253.86) | 2.13(0.95,3.31) | 25013.39(11320.72,38706.07) | 2.32(1.05,3.59) | 18007.55(8034.14,27980.95) | 1.95(0.87,3.03) |
| 2047 | 42610.23(17533.4,67687.07) | 2.09(0.86,3.32) | 24930.25(10433.36,39427.13) | 2.27(0.95,3.59) | 18152.01(7524.15,28779.86) | 1.93(0.8,3.06) |
| 2048 | 42761.83(16191.37,69332.28) | 2.06(0.78,3.34) | 24827.43(9506,40148.86) | 2.22(0.85,3.59) | 18287.57(6989.49,29585.64) | 1.91(0.73,3.09) |
| 2049 | 42692.39(14794.39,70590.39) | 2.02(0.7,3.34) | 24826.49(8768.99,40883.98) | 2.18(0.77,3.59) | 18323.51(6335.26,30311.77) | 1.88(0.65,3.11) |
| 2050 | 42581.15(13118.43,72043.86) | 1.98(0.61,3.35) | 24686.5(7765.24,41607.77) | 2.13(0.67,3.59) | 18443.24(5751.12,31135.37) | 1.86(0.58,3.14) |

**Abbreviations: ASR, age-standardised rate; UI: uncertainly interval; BAPC: Bayesian age-period-cohort.**

**Table S15.** **Projected number of DALYs and age-standardised DALY rates of elderly-onset rheumatoid arthritis worldwide from 2022 to 2050 based on the BAPC model, by sex.**

| **Year** | **Both** | | **Female** | | **Male** | |
| --- | --- | --- | --- | --- | --- | --- |
|  | **Number (95 % UI)** | **ASRs (95 % UI)** | **Number (95 % UI)** | **ASRs (95 % UI)** | **Number (95 % UI)** | **ASRs (95 % UI)** |
| 2022 | 1605026.68(1590141.65,1619911.7) | 142.33(141.01,143.65) | 1109672.51(1098883.33,1120461.69) | 183.07(181.29,184.85) | 494977.78(489710.43,500245.13) | 94.91(93.9,95.92) |
| 2023 | 1647584.02(1622839.61,1672328.44) | 141.82(139.69,143.95) | 1137486.75(1120062.14,1154911.36) | 182.13(179.34,184.92) | 509431.37(500890.35,517972.4) | 94.84(93.25,96.43) |
| 2024 | 1693526.1(1655773.97,1731278.24) | 141.31(138.16,144.46) | 1167479.09(1141189.74,1193768.44) | 181.19(177.11,185.27) | 524986.85(512075.53,537898.16) | 94.74(92.41,97.07) |
| 2025 | 1741465.24(1687910.46,1795020.01) | 140.8(136.47,145.13) | 1198718.52(1161745.91,1235691.13) | 180.27(174.71,185.83) | 541183.2(522998.18,559368.21) | 94.64(91.46,97.82) |
| 2026 | 1789772.01(1717698.01,1861846) | 140.3(134.65,145.95) | 1230114.09(1180733.24,1279494.95) | 179.36(172.16,186.56) | 557517.96(533100.21,581935.71) | 94.53(90.39,98.67) |
| 2027 | 1836611.43(1743591.17,1929631.69) | 139.79(132.71,146.87) | 1260462.86(1196887.21,1324038.51) | 178.44(169.44,187.44) | 573413.96(541948.08,604879.83) | 94.4(89.22,99.58) |
| 2028 | 1882961.95(1766409.81,1999514.08) | 139.26(130.64,147.88) | 1290341.25(1210851.37,1369831.12) | 177.51(166.57,188.44) | 589219.26(549770.26,628668.26) | 94.25(87.94,100.56) |
| 2029 | 1930113.1(1787345.51,2072880.7) | 138.73(128.47,148.99) | 1320581.93(1223488.12,1417675.73) | 176.58(163.59,189.56) | 605345.15(557023.94,653666.37) | 94.08(86.57,101.59) |
| 2030 | 1977176.51(1805663.31,2148689.7) | 138.2(126.21,150.19) | 1350687.26(1234282.33,1467092.18) | 175.66(160.53,190.8) | 621441.52(563340.3,679542.74) | 93.91(85.13,102.69) |
| 2031 | 2023095.72(1820278.18,2225913.26) | 137.68(123.88,151.49) | 1380048.7(1242635.36,1517462.04) | 174.77(157.36,192.17) | 637131.92(568384.2,705879.64) | 93.73(83.62,103.85) |
| 2032 | 2066619.87(1830023.94,2303215.79) | 137.15(121.45,152.85) | 1407932.36(1247857.3,1568007.42) | 173.86(154.09,193.63) | 651968.87(571707.22,732230.51) | 93.54(82.02,105.06) |
| 2033 | 2108051.4(1835262.18,2380840.63) | 136.61(118.93,154.29) | 1434522.65(1250171.16,1618874.13) | 172.95(150.72,195.18) | 666045.54(573436.24,758654.85) | 93.33(80.35,106.31) |
| 2034 | 2148434.95(1836958.74,2459911.16) | 136.07(116.34,155.8) | 1460411.5(1250131.02,1670691.98) | 172.05(147.28,196.82) | 679742.41(573919.2,785565.62) | 93.11(78.61,107.6) |
| 2035 | 2187896.81(1835228.2,2540565.42) | 135.54(113.69,157.38) | 1485669.67(1247802.19,1723537.16) | 171.16(143.76,198.57) | 693106.6(573197.09,813016.11) | 92.88(76.81,108.95) |
| 2036 | 2226393.03(1830008.92,2622777.14) | 135.01(110.97,159.05) | 1510265.92(1243144.12,1777387.73) | 170.29(140.17,200.41) | 706139.32(571259.25,841019.38) | 92.65(74.95,110.35) |
| 2037 | 2263721.09(1821101.86,2706340.32) | 134.48(108.19,160.78) | 1534087.27(1236042.84,1832131.69) | 169.42(136.5,202.33) | 718780.83(568042,869519.66) | 92.42(73.03,111.8) |
| 2038 | 2299776.21(1808470.76,2791081.66) | 133.95(105.33,162.57) | 1557083.82(1226492.93,1887674.71) | 168.55(132.76,204.33) | 730973.97(563516.24,898431.71) | 92.17(71.06,113.29) |
| 2039 | 2334930.9(1792446.2,2877415.59) | 133.42(102.42,164.42) | 1579474.14(1214694.42,1944253.86) | 167.69(128.96,206.42) | 742846.14(557795.25,927897.03) | 91.92(69.02,114.82) |
| 2040 | 2369846.43(1773541.1,2966151.76) | 132.9(99.46,166.34) | 1601662.14(1200961.01,2002363.27) | 166.84(125.1,208.58) | 754637.18(551057.44,958216.92) | 91.67(66.94,116.4) |
| 2041 | 2405081.25(1752121.98,3058040.51) | 132.38(96.44,168.32) | 1623988.24(1185519.52,2062456.95) | 166(121.18,210.82) | 766556.06(543434.49,989677.63) | 91.42(64.81,118.03) |
| 2042 | 2441115.21(1728443.86,3153786.57) | 131.86(93.36,170.36) | 1646750.59(1168535.12,2124966.06) | 165.17(117.2,213.13) | 778777.07(535012.64,1022541.5) | 91.16(62.63,119.7) |
| 2043 | 2478978.99(1703144.97,3254813.01) | 131.34(90.24,172.45) | 1670608.72(1150421.97,2190795.47) | 164.34(113.17,215.51) | 791637.9(525986.15,1057289.65) | 90.9(60.4,121.41) |
| 2044 | 2518558.67(1676005.19,3361112.15) | 130.83(87.06,174.6) | 1695497.2(1131051.76,2259942.64) | 163.52(109.08,217.95) | 805090.87(516268.63,1093913.1) | 90.64(58.13,123.16) |
| 2045 | 2558723.77(1646147.37,3471300.16) | 130.32(83.84,176.8) | 1720704.31(1109875.64,2331532.98) | 162.7(104.95,220.46) | 818746.9(505561.02,1131932.77) | 90.38(55.81,124.95) |
| 2046 | 2598338.32(1612775.1,3583901.53) | 129.81(80.57,179.05) | 1745522.78(1086402.7,2404642.87) | 161.9(100.76,223.03) | 832226.54(493603.43,1170849.65) | 90.12(53.45,126.79) |
| 2047 | 2636270.62(1575196.32,3697344.92) | 129.31(77.26,181.35) | 1769240.18(1060199.85,2478280.52) | 161.1(96.54,225.66) | 845142.33(480166.63,1210118.03) | 89.86(51.05,128.67) |
| 2048 | 2673762.08(1534217.53,3813306.63) | 128.81(73.91,183.7) | 1792725.96(1031829.03,2553622.89) | 160.3(92.26,228.34) | 857868.77(465484.62,1250252.91) | 89.6(48.62,130.58) |
| 2049 | 2711729.75(1490340.62,3933118.88) | 128.31(70.52,186.1) | 1816551.68(1001607.48,2631495.87) | 159.51(87.95,231.07) | 870722.71(449724.36,1291721.06) | 89.34(46.14,132.53) |
| 2050 | 2748628.11(1442692.28,4054563.94) | 127.81(67.08,188.54) | 1839618.63(968912.86,2710324.4) | 158.73(83.6,233.85) | 883244.88(432637.81,1333851.96) | 89.08(43.63,134.52) |

**Abbreviations: ASR, age-standardised rate; DALY=disability-adjusted life-years; UI: uncertainly interval; BAPC: Bayesian age-period-cohort.**

**
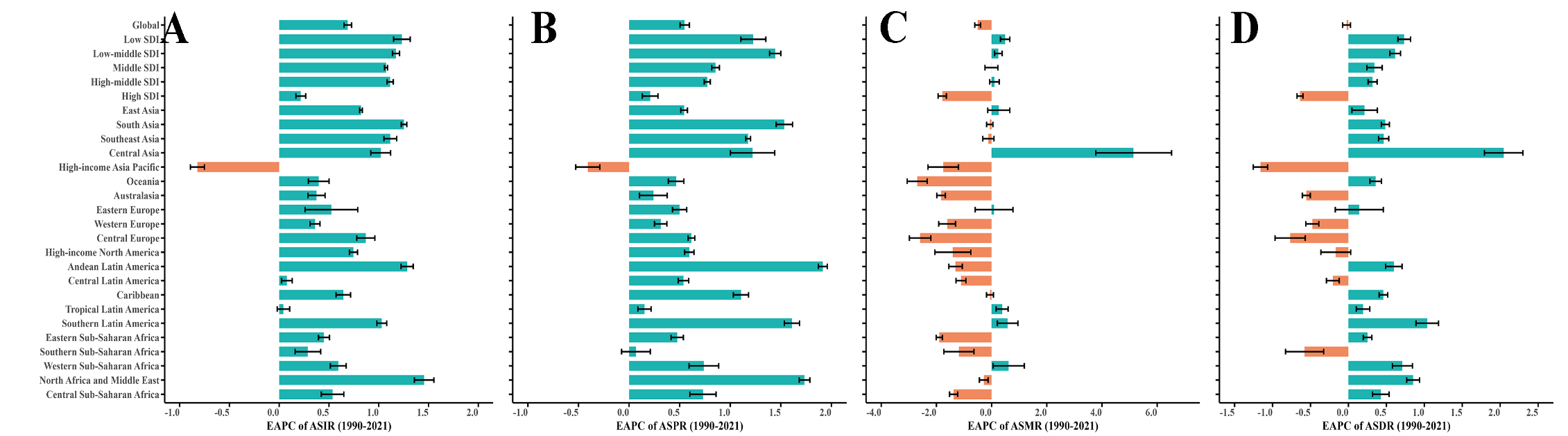
**

**Figure. S1.** **Estimated annual percentage change in age-standardised (A), prevalence (B), mortality (C), and DALYs (D) rates of elderly-onset rheumatoid arthritis globally and regionally, 1990-2021.**

**
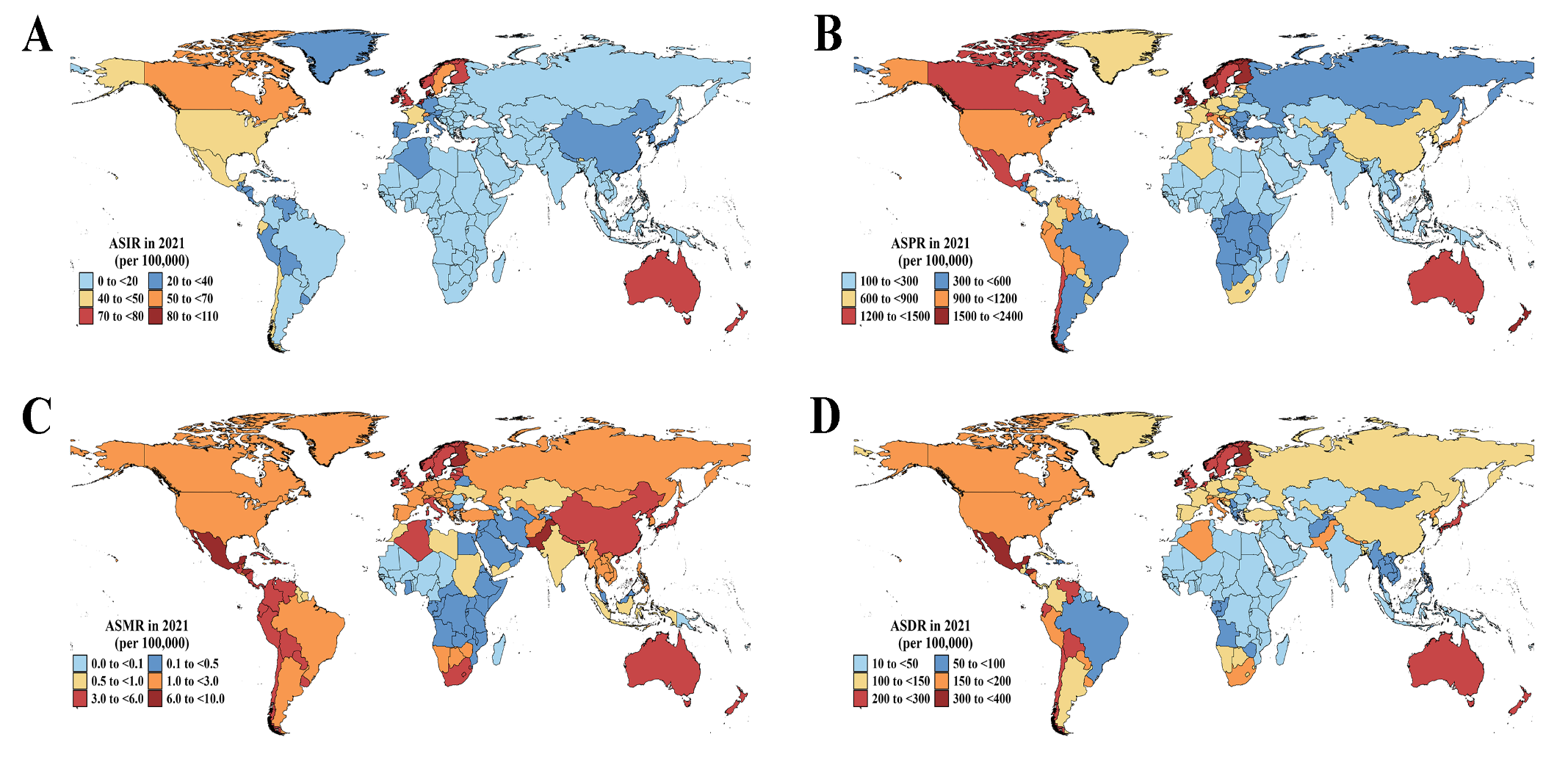
****Figure. S2.** **Age-standardised** **incidence (A), prevalence (B), mortality (C), and DALYs (D) rates of elderly-onset rheumatoid arthritis in 204 countries and territories in 2021.**

**
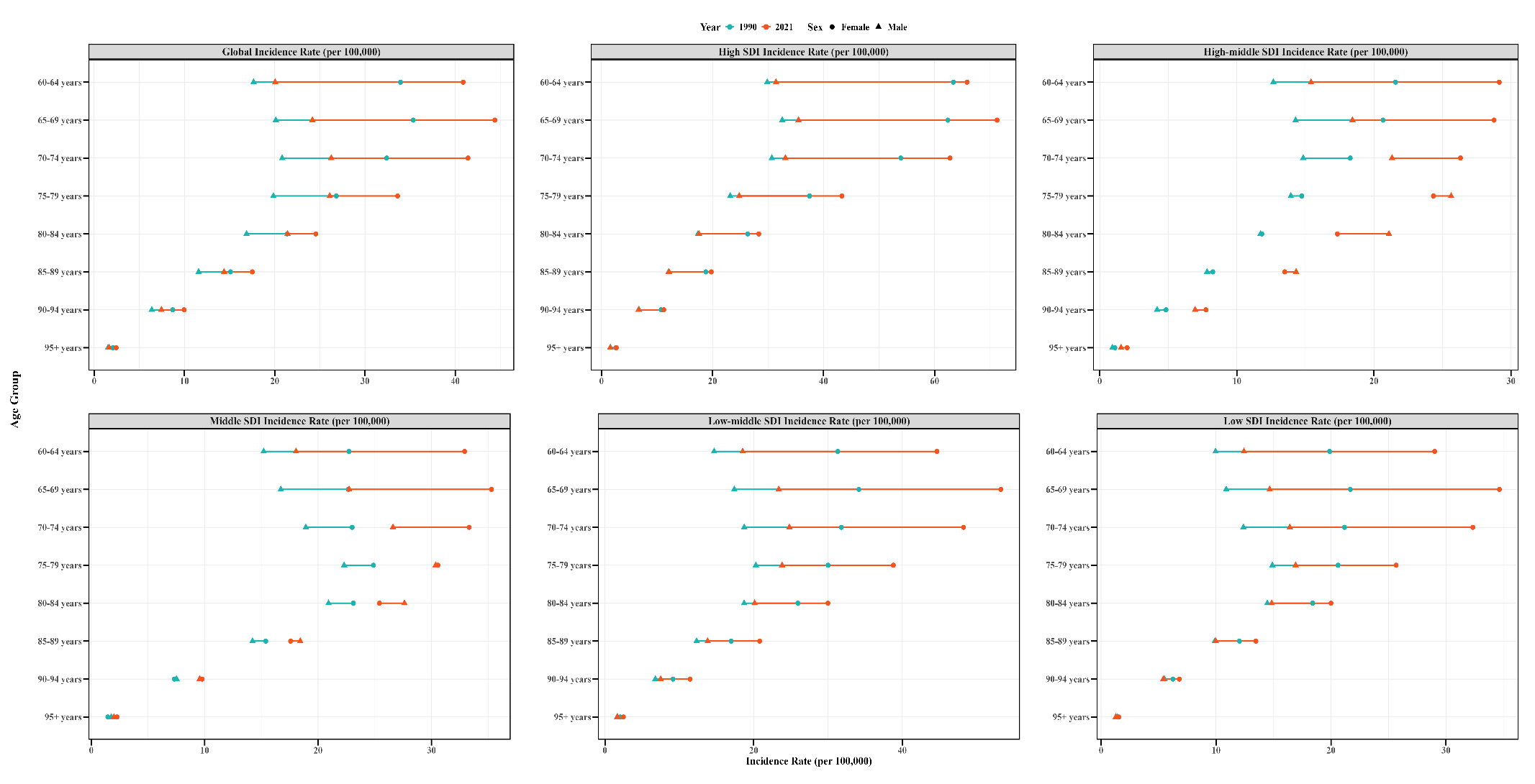
Figure. S3.** **Age-standardised incidence rates of elderly-onset rheumatoid arthritis by sex, age group, and socio-demographic index, 1990 and 2021.**

**
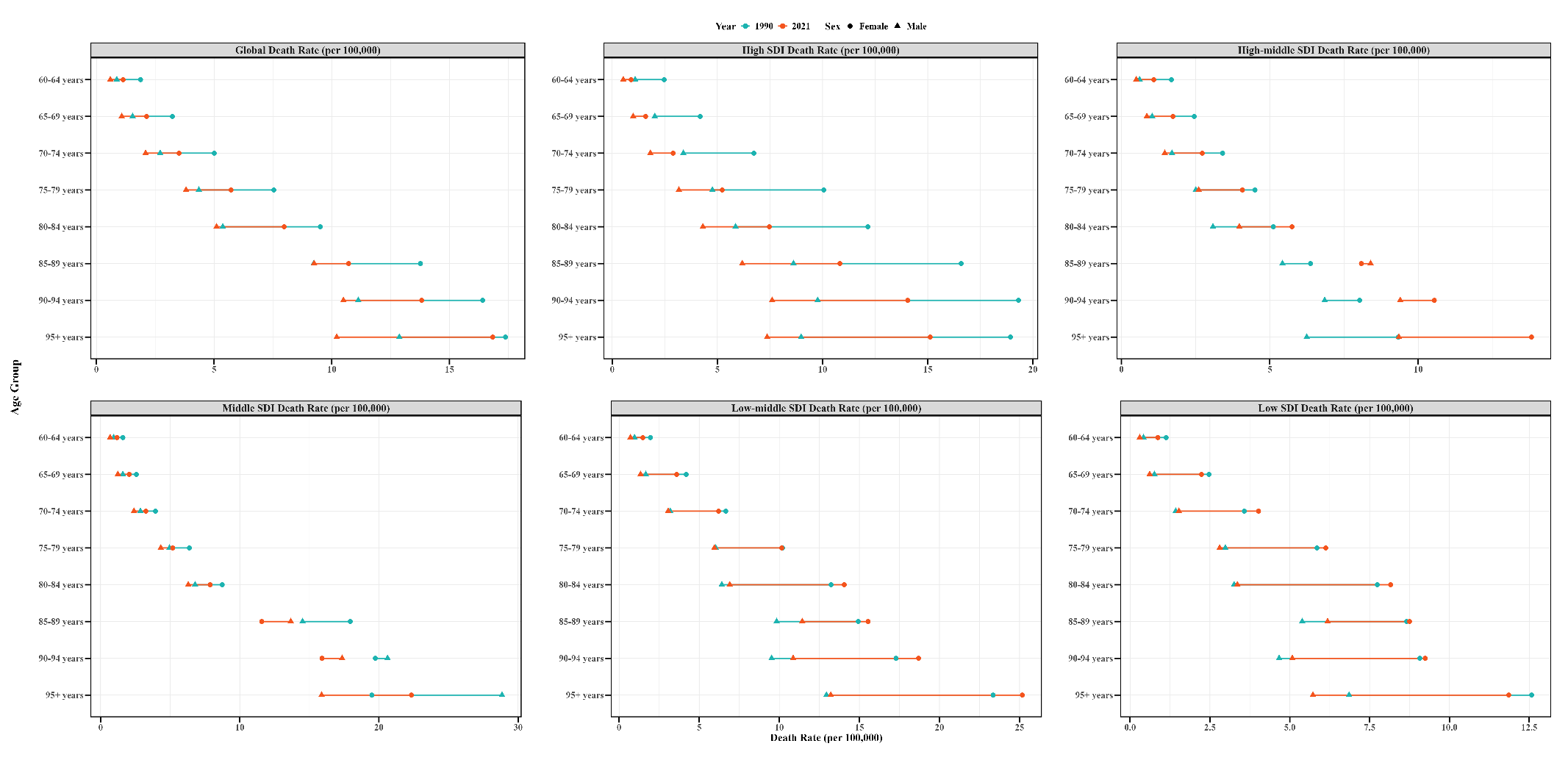
****Figure. S4.** **Age-standardised mortality rates of elderly-onset rheumatoid arthritis by sex, age group, and socio-demographic index, 1990 and 2021.**

**
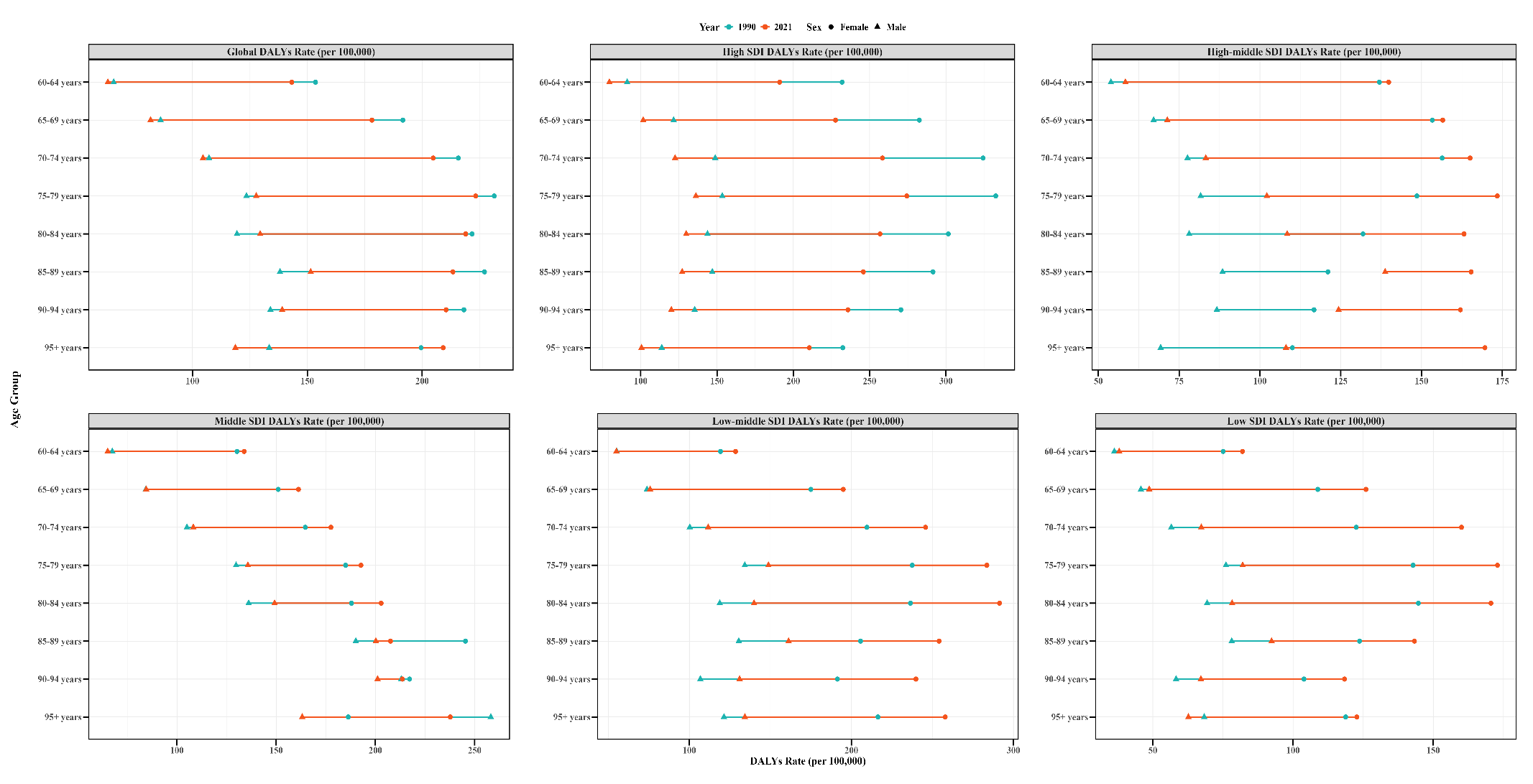
Figure. S5.** **Age-standardised DALYs rates of elderly-onset rheumatoid arthritis by sex, age group, and socio-demographic index, 1990 and 2021.**

**
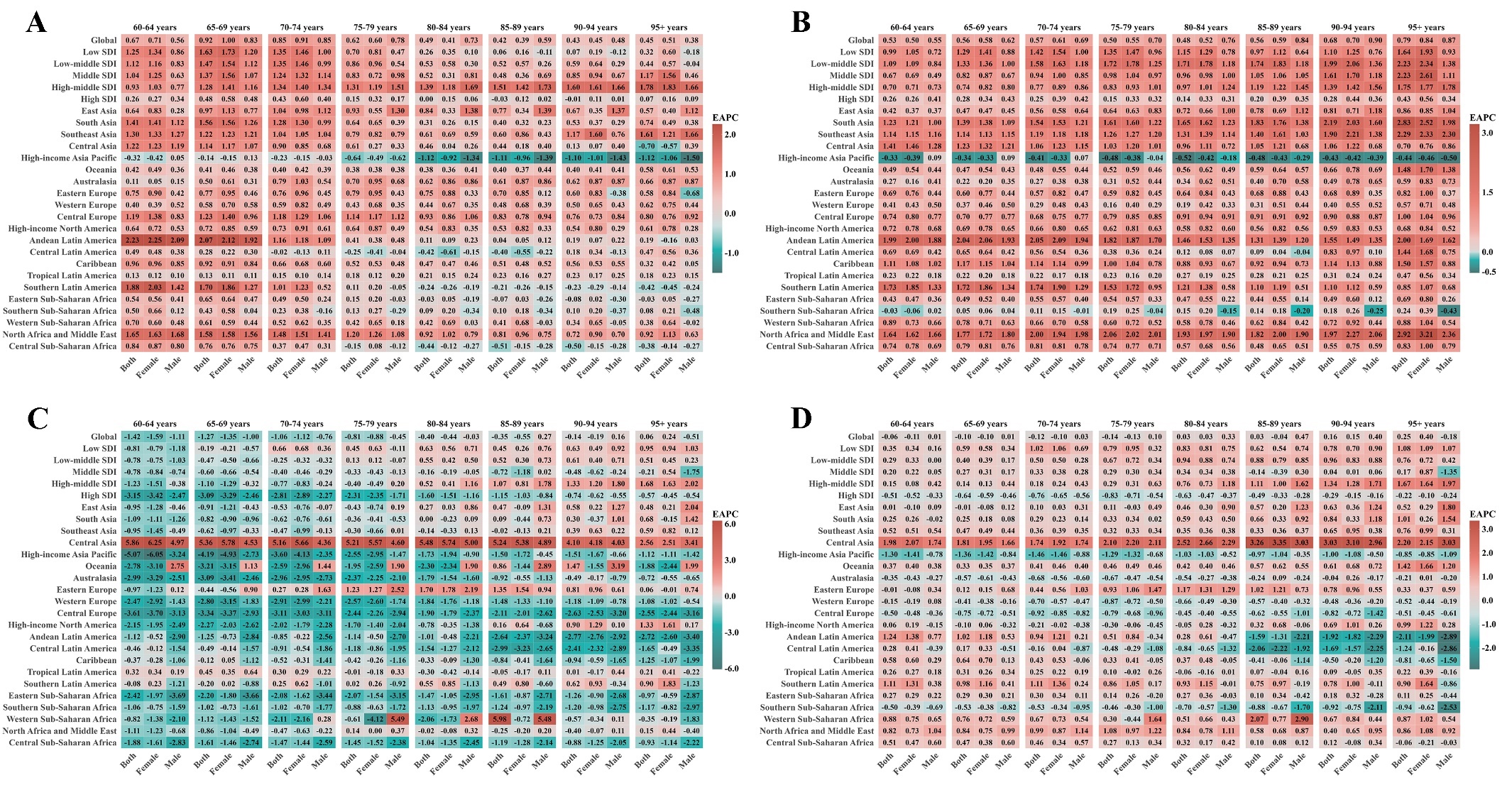
Figure. S6. Estimated annual percentage change in the age-standardized incidence (A), prevalence (B), mortality (C), and DALYs (D) rates of elderly-onset rheumatoid arthritis by sex and age group, globally and regionally.**

**
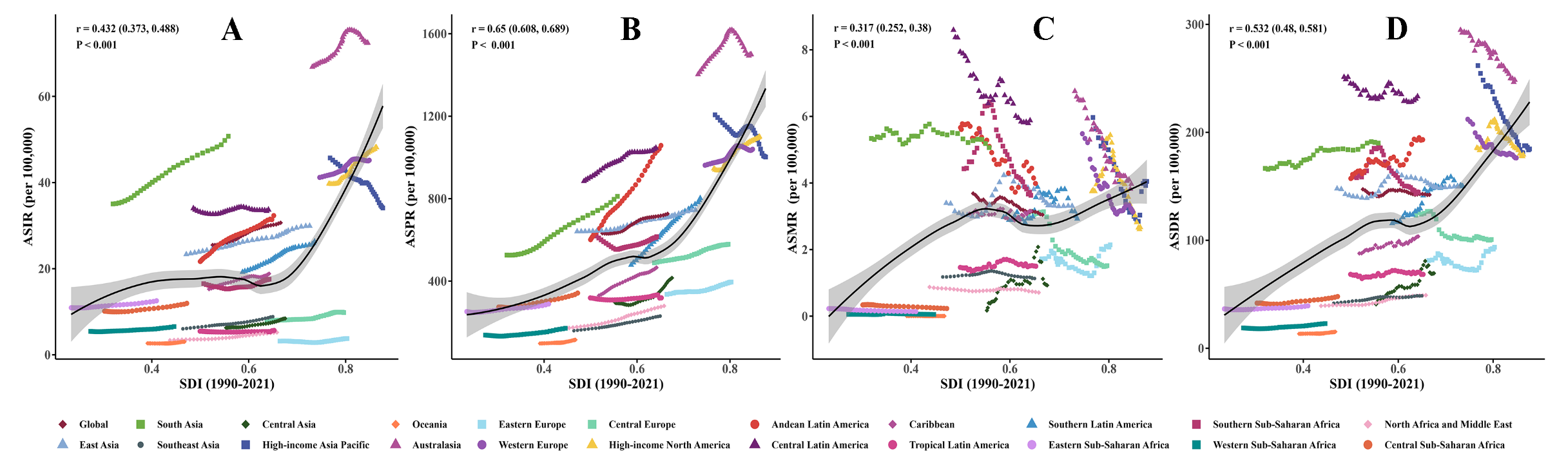
Figure. S7. Age-standardised incidence (A), prevalence (B), mortality (C), and DALYs (D) rates of elderly-onset rheumatoid arthritis, globally and for 21 GBD regions, by socio-demographic index, from 1990 to 2021.**

**Figure. S8. Age-standardised incidence (A), prevalence (B), mortality (C), and DALYs (D) rates of elderly-onset rheumatoid arthritis** **in 204 countries and territories in 2021, by socio-demographic index
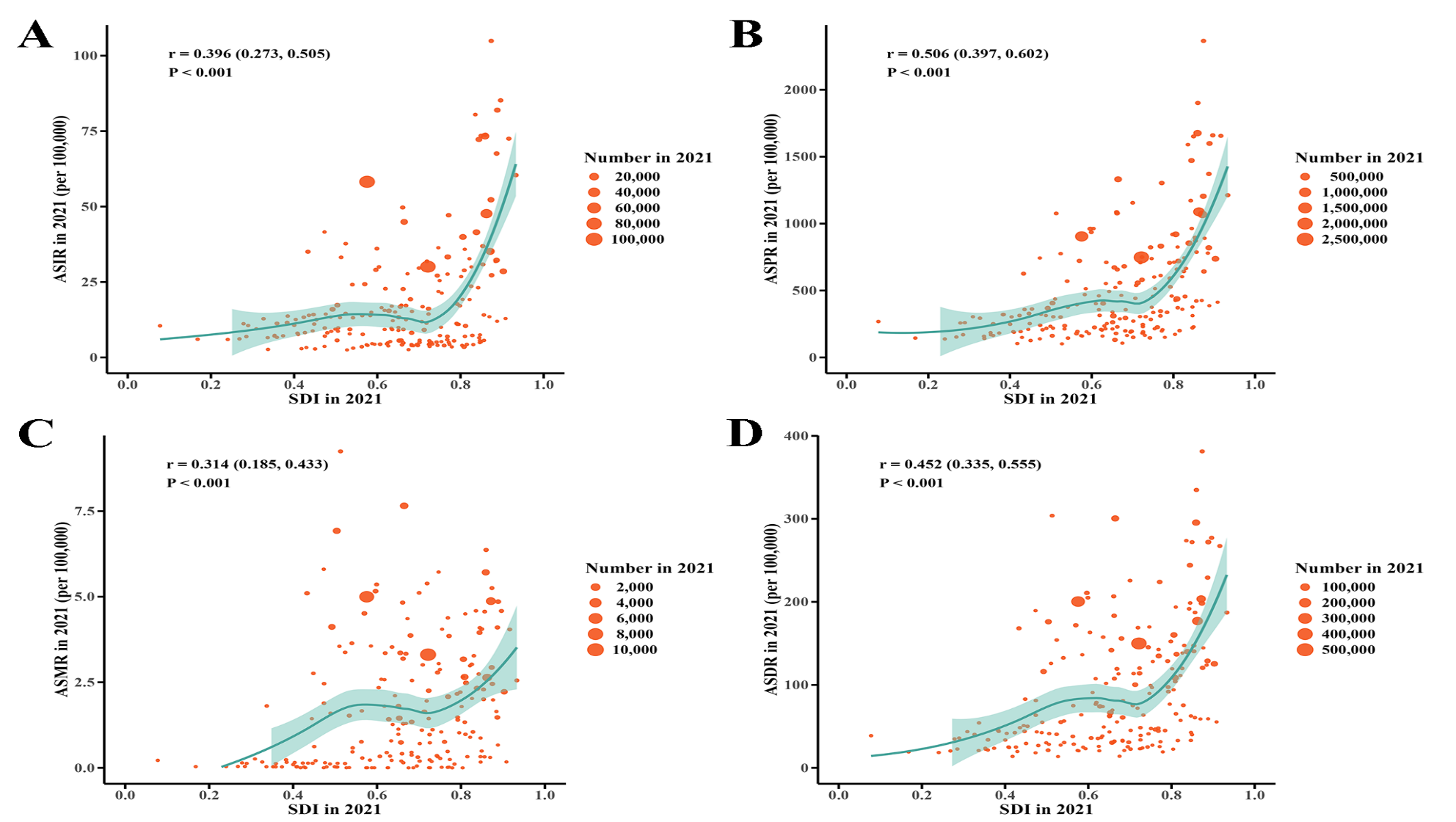
 in 2021.**

**
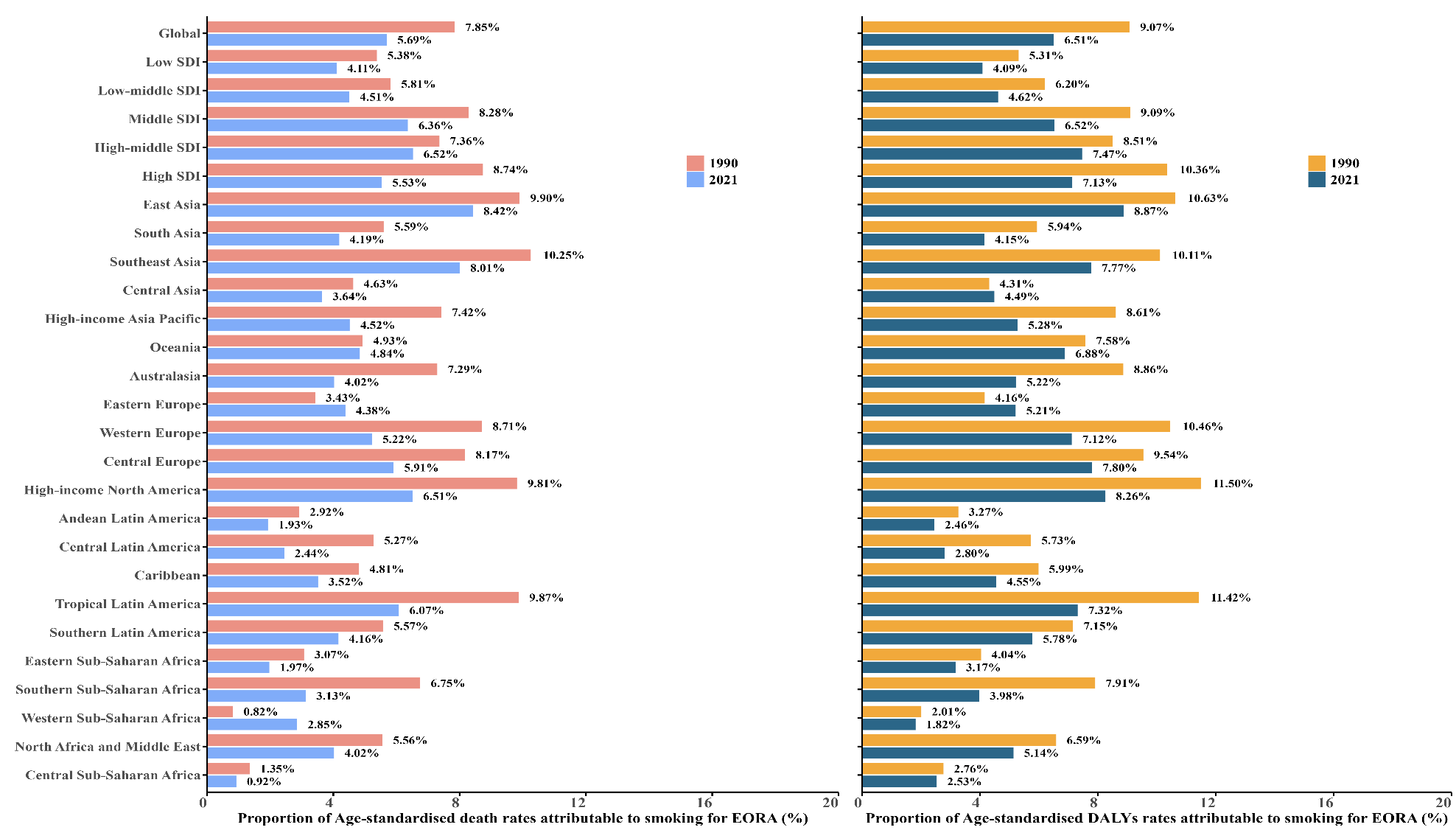
Figure. S9. Proportions and trends of age-standardised mortality and DALYs rates for elderly-onset rheumatoid arthritis attributable to smoking in 1990 and 2021, globally and regionally.**

**Figure. S10. Proportions and trends of age-standardised mortality (A, B) and DALYs (C, D) rates for elderly-onset rheumatoid arthritis attributable to smoking in 1990 and 2021, at the national level.
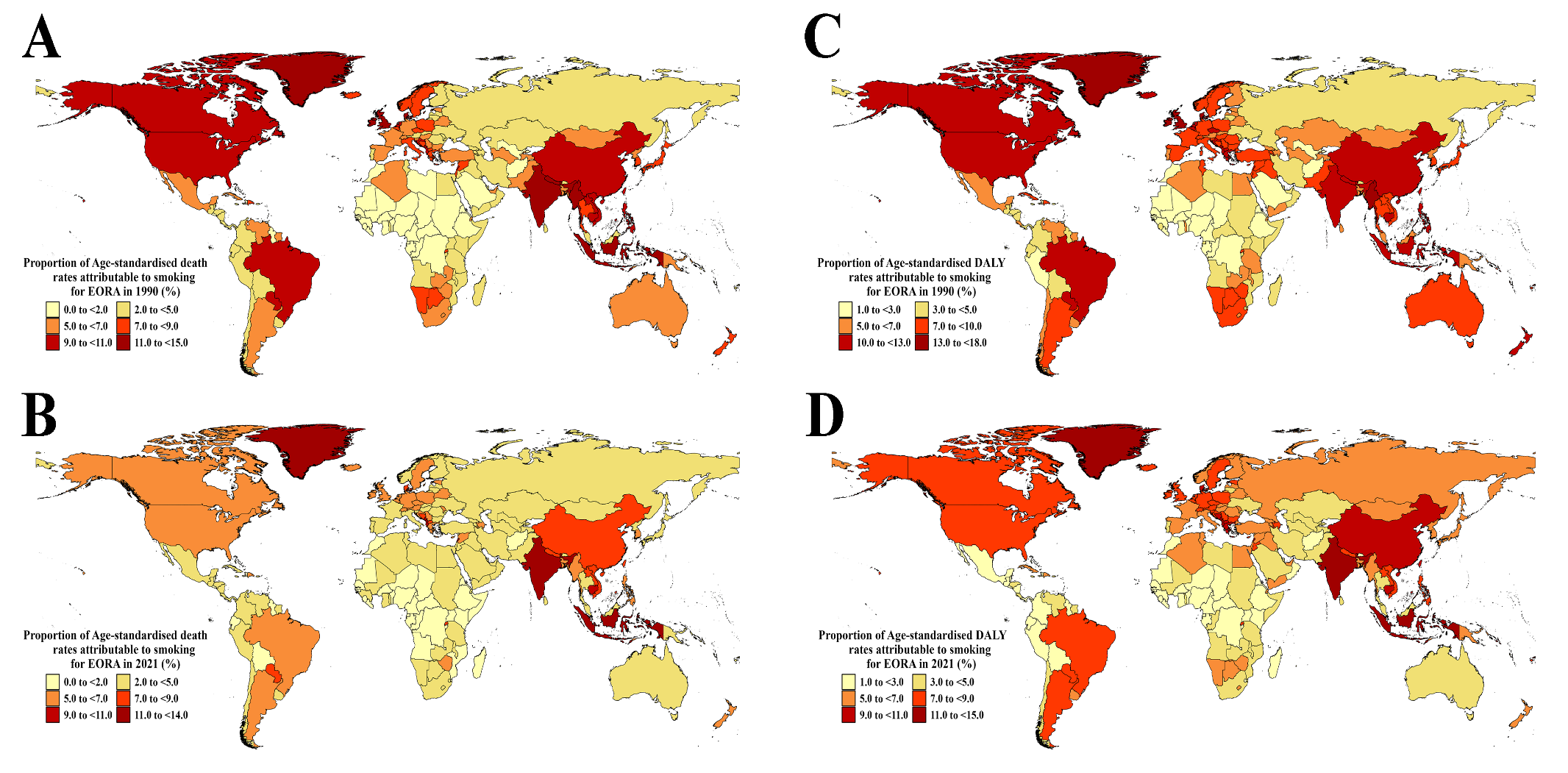
**
